# Exploring the Horizon: Anti-Fibroblast Growth Factor Receptor Therapy in Pancreatic Cancer with Aberrant Fibroblast Growth Factor Receptor Expression—A Scoping Review

**DOI:** 10.3390/cancers16162912

**Published:** 2024-08-22

**Authors:** Elena Orlandi, Massimo Guasconi, Stefano Vecchia, Serena Trubini, Mario Giuffrida, Manuela Proietto, Elisa Anselmi, Patrizio Capelli, Andrea Romboli

**Affiliations:** 1 Department of Oncology-Hematology, Azienda USL of Piacenza, 29121 Piacenza, Italy; s.trubini@ausl.pc.it (S.T.); m.proietto@ausl.pc.it (M.P.); e.anselmi@ausl.pc.it (E.A.); 2 Department of Medicine and Surgery, University of Parma, 43121 Parma, Italy; massimo.guasconi@unipr.it; 3 Department of Health Professions Management, Azienda USL of Piacenza, 29121 Piacenza, Italy; 4 Department of Pharmacy, Azienda USL of Piacenza, 29121 Piacenza, Italy; s.vecchia@ausl.pc.it; 5 Department of General Surgery, Azienda USL of Piacenza, 29121 Piacenza, Italy; m.giuffrida@ausl.pc.it (M.G.); p.capelli@ausl.pc.it (P.C.); a.romboli@ausl.pc.it (A.R.)

**Keywords:** FGFR, target therapy, pancreatic cancer, FGFR alteration, scoping review

## Abstract

**Simple Summary:**

Pancreatic cancer is one of the deadliest cancers due to its subtle onset and advanced stage at diagnosis. With most patients having inoperable cancer at diagnosis, survival rates remain low despite existing treatments. Our review aims to map and summarize current studies on FGFR inhibitors, a promising new treatment targeting specific cancer pathways. We found that while there are many preclinical studies, clinical research is still emerging, focusing mainly on the efficacy and safety of these treatments. Although FGFR alterations are relatively rare in pancreatic cancer, the few available real-life data are promising. Future research should aim to better identify the genetic drivers of this cancer and gather more data on long-term outcomes. This could lead to improved treatments and better survival rates for patients with pancreatic cancer.

**Abstract:**

Pancreatic cancer is a highly lethal disease, often diagnosed at advanced stages, with a 5-year overall survival rate of around 10%. Current treatments have limited effectiveness, underscoring the need for new therapeutic options. This scoping review aims to identify and summarize preclinical and clinical studies on FGFR (Fibroblast Growth Factor Receptor) inhibitors, including tyrosine kinase inhibitors (TKIs) and FGFR-specific inhibitors, in pancreatic cancer with FGFR alterations. We included studies analyzing efficacy, safety, and survival outcomes in various populations. A comprehensive search across major databases identified 73 relevant studies: 32 preclinical, 16 clinical, and 25 from gray literature. The clinical trials focused primarily on efficacy (20 studies) and safety (14 studies), with fewer studies addressing survival outcomes. FGFR1 was the most studied alteration, followed by FGFR2 and FGFR4. Although FGFR alterations are relatively rare in pancreatic cancer, the available data, including promising real-life outcomes, suggest significant potential for FGFR inhibitors. However, more extensive research is needed to identify the correct genetic drivers and gather robust survival data. Ongoing and future trials are expected to provide more comprehensive insights, potentially leading to improved targeted therapies for pancreatic cancer patients with FGFR alterations.

## 1. Introduction

Pancreatic cancer (PC) stands out as one of the deadliest forms of cancer, characterized by a subtle onset, aggressive behavior, and a 5-year overall survival rate hovering around 10%, primarily due to the majority of cases being diagnosed at an advanced stage, owing to the clinical characteristics of the disease and its often asymptomatic nature [1]. As a result, 80–90% of patients have unresectable cancer at the time of diagnosis [2]. PC represents a substantial challenge in the management of oncological diseases, ranking as the 12th most prevalent malignancy and the 7th leading cause of cancer-related mortality (accounting for 4.7% of all cancers) [1]. Chemotherapeutic regimens currently represent the standard of care, with median survival rates that seldom exceed one year from the initiation of first-line treatment [3]. Although some promising results have been observed with the use of the poly (ADP-ribose) polymerase (PARP) inhibitor olaparib in patients with germline BRCA mutations [4], the results of phase II/III trials investigating both targeted therapies and their combinations have failed to demonstrate a significant advantage [5,6,7,8].

The role of FGFR (Fibroblast Growth Factor Receptor) in tumor carcinogenesis pathways is integral to the regulation of key cellular processes. FGFR actively participates in signaling cascades governing cell growth, survival, and differentiation, positioning itself as a pivotal factor in the initiation and progression of tumorigenesis [9]. Dysregulation of FGFR signaling, often associated with genetic alterations such as mutations, amplifications, or overexpression, can instigate uncontrolled cell proliferation and contribute significantly to the pathogenesis of various cancers [10]. This aberrant activation of FGFR pathways leads to downstream effects on critical signaling pathways, including MAPK (Mitogen-Activated Protein Kinase) and PI3K/Akt (Phosphoinositide 3-Kinase/Protein Kinase B), resulting in enhanced cell survival, angiogenesis, and metastatic potential [11]. The pivotal role of FGFR in pancreatic cancer (PC) has been extensively explored. Elevated levels of FGFR expression have been associated with an advanced tumor stage, while lower FGFR expression has been significantly correlated with extended post-operative survival; compared to basic FGF expression in patients with PC, the overexpression of FGFR demonstrates itself as a more valuable prognostic indicator [12].

FGFR1, identified over two decades ago as abnormally expressed in PC, has been associated with aberrant autocrine and paracrine pathways [13]. In up to 10% of patients with advanced pancreatic ductal adenocarcinoma (PDAC), there is a description of amplification and overexpression of FGFR1 [14]. However, this occurrence is less common in cohorts of patients with resectable PDAC [15]. FGFR1 exhibited a broader expression pattern in both healthy and diseased pancreatic tissues; it demonstrated a robust correlation with the epithelial-to-mesenchymal transition (EMT) phenotype but did not show a significant association with the epithelial subtypes of PDAC, whether classical or basal-like [16]. Nuclear FGFR1 also regulates Neuregulin transcription, which acts in an autocrine fashion in pancreatic stellate cells, promoting invasion [17]. Distinct isoforms of FGFR1, such as FGFR1 IIIb and FGFR1 IIIc, exert varying influences on tumorigenesis, with IIIb suppressing tumor formation and growth in mice and IIIc promoting cell growth through mitogenic signaling via the FRS2-MAPK pathway, with the potential to increase the transformation of pancreatic ductal cells [18,19]. In only 0.54% of PDAC cases, FGFR2 is amplified, yet it does not exhibit mutations [20], while FGFR2 fusion has been identified in 6.7% of patients [21]. FGFR-2 and its isoforms clinically contribute to aggressiveness in PDAC [22]; previous studies demonstrated the correlation between the level of FGFR-2 IIIb and venous invasion and VEGF-A expression [23]; FGFR2 IIIc overexpression promoted cell proliferation and enhanced tumor growth and liver metastases in vivo [24].

Regarding the limited data showing that FGFR3 can exhibit both tumor-suppressive and oncogenic properties, it may be prudent to consider this information when evaluating the targeting of a TKI [25]. FGFR4 expression is exclusively detected in epithelial cells, significantly elevated in the classical/epithelial phenotype, and is associated with better outcomes [16]. It is substantially increased in high-grade pancreatic intraepithelial neoplasia (PanIN) and PDAC compared with normal and low-grade PanIN [26]. The stimulation of PDAC cells with FGFR4 significantly enhanced cell adhesion to laminin and fibronectin while concurrently reducing cell migration, with the upregulation of the integrin α4 family as the underlying cause of these effects, suggesting a potential tumor-suppressive function. Conversely, cell morphology and proliferation in PDAC cells remained unaffected [26]. Endogenous FGFR4 levels limit the malignant phenotype of PDAC, primarily by restraining mTORC1 pathway activity [16]. FGFR4-deficient cells showed increased activity in the MAPK and mTORC1 pathways. Downregulation of FGFR4 correlated with enriched PI3K/Akt/mTOR signatures and increased phosphorylation of 4E-BP1, a known mTORC1 substrate linked to cell proliferation [27]. Currently available therapeutic options targeting the FGF/FGFR-induced signaling cascade mainly include non-selective tyrosine kinase inhibitors (TKIs), selective TKIs (pan-FGFR, FGFR1/2/3, and FGFR4 inhibitors), monoclonal antibodies, and FGF ligand traps [28]. Even considering that the high heterogeneity of different tumor types significantly influences the efficacy of these drug classes, non-selective TKIs have generally shown non-significant effects in clinical trials for tumors with altered FGFR, despite dose-limiting toxicities such as hypertension [28]. Among selective TKIs, several drugs are in the early stages of trials for solid tumors with FGFR alterations. AZD4547, Debio-1347, and E7090 are FGFR1/2/3 inhibitors that have shown promising results in phase I and II studies [29,30,31]. Robitinib is an FGFR4 inhibitor with available phase I/II study results [32]. FP-1039 is an FGF ligand trap that has completed phase I trials [33]. The effectiveness of these drugs varies widely across different types of tumors, potentially due to tumor heterogeneity.

The identification of potential treatments for patients exhibiting FGFR pathway alterations in metastatic pancreatic cancer is of significant importance, given the limited therapeutic options available and the poor prognosis associated with this disease. In order to provide a comprehensive picture of treatment against pancreatic cancer with FGFR alteration, this scoping review aims to identify and present the available information concerning preclinical or clinical study and the results of anti-FGFR (TKI or FGFR-specific inhibitors) in pancreatic cancer harboring FGFR alterations, and regarding clinical study in terms of population analyzed, toxicity, and efficacy.

## 2. Materials and Methods

We prepared and elaborated this document according to the latest review process proposed in 2022 by the JBI [34], and the preferred reporting items for systematic reviews and meta-analyses extension for scoping reviews (PRISMA-ScR) checklist for reporting was used [35]. This scoping was registered prospectively with Figshare as suggested by JBI [34]: https://doi.org/10.6084/m9.figshare.26001772.

### 2.1. Research Question

The aim of this research was to identify the presence of available information concerning preclinical or clinical studies and the results of anti-FGFR (TKI or FGFR-specific inhibitor) in pancreatic cancer harboring FGFR alterations, and regarding clinical studies in terms of the population analyzed, toxicity, and efficacy.

### 2.2. Inclusion Criteria

Studies were included if they met the following population, concept, and context criteria:

Type of participants: We focused on patients with a diagnosis of pancreatic cancer disease with proven FGFR alteration. Regarding clinical studies, we included patients of any age and both genders who received various anti-FGFR treatments. We considered oncological outcomes such as progression-free survival (PFS), overall survival (OS), and recurrence rates. Articles were also eligible for inclusion if they performed a subgroup analysis considering the specific population with “FGFR mutation in pancreatic cancer”. Preclinical studies both in vivo and in vitro focusing on the targeted therapy against FGFR aberrations in pancreatic cancer were also included.

Concept: We considered any studies that included anti-FGFR treatment, both tyrosine kinase inhibitors (TKI), or FGFR-specific inhibitors.

Context: The studies focused on a target therapy directed toward dysregulation of FGFR signaling with aberrant activation of FGFR pathways.

Type of evidence source: This scoping review systematically considered randomized controlled trials, non-randomized controlled trials, before and after studies, and interrupted time-series studies. In addition, analytical observational studies, including prospective and retrospective cohort studies, case–control studies, and analytical cross-sectional studies, were considered for inclusion. This review also considered descriptive observational study designs, including case series, individual case reports, and descriptive cross-sectional studies, for inclusion. Text and opinion papers were also considered for inclusion in this scoping review. Gray literature was included as long as the research design was clearly recognizable. There were no date limitations or language restrictions. The reference lists of retrieved studies were reviewed to identify additional reports of relevant trials.

### 2.3. Exclusion Criteria

Studies that did not meet the specific above-stated inclusion criteria were excluded.

### 2.4. Search Strategy

A search was carried out on the online databases PubMed, Embase, Cochrane, Scopus, Web of Science, EBSCO, Open Dissertation databases, and the CENTRAL registry. An initial search strategy was launched in PubMed, then the strategy was supplemented and modified based on the initial results obtained. The search strategy used for PubMed is reported in the Supplementary Materials, and the strategies used for the other databases were derived from that for PubMed. Additionally, we included ongoing studies, as well as studies listed on ClinicalTrials.gov, even if a full publication was not available; the keyword selected for the conditions was “FGFR”. The search was initially conducted in September 2023 and was updated periodically to ensure the inclusion of the most recent literature. The most recent search was executed on 1 June 2024.

### 2.5. Study Selection

**Figure 1:**
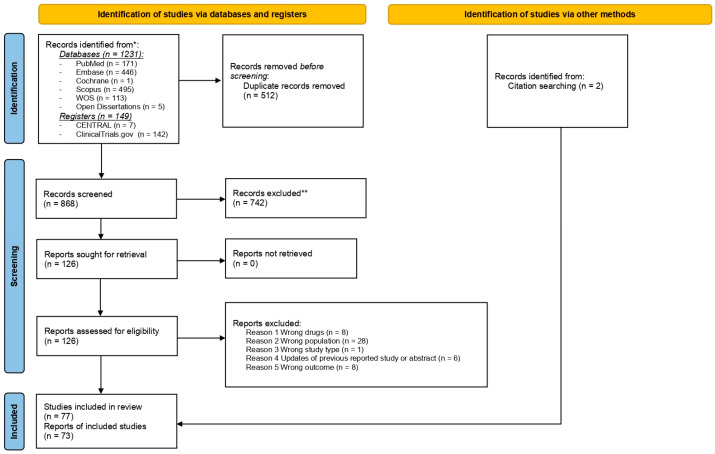
PRISMA 2020 flow diagram. * Consider, if feasible to do so, reporting the number of records identified from each database or register searched (rather than the total number across all databases/registers). ** If automation tools were used, indicate how many records were excluded by a human and how many were excluded by automation tools [36].

After removing duplicates, two researchers independently reviewed the titles and abstracts of the studies to determine which ones to include. The review process consisted of two levels of screening using Rayyan QCRI online software: (1) a title and abstract review and (2) a full-text review. For both levels, two authors independently screened the articles, with conflicts resolved by a third author. The reasons for exclusion were recorded and presented in the PRISMA flow diagram (Figure 1).

### 2.6. Data Extraction

For each outcome, the information from the included texts was extracted into a dedicated data form. The data form was reviewed by the research team and pretested by all reviewers before implementation to ensure accuracy. The collected data included the following information: title; authors; publication year; country in which the study was performed for the clinical trial; study design; baseline characteristics of enrolled patients, including performance status, type of anti-FGFR drugs, type of FGFR, concept and context; and key findings relevant to the scoping review research questions.

### 2.7. Data Synthesis

The analysis and presentation of results followed the JBI guidelines for scoping reviews. The results were also displayed using charts and figures. The most effective method for presenting data was determined based on the findings at the conclusion of the study.

## 3. Results

Out of the 1380 studies found during the initial literature search, 73 were included in the final analysis. The primary reasons for excluding the 742 papers during the screening phase were the wrong population, wrong drug, wrong publication type, and wrong outcomes, as detailed in Supplementary Material Table S2 (reason for exclusion). The details of the excluded studies, along with their references, are also specified in the Supplementary Materials. A summary of the study selection process is illustrated in the PRISMA flow diagram (Figure 1).

The majority of studies reported in the literature were preclinical (n = 32): 16 were clinical studies and 25 were studies from gray literature (Table 1).

Regarding the trials (n = 12), five were phase I studies, two were phase II studies, one was a phase I/II basket trial, and four were phase II basket trials (references in Tables S3 and S4 in Supplementary Materials).

The studies selected from clinicaltrial.gov included 25 trials, of which 4 were phase I studies, 1 was a phase I/II study, 17 were phase II studies, 1 was a phase III study, and 1 was defined as a post-market study (expanding access program) (NCT numbers in Supplementary Materials).

### 3.1. Study Distribution Over Time

**Figure 2:**
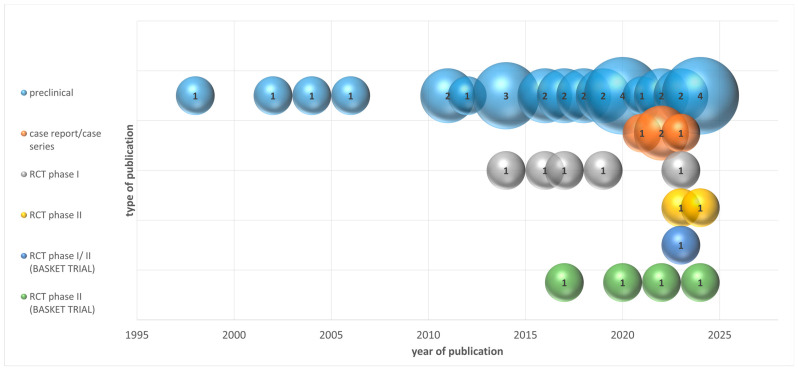
Distribution of studies by type of publication and year of publication.

The studies were published over a span of 25 years, with an increasing trend in the number of publications over time. The periods of publication are detailed in Figure 2, with the majority of studies (18) being published between 2018 and 2022.

### 3.2. Preclinical Studies

**Figure 3:**
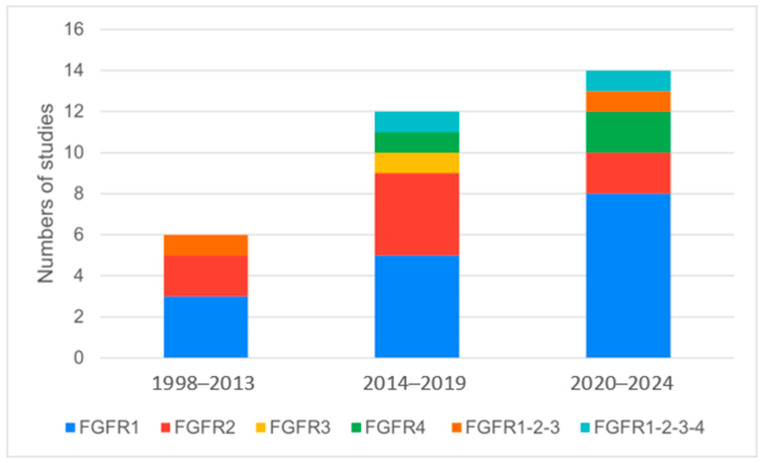
Classification of preclinical studies on specific FGFR alteration, based on the years of publication.

In the preclinical setting, 32 studies focused on various FGFR alterations in pancreatic cancer. FGFR1 was the most frequently studied alteration, being the focus of 16 studies, followed by FGFR2 (8 studies), FGFR3 (1 study), FGFR4 (3 studies), and combinations of FGFR alterations (4 studies) (references in Table S5 in Supplementary Materials, Table 2, Figure 3).

### 3.3. Clinical Studies

The clinical studies included 16 studies examining FGFR alterations and their impact on pancreatic cancer treatment. Various selective FGFR inhibitors were investigated, including Erdafitinib (three studies), Pemigatinib (four studies), and Futibatinib (two studies), as well as non-selective TKIs like Pazopanib, Ponatinib, and Dovitinib (one study each). The studies primarily focused on the efficacy (five studies) and safety (eight studies) of these treatments, with a smaller subset examining survival outcomes (one study) (references in Tables S3 and S4 in Supplementary Materials, Table 1).

### 3.4. Gray Literature

A total of 25 studies were identified from gray literature, primarily sourced from clinicaltrial.gov. These studies often included both adult (22 studies) and pediatric populations (3 studies). The status of these studies varied, with 15 being active but not recruiting, 9 closed or terminated, and 1 approved for marketing (Table 3).

The studies assessed FGFR alterations generically (21 studies) and specifically FGFR2 expression or overexpression (3 studies). The types of drugs investigated included selective inhibitors pan-FGFR (11 studies) and non-selective TKIs (1 study) (NCT numbers in Supplementary Materials).

### 3.5. Clinical Outcomes

The primary clinical outcomes assessed in the studies included safety, efficacy, and survival. In the gray literature, 20 studies reported on efficacy, 6 on safety, and 2 on survival outcomes. Among the clinical studies, eight reported on safety, five on efficacy, and one on survival outcomes (references in Tables S3 and S4 in Supplementary Materials, Table 1).

### 3.6. Synthesis of Results

The review identified 73 relevant studies, including 32 preclinical and 41 clinical studies, focusing on FGFR inhibitors in pancreatic cancer with FGFR alterations. Clinical studies primarily assessed efficacy, with 20 studies, and safety, with 14 studies, while only a few focused on survival outcomes. The majority of trials involved adult populations, with manageable toxicity profiles documented. Ongoing trials are expected to provide more comprehensive data on the efficacy and survival outcomes of FGFR-targeted therapies in this patient population.

## 4. Discussion

In the present scoping review, we mapped and summarized the current literature reporting epidemiological data on FGFR inhibitors against FGFR alterations in pancreatic cancer. Considering the current lack of a comprehensive overview in this field, conducting a scoping review is the most effective and suitable approach to systematically map the existing literature and provide a broad understanding of anti-FGFR therapy in pancreatic cancer with FGFR alterations.

FGFR1 alteration was the most frequently studied FGFR alteration in preclinical studies, followed by FGFR2, with an increase in evaluations of FGFR4 alterations in the past four years (Figure 3). The review highlighted the limited number of studies specifically dedicated to pancreatic cancer, reflecting the relatively low percentage of FGFR alterations in this cancer type [12]. Various FGFR inhibitors have demonstrated promising efficacy in clinical settings. Specific inhibitors such as Erdafitinib, Pemigatinib, and Futibatinib have been evaluated in multiple clinical trials, showcasing significant antitumor activity in patients with advanced pancreatic cancer harboring FGFR alterations [37,38,39,40]. These studies have reported the Overall Response Rate (ORR) and PFS benefits in a subset of patients, suggesting that FGFR inhibitors can provide a targeted therapeutic option. Ongoing clinical trials continue to explore the full potential of these inhibitors, including their use in combination with other therapeutic agents, to enhance treatment outcomes and overcome resistance mechanisms.

The safety profiles of FGFR inhibitors have been well documented across several clinical studies. Common adverse events associated with these agents include hyperphosphatemia, stomatitis, fatigue, and gastrointestinal disturbances [33,38,39,41,42,43,44]. These side effects are generally manageable with dose adjustments and supportive care. However, while the safety data are robust, there is a need for more extensive studies to fully understand the long-term impact of FGFR inhibitors on survival outcomes. Current evidence suggests that while these agents can provide disease control, their effect on OS remains to be conclusively determined. The ongoing clinical trials primarily emphasize evaluating the efficacy of FGFR inhibitors, with a significant number of studies focusing on this aspect. Notably, two studies have designated survival as the primary outcome, highlighting a crucial interest in understanding the long-term benefits of these treatments. Therefore, we anticipate that future results from these trials will provide more comprehensive and robust data on both the efficacy of FGFR inhibitors and their impact on survival outcomes in patients with pancreatic cancer.

The promising real-life data from the case reports and case series identified underscore the potential of FGFR inhibitors in treating pancreatic cancer, despite the relatively low frequency of FGFR alterations in this disease.

The preclinical studies provided further insights into the significance of FGFR alterations in pancreatic cancer. FGFR1 was the most frequently studied receptor alteration, being the focus of 16 preclinical studies, followed by FGFR2 (8 studies), FGFR3 (1 study), and FGFR4 (3 studies). Additionally, combinations of FGFR alterations were investigated in four studies. These preclinical findings underline the critical role of FGFR1 and FGFR2 in the pathophysiology of pancreatic cancer.

### 4.1. Research Implications

Given the current landscape of research, our scoping review highlights significant gaps and challenges in the literature concerning anti-FGFR therapy for pancreatic cancer with FGFR alterations. While numerous preclinical studies exist, there is a notable scarcity of clinical trials, most of which have been conducted in recent years. This trend is further supported by the increasing number of ongoing trials.

Many of these trials have been withdrawn or terminated, often due to issues such as low enrollment rates or the termination of agreements with drug suppliers. Additionally, the majority of trials are basket trials, which lack a specific focus on pancreatic cancer, thereby limiting the generalizability of their findings to this particular patient population.

Despite the generally unremarkable ORR observed, the few available real-life observational data indicate significantly promising outcomes. To advance the field, future research should aim to address these gaps by conducting more targeted trials specifically on pancreatic cancer, ensuring adequate enrollment, and fostering collaborations to maintain continuity with drug suppliers. Such efforts will be crucial in enhancing the applicability and impact of anti-FGFR therapies for patients with pancreatic cancer.

### 4.2. Strengths and Limitations

This is the first scoping review to encompass such a wide range of studies reporting on anti-FGFR therapies for pancreatic cancer with FGFR alterations. This review includes both preclinical and clinical studies, identifying the volume and distribution of the existing evidence base. We have also mapped the key concepts and research priorities within this literature.

The strength of this study lies in its methodology. To capture the highest possible number of relevant studies, we employed a comprehensive search strategy incorporating various terms. Additionally, the search was conducted across major databases without any restrictions. In line with the objectives of scoping reviews, the inclusion criteria were intentionally broad, allowing us to gather findings from diverse sources. Moreover, to ensure completeness and transparency in reporting, the PRISMA-ScR checklist was utilized.

However, there are some limitations to consider. Firstly, many trials were withdrawn or terminated, often due to low enrollment or discontinued partnerships with drug suppliers. Additionally, most of the trials were basket trials, which do not focus specifically on pancreatic cancer, thus limiting the generalizability of the results to this specific population. Although we followed a rigorous approach and included various types of publications, this strategy may have excluded findings from studies addressing other research questions.

## 5. Conclusions

This scoping review provides a comprehensive overview of the available preclinical and clinical studies on FGFR inhibitors in pancreatic cancer harboring FGFR alterations. The results indicate that while there are a substantial number of preclinical studies (32) exploring the underlying mechanisms and potential therapeutic targets, clinical studies (41) are still emerging, with a significant focus on drug efficacy (20 studies) and safety (14 studies). Only a few studies have thus far reported on survival outcomes, highlighting a gap in long-term efficacy data. These findings underscore the potential of FGFR inhibitors as a targeted therapy for pancreatic cancer patients with FGFR alterations, despite the relatively low incidence of these alterations in this cancer type. More extensive research is needed to better identify the correct genetic drivers and to gather robust data on survival outcomes. Future studies should focus on optimizing FGFR-targeted therapies, exploring combination treatments, and validating biomarkers for patient selection to maximize therapeutic benefits.

In summary, while the current data are encouraging, continued efforts in clinical research are essential to fully understand and harness the potential of FGFR inhibitors in pancreatic cancer treatment.

## Figures and Tables

**Table 1:** Population characteristics: (total number of studies = 73).

Variables	Numbers of Studies
Type of studies	
Preclinical	32
Clinical	16
Gray literature	25
Abstract ^a^	4
Sample size	
Only one case	7
1–10 patients	7
>10	2
Included only patients with pancreatic cancer ^b^	5
FGFR alterations	
FGFR alteration generic	9
FGFR2 fusion ^c^	4
FGFR 2 amplification/rearrangement	3
Type of drugs	
Selective inhibitors pan-FGFR	
Erdafinitinib	3
Pemigatinib	4
Futibatinib	2
Selective inhibitors FGFR 1/2/3	
Lirafugratinib	1
Fexagratinib	2
Monoclonal antibodies	
Aprutumab ixadotin	1
FGF ligand traps	
FB-1039	1
Non-selective TKIs	
Pazopanib	1
Ponatinib	1
Dovitinib	1
Clinical outcomes (primary)	
Safety	8
Survival outcomes	1
Efficacy	5

^a^ included in clinical studies; ^b^ 2 abstract congress presentation and 3 case reports; ^c^ 2 studies reporting cases of fusion and rearrangement.

**Table 2:** Numbers of studies dealing in preclinical setting (numbers of total studies = 32).

Variables	Numbers of Studies
Period of publication	
1998–2002	1
2003–2007	2
2008–2012	3
2013–2017	7
2018–2022	13
Up to 2023	6
Type of FGFR alteration	
FGFR1	16
FGFR2	8
FGFR3	1
FGFR4	3
FGFR1-2-3	3
FGFR1-2-3-4	1

**Table 3:** Total of studies dealing in gray literature (total studies = 25).

Variables *	Numbers of Studies
Gray literature (clinicaltrial.gov)	25
Included patients	
Only adult	22
Adult and child	3
Status of Study	
Closed, terminated, withdrawn, or unknown	9
Active but not recruiting; recruiting	15
Approved for marketing	1
FGFR alterations	
FGFR alteration (generic)	21
FGFR2 expression or overexpression	3
Type of drugs	
Selective inhibitors pan-FGFR	11
Non-selective TKIs	1
Phase of study	
Phase I	4
Phase I/II	1
Phase II	17
Phase III	1
Post-market	1
Clinical outcomes (primary)	
Safety ^a^	6
Survival outcomes ^b^	2
Efficacy ^a^	20

* Source clinicaltrial.gov; ^a^ four studies with mixed outcomes with efficacy/safety or part A and part B studies; ^b^ only one study with dedicated survival outcomes.

## Supplementary Materials

The following supporting information can be downloaded at: https://www.mdpi.com/article/10.3390/cancers16162912/s1, Table S1: Search strategy; Table S2: Reason and round of exclusion; Table S3: Characteristics of primary research; Table S4: Primary research; population characteristics; Table S5: FGFR receptor alteration characteristics in preclinical studies; Table S6: Included gray literature characteristics from clinicaltrial.gov.

## References

[1] Ilic I., Ilic M. (2022). International Patterns in Incidence and Mortality Trends of Pancreatic Cancer in the Last Three Decades: A Joinpoint Regression Analysis. World J. Gastroenterol..

[2] Rawla P., Sunkara T., Gaduputi V. (2019). Epidemiology of Pancreatic Cancer: Global Trends, Etiology and Risk Factors. World J. Oncol..

[3] Di Costanzo F., Antonuzzo L., Mazza E., Giommoni E. (2023). Optimizing First-Line Chemotherapy in Metastatic Pancreatic Cancer: Efficacy of FOLFIRINOX versus Nab-Paclitaxel Plus Gemcitabine. Cancers.

[4] Golan T., Hammel P., Reni M., Van Cutsem E., Macarulla T., Hall M.J., Park J.O., Hochhauser D., Arnold D., Oh D.Y. (2019). Maintenance Olaparib for Germline BRCA-Mutated Metastatic Pancreatic Cancer. N. Engl. J. Med..

[5] Philip P.A., Benedetti J., Corless C.L., Wong R., O’Reilly E.M., Flynn P.J., Rowland K.M., Atkins J.N., Mirtsching B.C., Rivkin S.E. (2010). Phase III Study Comparing Gemcitabine Plus Cetuximab versus Gemcitabine in Patients with Advanced Pancreatic Adenocarcinoma: Southwest Oncology Group-Directed Intergroup Trial S0205. J. Clin. Oncol..

[6] Infante J.R., Somer B.G., Park J.O., Li C.P., Scheulen M.E., Kasubhai S.M., Oh D.Y., Liu Y., Redhu S., Steplewski K. (2014). A Randomised, Double-Blind, Placebo-Controlled Trial of Trametinib, an Oral MEK Inhibitor, in Combination with Gemcitabine for Patients with Untreated Metastatic Adenocarcinoma of the Pancreas. Eur. J. Cancer.

[7] Bodoky G., Timcheva C., Spigel D.R., La Stella P.J., Ciuleanu T.E., Pover G., Tebbutt N.C. (2012). A Phase II Open-Label Randomized Study to Assess the Efficacy and Safety of Selumetinib (AZD6244 [ARRY-142886]) versus Capecitabine in Patients with Advanced or Metastatic Pancreatic Cancer Who Have Failed First-Line Gemcitabine Therapy. Investig. New Drugs.

[8] Moore M.J., Goldstein D., Hamm J., Figer A., Hecht J.R., Gallinger S., Au H.J., Murawa P., Walde D., Wolff R.A. (2007). National Cancer Institute of Canada Clinical Trials Group. Erlotinib Plus Gemcitabine Compared with Gemcitabine Alone in Patients with Advanced Pancreatic Cancer: A Phase III Trial of the National Cancer Institute of Canada Clinical Trials Group. J. Clin. Oncol..

[9] Johnson D.E., Williams L.T. (1993). Structural and Functional Diversity in the FGF Receptor Multigene Family. Adv. Cancer Res..

[10] Helsten T., Elkin S., Arthur E., Tomson B.N., Carter J., Kurzrock R. (2016). The FGFR Landscape in Cancer: Analysis of 4853 Tumors by Next-Generation Sequencing. Clin. Cancer Res..

[11] Turner N., Grose R. (2010). Fibroblast Growth Factor Signalling: From Development to Cancer. Nat. Rev. Cancer.

[12] Ohta T., Yamamoto M., Numata M., Iseki S., Tsukioka Y., Miyashita T., Kayahara M., Nagakawa T., Miyazaki I., Nishikawa K. (1995). Expression of Basic Fibroblast Growth Factor and its Receptor in Human Pancreatic Carcinomas. Br. J. Cancer.

[13] Kobrin M.S., Yamanaka Y., Friess H., Lopez M.E., Korc M. (1993). Aberrant Expression of Type I Fibroblast Growth Factor Receptor in Human Pancreatic Adenocarcinomas. Cancer Res..

[14] Aguirre A.J., Nowak J.A., Camarda N.D., Moffitt R.A., Ghazani A.A., Hazar-Rethinam M. (2018). Real-Time Genomic Characterization of Advanced Pancreatic Cancer to Enable Precision Medicine. Cancer Discov..

[15] Cancer Genome Atlas Research Network (2017). Integrated Genomic Characterization of Pancreatic Ductal Adenocarcinoma. Cancer Cell.

[16] D’Agosto S., Pezzini F., Veghini L., Delfino P., Fiorini C., Temgue Tane G.D., Del Curatolo A., Vicentini C., Ferrari G., Pasini D. (2022). Loss of FGFR4 Promotes the Malignant Phenotype of PDAC. Oncogene.

[17] Coetzee A.S., Carter E.P., Rodríguez-Fernández L. (2023). Nuclear FGFR1 Promotes Pancreatic Stellate Cell-Driven Invasion through Up-Regulation of Neuregulin 1. Oncogene.

[18] Liu Z., Neiss N., Zhou S., Henne-Bruns D., Korc M., Bachem M., Kornmann M. (2007). Identification of a Fibroblast Growth Factor Receptor 1 Splice Variant that Inhibits Pancreatic Cancer Cell Growth. Cancer Res..

[19] Kornmann M., Ishiwata T., Matsuda K., Lopez M.E., Fukahi K., Asano G., Beger H.G., Korc M. (2002). IIIc Isoform of Fibroblast Growth Factor Receptor 1 is Overexpressed in Human Pancreatic Cancer and Enhances Tumorigenicity of Hamster Ductal Cells. Gastroenterology.

[20] Li J., Hu K., Huang J., Zhou L., Yan Y., Xu Z. (2021). A Pan-Cancer Analysis of the Expression Landscape and Clinical Relevance of Fibroblast Growth Factor Receptor 2 in Human Cancers. Front. Oncol..

[21] Helal C., Valéry M., Ducreux M., Hollebecque A., Smolenschi C. (2022). FGFR2 Fusion in Metastatic Pancreatic Ductal Adenocarcinoma: Is There Hope?. Eur. J. Cancer.

[22] Matsuda Y., Yoshimura H., Suzuki T., Uchida E., Naito Z., Ishiwata T. (2014). Inhibition of Fibroblast Growth Factor Receptor 2 Attenuates Proliferation and Invasion of Pancreatic Cancer. Cancer Sci..

[23] Cho K., Ishiwata T., Uchida E. (2007). Enhanced Expression of Keratinocyte Growth Factor and its Receptor Correlates with Venous Invasion in Pancreatic Cancer. Am. J. Pathol..

[24] Ishiwata T., Matsuda Y., Yamamoto T., Uchida E., Korc M., Naito Z. (2012). Enhanced Expression of Fibroblast Growth Factor Receptor 2 IIIc Promotes Human Pancreatic Cancer Cell Proliferation. Am. J. Pathol..

[25] Lafitte M., Moranvillier I., Garcia S., Peuchant E., Iovanna J., Rousseau B., Dubus P., Guyonnet-Dupérat V., Belleannée G., Ramos J. (2013). FGFR3 Has Tumor Suppressor Properties in Cells with Epithelial Phenotype. Mol. Cancer.

[26] Motoda N., Matsuda Y., Onda M., Ishiwata T., Uchida E., Naito Z. (2011). Overexpression of Fibroblast Growth Factor Receptor 4 in High-Grade Pancreatic Intraepithelial Neoplasia and Pancreatic Ductal Adenocarcinoma. Int. J. Oncol..

[27] Dowling R.J., Topisirovic I., Alain T., Bidinosti M., Fonseca B.D., Petroulakis E. (2010). mTORC1-Mediated Cell Proliferation, but Not Cell Growth, Controlled by the 4E-BPs. Science.

[28] Du S., Zhang Y., Xu J. (2023). Current Progress in Cancer Treatment by Targeting FGFR Signaling. Cancer Biol. Med..

[29] Chae Y.K., Hong F., Vaklavas C., Cheng H.H., Hammerman P., Mitchell E.P. (2020). Phase II Study of AZD4547 in Patients with Tumors Harboring Aberrations in the FGFR Pathway: Results from the NCI-MATCH Trial (EAY131) Subprotocol W. J. Clin. Oncol..

[30] Voss M.H., Hierro C., Heist R.S., Cleary J.M., Meric-Bernstam F., Tabernero J. (2019). A Phase I, Open-Label, Multicenter, Dose-Escalation Study of the Oral Selective FGFR Inhibitor Debio 1347 in Patients with Advanced Solid Tumors Harboring FGFR Gene Alterations. Clin. Cancer Res..

[31] Koyama T., Shimizu T., Iwasa S., Fujiwara Y., Kondo S., Kitano S. (2020). First-in-Human Phase I Study of E7090, a Novel Selective Fibroblast Growth Factor Receptor Inhibitor, in Patients with Advanced Solid Tumors. Cancer Sci..

[32] Chan S.L., Schuler M., Kang Y.K., Yen C.J., Edeline J., Choo S.P. (2022). A First-in-Human Phase 1/2 Study of FGF401 and Combination of FGF401 with Spartalizumab in Patients with Hepatocellular Carcinoma or Biomarker-Selected Solid Tumors. J. Exp. Clin. Cancer Res..

[33] Tolcher A.W., Papadopoulos K.P., Patnaik A., Wilson K., Thayer S., Zanghi J. (2016). A Phase I, First-in-Human Study of FP-1039 (GSK3052230), a Novel FGF Ligand Trap, in Patients with Advanced Solid Tumors. Ann. Oncol..

[34] Peters M.D.J., Godfrey C., McInerney P., Khalil H., Larsen P., Marnie C., Pollock D., Tricco A.C., Munn Z. (2022). Best Practice Guidance and Reporting Items for the Development of Scoping Review Protocols. JBI Evid. Synth..

[35] Tricco A.C., Lillie E., Zarin W. (2018). PRISMA Extension for Scoping Reviews (PRISMA-ScR): Checklist and Explanation. Ann. Intern. Med..

[36] Page M.J., McKenzie J.E., Bossuyt P.M., Boutron I., Hoffman T.C., Mulrow C.D., Shamseer L., Tetzlaff J.M., Akl E.A., Brennan S.E. (2021). The PRISMA 2020 statement an updated guideline for reporting systematic reviews. BMJ.

[37] Rodon J., Damian S., Furqan M., García-Donas J., Imai H., Italiano A., Spanggaard I., Ueno M., Yokota T., Veronese M.L. (2024). Pemigatinib in previously treated solid tumors with activating FGFR1-FGFR3 alterations: Phase 2 FIGHT-207 basket trial. Nat. Med..

[38] Subbiah V., Iannotti N.O., Gutierrez M., Smith D.C., Féliz L., Lihou C.F., Tian C., Silverman I.M., Ji T., Saleh M. (2022). FIGHT-101, a first-in-human study of potent and selective FGFR 1-3 inhibitor pemigatinib in pan-cancer patients with FGF/FGFR alterations and advanced malignancies. Ann. Oncol..

[39] Kuboki Y., Matsubara N., Bando H., Shitara K., Yoh K., Kojima T., Ohno I., Takahashi H., Harano K., Kondo S. (2017). First-in-human (FIH) study of TAS-120, a highly selective covalent oral fibroblast growth factor receptor (FGFR) inhibitor, in patients (pts) with advanced solid tumors. Ann. Oncol..

[40] Pant S., Schuler M., Iyer G., Witt O., Doi T., Qin S., Tabernero J., Reardon D.A., Massard C., Minchon A. (2023). Erdafitinib in patients with advanced solid tumours with FGFR alterations (RAGNAR): An international, single-arm, phase 2 study. Lancet Oncol..

[41] Saka H., Kitagawa C., Kogure Y., Takahashi Y., Fujikawa K., Sagawa T., Iwasa S., Takahashi N., Fukao T., Tchinou C. (2017). Safety, tolerability and pharmacokinetics of the fibroblast growth factor receptor inhibitor AZD4547 in Japanese patients with advanced solid tumours: A Phase I study. Investig. New Drugs.

[42] Quintela-Fandino M., Bueno M.J., Lombardia L., Gil M., Gonzalez-Martin A., Marquez R., Bratos R., Guerra J., Tan E., Lopez A. (2014). Elective activity over a constitutively active RET-variant of the oral multikinase inhibitor dovitinib: Results of the CNIO-BR002 phase I-trial. Mol. Oncol..

[43] Doi T., Shitara K., Kojima T., Kuboki Y., Matsubara N., Bando H., Yoh K., Naito Y., Hirai H., Kurokawa Y. (2023). Phase I study of the irreversible fibroblast growth factor receptor 1–4 inhibitor futibatinib in Japanese patients with advanced solid tumors. Cancer Sci..

[44] Kim S.B., Meric-Bernstam F., Kalyan A., Babich A., Liu R., Tanigawa T., Sommer A., Osada M., Reetz F., Laurent D. (2019). First-in-Human Phase I Study of Aprutumab Ixadotin, a Fibroblast Growth Factor Receptor 2 Antibody Drug Conjugate (BAY 1187982) in Patients with Advanced Cancer. Target Oncol..

