# Supplementary material for: Exploring the Horizon: Anti-Fibroblast Growth Factor Receptor Therapy in Pancreatic Cancer with Aberrant Fibroblast Growth Factor Receptor Expression—A Scoping Review
Source: Cancers (Basel). 2024 Aug 22;16(16):2912. doi: 10.3390/cancers16162912 (PMC11352631; doi:10.3390/cancers16162912)
Supplement: Supplementary file 1 [file cancers-16-02912-s001.zip › cancers-3085373-supplementary.pdf]

Supplementary Materials: Exploring the Horizon: Anti-FGFR Therapy in Pancreatic Cancer with Aberrant FGFR Expression—A Scoping Review

Elena Orlandi [1,*], Massimo Guasconi [2,3], Stefano Vecchia [4], Serena Trubini [1], Mario Giuffrida [5], Manuela Proietto [1], Elisa Anselmi [1], Patrizio Capelli [5] and Andrea Romboli [5]

[1] Department of Oncology-Hematology, Azienda USL of Piacenza, 29121 Piacenza, Italy; s.trubini@ausl.pc.it (S.T.); m.proietto@ausl.pc.it (M.P.); e.anselmi@ausl.pc.it (E.A.)
[2] Department of Medicine and Surgery, University of Parma, 43121 Parma, Italy; massimo.guasconi@unipr.it
[3] Department of Health Professions Management, Azienda USL of Piacenza, 29121 Piacenza, Italy
[4] Department of Pharmacy, Azienda USL of Piacenza, 29121 Piacenza, Italy; s.vecchia@ausl.pc.it
[5] Department of General Surgery, Azienda USL of Piacenza, 29121 Piacenza, Italy; m.giuffrida@ausl.pc.it (M.G.); p.capelli@ausl.pc.it (P.C.); a.romboli@ausl.pc.it (A.R.)
* Correspondence: elena.orlandi1987@gmail.com

Table S1. Search strategy.

PubMed	("Pancreatic Neoplasms"[Mesh] OR "Pancreas carcinoma"[Title/Abstract:~5] OR "Pancreas carcinomas"[Title/Abstract:~5] OR "Pancreas neoplasm"[Title/Abstract:~5] OR "Pancreas neoplasms"[Title/Abstract:~5] OR "Pancreas Cancer"[Title/Abstract:~5] OR "Pancreas Cancers"[Title/Abstract:~5] OR "Pancreatic Carcinoma"[Title/Abstract:~5] OR "Pancreatic Carcinomas"[Title/Abstract:~5] OR "Pancreatic neoplasm"[Title/Abstract:~5] OR "Pancreatic neoplasms"[Title/Abstract:~5] OR "Pancreatic Cancer"[Title/Abstract:~5] OR "Pancreatic Cancers"[Title/Abstract:~5] OR "neoplasia pancreas"[Title/Abstract:~5] OR "neoplastic pancreas"[Title/Abstract:~5] OR "neoplastic pancreatic"[Title/Abstract:~5] OR "pancreas tumorigenesis"[Title/Abstract:~5] OR "pancreatic tumorigenesis"[Title/Abstract:~5] OR "pancreas tumor"[Title/Abstract:~5] OR "pancreas tumors"[Title/Abstract:~5] OR "pancreas tumour"[Title/Abstract:~5] OR "pancreas tumours"[Title/Abstract:~5] OR "pancreatic tumor"[Title/Abstract:~5] OR "pancreatic tumors"[Title/Abstract:~5] OR "pancreatic tumour"[Title/Abstract:~5] OR "pancreatic tumours"[Title/Abstract:~5]) AND ("Receptors, Fibroblast Growth Factor"[Mesh] OR FGFR[Title/Abstract] OR "Fibroblast Growth Factor Receptor*"[Title/Abstract] OR "FGF Receptor*"[Title/Abstract])
Embase	('pancreas cancer'/exp OR 'pancreas tumor'/exp OR 'pancreas carcinoma'/exp OR (pancreas NEAR/5 carcinoma) OR (pancreas NEAR/5 carcinomas) OR (pancreas NEAR/5 neoplasm) OR (pancreas NEAR/5 neoplasms) OR (pancreas NEAR/5 cancer) OR (pancreas NEAR/5 cancers) OR (pancreatic NEAR/5 carcinoma) OR (pancreatic NEAR/5 carcinomas) OR (pancreatic NEAR/5 neoplasm) OR (pancreatic NEAR/5 neoplasms) OR (pancreatic NEAR/5 cancer) OR (pancreatic NEAR/5 cancers) OR (neoplasia NEAR/5 pancreas) OR (neoplastic NEAR/5 pancreas) OR (neoplastic NEAR/5 pancreatic) OR (pancreas NEAR/5 tumorigenesis) OR (pancreatic NEAR/5 tumorigenesis) OR (pancreas NEAR/5 tumor) OR (pancreas NEAR/5 tumour) OR (pancreatic NEAR/5 tumor) OR (pancreatic NEAR/5 tumour) AND ('fibroblast growth factor receptor'/exp OR 'fgfr':ti,ab,kw OR 'fibroblast growth factor receptor*':ti,ab,kw OR 'fgf receptor*':ti,ab,kw)

Central	([Pancreatic Neoplasms] explode all trees OR Pancreas NEAR/5 carcinoma OR Pancreas NEAR/5 carcinomas OR Pancreas NEAR/5 neoplasm OR Pancreas NEAR/5 neoplasms OR Pancreas NEAR/5 cancer OR Pancreas NEAR/5 cancers OR Pancreatic NEAR/5 carcinoma OR Pancreatic NEAR/5 carcinomas OR Pancreatic NEAR/5 neoplasm OR Pancreatic NEAR/5 neoplasms OR Pancreatic NEAR/5 cancer OR Pancreatic NEAR/5 cancers OR neoplasia NEAR/5 pancreas OR neoplastic NEAR/5 pancreas OR neoplastic NEAR/5 pancreatic OR pancreas NEAR/5 tumorigenesis OR pancreatic NEAR/5 tumorigenesis OR pancreas NEAR/5 tumor OR pancreas NEAR/5 tumors OR pancreas NEAR/5 tumour OR pancreas NEAR/5 tumours OR pancreatic NEAR/5 tumor OR pancreatic NEAR/5 tumors OR pancreatic NEAR/5 tumour OR pancreatic NEAR/5 tumours) AND ([Receptors, Fibroblast Growth Factor] explode all trees OR FGFR OR Fibroblast NEXT Growth NEXT Factor NEXT Receptor* OR FGF NEXT Receptor*)
Cochrane	([Pancreatic Neoplasms] explode all trees OR Pancreas NEAR/5 carcinoma OR Pancreas NEAR/5 carcinomas OR Pancreas NEAR/5 neoplasm OR Pancreas NEAR/5 neoplasms OR Pancreas NEAR/5 cancer OR Pancreas NEAR/5 cancers OR Pancreatic NEAR/5 carcinoma OR Pancreatic NEAR/5 carcinomas OR Pancreatic NEAR/5 neoplasm OR Pancreatic NEAR/5 neoplasms OR Pancreatic NEAR/5 cancer OR Pancreatic NEAR/5 cancers OR neoplasia NEAR/5 pancreas OR neoplastic NEAR/5 pancreas OR neoplastic NEAR/5 pancreatic OR pancreas NEAR/5 tumorigenesis OR pancreatic NEAR/5 tumorigenesis OR pancreas NEAR/5 tumor OR pancreas NEAR/5 tumors OR pancreas NEAR/5 tumour OR pancreas NEAR/5 tumours OR pancreatic NEAR/5 tumor OR pancreatic NEAR/5 tumors OR pancreatic NEAR/5 tumour OR pancreatic NEAR/5 tumours) AND ([Receptors, Fibroblast Growth Factor] explode all trees OR FGFR OR Fibroblast NEXT Growth NEXT Factor NEXT Receptor* OR FGF NEXT Receptor*)
Scopus	TITLE-ABS-KEY ("Pancreas carcinoma" OR "Pancreas carcinomas" OR "Pancreas neoplasm" OR "Pancreas neoplasms" OR "Pancreas cancer" OR "Pancreas cancers" OR "Pancreatic carcinoma" OR "Pancreatic carcinomas" OR "Pancreatic neoplasm" OR "Pancreatic neoplasms" OR "Pancreatic cancer" OR "Pancreatic cancers" OR "neoplasia pancreas" OR "neoplastic pancreas" OR "neoplastic pancreatic" OR "pancreas tumorigenesis" OR "pancreatic tumorigenesis" OR "pancreas tumor" OR "pancreas tumors" OR "pancreas tumour" OR "pancreas tumours" OR "pancreatic tumor" OR "pancreatic tumors" OR "pancreatic tumour" OR "pancreatic tumours") AND TITLE-ABS-KEY (fgfr OR "Fibroblast Growth Factor Receptor*" OR "FGF Receptor*")

Web of Science	((TI=(Pancreas NEAR/5 carcinoma OR Pancreas NEAR/5 carcinomas OR Pancreas NEAR/5 neoplasm OR Pancreas NEAR/5 neoplasms OR Pancreas NEAR/5 cancer OR Pancreas NEAR/5 cancers OR Pancreatic NEAR/5 carcinoma OR Pancreatic NEAR/5 carcinomas OR Pancreatic NEAR/5 neoplasm OR Pancreatic NEAR/5 neoplasms OR Pancreatic NEAR/5 cancer OR Pancreatic NEAR/5 cancers OR neoplasia NEAR/5 pancreas OR neoplastic NEAR/5 pancreas OR neoplastic NEAR/5 pancreatic OR pancreas NEAR/5 tumorigenesis OR pancreatic NEAR/5 tumorigenesis OR pancreas NEAR/5 tumor OR pancreas NEAR/5 tumors OR pancreas NEAR/5 tumour OR pancreas NEAR/5 tumours OR pancreatic NEAR/5 tumor OR pancreatic NEAR/5 tumors OR pancreatic NEAR/5 tumour OR pancreatic NEAR/5 tumours)) OR AB=(Pancreas NEAR/5 carcinoma OR Pancreas NEAR/5 carcinomas OR Pancreas NEAR/5 neoplasm OR Pancreas NEAR/5 neoplasms OR Pancreas NEAR/5 cancer OR Pancreas NEAR/5 cancers OR Pancreatic NEAR/5 carcinoma OR Pancreatic NEAR/5 carcinomas OR Pancreatic NEAR/5 neoplasm OR Pancreatic NEAR/5 neoplasms OR Pancreatic NEAR/5 cancer OR Pancreatic NEAR/5 cancers OR neoplasia NEAR/5 pancreas OR neoplastic NEAR/5 pancreas OR neoplastic NEAR/5 pancreatic OR pancreas NEAR/5 tumorigenesis OR pancreatic NEAR/5 tumorigenesis OR pancreas NEAR/5 tumor OR pancreas NEAR/5 tumors OR pancreas NEAR/5 tumour OR pancreas NEAR/5 tumours OR pancreatic NEAR/5 tumor OR pancreatic NEAR/5 tumors OR pancreatic NEAR/5 tumour OR pancreatic NEAR/5 tumours)) OR KP=(Pancreas NEAR/5 carcinoma OR Pancreas NEAR/5 carcinomas OR Pancreas NEAR/5 neoplasm OR Pancreas NEAR/5 neoplasms OR Pancreas NEAR/5 cancer OR Pancreas NEAR/5 cancers OR Pancreatic NEAR/5 carcinoma OR Pancreatic NEAR/5 carcinomas OR Pancreatic NEAR/5 neoplasm OR Pancreatic NEAR/5 neoplasms OR Pancreatic NEAR/5 cancer OR Pancreatic NEAR/5 cancers OR neoplasia NEAR/5 pancreas OR neoplastic NEAR/5 pancreas OR neoplastic NEAR/5 pancreatic OR pancreas NEAR/5 tumorigenesis OR pancreatic NEAR/5 tumorigenesis OR pancreas NEAR/5 tumor OR pancreas NEAR/5 tumors OR pancreas NEAR/5 tumour OR pancreas NEAR/5 tumours OR pancreatic NEAR/5 tumor OR pancreatic NEAR/5 tumors OR pancreatic NEAR/5 tumour OR pancreatic NEAR/5 tumours) AND ((TI=(FGFR OR Fibroblast NEXT Growth NEXT Factor NEXT Receptor* OR FGF NEXT Receptor*)) OR AB=(FGFR OR Fibroblast NEXT Growth NEXT Factor NEXT Receptor* OR FGF NEXT Receptor*)) OR KP=(FGFR OR Fibroblast NEXT Growth NEXT Factor NEXT Receptor* OR FGF NEXT Receptor*)
Open dissertation	("Pancreas carcinoma" OR "Pancreas carcinomas" OR "Pancreas neoplasm" OR "Pancreas neoplasms" OR "Pancreas cancer" OR "Pancreas cancers" OR "Pancreatic carcinoma" OR "Pancreatic carcinomas" OR "Pancreatic neoplasm" OR "Pancreatic neoplasms" OR "Pancreatic cancer" OR "Pancreatic cancers" OR "neoplasia pancreas" OR "neoplastic pancreas" OR "neoplastic pancreatic" OR "pancreas tumorigenesis" OR "pancreatic tumorigenesis" OR "pancreas tumor" OR "pancreas tumors" OR "pancreas tumour" OR "pancreas tumours" OR "pancreatic tumor" OR "pancreatic tumors" OR "pancreatic tumour" OR "pancreatic tumours") AND (fgfr OR "Fibroblast Growth Factor Receptor*" OR "FGF Receptor*")

Table S2. Reason and round of exclusion.

Authors/NCT number	Title	Year	Journal	Url	Reason of exclusion	Doi	Round of exclusion
Xu, J and Shen, L and Bai, C and Li, J and Zhou, Z and Yu, X and Li, Z and Li, E and Yuan, X and Chi, Y and et al.	Surufatinib (S) for patients (Pts) with advanced pancreatic neuroendocrine tumours (SANET-p): a randomized, double-blind, placebo (P)-controlled phase III trial (NCT02589821)	2020		https://www.cochranelibrary.com/central/doi/10.1002/central/CN-02176940/full	wrong population		Round I
Xu, J and Shen, L and Zhou, Z and Li, J and Bai, C and Chi, Y and Li, Z and Xu, N and Jia, R and Li, E and et al.	Efficacy and safety of surufatinib in patients with well-differentiated advanced extrapancreatic neuroendocrine tumors (NETs): results from the randomized phase III study (SANET-ep)	2019		https://www.cochranelibrary.com/central/doi/10.1002/central/CN-02096038/full	wrong population		Round I
Salmon, JS and Hwang, JJ and Robinson, MM and Symanowski, JT and Dillon, LM and Roy, LD and Beldner, MA and Preston, K and Buige, S and Nazemzadeh, R and et al.	Phase II study of regorafenib (Reg) in patients with previously treated advanced pancreatic cancer (APC)	2017		https://www.cochranelibrary.com/central/doi/10.1002/central/CN-01398330/full	wrong drug		Round I
Coleman, SJ and Chioni, AM and Ghallab, M and Anderson, RK and Lemoine, NR and Kocher, HM and Grose, RP	Nuclear translocation of FGFR1 and FGF2 in pancreatic stellate cells facilitates pancreatic cancer cell invasion	2014	EMBO MOLECULAR MEDICINE		wrong publication type	10.1002/emmm.201302698	Round I
Verma, HK and Kampalli, PK and Lakkakula, S and Chalikonda, G and Bhaskar, LVKS and Pattnaik, S	A Retrospective Look at Anti-EGFR Agents in Pancreatic Cancer Therapy	2019	CURRENT DRUG METABOLISM		wrong publication type	10.2174/1389200220666191122104955	Round I
Crawford, K and Bontrager, E and Schwarz, MA and Chaturvedi, A and Lee, DD and Sazzad, HM and von Holzen, U and Zhang, CH and Schwarz, RE and Awasthi, N	Targeted FGFR/VEGFR/PDGFR inhibition with dovitinib enhances the effects of nab-paclitaxel in preclinical gastric cancer models	2021	CANCER BIOLOGY & THERAPY		wrong drug	10.1080/15384047.2021.2011642	Round I
Wei, MM and Peng, X and Xing, L and Dai, Y and Huang, RM and Geng, MY and Zhang, A and Ai, J and Song, ZL	Design, synthesis and biological evaluation of a series of novel 2-benzamide-4-(6-oxy-N-methyl-l-naphthamide)-pyridine derivatives as potent fibroblast growth factor receptor (FGFR) inhibitors	2018	EUROPEAN JOURNAL OF MEDICINAL CHEMISTRY		wrong population	10.1016/j.ejmech.2018.05.005	Round I
Baranov, E and Alkhateeb, K and Isidro, R and Doyle, L and Abrams, T and Zhao, L	Structural Variant Analysis of FGFR-Rearranged Pancreatic and Extrahepatic Biliary Carcinomas	2023	LABORATORY INVESTIGATION		wrong publication type		Round I
Shin, EY and Lee, BH and Yang, JH and Shin, KS and Lee, GK and Yun, HY and Song, YJ and Park, SC and Kim, EG	Up-regulation and co-expression of fibroblast growth factor receptors in human gastric cancer	2000	JOURNAL OF CANCER RESEARCH AND CLINICAL ONCOLOGY		wrong population	10.1007/s004320000128	Round I
Ye, YH and Huang, ZY and Zhang, MQ and Li, JY and Zhang, YQ and Lou, CH	Synergistic therapeutic potential of alpelisib in cancers (excluding breast cancer): Preclinical and clinical evidences	2023	BIOMEDICINE & PHARMACOTHERAPY		wrong publication type	10.1016/j.biopha.2022.114183	Round I
Vickers, SM and Huang, ZQ and Macmillan-Crow, L and Thompson, JA	Ligand activation of alternatively spliced FGFR-1 modulates pancreatic cancer cell malignancy	2001	GASTROENTEROLOGY		wrong publication type		Round I

Arbeit, JM and Olson, DC and Hanahan, D	Upregulation of fibroblast growth factors and their receptors during multi-stage epidermal carcinogenesis in K14-HPV16 transgenic mice	1996	ONCOGENE		wrong population		Round I
Azad, T and Nouri, K and van Rensburg, HJJ and Maritan, SM and Wu, LQ and Hao, YW and Montminy, T and Yu, JH and Khanal, P and Mulligan, LM and Yang, XL	A gain-of-functional screen identifies the Hippo pathway as a central mediator of receptor tyrosine kinases during tumorigenesis	2020	ONCOGENE		wrong population	10.1038/s41388-019-0988-y	Round I
Ferguson, HR and Smith, MP and Francavilla, C	Fibroblast Growth Factor Receptors (FGFRs) and Noncanonical Partners in Cancer Signaling	2021	CELLS		wrong population	10.3390/cells10051201	Round I
Hallinan, N and Finn, S and Cuffe, S and Rafee, S and O'Byrne, K and Gately, K	Targeting the fibroblast growth factor receptor family in cancer	2016	CANCER TREATMENT REVIEWS		wrong publication type	10.1016/j.ctrv.2016.03.015	Round I
Feng, SC and Zhou, L and Nice, EC and Huang, CH	Fibroblast growth factor receptors: multifactorial-contributors to tumor initiation and progression	2015	HISTOLOGY AND HISTOPATHOLOGY		wrong publication type		Round I
Korc, M and Friesel, RE	The Role of Fibroblast Growth Factors in Tumor Growth	2009	CURRENT CANCER DRUG TARGETS		wrong publication type	10.2174/156800909789057006	Round I
Onda, M and Naito, Z and Wang, RJ and Fujii, T and Kawahara, K and Ishiwata, T and Sugisaki, Y	Expression of keratinocyte growth factor receptor (KGFR/FGFR2 IIIb) in vascular smooth muscle cells	2003	PATHOLOGY INTERNATIONAL		wrong population	10.1046/j.1440-1827.2003.01445.x	Round I
Stangis, MM and Colah, AN and McLean, D and Halberg, RB and Collier, LS and Ricke, WA	Potential roles of FGF5 as a candidate therapeutic target in prostate cancer	2023	AMERICAN JOURNAL OF CLINICAL AND EXPERIMENTAL UROLOGY		wrong population		Round I
Chan, JK and Pham, HY and You, XJ and Cloven, NG and Burger, RA and Rose, GS and Van Nostrand, K and Korc, M and DiSaia, PJ and Fan, H	Suppression of ovarian cancer cell tumorigenicity and evasion of cisplatin resistance using a truncated epidermal growth factor receptor in a rat model	2005	CANCER RESEARCH		wrong population	10.1158/0008-5472.CAN-03-3013	Round I
Turkington, RC and Longley, DB and Allen, WL and Stevenson, L and McLaughlin, K and Dunne, PD and Blayney, JK and Salto-Tellez, M and Van Schaeybroeck, S and Johnston, PG	Fibroblast growth factor receptor 4 (FGFR4): a targetable regulator of drug resistance in colorectal cancer	2014	CELL DEATH & DISEASE		wrong population	10.1038/cddis.2014.10	Round I
FernÃ¡ndez-GuizÃ¡n, A and LÃ³pez-Soto, A and Acebes-Huerta, A and Huergo-Zapico, L and Villa-Alvarez, M and NÃºÃ±ez, LE and MorÃ-s, F and Gonzalez, S	Pleiotropic Anti-Angiogenic and Anti-Oncogenic Activities of the Novel Mithralog Demycarosyl-3D-Î²-D-Digitoxosyl-Mithramycin SK (EC-8042)	2015	PLOS ONE		wrong publication type	10.1371/journal.pone.0140786	Round I
Nel, I and Gauler, TC and Bublitz, K and Lazaridis, L and Goergens, A and Giebel, B and Schuler, M and Hoffmann, AC	Circulating Tumor Cell Composition in Renal Cell Carcinoma	2016	PLOS ONE		wrong population	10.1371/journal.pone.0153018	Round I
Estes, NR and Thottassery, JV and Kern, FG	siRNA mediated knockdown of fibroblast growth factor receptors 1 or 3 inhibits FGF-induced anchorage-independent clonogenicity but does not affect MAPK activation	2006	ONCOLOGY REPORTS		wrong outcomes		Round I

Vienot, A and Neuzillet, C	Cholangiocarcinoma: the quest for a second-line systemic treatment	2019	TRANSLATIONAL CANCER RESEARCH		wrong publication type	10.21037/tcr.2018.10.05	Round I
Thomson, S and Petti, F and Sujka-Kwok, I and Mercado, P and Bean, J and Monaghan, M and Seymour, SL and Argast, GM and Epstein, DM and Haley, JD	A systems view of epithelial-mesenchymal transition signaling states	2011	CLINICAL & EXPERIMENTAL METASTASIS		wrong publication type	10.1007/s10585-010-9367-3	Round I
Glorieux, C and Xia, XJ and Huang, P	The Role of Oncogenes and Redox Signaling in the Regulation of PD-L1 in Cancer	2021	CANCERS		wrong publication type	10.3390/cancers13174426	Round I
Rastad, H. and Samimisedeh, P. and Alan, M.S. and Afshar, E.J. and Ghalami, J. and Hashemnejad, M. and Alan, M.S.	The role of lncRNA CERS6-AS1 in cancer and its molecular mechanisms: A systematic review and meta-analysis	2023	Pathology Research and Practice	https://www.scopus.com/inward/record.uri?eid=2-s2.0-85144981589&doi=10.1016%2fj.prp.2022.154245&partnerID=40&md5=ed2de13ced591ad16b3b06fb6b092dd3	wrong population		Round I
Levantini, E. and Maroni, G. and Del Re, M. and Tenen, D.G.	EGFR signaling pathway as therapeutic target in human cancers	2022	Seminars in Cancer Biology	https://www.scopus.com/inward/record.uri?eid=2-s2.0-85128199386&doi=10.1016%2fj.semcancer.2022.04.002&partnerID=40&md5=fe40a196153aa3e54fe17f71e65d8b9f	wrong population		Round I
Valery, M. and Vasseur, D. and Fachinetti, F. and BoilÃ¨ve, A. and Smolenschi, C. and Tarabay, A. and Antoun, L. and Perret, A. and Fuerea, A. and Pudlarz, T. and Boige, V. and Hollebecque, A. and Ducreux, M.	Targetable Molecular Alterations in the Treatment of Biliary Tract Cancers: An Overview of the Available Treatments	2023	Cancers	https://www.scopus.com/inward/record.uri?eid=2-s2.0-85172794627&doi=10.3390%2fcancers15184446&partnerID=40&md5=ecd7de24f7ee7682c53dd64fe566ab91	wrong population		Round I
Wu, K. and Niu, C. and Liu, H. and Fu, L.	Research progress on PRMTs involved in epigenetic modification and tumour signalling pathway regulation (Review)	2023	International Journal of Oncology	https://www.scopus.com/inward/record.uri?eid=2-s2.0-85151881226&doi=10.3892%2fijo.2023.5510&partnerID=40&md5=05958c01ba092e12da950ef628dc4921	wrong population		Round I
Guo, Y. and Tian, C. and Cheng, Z. and Chen, R. and Li, Y. and Su, F. and Shi, Y. and Tan, H.	Molecular and Functional Heterogeneity of Primary Pancreatic Neuroendocrine Tumors and Metastases	2023	Neuroendocrinology	https://www.scopus.com/inward/record.uri?eid=2-s2.0-85169174857&doi=10.1159%2f000530968&partnerID=40&md5=c8b74a4a5ae42a7316fcec8d93c27e0d	wrong population		Round I
Lebedeva, A. and Timokhin, G. and Ignatova, E. and Kavun, A. and Veselovsky, E. and Sharova, M. and Mileyko, V. and Yakushina, V. and Kuznetsova, O. and Stepanova, M. and Shilo, P. and Moiseenko, F. and Volkov, N. and Plaksa, I. and Isaev, A. and Gayryan, M. and Artemyeva, E. and Zhabina, A. and Kramchaninov, M. and Shamrikova, V. and Pokataev, I. and Rumyantsev, A. and Ledin, E. and Tryakin, A. and Fedyanin, M. and Ivanov, M.	Utility of public knowledge bases for the interpretation of comprehensive tumor molecular profiling results	2023	Clinical and Experimental Medicine	https://www.scopus.com/inward/record.uri?eid=2-s2.0-85147665117&doi=10.1007%2fs10238-023-01011-6&partnerID=40&md5=8e2daf56baecd256a977bc55242f08c2	wrong population		Round I

Hashemi, M. and Hajimazdarany, S. and Mohan, C.D. and Mohammadi, M. and Rezaei, S. and Olyaee, Y. and Goldoost, Y. and Ghorbani, A. and Mirmazloomi, S.R. and Gholinia, N. and Kakavand, A. and Salimimoghadam, S. and Ertas, Y.N. and Rangappa, K.S. and Taheriazam, A. and Entezari, M.	Long non-coding RNA/epithelial-mesenchymal transition axis in human cancers: Tumorigenesis, chemoresistance, and radioresistance	2022	Pharmacological Research	https://www.scopus.com/inward/record.uri?eid=2-s2.0-85141493898&doi=10.1016%2fj.phrs.2022.106535&partnerID=40&md5=8aac9f6c1d937e760513c755cf75dc54	wrong population		Round I
Matsuoka, T. and Yashiro, M.	Current status and perspectives of genetic testing in gastrointestinal cancer (Review)	2024	Oncology Letters	https://www.scopus.com/inward/record.uri?eid=2-s2.0-85179467670&doi=10.3892%2fol.2023.14155&partnerID=40&md5=58ece905ac9ad55411ebf7014640957f	wrong population		Round I
Ramjeesingh, R. and Chaudhury, P. and Tam, V.C. and Roberge, D. and Lim, H.J. and Knox, J.J. and Asselah, J. and Doucette, S. and Chhiber, N. and Goodwin, R.	A Practical Guide for the Systemic Treatment of Biliary Tract Cancer in Canada	2023	Current Oncology	https://www.scopus.com/inward/record.uri?eid=2-s2.0-85168724751&doi=10.3390%2fcurroncol30080517&partnerID=40&md5=42dc25414b0cdca6d9f11785f8b914de	wrong population		Round I
Das, R. and Agrawal, S. and Kumar, P. and Singh, A.K. and Shukla, P.K. and Bhattacharya, I. and Tiwari, K.N. and Mishra, S.K. and Tripathi, A.K.	Network pharmacology of apigeniflavan: a novel bioactive compound of Trema orientalis Linn. in the treatment of pancreatic cancer through bioinformatics approaches	2023	3 Biotech	https://www.scopus.com/inward/record.uri?eid=2-s2.0-85158049439&doi=10.1007%2fs13205-023-03570-7&partnerID=40&md5=7ca8484f80f9c239df6c2f7278cc0bf3	wrong population		Round I
Toledano, S. and Neufeld, G.	Plexins as Regulators of Cancer Cell Proliferation, Migration, and Invasivity	2023	Cancers	https://www.scopus.com/inward/record.uri?eid=2-s2.0-85168802710&doi=10.3390%2fcancers15164046&partnerID=40&md5=b75de7be443e689e388ce99460670128	wrong population		Round I
Wang, V. and Gauthier, M. and Decot, V. and Reppel, L. and Bensoussan, D.	Systematic Review on CAR-T Cell Clinical Trials Up to 2022: Academic Center Input	2023	Cancers	https://www.scopus.com/inward/record.uri?eid=2-s2.0-85149133583&doi=10.3390%2fcancers15041003&partnerID=40&md5=dffb093ce8623d681dffcefbb0e064e9	wrong population		Round I
Greene, L.A. and Zhou, Q. and Siegelin, M.D. and Angelastro, J.M.	Targeting Transcription Factors ATF5, CEBPB and CEBPD with Cell-Penetrating Peptides to Treat Brain and Other Cancers	2023	Cells	https://www.scopus.com/inward/record.uri?eid=2-s2.0-85148859606&doi=10.3390%2fcells12040581&partnerID=40&md5=f85408f3a1c9b22a23f2dac02b108d01	wrong population		Round I
Su, C.-Y. and Yan, Y.-Y. and Zhang, J.-Y. and Zhang, Y.-K. and Chen, Z.-S.	Editorial: Targeted cancer therapies, from small molecules to antibodies, volume II	2023	Frontiers in Pharmacology	https://www.scopus.com/inward/record.uri?eid=2-s2.0-85165078438&doi=10.3389%2ffphar.2023.1147488&partnerID=40&md5=a25d1787f71a06f825e598ecd7c44cba	wrong population		Round I
Kaur, G. and Khanna, B. and Yusuf, M. and Sharma, A. and Khajuria, A. and Alajangi, H.K. and Jaiswal, P.K. and Sachdeva, M. and Barnwal, R.P. and Singh, G.	A Path of Novelty from Nanoparticles to Nanobots: Theragnostic Approach for Targeting Cancer Therapy	2024	Critical Reviews in Therapeutic Drug Carrier Systems	https://www.scopus.com/inward/record.uri?eid=2-s2.0-85184345289&doi=10.1615%2fCritRevTherDrugCarrierSyst.2023046674&partnerID=40&md5=a9208637c928cde0adcfdfb6bf93c2ea	wrong population		Round I
Qian, J. and Li, J.N. and Rose, E.K. and Vandergriff, T. and Khosama, L. and Beg, M.S. and Mauskar, M.M. and Wang, R.C.	Fibroblast growth factor receptor inhibitor therapy induced calcinosis cutis treated with sodium thiosulfate	2023	JAAD Case Reports	https://www.scopus.com/inward/record.uri?eid=2-s2.0-85144356754&doi=10.1016%2fj.jdcr.2022.10.040&partnerID=40&md5=2808aaf0316ed2c18e74d40d0f95ccbd	wrong population		Round I

Gkountakos, A. and Mafficini, A. and Lou, E. and Malleo, G. and Salvia, R. and Calicchia, M. and Silvestris, N. and Racila, E. and Amin, K. and Veronese, N. and Brunetti, O. and Antonini, P. and Ingravallo, G. and Mattiolo, P. and Saponaro, C. and Nappo, F. and Simbolo, M. and Bariani, E. and Lonardi, S. and Fassan, M. and Milella, M. and Lawlor, R.T. and Scarpa, A. and Luchini, C.	Genomic characterization of undifferentiated sarcomatoid carcinoma of the pancreas	2022	Human Pathology	https://www.scopus.com/inward/record.uri?eid=2-s2.0-85136263349&doi=10.1016%2fj.humpath.2022.07.011&partnerID=40&md5=8715d7705de382e6ad862405d1846961	wrong population		Round I
Tanaka, N. and Sakamoto, T.	MT1-MMP as a Key Regulator of Metastasis	2023	Cells	https://www.scopus.com/inward/record.uri?eid=2-s2.0-85170187435&doi=10.3390%2fcells12172187&partnerID=40&md5=cc089383a4f4117cebadab998dc82459	wrong population		Round I
Gonzalez-CÃ¡rdenas, M. and TreviÃ±o, V.	The Impact of Mutational Hotspots on Cancer Survival	2024	Cancers	https://www.scopus.com/inward/record.uri?eid=2-s2.0-85187437962&doi=10.3390%2fcancers16051072&partnerID=40&md5=037e512ac54d5bb2481138bf2b3a43f0	wrong population		Round I
Singh, S. and Sadhukhan, S. and Sonawane, A.	20 years since the approval of first EGFR-TKI, gefitinib: Insight and foresight	2023	Biochimica et Biophysica Acta - Reviews on Cancer	https://www.scopus.com/inward/record.uri?eid=2-s2.0-85172375350&doi=10.1016%2fj.bbcan.2023.188967&partnerID=40&md5=6d5b06b71e20f4c06b8f5b6e836244d5	wrong population		Round I
Guijarro, L.G. and Justo Bermejo, F.J. and Boaru, D.L. and De Castro-Martinez, P. and De Leon-Oliva, D. and Fraile-MartÃnez, O. and Garcia-Montero, C. and Alvarez-Mon, M. and Toledo-Lobo, M.D.V. and Ortega, M.A.	Is Insulin Receptor Substrate4 (IRS4) a Platform Involved in the Activation of Several Oncogenes?	2023	Cancers	https://www.scopus.com/inward/record.uri?eid=2-s2.0-85172800683&doi=10.3390%2fcancers15184651&partnerID=40&md5=d32522011d92160648501471a8fb8246	wrong population		Round I
Pietan, L. and Vaughn, H. and Howe, J.R. and Bellizzi, A.M. and Smith, B.J. and Darbro, B. and Braun, T. and Casavant, T.	Prioritization of Fluorescence In Situ Hybridization (FISH) Probes for Differentiating Primary Sites of Neuroendocrine Tumors with Machine Learning	2023	International Journal of Molecular Sciences	https://www.scopus.com/inward/record.uri?eid=2-s2.0-85180715910&doi=10.3390%2fijms242417401&partnerID=40&md5=000aff51e79eb2c181a63953fed13a59	wrong population		Round I
Voutsadakis, I.A. and Digklia, A.	Pancreatic adenocarcinomas without KRAS, TP53, CDKN2A and SMAD4 mutations and CDKN2A/CDKN2B copy number alterations: a review of the genomic landscape to unveil therapeutic avenues	2023	Chinese Clinical Oncology	https://www.scopus.com/inward/record.uri?eid=2-s2.0-85150314900&doi=10.21037%2fcco-22-108&partnerID=40&md5=a46176c3f10b2d0eaf05c9bc38aa0099	wrong population		Round I
Afaq, F. and Agarwal, S. and Bajpai, P. and Diffalha, S.A. and Kim, H.-G. and Peter, S. and Khushman, M. and Chauhan, S.C. and Mukherjee, P. and Varambally, S. and Manne, U.	Targeting of oncogenic AAA-ATPase TRIP13 reduces progression of pancreatic ductal adenocarcinoma	2024	Neoplasia (United States)	https://www.scopus.com/inward/record.uri?eid=2-s2.0-85178159530&doi=10.1016%2fj.neo.2023.100951&partnerID=40&md5=a90f29b9e4c8decff3dcc95a75b7fb0e	wrong population		Round I
Zhang, B. and Guo, X. and Huang, L. and Zhang, Y. and Li, Z. and Su, D. and Lin, L. and Zhou, P. and Ye, H. and Lu, Y. and Zhou, Q.	Tumour-associated macrophages and Schwann cells promote perineural invasion via paracrine loop in pancreatic ductal adenocarcinoma	2024	British Journal of Cancer	https://www.scopus.com/inward/record.uri?eid=2-s2.0-85180251909&doi=10.1038%2fs41416-023-02539-w&partnerID=40&md5=a51929d3460feac856aac8ba50e41696	wrong population		Round I

Servetto, A. and Esposito, D. and Ferrara, R. and Signorelli, D. and Belli, S. and Napolitano, F. and Santaniello, A. and Ciciola, P. and Formisano, L. and Bianco, R.	RET rearrangements in non-small cell lung cancer: Evolving treatment landscape and future challenges	2022	Biochimica et Biophysica Acta - Reviews on Cancer	https://www.scopus.com/inward/record.uri?eid=2-s2.0-85139418533&doi=10.1016%2fj.bbcan.2022.188810&partnerID=40&md5=4e54bc3c4eb6416183aff78c036e5125	wrong population		Round I
Fink, J.L. and Jaradi, B. and Stone, N. and Anderson, L. and Leo, P.J. and Marshall, M. and Ellis, J. and Waring, P.M. and O'Byrne, K.	Minimizing Sample Failure Rates for Challenging Clinical Tumor Samples	2023	Journal of Molecular Diagnostics	https://www.scopus.com/inward/record.uri?eid=2-s2.0-85156177594&doi=10.1016%2fj.jmoldx.2023.01.008&partnerID=40&md5=0b5fb660755877a1b60746d92a54d243	wrong population		Round I
Zhang, Y. and Chen, F. and Chandrashekar, D.S. and Varambally, S. and Creighton, C.J.	Proteogenomic characterization of 2002 human cancers reveals pan-cancer molecular subtypes and associated pathways	2022	Nature Communications	https://www.scopus.com/inward/record.uri?eid=2-s2.0-85130073110&doi=10.1038%2fs41467-022-30342-3&partnerID=40&md5=88e375aa1be328f9c5d6696a1e176cb8	wrong population		Round I
Zhang, C. and Niu, W. and Xu, Y. and Lu, Y. and Huang, L. and Li, S. and Jiang, X. and Wu, J.	Multivisceral resection of nonfunctional pancreatic neuroendocrine neoplasm with nearby organ invasion: a case report	2023	Frontiers in Endocrinology	https://www.scopus.com/inward/record.uri?eid=2-s2.0-85173787165&doi=10.3389%2ffendo.2023.1236685&partnerID=40&md5=d829146ce42963808e4880622c6f1345	wrong population		Round I
Ricci, V. and Fabozzi, T. and Bareschino, M.A. and Barletta, E. and Germano, D. and Paciolla, I. and Tinessa, V. and Grimaldi, A.M.	Pancreatic Cancer: Beyond Brca Mutations	2022	Journal of Personalized Medicine	https://www.scopus.com/inward/record.uri?eid=2-s2.0-85144681503&doi=10.3390%2fjpm12122076&partnerID=40&md5=393b439d93e78969c756525f29966e66	wrong population		Round I
Roskoski, R.	Properties of FDA-approved small molecule protein kinase inhibitors: A 2023 update	2023	Pharmacological Research	https://www.scopus.com/inward/record.uri?eid=2-s2.0-85145599715&doi=10.1016%2fj.phrs.2022.106552&partnerID=40&md5=baae53e737f71460e2fe8ef75950e055	wrong population		Round I
Liu, M. and Li, N. and Tang, H. and Chen, L. and Liu, X. and Wang, Y. and Lin, Y. and Luo, Y. and Wei, S. and Wen, W. and Chen, M. and Wang, J. and Zhang, N. and Chen, J.	The Mutational, Prognostic, and Therapeutic Landscape of Neuroendocrine Neoplasms	2023	Oncologist	https://www.scopus.com/inward/record.uri?eid=2-s2.0-85170294623&doi=10.1093%2foncolo%2foyad093&partnerID=40&md5=e6cf2c61c26a64cdf009228e2dde91ed	wrong population		Round I
Dong, Y. and Ren, C. and Zhu, M. and Zhang, D. and Wang, T. and Zhang, J. and Wang, J. and Mao, W. and Long, F.	Targeting FGFRs for tumor therapy: current status and novel strategies	2022	Future Medicinal Chemistry	https://www.scopus.com/inward/record.uri?eid=2-s2.0-85145969893&doi=10.4155%2ffmc-2022-0194&partnerID=40&md5=1e1cac35aff2dfc225125f57f04f53f3	wrong population		Round I
Barua, S. and Hsiao, S. and Clancy, E. and Freeman, C. and Mansukhani, M. and Fernandes, H.	Quality metrics for enhanced performance of an NGS panel using single-vial amplification technology	2024	Journal of Clinical Pathology	https://www.scopus.com/inward/record.uri?eid=2-s2.0-85143435538&doi=10.1136%2fjcp-2022-208536&partnerID=40&md5=a70b21d2e429038de54312e3a39b49ae	wrong population		Round I
Feng, C. and Zhang, L. and Chang, X. and Qin, D. and Zhang, T.	Regulation of post-translational modification of PD-L1 and advances in tumor immunotherapy	2023	Frontiers in Immunology	https://www.scopus.com/inward/record.uri?eid=2-s2.0-85167351624&doi=10.3389%2ffimmu.2023.1230135&partnerID=40&md5=4cb9a31e1a61a37b7cde91d725b86584	wrong population		Round I
Herreros-Villanueva, M. and Bujanda, L. and Ruiz-Rebollo, L. and Torremocha, R. and Ramos, R. and MartÃ-n, R. and Artigas, M.C.	Circulating tumor DNA tracking in patients with pancreatic cancer using next-generation sequencing	2022	Gastroenterologia y Hepatologia	https://www.scopus.com/inward/record.uri?eid=2-s2.0-85124909094&doi=10.1016%2fj.gastrohep.2021.12.011&partnerID=40&md5=15a39ffdc5853483401d9aead11ec4a3	wrong population		Round I

Geerinckx, B. and Teuwen, L.-A. and Foo, T. and Vandamme, T. and Smith, A. and Peeters, M. and Price, T.	Novel therapeutic strategies in pancreatic cancer: moving beyond cytotoxic chemotherapy	2023	Expert Review of Anticancer Therapy	https://www.scopus.com/inward/record.uri?eid=2-s2.0-85174390397&doi=10.1080%2f14737140.2023.2270161&partnerID=40&md5=b4bea012704e046f378dd1d701a32313	wrong population		Round I
Wagner, W. and Ochman, B. and Wagner, W.	Semaphorin 6 Familyâ€"An Important Yet Overlooked Group of Signaling Proteins Involved in Cancerogenesis	2023	Cancers	https://www.scopus.com/inward/record.uri?eid=2-s2.0-85179327898&doi=10.3390%2fcancers15235536&partnerID=40&md5=7da11ce53fc109ee58721766f9c29c52	wrong population		Round I
Sanchez, A. and Lhuillier, J. and Grosjean, G. and Ayadi, L. and Maenner, S.	The Long Non-Coding RNA ANRIL in Cancers	2023	Cancers	https://www.scopus.com/inward/record.uri?eid=2-s2.0-85168809869&doi=10.3390%2fcancers15164160&partnerID=40&md5=5b329a33a91c483e6f7c644756e7f321	wrong population		Round I
Rosen, E. and Drilon, A. and Chakravarty, D.	Precision Oncology: 2022 in Review	2022	Cancer Discovery	https://www.scopus.com/inward/record.uri?eid=2-s2.0-85143183803&doi=10.1158%2f2159-8290.CD-22-1154&partnerID=40&md5=e5fd4fe022568ec6caf44c2717a08618	wrong population		Round I
Springfeld, C. and Ferrone, C.R. and Katz, M.H.G. and Philip, P.A. and Hong, T.S. and Hackert, T. and BÃ¼chler, M.W. and Neoptolemos, J.	Neoadjuvant therapy for pancreatic cancer	2023	Nature Reviews Clinical Oncology	https://www.scopus.com/inward/record.uri?eid=2-s2.0-85150220468&doi=10.1038%2fs41571-023-00746-1&partnerID=40&md5=fb50e8f3053427a4f9ca1825ea804ace	wrong population		Round I
Rimal, R. and Desai, P. and Daware, R. and Hosseinnejad, A. and Prakash, J. and Lammers, T. and Singh, S.	Cancer-associated fibroblasts: Origin, function, imaging, and therapeutic targeting	2022	Advanced Drug Delivery Reviews	https://www.scopus.com/inward/record.uri?eid=2-s2.0-85136701209&doi=10.1016%2fj.addr.2022.114504&partnerID=40&md5=fec4625e329993f094f230597920172b	wrong population		Round I
Ben-Ammar, I. and Rousseau, A. and Nicolle, R. and Tarabay, A. and Boige, V. and Valery, M. and Pudlarz, T. and Malka, D. and Gelli, M. and Fernandez-De-Sevilla, E. and Fuerea, A. and Tanguy, M.-L. and Rouleau, E. and Barbe, R. and Mathieu, J.R.R. and Jaulin, F. and Smolenschi, C. and Hollebecque, A. and Ducreux, M. and Boileve, A.	Precision medicine for KRAS wild-type pancreatic adenocarcinomas	2024	European Journal of Cancer	https://www.scopus.com/inward/record.uri?eid=2-s2.0-85180595475&doi=10.1016%2fj.ejca.2023.113497&partnerID=40&md5=6b87fedb92c124fda108cd6b089a0385	wrong population		Round I
Han, W. and Shi, D. and Yang, Q. and Li, X. and Zhang, J. and Peng, C. and Yan, F.	Alteration of chromosome structure impacts gene expressions implicated in pancreatic ductal adenocarcinoma cells	2024	BMC Genomics	https://www.scopus.com/inward/record.uri?eid=2-s2.0-85185962205&doi=10.1186%2fs12864-024-10109-4&partnerID=40&md5=303d448df5ee3bf7d1c6c6a8d8dab33d	wrong population		Round I
Luo, L. and Wang, X. and Liao, Y.-P. and Xu, X. and Chang, C.H. and Nel, A.E.	Reprogramming the pancreatic cancer stroma and immune landscape by a silicasome nanocarrier delivering nintedanib, a protein tyrosine kinase inhibitor	2024	Nano Today	https://www.scopus.com/inward/record.uri?eid=2-s2.0-85181666571&doi=10.1016%2fj.nantod.2023.102058&partnerID=40&md5=8f0c016251f4120763d92c0d7649a2e5	wrong population		Round I
Neuzillet, C. and Artru, P. and Assenat, E. and Edeline, J. and Adhoute, X. and Sabourin, J.-C. and Turpin, A. and Coriat, R. and Malka, D.	Optimizing Patient Pathways in Advanced Biliary Tract Cancers: Recent Advances and a French Perspective	2023	Targeted Oncology	https://www.scopus.com/inward/record.uri?eid=2-s2.0-85147585799&doi=10.1007%2fs11523-022-00942-6&partnerID=40&md5=3d701aa29eb7cc66e69f6746e773c7a4	wrong population		Round I

Shirota, H. and Komine, K. and Takahashi, M. and Takahashi, S. and Miyauchi, E. and Niizuma, H. and Tada, H. and Shimada, M. and Niihori, T. and Aoki, Y. and Sugiyama, I. and Kawamura, M. and Yasuda, J. and Suzuki, S. and Iwaya, T. and Saito, M. and Saito, T. and Shibata, H. and Furukawa, T. and Ishioka, C.	Clinical decisions by the molecular tumor board on comprehensive genomic profiling tests in Japan: A retrospective observational study	2023	Cancer Medicine	https://www.scopus.com/inward/record.uri?eid=2-s2.0-85139906025&doi=10.1002%2fcam4.5349&partnerID=40&md5=71071b4ef0ce9014a10a5ecf54d7f69d	wrong population		Round I
Ashok Kumar, P. and Serinelli, S. and Zaccarini, D.J. and Huang, R. and Danziger, N. and Janovitz, T. and Basnet, A. and Sivapiragasam, A. and Graziano, S. and Ross, J.S.	Genomic landscape of clinically advanced KRAS wild-type pancreatic ductal adenocarcinoma	2023	Frontiers in Oncology	https://www.scopus.com/inward/record.uri?eid=2-s2.0-85164425803&doi=10.3389%2ffonc.2023.1169586&partnerID=40&md5=a73fbe06c25516792bf32dbd9ee17d29	wrong population		Round I
Takamizawa, S. and Katsuya, Y. and Chen, Y.-N. and Mizuno, T. and Koyama, T. and Sudo, K. and Yoshida, T. and Kondo, S. and Iwasa, S. and Yonemori, K. and Shimizu, T. and Yamamoto, N. and Suzuki, S.	Ocular toxicity of investigational anti-cancer drugs in early phase clinical trials	2023	Investigational New Drugs	https://www.scopus.com/inward/record.uri?eid=2-s2.0-85143349368&doi=10.1007%2fs10637-022-01321-8&partnerID=40&md5=8cf57e3b7494888c66ed601cddaf588d	wrong population		Round I
Murciano-Goroff, Y.R. and Schram, A.M. and Rosen, E.Y. and Won, H. and Gong, Y. and Noronha, A.M. and Janjigian, Y.Y. and Stadler, Z.K. and Chang, J.C. and Yang, S.-R. and Mandelker, D. and Offit, K. and Berger, M.F. and Donoghue, M.T.A. and Bandlamudi, C. and Drilon, A.	Reversion mutations in germline BRCA1/2-mutant tumors reveal a BRCA-mediated phenotype in non-canonical histologies	2022	Nature Communications	https://www.scopus.com/inward/record.uri?eid=2-s2.0-85142454077&doi=10.1038%2fs41467-022-34109-8&partnerID=40&md5=fd42d548cc5e1eec59bec962f721fef1	wrong population		Round I
Imamura, T. and Ashida, R. and Ohshima, K. and Uesaka, K. and Sugiura, T. and Okamura, Y. and Ohgi, K. and Ohnami, S. and Nagashima, T. and Yamaguchi, K.	Genomic landscape of pancreatic cancer in the Japanese version of the Cancer Genome Atlas	2023	Annals of Gastroenterological Surgery	https://www.scopus.com/inward/record.uri?eid=2-s2.0-85142812586&doi=10.1002%2fags3.12636&partnerID=40&md5=eb965da9f2d07a8eea1cecfbb0ae85e1	wrong population		Round I
Patil, D. and Akolkar, D. and Nagarkar, R. and Srivastava, N. and Datta, V. and Patil, S. and Apurwa, S. and Srinivasan, A. and Datar, R.	Multi-analyte liquid biopsies for molecular pathway guided personalized treatment selection in advanced refractory cancers: A clinical utility pilot study	2022	Frontiers in Oncology	https://www.scopus.com/inward/record.uri?eid=2-s2.0-85145897528&doi=10.3389%2ffonc.2022.972322&partnerID=40&md5=bcd24576b8d0be3377b5f5aaf2fcd01a	wrong population		Round I
Mousa, H. and Al saei, A. and Razali, R.M. and Zughaier, S.M.	Vitamin D status affects proteomic profile of HDL-associated proteins and inflammatory mediators in dyslipidemia	2024	Journal of Nutritional Biochemistry	https://www.scopus.com/inward/record.uri?eid=2-s2.0-85175566589&doi=10.1016%2fj.jnutbio.2023.109472&partnerID=40&md5=a6c93ad61db3c46df534a048e42d3153	wrong population		Round I
Zhang, Z. and Wang, H. and Yan, Q. and Cui, J. and Chen, Y. and Ruan, S. and Yang, J. and Wu, Z. and Han, M. and Huang, S. and Zhou, Q. and Zhang, C. and Hou, B.	Genome-wide CRISPR/Cas9 screening for drug resistance in tumors	2023	Frontiers in Pharmacology	https://www.scopus.com/inward/record.uri?eid=2-s2.0-85178928771&doi=10.3389%2ffphar.2023.1284610&partnerID=40&md5=043da8aba8b3d751713dafd9b69b576a	wrong population		Round I

Carpenter, E.S. and Elhossiny, A.M. and Kadiyala, P. and Li, J. and McGue, J. and Griffith, B.D. and Zhang, Y. and Edwards, J. and Nelson, S. and Lima, F. and Donahue, K.L. and Du, W. and Bischoff, A.C. and Alomari, D. and Watkoske, H.R. and Mattea, M. and The, S. and Espinoza, C.E. and Barrett, M. and Sonnenday, C.J. and Olden, N. and Chen, C.-T. and Peterson, N. and Gunchick, V. and Sahai, V. and Rao, A. and Bednar, F. and Shi, J. and Frankel, T.L. and Pasca Di Magliano, M.	Analysis of Donor Pancreata Defines the Transcriptomic Signature and Microenvironment of Early Neoplastic Lesions	2023	Cancer Discovery	https://www.scopus.com/inward/record.uri?eid=2-s2.0-85159743547&doi=10.1158%2f2159-8290.CD-23-0013&partnerID=40&md5=051f5470b10490d20bef2ab63159fdcb	wrong population		Round I
Tie, Y. and Tang, F. and Wei, Y.-Q. and Wei, X.-W.	Immunosuppressive cells in cancer: mechanisms and potential therapeutic targets	2022	Journal of Hematology and Oncology	https://www.scopus.com/inward/record.uri?eid=2-s2.0-85130251285&doi=10.1186%2fs13045-022-01282-8&partnerID=40&md5=683e55cde02b6098c303cf45c9bb531f	wrong population		Round I
Gu, Y. and Becker, M.A. and MÃ¼ller, L. and Reuss, K. and Umlauf, F. and Tang, T. and Menger, M.D. and Laschke, M.W.	MicroRNAs in Tumor Endothelial Cells: Regulation, Function and Therapeutic Applications	2023	Cells	https://www.scopus.com/inward/record.uri?eid=2-s2.0-85164499940&doi=10.3390%2fcells12131692&partnerID=40&md5=0b79605f4d2e83b9d2767ab29f0772a7	wrong population		Round I
Zhou, D. and Zheng, L.	Recent advances in cancer-associated fibroblast: Biomarkers, signaling pathways, and therapeutic opportunities	2024	Chinese Medical Journal	https://www.scopus.com/inward/record.uri?eid=2-s2.0-85188521702&doi=10.1097%2fCM9.0000000000003031&partnerID=40&md5=93bbeb9861ff66bfe2da36a5a4fe475f	wrong population		Round I
Aoyama, R. and Nishikubo, H. and Kawabata, K. and Kanei, S. and Yamamoto, Y. and Nishimura, S. and Yashiro, M.	Clinical Significance of Multi-Cancer Genome Profiling: Data from a Single Hospital in Japan	2024	Cancer Genomics and Proteomics	https://www.scopus.com/inward/record.uri?eid=2-s2.0-85181395095&doi=10.21873%2fcgp.20431&partnerID=40&md5=38e67380f00c22a6dbb53348ec8e5154	wrong population		Round I
Lam, R.C.T. and Johnson, D. and Lam, G. and Li, M.L.Y. and Wong, J.W.L. and Lam, W.K.J. and Chan, K.C.A. and Ma, B.	Clinical applications of circulating tumor-derived DNA in the management of gastrointestinal cancers â€“ current evidence and future directions	2022	Frontiers in Oncology	https://www.scopus.com/inward/record.uri?eid=2-s2.0-85139828829&doi=10.3389%2ffonc.2022.970242&partnerID=40&md5=1b5f4f08587cceeeb74f90495873e983	wrong population		Round I
Ribatti, D. and Annese, T. and Tamma, R.	Vascular co-option in resistance to anti-angiogenic therapy	2023	Frontiers in Oncology	https://www.scopus.com/inward/record.uri?eid=2-s2.0-85180660226&doi=10.3389%2ffonc.2023.1323350&partnerID=40&md5=6ad4fd7be97fd7bab32347791d294bc9	wrong population		Round I
Ruff, S.M. and Pawlik, T.M.	Clinical management of intrahepatic cholangiocarcinoma: surgical approaches and systemic therapies	2024	Frontiers in Oncology	https://www.scopus.com/inward/record.uri?eid=2-s2.0-85184682909&doi=10.3389%2ffonc.2024.1321683&partnerID=40&md5=26ce49ce859e70a076aa86adb52a5e1f	wrong population		Round I
Lee, M.S. and Kaseb, A.O. and Pant, S.	The Emerging Role of Circulating Tumor DNA in Non-Colorectal Gastrointestinal Cancers	2023	Clinical Cancer Research	https://www.scopus.com/inward/record.uri?eid=2-s2.0-85169501401&doi=10.1158%2f1078-0432.CCR-22-3626&partnerID=40&md5=f7977fc8ade84c1686ad2a41f5c3802b	wrong population		Round I

VÃzkeleti, L. and SpisÃ¡k, S.	Rewired Metabolism Caused by the Oncogenic Deregulation of MYC as an Attractive Therapeutic Target in Cancers	2023	Cells	https://www.scopus.com/inward/record.uri?eid=2-s2.0-85164715646&doi=10.3390%2fcells12131745&partnerID=40&md5=fcbb8bfe9599f3ea3fe1bd0de37e3bc4	wrong population		Round I
Wang, C.-X. and Wang, T.-T. and Zhang, K.-D. and Li, M.-Y. and Shen, Q.-C. and Lu, S.-Y. and Zhang, J.	Pan-KRAS inhibitors suppress proliferation through feedback regulation in pancreatic ductal adenocarcinoma	2022	Acta Pharmacologica Sinica	https://www.scopus.com/inward/record.uri?eid=2-s2.0-85127350733&doi=10.1038%2fs41401-022-00897-4&partnerID=40&md5=6bf3f4c087c5c07a629826dfda6bf315	wrong population		Round I
Morishita, A. and Oura, K. and Tadokoro, T. and Shi, T. and Fujita, K. and Tani, J. and Atsukawa, M. and Masaki, T.	Galectin-9 in Gastroenterological Cancer	2023	International Journal of Molecular Sciences	https://www.scopus.com/inward/record.uri?eid=2-s2.0-85152335041&doi=10.3390%2fijms24076174&partnerID=40&md5=7d4c911024b1359f118e20ea144b6cdf	wrong population		Round I
Jabir, N.R. and Rehman, M.T. and AlAjmi, M.F. and Ahmed, B.A. and Tabrez, S.	Prioritization of bioactive compounds envisaging yohimbine as a multi targeted anticancer agent: insight from molecular docking and molecular dynamics simulation	2023	Journal of Biomolecular Structure and Dynamics	https://www.scopus.com/inward/record.uri?eid=2-s2.0-85144249469&doi=10.1080%2f07391102.2022.2158137&partnerID=40&md5=744581f43c02f72a8bf1bf6ef9face35	wrong population		Round I
McGrath, M.K. and Abolhassani, A. and Guy, L. and Elshazly, A.M. and Barrett, J.T. and Mivechi, N.F. and Gewirtz, D.A. and Schoenlein, P.V.	Autophagy and senescence facilitate the development of antiestrogen resistance in ER positive breast cancer	2024	Frontiers in Endocrinology	https://www.scopus.com/inward/record.uri?eid=2-s2.0-85189010367&doi=10.3389%2ffendo.2024.1298423&partnerID=40&md5=97b3542bb7db4013ddcc7438cb991701	wrong population		Round I
Hussain, S. and Mursal, M. and Verma, G. and Hasan, S.M. and Khan, M.F.	Targeting oncogenic kinases: Insights on FDA approved tyrosine kinase inhibitors	2024	European Journal of Pharmacology	https://www.scopus.com/inward/record.uri?eid=2-s2.0-85187659853&doi=10.1016%2fj.ejphar.2024.176484&partnerID=40&md5=252401a7c70b8f47ecbc14451dc4519f	wrong population		Round I
Huang, Y. and Zhang, W. and Xu, C. and Li, Q. and Zhang, W. and Xu, W. and Zhang, M.	Presence of PD-1 similarity genes in monocytes may promote the development of type 1 diabetes mellitus and poor prognosis of pancreatic cancer	2023	BMJ Open Diabetes Research and Care	https://www.scopus.com/inward/record.uri?eid=2-s2.0-85157979643&doi=10.1136%2fbmjdrc-2022-003196&partnerID=40&md5=f0ef837d0972a38ba8fa1679133dbbd2	wrong population		Round I
Singh, G. and Thakur, N. and Kumar, U.	RAS: Circuitry and therapeutic targeting	2023	Cellular Signalling	https://www.scopus.com/inward/record.uri?eid=2-s2.0-85140962238&doi=10.1016%2fj.cellsig.2022.110505&partnerID=40&md5=a4d0a449ff0dc763a1c1db7b1e8724ef	wrong population		Round I
Laface, C. and Memeo, R.	Clinical Updates for Gastrointestinal Malignancies	2023	Journal of Personalized Medicine	https://www.scopus.com/inward/record.uri?eid=2-s2.0-85172928398&doi=10.3390%2fjpm13091424&partnerID=40&md5=7bec70c309d339494e9e6cde4428f474	wrong population		Round I
Qian, C. and Liu, C. and Liu, W. and Zhou, R. and Zhao, L.	Targeting vascular normalization: a promising strategy to improve immuneâ€“vascular crosstalk in cancer immunotherapy	2023	Frontiers in Immunology	https://www.scopus.com/inward/record.uri?eid=2-s2.0-85180863737&doi=10.3389%2ffimmu.2023.1291530&partnerID=40&md5=bb0a89d1202529beb42bf3d8f702a375	wrong population		Round I
Vera, R. and Ibarrola-de-AndrÃ©s, C. and Adeva, J. and PÃ©rez-Rojas, J. and GarcÃa-Alfonso, P. and RodrÃguez-Gil, Y. and Macarulla, T. and Serrano-PiÃ±ol, T. and MondÃ©jar, R. and Madrigal-Rubiales, B.	Expert consensus of the Spanish Society of Pathology and the Spanish Society of Medical Oncology on the determination of biomarkers in pancreatic and biliary tract cancer	2022	Clinical and Translational Oncology	https://www.scopus.com/inward/record.uri?eid=2-s2.0-85137031845&doi=10.1007%2fs12094-022-02873-0&partnerID=40&md5=b801988c25d79e32086ae63d0d910d22	wrong population		Round I

Simbolo, M. and Silvestris, N. and Malleo, G. and Mafficini, A. and Maggino, L. and Cocomazzi, A. and Veghini, L. and Mombello, A. and Pezzini, F. and Sereni, E. and Martelli, F.M. and Gkountakos, A. and Ciaparrone, C. and Piredda, M.L. and Ingravallo, G. and Paolino, G. and Nappo, F. and Rapposelli, I.G. and Frassinetti, L. and Saragoni, L. and Lonardi, S. and Pea, A. and Paiella, S. and Fassan, M. and Brunetti, O. and Cingarlini, S. and Salvia, R. and Milella, M. and Corbo, V. and Lawlor, R.T. and Scarpa, A. and Luchini, C.	Clinical and Genomic Characterization of Pancreatic Ductal Adenocarcinoma with Signet-Ring/Poorly Cohesive Cells	2023	Modern Pathology	https://www.scopus.com/inward/record.uri?eid=2-s2.0-85172424705&doi=10.1016%2fj.modpat.2023.100251&partnerID=40&md5=13cb7d782efbb93e59cc518a5a7cdcb1	wrong population		Round I
Karimi, E. and Dehghani, A. and Azari, H. and Zarei, M. and Shekari, M. and Mousavi, P.	Molecular Mechanisms of miR-214 Involved in Cancer and Drug Resistance	2023	Current Molecular Medicine	https://www.scopus.com/inward/record.uri?eid=2-s2.0-85161112725&doi=10.2174%2f1566524022666220428112744&partnerID=40&md5=85172985240281099e24015069793950	wrong population		Round I
Demols, A. and Rocq, L. and Perez-Casanova, L. and Charry, M. and De NÃ¨ve, N. and Ramadhan, A. and Van Campenhout, C. and De Clercq, S. and Maris, C. and Closset, J. and Lucidi, V. and Salmon, I. and D'haene, N.	A Two-Step Diagnostic Approach for NTRK Gene Fusion Detection in Biliary Tract and Pancreatic Adenocarcinomas	2023	Oncologist	https://www.scopus.com/inward/record.uri?eid=2-s2.0-85164238048&doi=10.1093%2foncolo%2foyad075&partnerID=40&md5=8351bd78d1ded8c3ff331c7f6c7c38d9	wrong population		Round I
Mota, J. and Lima, A.M.M. and Gomes, J.I.S. and Souza de Andrade, M. and Brito, H.O. and Silva, M.M.A.L. and Faustino-Rocha, A.I. and Oliveira, P.A. and Lopes, F.F. and Gil da Costa, R.M.	Klotho in Cancer: Potential Diagnostic and Prognostic Applications	2023	Diagnostics	https://www.scopus.com/inward/record.uri?eid=2-s2.0-85176338013&doi=10.3390%2fdiagnostics13213357&partnerID=40&md5=e71b547bfb3ed35a310158ef3202ba29	wrong population		Round I
Mestre-Alagarda, C. and Srirajaskanthan, R. and Zen, Y. and Giwa, M. and Howard, M. and Ooft, M.L.	Genetic and epigenetic prognosticators of neuroendocrine tumours of the GI tract, liver, biliary tract and pancreas: A systematic review and meta-analysis	2024	Histopathology	https://www.scopus.com/inward/record.uri?eid=2-s2.0-85167698784&doi=10.1111%2fhis.15025&partnerID=40&md5=411004d9bc229a945e1f5de0712474b3	wrong population		Round I
Zhen, D.B. and Safyan, R.A. and Konick, E.Q. and Nguyen, R. and Prichard, C.C. and Chiorean, E.G.	The role of molecular testing in pancreatic cancer	2023	Therapeutic Advances in Gastroenterology	https://www.scopus.com/inward/record.uri?eid=2-s2.0-85159103267&doi=10.1177%2f17562848231171456&partnerID=40&md5=8f812c3a3e8bd6b1ee23d776ab5b6451	wrong population		Round I
Taherian, M. and Wang, H. and Wang, H.	Pancreatic Ductal Adenocarcinoma: Molecular Pathology and Predictive Biomarkers	2022	Cells	https://www.scopus.com/inward/record.uri?eid=2-s2.0-85139777434&doi=10.3390%2fcells11193068&partnerID=40&md5=83ac289c69bc27477c81e4b50fd4c5b4	wrong population		Round I

Chen, M. and Jian, D. and Sidorov, M. and Woo, R.W.L. and Kim, A. and Stone, D.E. and Nazarian, A. and Nosrati, M. and Ice, R.J. and de Semir, D. and Dar, A.A. and LuÅ¡trik, R. and KokoÅ¡ar, J. and Ausec, L. and Rowbotham, M.C. and Tranah, G.J. and Kashani-Sabet, M. and Soroceanu, L. and McAllister, S.D. and Desprez, P.-Y.	Pitfalls and Rewards of Setting Up a Liquid Biopsy Approach for the Detection of Driver Mutations in Circulating Tumor DNAs: Our Institutional Experience	2022	Journal of Personalized Medicine	https://www.scopus.com/inward/record.uri?eid=2-s2.0-85149568683&doi=10.3390%2fjpm12111845&partnerID=40&md5=a5c358cd3c2ccbf01f7c54c6e87f9b6c	wrong population		Round I
Lee, Y.E. and Go, G.-Y. and Koh, E.-Y. and Yoon, H.-N. and Seo, M. and Hong, S.-M. and Jeong, J.H. and Kim, J.-C. and Cho, D. and Kim, T.S. and Kim, S.C. and Jun, E. and Jang, M.	Synergistic therapeutic combination with a CAF inhibitor enhances CAR-NK-mediated cytotoxicity via reduction of CAF-released IL-6	2023	Journal for ImmunoTherapy of Cancer	https://www.scopus.com/inward/record.uri?eid=2-s2.0-85148969089&doi=10.1136%2fjitc-2022-006130&partnerID=40&md5=c8a11d7e350ff8a30649fdd6dadd5a15	wrong population		Round I
Morrison, J and Thoma, C and Goodall, RJ and Lyons, TJ and Gaitskell, K and Wiggans, AJ and Bryant, A	Epidermal growth factor receptor blockers for the treatment of ovarian cancer	2018	Cochrane Database of Systematic Reviews	http://dx.doi.org/10.1002/14651858.CD007927.pub4	wrong population		Round I
Huang, J. and Xiao, K.	Nanoparticles-Based Strategies to Improve the Delivery of Therapeutic Small Interfering RNA in Precision Oncology	2022	Pharmaceutics	https://www.scopus.com/inward/record.uri?eid=2-s2.0-85137399902&doi=10.3390%2fpharmaceutics14081586&partnerID=40&md5=eaa23adfaa674744bbf5bd691208ccfd	wrong population		Round I
Zahra, F.T. and Sajib, M.S. and Mikelis, C.M.	Role of bfgf in acquired resistance upon anti-vegf therapy in cancer	2021	Cancers	https://www.scopus.com/inward/record.uri?eid=2-s2.0-85102684090&doi=10.3390%2fcancers13061422&partnerID=40&md5=3808bdf4929bd7520c627a4cdb40ab24	wrong population		Round I
Mateus, D. and Wiedlocha, A.	FGFâ€"FGFR signaling promotes the development of invasive cervical cancer	2022	FEBS Journal	https://www.scopus.com/inward/record.uri?eid=2-s2.0-85124502732&doi=10.1111%2ffebs.16384&partnerID=40&md5=e5fa85abbb00ea82ffd18217ad2b4573	wrong population		Round I
Zheng, X. and Xu, H. and Lin, T. and Tan, P. and Xiong, Q. and Yi, X. and Qiu, S. and Yang, L. and Shen, B. and Ai, J. and Wei, Q.	CD93 orchestrates the tumor microenvironment and predicts the molecular subtype and therapy response of bladder cancer	2022	Computers in Biology and Medicine	https://www.scopus.com/inward/record.uri?eid=2-s2.0-85133443901&doi=10.1016%2fj.compbiomed.2022.105727&partnerID=40&md5=e05ffe0e187bb32022b7301db07fb6af	wrong population		Round I
Van Der Wijngaart, H. and Hoes, L.R. and Van Berge Henegouwen, J.M. and Van Der Velden, D.L. and Zeverijn, L.J. and Roepman, P. and Van Werkhoven, E. and De Leng, W.W.J. and Jansen, A.M.L. and Mehra, N. and Robbrecht, D.G.J. and Labots, M. and De Groot, D.J.A. and Hoeben, A. and Hamberg, P. and Gelderblom, H. and Voest, E.E. and Verheul, H.M.W.	Patients with Biallelic BRCA1/2 Inactivation respond to Olaparib treatment across Histologic tumor types	2021	Clinical Cancer Research	https://www.scopus.com/inward/record.uri?eid=2-s2.0-85119927163&doi=10.1158%2f1078-0432.CCR-21-1104&partnerID=40&md5=7e37c1dd62f655bd6c2a0ddf3377b681	wrong population		Round I
Umemoto, K. and Yamamoto, H. and Oikawa, R. and Takeda, H. and Doi, A. and Horie, Y. and Arai, H. and Ogura, T. and Mizukami, T. and Izawa, N. and Moore, J.A. and Sokol, E.S. and Sunakawa, Y.	The Molecular Landscape of Pancreatobiliary Cancers for Novel Targeted Therapies from Real-World Genomic Profiling	2022	Journal of the National Cancer Institute	https://www.scopus.com/inward/record.uri?eid=2-s2.0-85138443916&doi=10.1093%2fjnci%2fdjac106&partnerID=40&md5=4c2c395fd04b066529cfd1bfb085b427	wrong population		Round I

Catenacci, D.V.T.	Exploring New Approaches for Locally Advanced Gastroesophageal Adenocarcinomas: TNT, Irinotecan, and ctDNA	2021	Clinical Cancer Research	https://www.scopus.com/inward/record.uri?eid=2-s2.0-85120502527&doi=10.1158%2f1078-0432.CCR-21-2777&partnerID=40&md5=9550fb1e26c17e63706517e45ed558df	wrong population		Round I
PorÄ™bska, N. and PoÅºniak, M. and Matynia, A. and Å»ukowska, D. and Zakrzewska, M. and Otlewski, J. and OpaliÅ„ski, Å.	Galectins as modulators of receptor tyrosine kinases signaling in health and disease	2021	Cytokine and Growth Factor Reviews	https://www.scopus.com/inward/record.uri?eid=2-s2.0-85104134890&doi=10.1016%2fj.cytogfr.2021.03.004&partnerID=40&md5=bd09ead09b33735fb9d77324abb1bad5	wrong population		Round I
Huang, H.-M. and Li, H.-X.	Tumor heterogeneity and the potential role of liquid biopsy in bladder cancer	2021	Cancer Communications	https://www.scopus.com/inward/record.uri?eid=2-s2.0-85098291125&doi=10.1002%2fcac2.12129&partnerID=40&md5=320a150153e7770ccbc32c698fe0cde3	wrong population		Round I
Philip, P.A. and Azar, I. and Xiu, J. and Hall, M.J. and Hendifar, A.E. and Lou, E. and Hwang, J.J. and Gong, J. and Feldman, R. and Ellis, M. and Stafford, P. and Spetzler, D. and Khushman, M.M. and Sohal, D. and Lockhart, A.C. and Weinberg, B.A. and El-Deiry, W.S. and Marshall, J. and Shields, A.F. and Korn, W.M.	Molecular Characterization of KRAS Wild-type Tumors in Patients with Pancreatic Adenocarcinoma	2022	Clinical Cancer Research	https://www.scopus.com/inward/record.uri?eid=2-s2.0-85131903186&doi=10.1158%2f1078-0432.CCR-21-3581&partnerID=40&md5=350a7d7171f5d1ec8daf191a8cd72284	wrong population		Round I
Xu, Z.-H. and Wang, W.-Q. and Liu, L. and Lou, W.-H.	A special subtype: Revealing the potential intervention and great value of KRAS wildtype pancreatic cancer	2022	Biochimica et Biophysica Acta - Reviews on Cancer	https://www.scopus.com/inward/record.uri?eid=2-s2.0-85132790531&doi=10.1016%2fj.bbcan.2022.188751&partnerID=40&md5=19bba0fd71d20d22408760bafc5871bf	wrong population		Round I
Matsudera, S. and Kano, Y. and Aoyagi, Y. and Tohyama, K. and Takahashi, K. and Kumaki, Y. and Mitsumura, T. and Kimura, K. and Onishi, I. and Takemoto, A. and Ban, D. and Ono, H. and Kudo, A. and Oshima, N. and Ogino, K. and Watanabe, S. and Tani, Y. and Yamaguchi, T. and Nakajima, M. and Morita, S. and Yamaguchi, S. and Takagi, M. and Ishikawa, T. and Nakagawa, T. and Okamoto, K. and Uetake, H. and Tanabe, M. and Miyake, S. and Tsuchioka, T. and Kojima, K. and Ikeda, S.	A Pilot Study Analyzing the Clinical Utility of Comprehensive Genomic Profiling Using Plasma Cell-Free DNA for Solid Tumor Patients in Japan (PROFILE Study)	2021	Annals of Surgical Oncology	https://www.scopus.com/inward/record.uri?eid=2-s2.0-85103150897&doi=10.1245%2fs10434-021-09856-5&partnerID=40&md5=ab0a9c23a980d1c8973f09c6c870119f	wrong population		Round I
Vinusri, S. and Gnanam, R. and Caroline, R. and Santhanakrishnan, V.P. and Kandavelmani, A.	Anticancer Potential of Hydroxychavicol Derived from Piper betle L: An in Silico and Cytotoxicity Study	2022	Nutrition and Cancer	https://www.scopus.com/inward/record.uri?eid=2-s2.0-85131924603&doi=10.1080%2f01635581.2022.2085310&partnerID=40&md5=563d2603b75d1ab262b97fbd9fb998a1	wrong population		Round I
AlSudais, H. and Rajgara, R. and Saleh, A. and Wiper-Bergeron, N.	C/EBPÎ² promotes the expression of atrophy-inducing factors by tumours and is a central regulator of cancer cachexia	2022	Journal of Cachexia, Sarcopenia and Muscle	https://www.scopus.com/inward/record.uri?eid=2-s2.0-85122876770&doi=10.1002%2fjcsm.12909&partnerID=40&md5=6368f824de88fa7ac32e9b741ea1f9bf	wrong population		Round I

Sompel, K. and Elango, A. and Smith, A.J. and Tennis, M.A.	Cancer chemoprevention through Frizzled receptors and EMT	2021	Discover Oncology	https://www.scopus.com/inward/record.uri?eid=2-s2.0-85114684149&doi=10.1007%2fs12672-021-00429-2&partnerID=40&md5=94ed873972b8b73324e2f045aedb6d64	wrong population		Round I
LV, B. and Wang, Y. and Ma, D. and Cheng, W. and Liu, J. and Yong, T. and Chen, H. and Wang, C.	Immunotherapy: Reshape the Tumor Immune Microenvironment	2022	Frontiers in Immunology	https://www.scopus.com/inward/record.uri?eid=2-s2.0-85134473590&doi=10.3389%2ffimmu.2022.844142&partnerID=40&md5=6f7a161debc62e0ad3d85a87e4bb7a74	wrong population		Round I
Sigorski, D. and RÃ³Å¼anowski, P. and IÅ¼ycka-Åšwieszewska, E. and Wiktorska, K.	Antibodyâ€"Drug Conjugates in Uro-Oncology	2022	Targeted Oncology	https://www.scopus.com/inward/record.uri?eid=2-s2.0-85130197599&doi=10.1007%2fs11523-022-00872-3&partnerID=40&md5=9721dd917b37512d0ef6c4b5ff98cbab	wrong population		Round I
Paschold, L. and Binder, M.	Circulating Tumor DNA in Gastric and Gastroesophageal Junction Cancer	2022	Current Oncology	https://www.scopus.com/inward/record.uri?eid=2-s2.0-85125595706&doi=10.3390%2fcurroncol29030120&partnerID=40&md5=96e6c78aeb08d938ee778d90b63f163b	wrong population		Round I
Wang, S. and Chen, J. and Guo, X.-Z.	KAI1/CD82 gene and autotaxin-lysophosphatidic acid axis in gastrointestinal cancers	2022	World Journal of Gastrointestinal Oncology	https://www.scopus.com/inward/record.uri?eid=2-s2.0-85135798827&doi=10.4251%2fwjgo.v14.i8.1388&partnerID=40&md5=923b443176d05fc2a68c6a325f1fca9d	wrong population		Round I
Fanfani, V. and Citi, L. and Harris, A.L. and Pezzella, F. and Stracquadanio, G.	The landscape of the heritable cancer genome	2021	Cancer Research	https://www.scopus.com/inward/record.uri?eid=2-s2.0-85106191611&doi=10.1158%2f0008-5472.CAN-20-3348&partnerID=40&md5=5c68f5b984bfc9c9b85872c7c1b0b6b8	wrong population		Round I
Pawar, S. and Sharma, A.	Molecular targets in GI malignancies - A pathologist's perspective	2021	Indian Journal of Pathology and Microbiology	https://www.scopus.com/inward/record.uri?eid=2-s2.0-85108144805&doi=10.4103%2fIJPM.IJPM_1239_20&partnerID=40&md5=3abb93299600161731754ba1e9b3c4d9	wrong population		Round I
Hu, H. and Chen, Y. and Tan, S. and Wu, S. and Huang, Y. and Fu, S. and Luo, F. and He, J.	The Research Progress of Antiangiogenic Therapy, Immune Therapy and Tumor Microenvironment	2022	Frontiers in Immunology	https://www.scopus.com/inward/record.uri?eid=2-s2.0-85126192987&doi=10.3389%2ffimmu.2022.802846&partnerID=40&md5=07a7543747ad58879b26088a47c6dd93	wrong population		Round I
Saleh, M.M. and Abuirmeileh, A.N. and Al-Rousan, R.M. and Abudoleh, S.M. and Hassouneh, L.K. and Zihlif, M.A. and Taha, M.O. and Abutayeh, R.F. and Mansour, H. and Abu-Irmaileh, B.	Biological Evaluation and Reverse Pharmacophore Mapping of Innovative Bis-Triazoles as Promising Anticancer Agents	2022	Open Medicinal Chemistry Journal	https://www.scopus.com/inward/record.uri?eid=2-s2.0-85138620353&doi=10.2174%2f18741045-v16-e2207200&partnerID=40&md5=de92bf685cd2e6eb49f80138c9c30bcc	wrong population		Round I

Roepman, P. and de Bruijn, E. and van Lieshout, S. and Schoenmaker, L. and Boelens, M.C. and Dubbink, H.J. and Geurts-Giele, W.R.R. and Groenendijk, F.H. and Huibers, M.M.H. and Kranendonk, M.E.G. and Roemer, M.G.M. and Samsom, K.G. and Steehouwer, M. and de Leng, W.W.J. and Hoischen, A. and Ylstra, B. and Monkhorst, K. and van der Hoeven, J.J.M. and Cuppen, E.	Clinical Validation of Whole Genome Sequencing for Cancer Diagnostics	2021	Journal of Molecular Diagnostics	https://www.scopus.com/inward/record.uri?eid=2-s2.0-85109083940&doi=10.1016%2fj.jmoldx.2021.04.011&partnerID=40&md5=7125d1ed9ed8b3c2003b6c35ff252db1	wrong population		Round I
Rizzo, A. and Brandi, G.	Neoadjuvant therapy for cholangiocarcinoma: A comprehensive literature review	2021	Cancer Treatment and Research Communications	https://www.scopus.com/inward/record.uri?eid=2-s2.0-85102795765&doi=10.1016%2fj.ctarc.2021.100354&partnerID=40&md5=a98fb93ae8174c9071060b1ecd38b972	wrong population		Round I
Zalyte, E. and Cicenas, J.	Starvation mediates pancreatic cancer cell sensitivity to ferroptosis via ERK1/2, JNK and changes in the cell mesenchymal state	2022	International Journal of Molecular Medicine	https://www.scopus.com/inward/record.uri?eid=2-s2.0-85129351165&doi=10.3892%2fIJMM.2022.5140&partnerID=40&md5=521a652398c63c2e2ca99c6ece58c7a0	wrong population		Round I
Shichi, Y. and Gomi, F. and Sasaki, N. and Nonaka, K. and Arai, T. and Ishiwata, T.	Epithelial and Mesenchymal Features of Pancreatic Ductal Adenocarcinoma Cell Lines in Two- and Three-Dimensional Cultures	2022	Journal of Personalized Medicine	https://www.scopus.com/inward/record.uri?eid=2-s2.0-85136604174&doi=10.3390%2fjpm12050746&partnerID=40&md5=9958c1775f79e51e5985b0f4e5dd5a8a	wrong population		Round I
Shoucair, S. and Chen, J. and Martinson, J.R. and Habib, J.R. and Kinny-KÃ¶ster, B. and Pu, N. and Van Oosten, A.F. and Javed, A.A. and Shin, E.J. and Ali, S.Z. and Lafaro, K.J. and Wolfgang, C.L. and He, J. and Yu, J.	Association of Matrix Metalloproteinase 7 Expression With Pathologic Response After Neoadjuvant Treatment in Patients With Resected Pancreatic Ductal Adenocarcinoma	2022	JAMA Surgery	https://www.scopus.com/inward/record.uri?eid=2-s2.0-85131645241&doi=10.1001%2fjamasurg.2022.1362&partnerID=40&md5=1f70f20a40875ec8c9f040380818b406	wrong population		Round I
Ramseier, J.Y. and Perkins, S.H.	Genodermatoses associated with melanocytic nevi	2022	Clinics in Dermatology	https://www.scopus.com/inward/record.uri?eid=2-s2.0-85128171484&doi=10.1016%2fj.clindermatol.2022.02.007&partnerID=40&md5=9cbec7d7aef8f2323c16691e7f669452	wrong population		Round I
Anoshkin, K. and Vasilyev, I. and Karandasheva, K. and Shugay, M. and Kudryavtseva, V. and Egorov, A. and Gurevich, L. and Mironova, A. and Serikov, A. and Kutsev, S. and Strelnikov, V.	New Regions With Molecular Alterations in a Rare Case of Insulinomatosis: Case Report With Literature Review	2021	Frontiers in Endocrinology	https://www.scopus.com/inward/record.uri?eid=2-s2.0-85118562298&doi=10.3389%2ffendo.2021.760154&partnerID=40&md5=251bc47a679fe99429f04270d90d8ad3	wrong population		Round I
Wang, Y. and Wang, Y. and Wan, R. and Hu, C. and Lu, Y.	Profilin 1 protein and its implications for cancers	2021	ONCOLOGY (United States)	https://www.scopus.com/inward/record.uri?eid=2-s2.0-85111662798&doi=10.46883%2fONC.2021.3507.0402&partnerID=40&md5=d0d86c31641144d01ea94c3e43e80ad2	wrong population		Round I
Ewendt, F. and Feger, M. and FÃ¶ller, M.	Role of Fibroblast Growth Factor 23 (FGF23) and Î±Klotho in Cancer	2021	Frontiers in Cell and Developmental Biology	https://www.scopus.com/inward/record.uri?eid=2-s2.0-85102034078&doi=10.3389%2ffcell.2020.601006&partnerID=40&md5=36aa644842457b0690542a9423876fb0	wrong population		Round I

Frezoulis, P. and Harper, A.	The role of toceranib phosphate in dogs with non-mast cell neoplasia: A systematic review	2022	Veterinary and Comparative Oncology	https://www.scopus.com/inward/record.uri?eid=2-s2.0-85122769605&doi=10.1111%2fvco.12799&partnerID=40&md5=cd6e38b7a5ac86445f8c34e0e3be7ac5	wrong population		Round I
Salati, M. and Rizzo, A.	The Last Dance for Chemotherapy Intensification in Non-Asian Advanced Biliary Tract Cancers?	2022	Journal of Clinical Oncology	https://www.scopus.com/inward/record.uri?eid=2-s2.0-85128493815&doi=10.1200%2fJCO.21.02509&partnerID=40&md5=c3509a1dcd13f9e5e93e7bf6b9053261	wrong population		Round I
Takano, S. and Fukasawa, M. and Shindo, H. and Takahashi, E. and Fukasawa, Y. and Kawakami, S. and Hayakawa, H. and Kuratomi, N. and Kadokura, M. and Maekawa, S. and Enomoto, N.	Digital next-generation sequencing of cell-free DNA for pancreatic cancer	2021	JGH Open	https://www.scopus.com/inward/record.uri?eid=2-s2.0-85102818596&doi=10.1002%2fjgh3.12530&partnerID=40&md5=fea6675bb15631bf55efd82776859fcf	wrong population		Round I
Bayle, A. and Martin-Romano, P. and Loriot, Y.	FIGHT against FGF/FGFR alterations: what are the next steps?	2022	Annals of Oncology	https://www.scopus.com/inward/record.uri?eid=2-s2.0-85128322747&doi=10.1016%2fj.annonc.2022.03.014&partnerID=40&md5=cd90bc1cadb00e7261301194a1093bf4	wrong population		Round I
Yang, X. and Qin, C. and Zhao, B. and Li, T. and Wang, Y. and Li, Z. and Li, T. and Wang, W.	Long Noncoding RNA and Circular RNA: Two Rising Stars in Regulating Epithelial-Mesenchymal Transition of Pancreatic Cancer	2022	Frontiers in Oncology	https://www.scopus.com/inward/record.uri?eid=2-s2.0-85132936391&doi=10.3389%2ffonc.2022.910678&partnerID=40&md5=88c8fb5ecb9662a3f6cd1faedd38aa34	wrong population		Round I
Chung, C. and Galvin, R. and Achenbach, E. and Dziadkowiec, O. and Sen, S.	Characterization of Blood-Based Molecular Profiling in Pancreatic Adenocarcinoma	2021	ONCOLOGY (United States)	https://www.scopus.com/inward/record.uri?eid=2-s2.0-85123737160&doi=10.46883%2f2021.25920931&partnerID=40&md5=73df29e907b39afc48ef8751f3b39fcf	wrong population		Round I
Nandi, S. and Dey, R. and Samadder, A. and Saxena, A. and Saxena, A.K.	Natural Sourced Inhibitors of EGFR, PDGFR, FGFR and VEGFR-Mediated Signaling Pathways as Potential Anticancer Agents	2022	Current Medicinal Chemistry	https://www.scopus.com/inward/record.uri?eid=2-s2.0-85122261546&doi=10.2174%2f0929867328666210303101345&partnerID=40&md5=64e6ce086fab7d25856c9ffc219c543d	wrong population		Round I
Paolino, G. and Esposito, I. and Hong, S.-M. and Basturk, O. and Mattiolo, P. and Kaneko, T. and Veronese, N. and Scarpa, A. and Adsay, V. and Luchini, C.	Intraductal tubulopapillary neoplasm (ITPN) of the pancreas: a distinct entity among pancreatic tumors	2022	Histopathology	https://www.scopus.com/inward/record.uri?eid=2-s2.0-85130685113&doi=10.1111%2fhis.14698&partnerID=40&md5=d26da4227c967596350ea6dbf5a551ff	wrong population		Round I
Huang, L. and Guo, Z. and Wang, F. and Fu, L.	KRAS mutation: from undruggable to druggable in cancer	2021	Signal Transduction and Targeted Therapy	https://www.scopus.com/inward/record.uri?eid=2-s2.0-85119354652&doi=10.1038%2fs41392-021-00780-4&partnerID=40&md5=f2d4f884172d3a4290152fac6ea384a2	wrong population		Round I
Sobhani, N. and Fassl, A. and Mondani, G. and Generali, D. and Otto, T.	Targeting aberrant FGFR signaling to overcome CDK4/6 inhibitor resistance in breast cancer	2021	Cells	https://www.scopus.com/inward/record.uri?eid=2-s2.0-85100965893&doi=10.3390%2fcells10020293&partnerID=40&md5=ecf2131a2c9259160715db36560bcb41	wrong population		Round I
Zhou, M. and Wang, C. and Lu, S. and Xu, Y. and Li, Z. and Jiang, H. and Ma, Y.	Tumor-associated macrophages in cholangiocarcinoma: complex interplay and potential therapeutic target	2021	EBioMedicine	https://www.scopus.com/inward/record.uri?eid=2-s2.0-85105943636&doi=10.1016%2fj.ebiom.2021.103375&partnerID=40&md5=adaeecbff9db03f80741f1e83e5614b8	wrong population		Round I

Awasthi, N. and Schwarz, M.A. and Zhang, C. and Klinz, S.G. and Meyer-Losic, F. and Beaufils, B. and Thiagalingam, A. and Schwarz, R.E.	Augmenting Experimental Gastric Cancer Activity of Irinotecan through Liposomal Formulation and Antiangiogenic Combination Therapy	2022	Molecular Cancer Therapeutics	https://www.scopus.com/inward/record.uri?eid=2-s2.0-85134083503&doi=10.1158%2f1535-7163.MCT-21-0860&partnerID=40&md5=8b2cc5a94dd9a27e2cc554c5049fe879	wrong population		Round I
Zhang, Y. and Sun, J. and Song, Y. and Gao, P. and Wang, X. and Chen, M. and Li, Y. and Wu, Z.	Roles of fusion genes in digestive system cancers: Dawn for cancer precision therapy	2022	Critical Reviews in Oncology/Hematology	https://www.scopus.com/inward/record.uri?eid=2-s2.0-85124139465&doi=10.1016%2fj.critrevonc.2022.103622&partnerID=40&md5=7d4a847236304b897eafe217b933dfb1	wrong population		Round I
Nita, A. and Abraham, S.P. and Krejci, P. and Bosakova, M.	Oncogenic fgfr fusions produce centrosome and cilia defects by ectopic signaling	2021	Cells	https://www.scopus.com/inward/record.uri?eid=2-s2.0-85110309511&doi=10.3390%2fcells10061445&partnerID=40&md5=5b0f27da06e4fdfdeb27d3827609b5f5	wrong population		Round I
Ney, A. and Garcia-Sampedro, A. and Goodchild, G. and Acedo, P. and Fusai, G. and Pereira, S.P.	Biliary Strictures and Cholangiocarcinoma â€“ Untangling a Diagnostic Conundrum	2021	Frontiers in Oncology	https://www.scopus.com/inward/record.uri?eid=2-s2.0-85117130262&doi=10.3389%2ffonc.2021.699401&partnerID=40&md5=64b27a2842d0fee5165fafc5dfcdf576	wrong population		Round I
Hwang, J.W. and Cho, Y. and Bae, G.-U. and Kim, S.-N. and Kim, Y.K.	Protein arginine methyltransferases: promising targets for cancer therapy	2021	Experimental and Molecular Medicine	https://www.scopus.com/inward/record.uri?eid=2-s2.0-85106242160&doi=10.1038%2fs12276-021-00613-y&partnerID=40&md5=ed1441a59e2d550c30de47a42ab27e64	wrong population		Round I
Omiyale, A.O.	Adult pancreatoblastoma: Current concepts in pathology	2021	World Journal of Gastroenterology	https://www.scopus.com/inward/record.uri?eid=2-s2.0-85109887521&doi=10.3748%2fwjg.v27.i26.4172&partnerID=40&md5=f4c5540c6944706c1046b38bd0672bed	wrong population		Round I
Melisi, D. and Cavaliere, A. and Gobbo, S. and Fasoli, G. and Allegrini, V. and Simionato, F. and Gaule, M. and Casalino, S. and Pesoni, C. and Zecchetto, C. and Merz, V. and Mambrini, A. and Barbi, E. and Girelli, R. and Giardino, A. and Frigerio, I. and Scalamogna, R. and Avitabile, A. and Castellani, S. and Milella, M. and Butturini, G.	Role of next-generation genomic sequencing in targeted agents repositioning for pancreaticoduodenal cancer patients	2021	Pancreatology	https://www.scopus.com/inward/record.uri?eid=2-s2.0-85106212869&doi=10.1016%2fj.pan.2021.04.004&partnerID=40&md5=c6835df3c288f2405463b0649f43b0b2	wrong population		Round I
GonzÃ¡lez, A. and Alonso-GonzÃ¡lez, C. and GonzÃ¡lez-GonzÃ¡lez, A. and MenÃ©ndez-MenÃ©ndez, J. and Cos, S. and MartÃnez-Campa, C.	Melatonin as an adjuvant to antiangiogenic cancer treatments	2021	Cancers	https://www.scopus.com/inward/record.uri?eid=2-s2.0-85108685779&doi=10.3390%2fcancers13133263&partnerID=40&md5=d340718109eda54a22ec2f6f9dad8019	wrong population		Round I
Mandal, S. and Bandyopadhyay, S. and Tyagi, K. and Roy, A.	Recent advances in understanding the molecular role of phosphoinositide-specific phospholipase C gamma 1 as an emerging onco-driver and novel therapeutic target in human carcinogenesis	2021	Biochimica et Biophysica Acta - Reviews on Cancer	https://www.scopus.com/inward/record.uri?eid=2-s2.0-85113499733&doi=10.1016%2fj.bbcan.2021.188619&partnerID=40&md5=7ad37e927ac7db0d4bd65ebab068b203	wrong population		Round I
Kim, J.H. and Jeong, S.Y. and Jang, H.J. and Park, S.T. and Kim, H.S.	FGFR4 Gly388Arg Polymorphism Reveals a Poor Prognosis, Especially in Asian Cancer Patients: A Meta-Analysis	2021	Frontiers in Oncology	https://www.scopus.com/inward/record.uri?eid=2-s2.0-85118381271&doi=10.3389%2ffonc.2021.762528&partnerID=40&md5=05488d3678378de68835d99b26fa64be	wrong population		Round I

Fusco, M.J. and Saeed-Vafa, D. and Carballido, E.M. and Boyle, T.A. and Malafa, M. and Blue, K.L. and Teer, J.K. and Walko, C.M. and McLeod, H.L. and Kevin Hicks, J. and Extermann, M. and Fleming, J.B. and Knepper, T.C. and Kim, D.W.	Identification of targetable gene fusions and structural rearrangements to foster precision medicine in kras wild-type pancreatic cancer	2021	JCO Precision Oncology	https://www.scopus.com/in-ward/record.uri?eid=2-s2.0-85120159417&doi=10.1200%2fPO.20.00265&part-nerID=40&md5=e9892cc3d9db45769f4a5b015f3881dc	wrong population		Round I
Pfeifer, E. and Burchell, J.M. and Dazzi, F. and Sarker, D. and Beatson, R.	Apoptosis in the pancreatic cancer tumor microenvironmentâ€" the double-edged sword of cancer-associated fibroblasts	2021	Cells	https://www.scopus.com/in-ward/record.uri?eid=2-s2.0-85114078794&doi=10.3390%2fcells10071653&part-nerID=40&md5=9274f6ab75525fc7a5d6067fd1d59eb7	wrong population		Round I
Merz, V. and Gaule, M. and Zecchetto, C. and Cavaliere, A. and Casalino, S. and Pesoni, C. and Contarelli, S. and Sabbadini, F. and Bertolini, M. and Mangiameli, D. and Milella, M. and Fedele, V. and Melisi, D.	Targeting KRAS: The Elephant in the Room of Epithelial Cancers	2021	Frontiers in Oncology	https://www.scopus.com/in-ward/record.uri?eid=2-s2.0-85103075352&doi=10.3389%2ffonc.2021.638360&partne-rID=40&md5=f0031cb955acda652e721debe1339744	wrong population		Round I
Chen, K. and Zhang, Y. and Qian, L. and Wang, P.	Emerging strategies to target RAS signaling in human cancer therapy	2021	Journal of Hematology and Oncology	https://www.scopus.com/in-ward/record.uri?eid=2-s2.0-85111344286&doi=10.1186%2fs13045-021-01127-w&part-nerID=40&md5=5fb43d458073013231a150eda5b2993b	wrong population		Round I
Rupp, B. and Owen, S. and Ball, H. and Smith, K.J. and Gunchick, V. and Keller, E.T. and Sahai, V. and Nagrath, S.	Integrated Workflow for the Label-Free Isolation and Genomic Analysis of Single Circulating Tumor Cells in Pancreatic Cancer	2022	International Journal of Molecular Sciences	https://www.scopus.com/in-ward/record.uri?eid=2-s2.0-85135134171&doi=10.3390%2fijms23147852&part-nerID=40&md5=d98080008176c90c2dbaf75c31f65759	wrong population		Round I
Raja, A. and Malik, M.F.A. and Haq, F.	Genomic relevance of FGF14 and associated genes on the prognosis of pancreatic cancer	2021	PLoS ONE	https://www.scopus.com/in-ward/record.uri?eid=2-s2.0-85106997890&doi=10.1371%2fjour-nal.pone.0252344&part-nerID=40&md5=61f8926cc974256c99c907690760c6c0	wrong population		Round I
Ali, A. and Shah, A.A. and Jeang, L.J. and Fallgatter, K.S. and George, T.J. and DeRemer, D.L.	Emergence of ocular toxicities associated with novel anticancer therapeutics: What the oncologist needs to know	2022	Cancer Treatment Reviews	https://www.scopus.com/in-ward/record.uri?eid=2-s2.0-85126604400&doi=10.1016%2fj.ctrv.2022.102376&partnerID=40&md5=b40f825280001eec9a10c2d0cb7b9a12	wrong population		Round I
Yi, Y.W. and You, K.S. and Park, J.-S. and Lee, S.-G. and Seong, Y.-S.	Ribosomal protein S6: A potential therapeutic target against cancer?	2022	International Journal of Molecular Sciences	https://www.scopus.com/in-ward/record.uri?eid=2-s2.0-85122324266&doi=10.3390%2fijms23010048&part-nerID=40&md5=ca0b23bb0fb27addcd9214ec9027db83	wrong population		Round I
Dlamini, Z. and Mathabe, K. and Padayachy, L. and Marima, R. and Evangelou, G. and Syrigos, K.N. and Bianchi, A. and Lolas, G. and Hull, R.	Many voices in a choir: Tumor-induced neurogenesis and neuronal driven alternative splicing sound like suspects in tumor growth and dissemination	2021	Cancers	https://www.scopus.com/in-ward/record.uri?eid=2-s2.0-85105113114&doi=10.3390%2fcancers13092138&part-nerID=40&md5=9d9ce04e0899c86fbe2f11889e6b6821	wrong population		Round I
Yin, C. and Kulasekaran, M. and Roy, T. and Decker, B. and Alexander, S. and Margolis, M. and Jha, R.C. and Kupfer, G.M. and He, A.R.	Homologous Recombination Repair in Biliary Tract Cancers: A Prime Target for PARP Inhibition?	2022	Cancers	https://www.scopus.com/in-ward/record.uri?eid=2-s2.0-85131052066&doi=10.3390%2fcancers14102561&part-nerID=40&md5=b504e320c2cb47356fc2ad314c306e54	wrong population		Round I

Weaver, A. and Bossaer, J.B.	Fibroblast growth factor receptor (FGFR) inhibitors: A review of a novel therapeutic class	2021	Journal of Oncology Pharmacy Practice	https://www.scopus.com/inward/record.uri?eid=2-s2.0-85098511973&doi=10.1177%2f1078155220983425&partnerID=40&md5=5296e0b21eb764227a06868638c89e3d	wrong population		Round I
Tella, S.H. and Starr, J.S. and Kommalapati, A. and Sonbol, M.B. and Halfdanarson, T.R.	Management of well-differentiated neuroendocrine tumors	2021	Clinical Advances in Hematology and Oncology	https://www.scopus.com/inward/record.uri?eid=2-s2.0-85115623390&partnerID=40&md5=e72dd393f3a5e9eefdec22272dce25a8	wrong population		Round I
Chida, K. and Kawazoe, A. and Kawazu, M. and Suzuki, T. and Nakamura, Y. and Nakatsura, T. and Kuwata, T. and Ueno, T. and Kuboki, Y. and Kotani, D. and Kojima, T. and Taniguchi, H. and Mano, H. and Ikeda, M. and Shitara, K. and Endo, I. and Yoshino, T.	A low tumor mutational burden and PTEN mutations are predictors of a negative response to PD-1 blockade in MSI-H/dMMR gastrointestinal tumors	2021	Clinical Cancer Research	https://www.scopus.com/inward/record.uri?eid=2-s2.0-85109138694&doi=10.1158%2f1078-0432.CCR-21-0401&partnerID=40&md5=c8e74764a00f9b5338d6e699421fbd03	wrong population		Round I
Hofmann, M.H. and Gerlach, D. and Misale, S. and Petronczki, M. and Kraut, N.	Expanding the Reach of Precision Oncology by Drugging All KRAS Mutants	2022	Cancer Discovery	https://www.scopus.com/inward/record.uri?eid=2-s2.0-85126019577&doi=10.1158%2f2159-8290.CD-21-1331&partnerID=40&md5=bc6d4a899c911dbd5a9b31be722a7efa	wrong population		Round I
Sha, H. and Gan, Y. and Xu, F. and Zhu, Y. and Zou, R. and Peng, W. and Wu, Z. and Ma, R. and Wu, J. and Feng, J.	MicroRNA-381 in human cancer: Its involvement in tumour biology and clinical applications potential	2022	Journal of Cellular and Molecular Medicine	https://www.scopus.com/inward/record.uri?eid=2-s2.0-85122462064&doi=10.1111%2fjcmm.17161&partnerID=40&md5=a66a5ba74dae4f14bfc352c0af642b99	wrong population		Round I
Miyabayashi, K. and Nakagawa, H. and Koike, K.	Molecular and Phenotypic Profiling for Precision Medicine in Pancreatic Cancer: Current Advances and Future Perspectives	2021	Frontiers in Oncology	https://www.scopus.com/inward/record.uri?eid=2-s2.0-85109141799&doi=10.3389%2ffonc.2021.682872&partnerID=40&md5=1f09c891c460148eae368124e219f920	wrong population		Round I
SÃ¡nchez-MarÃ-n, D. and Trujano-Camacho, S. and PÃ©rez-Plasencia, C. and De LeÃ³n, D.C. and Campos-Parra, A.D.	LncRNAs driving feedback loops to boost drug resistance: sinuous pathways in cancer	2022	Cancer Letters	https://www.scopus.com/inward/record.uri?eid=2-s2.0-85132450370&doi=10.1016%2fj.canlet.2022.215763&partnerID=40&md5=97a09d61d47d8078d4808b2f0d7b3ea4	wrong population		Round I
Gimeno-Valiente, F. and LÃ³pez-Rodas, G. and Castillo, J. and Franco, L.	Alternative Splicing, Epigenetic Modifications and Cancer: A Dangerous Triangle, or a Hopeful One?	2022	Cancers	https://www.scopus.com/inward/record.uri?eid=2-s2.0-85123072135&doi=10.3390%2fcancers14030560&partnerID=40&md5=52f87efc6e126ad6bf12e53505619090	wrong population		Round I
Rodrigues, P.M. and Vogel, A. and Arrese, M. and Balderramo, D.C. and Valle, J.W. and Banales, J.M.	Next-generation biomarkers for cholangiocarcinoma	2021	Cancers	https://www.scopus.com/inward/record.uri?eid=2-s2.0-85108726496&doi=10.3390%2fcancers13133222&partnerID=40&md5=62c2b94c51b621d1e309354f59abce22	wrong population		Round I
Xelwa, N. and Candy, G.P. and Devar, J. and Omoshoro-Jones, J. and Smith, M. and Nweke, E.E.	Targeting Growth Factor Signaling Pathways in Pancreatic Cancer: Towards Inhibiting Chemoresistance	2021	Frontiers in Oncology	https://www.scopus.com/inward/record.uri?eid=2-s2.0-85108970584&doi=10.3389%2ffonc.2021.683788&partnerID=40&md5=7cc0ec6064fd80cace8aff2f2d12f530	wrong population		Round I

Luchini, C. and Grant, R.C. and Scarpa, A. and Gall-inger, S.	Microsatellite instability/mis-match repair deficiency in pancreatic cancers: The same or different?	2021	Gut	https://www.scopus.com/in-ward/record.uri?eid=2-s2.0-85099756585&doi=10.1136%2fgutjnl-2020-323805&part-nerID=40&md5=eb-dbba9d3b59173f1b59534aeb9a98a4	wrong popu-lation		Round I
Schiffmann, L.M. and Bruns, C.J. and Schmidt, T.	Resistance Mechanisms of the Metastatic Tumor Microenvi-ronment to Anti-Angiogenic Therapy	2022	Frontiers in Oncology	https://www.scopus.com/in-ward/record.uri?eid=2-s2.0-85131721223&doi=10.3389%2ffonc.2022.897927&partne-rID=40&md5=2855a281b12ab6bc9c25badc58465c5c	wrong popu-lation		Round I
de la Haba-Rodriguez, J. and Lloret, F.F. and Sal-gado, M.A.V. and Arce, M.O. and GutiÃ©rrez, A.C. and JimÃ©nez, J.G.-D. and Zambrano, C.B. and Alonso, R.M.R. and LÃ³pez, R.L. and Salas, N.R.	SEOM-GETTHI clinical guideline for the practical management of molecular platforms (2021)	2022	Clinical and Transla-tional On-cology	https://www.scopus.com/in-ward/record.uri?eid=2-s2.0-85127454971&doi=10.1007%2fs12094-022-02817-8&part-nerID=40&md5=04baef49f21571ec02e173c7812244b3	wrong popu-lation		Round I
Singhi, A.D. and Wood, L.D.	Early detection of pancreatic cancer using DNA-based mo-lecular approaches	2021	Nature Re-views Gas-troenterol-ogy and Hepatology	https://www.scopus.com/in-ward/record.uri?eid=2-s2.0-85107566684&doi=10.1038%2fs41575-021-00470-0&part-nerID=40&md5=5f0e0c1e819508424534863478b3ea94	wrong popu-lation		Round I
Ray, T. and Ryusaki, T. and Ray, P.S.	Therapeutically Targeting Cancers That Overexpress FOXC1: A Transcriptional Driver of Cell Plasticity, Par-tial EMT, and Cancer Metasta-sis	2021	Frontiers in Oncology	https://www.scopus.com/in-ward/record.uri?eid=2-s2.0-85115131644&doi=10.3389%2ffonc.2021.721959&partne-rID=40&md5=7fade9335125d0853d7b27fc23bd0423	wrong popu-lation		Round I
Raja Arul, G.L. and Fernandez-Zapico, M.E.	PI3KÎ± targeting, nipping pan-creatic cancer evolution in the bud	2021	EMBO Mo-lecular Med-icine	https://www.scopus.com/in-ward/record.uri?eid=2-s2.0-85107051335&doi=10.15252%2femmm.202114362&part-nerID=40&md5=98b525ff84dcf536ba121f3da9744856	wrong popu-lation		Round I
Hayashi, Y. and Fujita, K.	Toward urinary cell-free DNA-based treatment of urothelial carcinoma: A narra-tive review	2021	Transla-tional An-drology and Urology	https://www.scopus.com/in-ward/record.uri?eid=2-s2.0-85104502352&doi=10.21037%2ftau-20-1259&part-nerID=40&md5=0249f0e4e229787d96a6d20fe12a5d14	wrong popu-lation		Round I
PeÃ±a, V. and Kohn, T.P.	Growing evidence links male infertility and genitourinary malignancies	2020	Fertility and Sterility	https://www.scopus.com/in-ward/record.uri?eid=2-s2.0-85091523304&doi=10.1016%2fj.fertnstert.2020.08.1411&part-nerID=40&md5=29a12a49a1d4a2f84ab02b482e68424d	wrong popu-lation		Round I
Liu, W. and Zhang, Y.-Z. and Bai, S.-P.	Possible anticancer mecha-nism of oridonin determined by reverse molecular docking and network pharmacological technology	2019	Chinese Journal of New Drugs	https://www.scopus.com/in-ward/record.uri?eid=2-s2.0-85074271047&part-nerID=40&md5=aedbdb4f1896e5da5556ef5cd2881d9e	wrong popu-lation		Round I
Conway, J.R. and Herrmann, D. and Evans, T.J. and Morton, J.P. and Timpson, P.	Combating pancreatic cancer with PI3K pathway inhibitors in the era of personalised med-icine	2019	Gut	https://www.scopus.com/in-ward/record.uri?eid=2-s2.0-85056269025&doi=10.1136%2fgutjnl-2018-316822&part-nerID=40&md5=c3d429328bd7405f16045ddd0f8214db	wrong popu-lation		Round I

Cox, V.L. and Bhosale, P. and Varadachary, G.R. and Wagner-Bartak, N. and Glitza, I.C. and Gold, K.A. and Atkins, J.T. and Soliman, P.T. and Hong, D.S. and Qayyum, A.	Cancer genomics and important oncologic mutations: A contemporary guide for body imagers	2017	Radiology	https://www.scopus.com/inward/record.uri?eid=2-s2.0-85018514339&doi=10.1148%2fradiol.2017152224&partnerID=40&md5=52c821acd9f870d7cdd95e07826d58d1	wrong population		Round I
El-Behaedi, S. and Landsman, R. and Rudloff, M. and Kolyvas, E. and Albalawy, R. and Zhang, X. and Bera, T. and Collins, K. and Kozlov, S. and Alewine, C.	Protein synthesis inhibition activity of mesothelin targeting immunotoxin LMB-100 decreases concentrations of oncogenic signaling molecules and secreted growth factors	2018	Toxins	https://www.scopus.com/inward/record.uri?eid=2-s2.0-85055907037&doi=10.3390%2ftoxins10110447&partnerID=40&md5=aa59364b081a836aa910b44cf1b6deab	wrong population		Round I
Vekris, A. and Pilalis, E. and Chatziioannou, A. and Petry, K.G.	A computational pipeline for the extraction of actionable biological information from NGS-phage display experiments	2019	Frontiers in Physiology	https://www.scopus.com/inward/record.uri?eid=2-s2.0-85072996406&doi=10.3389%2ffphys.2019.01160&partnerID=40&md5=394927c5aadc19cc0fbfe2da54101fd3	wrong population		Round I
Gupta, S. and Greenberg, S. and Grimmett, J. and Gaston, D. and Agarwal, N. and Lowrance, W. and Schiffman, J. and Kohlmann, W.	Inherited DNA repair gene mutations detected by tumor next generation sequencing in urinary tract cancers	2017	Familial Cancer	https://www.scopus.com/inward/record.uri?eid=2-s2.0-85015627814&doi=10.1007%2fs10689-017-9980-2&partnerID=40&md5=ef3024ce98db6a9d65c8f98bc8b028d2	wrong population		Round I
Boyle, E.A. and Pritchard, J.K. and Greenleaf, W.J.	High-resolution mapping of cancer cell networks using co-functional interactions	2018	Molecular Systems Biology	https://www.scopus.com/inward/record.uri?eid=2-s2.0-85058823929&doi=10.15252%2fmsb.20188594&partnerID=40&md5=27806bbf0fa41849726584aa5bf60b7a	wrong population		Round I
ZschÃ¤bitz, S. and GrÃ¼llich, C.	Lenvantinib: A Tyrosine Kinase Inhibitor of VEGFR 1-3, FGFR 1-4, PDGFRÎ±, KIT and RET	2018	Recent Results in Cancer Research	https://www.scopus.com/inward/record.uri?eid=2-s2.0-85051231940&doi=10.1007%2f978-3-319-91442-8_13&partnerID=40&md5=59d3ed752f4a4d7a158f24731819ad51	wrong population		Round I
Furuse, J.	Patterns of Recurrence and Its Effective Treatment	2020	Diseases of the Gallbladder	https://www.scopus.com/inward/record.uri?eid=2-s2.0-85151192251&doi=10.1007%2f978-981-15-6010-1_23&partnerID=40&md5=425ec28da81f93f642b4d650cb292fe6	wrong population		Round I
Modica, C. and Tortarolo, D. and Comoglio, P.M. and Basilico, C. and Vigna, E.	MET/HGF co-targeting in pancreatic cancer: A tool to provide insight into the tumor/stroma crosstalk	2018	International Journal of Molecular Sciences	https://www.scopus.com/inward/record.uri?eid=2-s2.0-85058376821&doi=10.3390%2fijms19123920&partnerID=40&md5=b888714b3fcdffa6e76a8c76430774f1	wrong population		Round I
Fujinaga, H. and Sakai, Y. and Yamashita, T. and Arai, K. and Terashima, T. and Komura, T. and Seki, A. and Kawaguchi, K. and Nasti, A. and Yoshida, K. and Wada, T. and Yamamoto, K. and Kume, K. and Hasegawa, T. and Takata, T. and Honda, M. and Kaneko, S.	Biological characteristics of gene expression features in pancreatic cancer cells induced by proton and X-ray irradiation	2019	International Journal of Radiation Biology	https://www.scopus.com/inward/record.uri?eid=2-s2.0-85064007754&doi=10.1080%2f09553002.2019.1558297&partnerID=40&md5=31132d3b20bc680b17ffd8c32fbc18e2	wrong population		Round I
Angel Fastina Mary, D. and Vijayashree Priyadharshini, J.	Novel gene mutations associated with crouzon syndrome	2020	Indian Journal of Forensic Medicine and Toxicology	https://www.scopus.com/inward/record.uri?eid=2-s2.0-85099570403&doi=10.37506%2fijfmt.v14i4.12554&partnerID=40&md5=28e98734bf645053cf5e7cd1eb47521b	wrong population		Round I

Vijayvergia, N. and Dasari, A.	Targeted Therapies in the Management of Well-Differentiated Digestive and Lung Neuroendocrine Neoplasms	2020	Current Treatment Options in Oncology	https://www.scopus.com/inward/record.uri?eid=2-s2.0-85092188580&doi=10.1007%2fs11864-020-00794-0&partnerID=40&md5=21125cd46b4b4398633ced1a528bc9cd	wrong population		Round I
Bofill, X. and Cole, P. and FernÃ¡ndez-Forner, D. and TorramadÃ©, E.	NME digest	2018	Drugs of the Future	https://www.scopus.com/inward/record.uri?eid=2-s2.0-85051255048&doi=10.1358%2fdof.2018.043.07.2866118&partnerID=40&md5=2db7f6ea98613452ccf508731e4efaaf	wrong population		Round I
Ali, A.I. and Oliver, A.J. and Samiei, T. and Chan, J.D. and Kershaw, M.H. and Slaney, C.Y.	Genetic redirection of T cells for the treatment of pancreatic cancer	2019	Frontiers in Oncology	https://www.scopus.com/inward/record.uri?eid=2-s2.0-85063332433&doi=10.3389%2ffonc.2019.00056&partnerID=40&md5=131dad39de075c83cc5302eabfd5e689	wrong population		Round I
Kondo, T. and Matsubara, J. and Quy, P.N. and Fukuyama, K. and Nomura, M. and Funakoshi, T. and Doi, K. and Sakamori, Y. and Yoshioka, M. and Yokoyama, A. and Tamaoki, M. and Kou, T. and Hirohashi, K. and Yamada, A. and Yamamoto, Y. and Minamiguchi, S. and Nishigaki, M. and Yamada, T. and Kanai, M. and Matsumoto, S. and Muto, M.	Comprehensive genomic profiling for patients with chemotherapy-naÃ¯ve advanced cancer	2021	Cancer Science	https://www.scopus.com/inward/record.uri?eid=2-s2.0-85096665338&doi=10.1111%2fcas.14674&partnerID=40&md5=6dfdbb8826d134357f6b664e1a01c0ba	wrong population		Round I
Uhrig, S. and Ellermann, J. and Walther, T. and Burkhardt, P. and FrÃƒÂ¶hlich, M. and Hutter, B. and Toprak, U.H. and Neumann, O. and Stenzinger, A. and Scholl, C. and FrÃƒÂ¶hling, S. and Brors, B.	Accurate and efficient detection of gene fusions from RNA sequencing data	2021	Genome Research	https://www.scopus.com/inward/record.uri?eid=2-s2.0-85099949520&doi=10.1101%2fGR.257246.119&partnerID=40&md5=54a33a901f66c04b6b70f645ff1ca457	wrong population		Round I
Rochefort, P. and Lardy-Cleaud, A. and Sarabi, M. and Desseigne, F. and Cattey-Javouhey, A. and de la FouchardiÃ¨re, C.	Long-Term Survivors in Metastatic Pancreatic Ductal Adenocarcinoma: A Retrospective and Matched Pair Analysis	2019	Oncologist	https://www.scopus.com/inward/record.uri?eid=2-s2.0-85067038680&doi=10.1634%2ftheoncologist.2018-0786&partnerID=40&md5=1cfbb11782312f881caad4e537835174	wrong population		Round I
Du, L. and Che, Z. and Wang-Gillam, A.	Promising therapeutics of gastrointestinal cancers in clinical trials	2017	Journal of Gastrointestinal Oncology	https://www.scopus.com/inward/record.uri?eid=2-s2.0-85021166367&doi=10.21037%2fjgo.2017.01.08&partnerID=40&md5=9ed9b00564a20eaccb6fa1041dc9de4e	wrong population		Round I
Kawano, M. and Miura, T. and Fujita, M. and Koike, S. and Imadome, K. and Ishikawa, A. and Yasuda, T. and Imamura, T. and Imai, T. and Nakayama, F.	The FGF1/CPP-C chimera protein protects against intestinal adverse effects of C-ion radiotherapy without exacerbating pancreatic carcinoma	2019	Clinical and Translational Radiation Oncology	https://www.scopus.com/inward/record.uri?eid=2-s2.0-85071351496&doi=10.1016%2fj.ctro.2018.10.004&partnerID=40&md5=edaf0dc04279181b7b1dc9a7734ece08	wrong population		Round I

Basturk, O. and Berger, M.F. and Yamaguchi, H. and Adsay, V. and Askan, G. and Bhanot, U.K. and Zehir, A. and Carneiro, F. and Hong, S.-M. and Zamboni, G. and Dikoglu, E. and Jobanputra, V. and Wrzeszczynski, K.O. and Balci, S. and Allen, P. and Ikari, N. and Takeuchi, S. and Akagawa, H. and Kanno, A. and Shimosegawa, T. and Morikawa, T. and Motoi, F. and Unno, M. and Higuchi, R. and Yamamoto, M. and Shimizu, K. and Furukawa, T. and Klimstra, D.S.	Pancreatic intraductal tubulopapillary neoplasm is genetically distinct from intraductal papillary mucinous neoplasm and ductal adenocarcinoma	2017	Modern Pathology	https://www.scopus.com/inward/record.uri?eid=2-s2.0-85034240350&doi=10.1038%2fmodpathol.2017.60&partnerID=40&md5=a0fe4f103a3f639e2eb6e032c2f72d41	wrong population		Round I
Shao, S. and Qin, T. and Qian, W. and Li, X. and Li, W. and Han, L. and Zhang, D. and Wang, Z. and Ma, Q. and Wu, Z. and Wu, E. and Lei, J.	Cav-1 Ablation in Pancreatic Stellate Cells Promotes Pancreatic Cancer Growth through Nrf2-Induced shh Signaling	2020	Oxidative Medicine and Cellular Longevity	https://www.scopus.com/inward/record.uri?eid=2-s2.0-85084402640&doi=10.1155%2f2020%2f1868764&partnerID=40&md5=54c369413c9486b73c8cf3565a2e3a23	wrong population		Round I
Meleti, M. and Cassi, D. and Vescovi, P. and Setti, G. and Pertinhez, T.A. and Pezzi, M.E.	Salivary biomarkers for diagnosis of systemic diseases and malignant tumors. A systematic review	2020	Medicina Oral Patologia Oral y Cirugia Bucal	https://www.scopus.com/inward/record.uri?eid=2-s2.0-85081075184&doi=10.4317%2fmedoral.23355&partnerID=40&md5=f21d04726a007f1b887c1c6710f408c9	wrong population		Round I
Wu, G. and Li, Z. and Jiang, P. and Zhang, X. and Xu, Y. and Chen, K. and Li, X.	MicroRNA-23a promotes pancreatic cancer metastasis by targeting epithelial splicing regulator protein 1	2017	Oncotarget	https://www.scopus.com/inward/record.uri?eid=2-s2.0-85030858128&doi=10.18632%2foncotarget.20692&partnerID=40&md5=d2d8a59c9b4d27a2a057d5ff126b0bb0	wrong population		Round I
Creemers, A. and Krausz, S. and Strijker, M. and van der Wel, M.J. and Soer, E.C. and Reinten, R.J. and Besselink, M.G. and Wilmink, J.W. and van de Vijver, M.J. and van Noesel, C.J.M. and Verheij, J. and Meijer, S.L. and Dijk, F. and Bijlsma, M.F. and van Oijen, M.G.H. and van Laarhoven, H.W.M.	Clinical value of ctDNA in upper-GI cancers: A systematic review and meta-analysis	2017	Biochimica et Biophysica Acta - Reviews on Cancer	https://www.scopus.com/inward/record.uri?eid=2-s2.0-85027855506&doi=10.1016%2fj.bbcan.2017.08.002&partnerID=40&md5=322e9e8c21e9250b2b8257784aa55a84	wrong population		Round I
Nadella, S. and Burks, J. and Al-Sabban, A. and Inyang, G. and Wang, J. and Tucker, R.D. and Zamanis, M.E. and Bukowski, W. and Shivapurkar, N. and Smith, J.P.	Dietary fat stimulates pancreatic cancer growth and promotes fibrosis of the tumor microenvironment through the cholecystokinin receptor	2018	American Journal of Physiology - Gastrointestinal and Liver Physiology	https://www.scopus.com/inward/record.uri?eid=2-s2.0-85055079554&doi=10.1152%2fajpgi.00123.2018&partnerID=40&md5=03926d09535926d3a4ba03c4fb6dcc2c	wrong population		Round I
Paasinen-Sohns, A. and Koelzer, V.H. and Frank, A. and Schafroth, J. and Gisler, A. and Sachs, M. and Graber, A. and Rothschild, S.I. and Wicki, A. and Cathomas, G. and Mertz, K.D.	Single-Center Experience with a Targeted Next Generation Sequencing Assay for Assessment of Relevant Somatic Alterations in Solid Tumors	2017	Neoplasia (United States)	https://www.scopus.com/inward/record.uri?eid=2-s2.0-85015609002&doi=10.1016%2fj.neo.2017.01.003&partnerID=40&md5=bc844dda3fbc937586eeee403e6fa025	wrong population		Round I

Sunami, K. and Ichikawa, H. and Kubo, T. and Kato, M. and Fujiwara, Y. and Shimomura, A. and Koyama, T. and Kakishima, H. and Kitami, M. and Matsushita, H. and Furukawa, E. and Narushima, D. and Nagai, M. and Taniguchi, H. and Motoi, N. and Sekine, S. and Maeshima, A. and Mori, T. and Watanabe, R. and Yoshida, M. and Yoshida, A. and Yoshida, H. and Satomi, K. and Sukeda, A. and Hashimoto, T. and Shimizu, T. and Iwasa, S. and Yonemori, K. and Kato, K. and Morizane, C. and Ogawa, C. and Tanabe, N. and Sugano, K. and Hiraoka, N. and Tamura, K. and Yoshida, T. and Fujiwara, Y. and Ochiai, A. and Yamamoto, N. and Kohno, T.	Feasibility and utility of a panel testing for 114 cancer-associated genes in a clinical setting: A hospital-based study	2019	Cancer Science	https://www.scopus.com/inward/record.uri?eid=2-s2.0-85063807614&doi=10.1111%2fcas.13969&partnerID=40&md5=fb0244e71ee250669b914774a173c47d	wrong population		Round I
Casadonte, R. and Kriegsmann, M. and Perren, A. and Baretton, G. and Deininger, S.-O. and Kriegsmann, K. and Welsch, T. and Pilarsky, C. and Kriegsmann, J.	Development of a Class Prediction Model to Discriminate Pancreatic Ductal Adenocarcinoma from Pancreatic Neuroendocrine Tumor by MALDI Mass Spectrometry Imaging	2019	Proteomics - Clinical Applications	https://www.scopus.com/inward/record.uri?eid=2-s2.0-85058847492&doi=10.1002%2fprca.201800046&partnerID=40&md5=0dd97e2a49a513615d327e57c83d4663	wrong population		Round I
Maron, S.B. and Xu, J. and Janjigian, Y.Y.	Targeting EGFR in Esophagogastric Cancer	2020	Frontiers in Oncology	https://www.scopus.com/inward/record.uri?eid=2-s2.0-85098066912&doi=10.3389%2ffonc.2020.553876&partnerID=40&md5=d6bbb148b3e85c9896d79f9933d01472	wrong population		Round I
Takano, S. and Fukasawa, M. and Kadokura, M. and Shindo, H. and Takahashi, E. and Hirose, S. and Fukasawa, Y. and Kawakami, S. and Hayakawa, H. and Maekawa, S. and Mochizuki, K. and Kawaida, H. and Kono, H. and Itakura, J. and Sato, T. and Ichikawa, D. and Enomoto, N.	Mutational patterns in pancreatic juice of intraductal papillary mucinous neoplasms and concomitant pancreatic cancer	2019	Pancreas	https://www.scopus.com/inward/record.uri?eid=2-s2.0-85071339633&doi=10.1097%2fMPA.0000000000001371&partnerID=40&md5=37f0e2bb0ef7f416e3ac4faa7772694d	wrong population		Round I
Okura, R. and Fujihara, S. and Iwama, H. and Morishita, A. and Chiyo, T. and Watanabe, M. and Hirose, K. and Kobayashi, K. and Fujimori, T. and Kato, K. and Kamada, H. and Kobara, H. and Mori, H. and Niki, T. and Hirashima, M. and Okano, K. and Suzuki, Y. and Masaki, T.	MicroRNA profiles during galectin-9-induced apoptosis of pancreatic cancer cells	2018	Oncology Letters	https://www.scopus.com/inward/record.uri?eid=2-s2.0-85035326080&doi=10.3892%2fol.2017.7316&partnerID=40&md5=2549980270cece2905af3a723606724d	wrong population		Round I
Vadlamudi, Y. and Dey, D.K. and Kang, S.C.	Emerging multi-cancer regulatory role of ESRP1: Orchestration of alternative splicing to control EMT	2020	Current Cancer Drug Targets	https://www.scopus.com/inward/record.uri?eid=2-s2.0-85091779325&doi=10.2174%2f1568009620666200621153831&partnerID=40&md5=5ab647dbeed68c2cba3a5ea1ef272f34	wrong population		Round I
Risdon, E.N. and Chau, C.H. and Price, D.K. and Sartor, O. and Figg, W.D.	PARP Inhibitors and Prostate Cancer: To Infinity and Beyond BRCA	2021	Oncologist	https://www.scopus.com/inward/record.uri?eid=2-s2.0-85090468676&doi=10.1634%2ftheoncologist.2020-0697&partnerID=40&md5=1e744780e748662b78c7f6bc7c0be9fb	wrong population		Round I

El-Deiry, W.S. and Goldberg, R.M. and Lenz, H.-J. and Shields, A.F. and Gibney, G.T. and Tan, A.R. and Brown, J. and Eisenberg, B. and Heath, E.I. and Phuphanich, S. and Kim, E. and Brenner, A.J. and Marshall, J.L.	The current state of molecular testing in the treatment of patients with solid tumors, 2019	2019	CA Cancer Journal for Clinicians	https://www.scopus.com/inward/record.uri?eid=2-s2.0-85066895151&doi=10.3322%2fcaac.21560&partnerID=40&md5=8b7794a62cf545baf7f4f2b7345d6b9a	wrong population		Round I
Zhang, J. and Fu, X. and Liu, D. and Yang, M. and Yang, J. and Huo, Y. and Liu, W. and Hua, R. and Sun, Y. and Wang, J.	Molecular markers associated with perineural invasion in pancreatic ductal adenocarcinoma	2020	Oncology Letters	https://www.scopus.com/inward/record.uri?eid=2-s2.0-85089910078&doi=10.3892%2fol.2020.11866&partnerID=40&md5=7a6c786f600074622ef3b3212549a39b	wrong population		Round I
Holzmann, K. and Marian, B.	Importance of translational research for targeting fibroblast growth factor receptor signaling in cancer	2019	Cells	https://www.scopus.com/inward/record.uri?eid=2-s2.0-85089613787&doi=10.3390%2fcells8101191&partnerID=40&md5=73dc27e9ed1588bb3f3628401fd97daa	wrong population		Round I
Du, J. and Yu, Y. and Zhan, J. and Zhang, H.	Targeted therapies against growth factor signaling in breast cancer	2017	Advances in Experimental Medicine and Biology	https://www.scopus.com/inward/record.uri?eid=2-s2.0-85040607672&doi=10.1007%2f978-981-10-6020-5_6&partnerID=40&md5=5aef4e447dfd9d5fcf40d95f7a9708f0	wrong population		Round I
Beg, S. and Bareja, R. and Ohara, K. and Eng, K.W. and Wilkes, D.C. and Pisapia, D.J. and Zoughbi, W.A. and Kudman, S. and Zhang, W. and Rao, R. and Manohar, J. and Kane, T. and Sigouros, M. and Xiang, J.Z. and Khani, F. and Robinson, B.D. and Faltas, B.M. and Sternberg, C.N. and Sboner, A. and Beltran, H. and Elemento, O. and Mosquera, J.M.	Integration of whole-exome and anchored PCR-based next generation sequencing significantly increases detection of actionable alterations in precision oncology	2021	Translational Oncology	https://www.scopus.com/inward/record.uri?eid=2-s2.0-85095941705&doi=10.1016%2fj.tranon.2020.100944&partnerID=40&md5=846784999087cc2e65e80261921e3af8	wrong population		Round I
Porta, R. and Borea, R. and Coelho, A. and Khan, S. and AraÃºjo, A. and Reclusa, P. and Franchina, T. and Van Der Steen, N. and Van Dam, P. and Ferri, J. and Sirera, R. and Naing, A. and Hong, D. and Rolfo, C.	FGFR a promising druggable target in cancer: Molecular biology and new drugs	2017	Critical Reviews in Oncology/Hematology	https://www.scopus.com/inward/record.uri?eid=2-s2.0-85018527962&doi=10.1016%2fj.critrevonc.2017.02.018&partnerID=40&md5=5c8369e73740a23330acedc2ee5e66e9	wrong population		Round I
Geethadevi, A. and Parashar, D. and Bishop, E. and Pradeep, S. and Chaluvally-Raghavan, P.	ERBB signaling in CTCs of ovarian cancer and glioblastoma	2017	Genes and Cancer	https://www.scopus.com/inward/record.uri?eid=2-s2.0-85042923599&doi=10.18632%2fgenesandcancer.162&partnerID=40&md5=989389a006eb155799576bc0b055080c	wrong population		Round I
de Weger, V.A. and Goel, S. and von Moos, R. and Schellens, J.H.M. and Mach, N. and Tan, E. and Anand, S. and Scott, J.W. and Lassen, U.	A drugâ€“drug interaction study to assess the effect of the CYP1A2 inhibitor fluvoxamine on the pharmacokinetics of dovitinib (TKI258) in patients with advanced solid tumors	2018	Cancer Chemotherapy and Pharmacology	https://www.scopus.com/inward/record.uri?eid=2-s2.0-85032792586&doi=10.1007%2fs00280-017-3469-4&partnerID=40&md5=510d47d9ad6cc5a16ed2d24dce1fb0ff	wrong population		Round I
Nielsen, M. and Graversen, M. and EllebÃ¦k, S.B. and Kristensen, T.K. and Fristrup, C. and Pfeiffer, P. and Mortensen, M.B. and Detlefsen, S.	Next-generation sequencing and histological response assessment in peritoneal metastasis from pancreatic cancer treated with PIPAC	2021	Journal of Clinical Pathology	https://www.scopus.com/inward/record.uri?eid=2-s2.0-85094189735&doi=10.1136%2fjclinpath-2020-206607&partnerID=40&md5=365edbd4fa76abd646333119be31b3a7	wrong population		Round I

Shanmugam, M.K. and Warrier, S. and Kumar, A.P. and Sethi, G. and Arfuso, F.	Potential role of natural compounds as anti-angiogenic agents in cancer	2017	Current Vascular Pharmacology	https://www.scopus.com/inward/record.uri?eid=2-s2.0-85032992211&doi=10.2174%2f1570161115666170713094319&partnerID=40&md5=a2c22861104074344bf1b502f255cfb8	wrong population		Round I
Palmieri, M. and Baldassarri, M. and Fava, F. and Fabbiani, A. and Gelli, E. and Tita, R. and Torre, P. and Petrioli, R. and Hadijstilianou, T. and Galimberti, D. and Cinotti, E. and Bengala, C. and MandalÃ , M. and Piu, P. and Miano, S.T. and Martellucci, I. and Vannini, A. and Pinto, A.M. and Mencarelli, M.A. and Marsili, S. and Renieri, A. and Frullanti, E.	Two-point-NGS analysis of cancer genes in cell-free DNA of metastatic cancer patients	2020	Cancer Medicine	https://www.scopus.com/inward/record.uri?eid=2-s2.0-85078654274&doi=10.1002%2fcam4.2782&partnerID=40&md5=376681f46ba7e7dc5e951b79fde1fb31	wrong population		Round I
Li, M.-Y. and Tang, X.-H. and Fu, Y. and Wang, T.-J. and Zhu, J.-M.	Regulatory mechanisms and clinical applications of the long non-coding RNA PVT1 in cancer treatment	2019	Frontiers in Oncology	https://www.scopus.com/inward/record.uri?eid=2-s2.0-85071687473&doi=10.3389%2ffonc.2019.00787&partnerID=40&md5=4cabbaf1635d81ac85e7417e56208880	wrong population		Round I
Germann, U.A. and Furey, B.F. and Markland, W. and Hoover, R.R. and Aronov, A.M. and Roix, J.J. and Hale, M. and Boucher, D.M. and Sorrell, D.A. and Martinez-Botella, G. and Fitzgibbon, M. and Shapiro, P. and Wick, M.J. and Samadani, R. and Meshaw, K. and Groover, A. and DeCrescenzo, G. and Namchuk, M. and Emery, C.M. and Saha, S. and Welsch, D.J.	Targeting the MAPK signaling pathway in cancer: Promising preclinical activity with the novel selective ERK1/2 inhibitor BVD-523 (ulixertinib)	2017	Molecular Cancer Therapeutics	https://www.scopus.com/inward/record.uri?eid=2-s2.0-85032801567&doi=10.1158%2f1535-7163.MCT-17-0456&partnerID=40&md5=935836d7ca36e5fdbcadfadadebcd0d6	wrong population		Round I
Chandana, S.R. and Babiker, H.M. and Mahadevan, D.	Therapeutic trends in pancreatic ductal adenocarcinoma (PDAC)	2019	Expert Opinion on Investigational Drugs	https://www.scopus.com/inward/record.uri?eid=2-s2.0-85060176807&doi=10.1080%2f13543784.2019.1557145&partnerID=40&md5=92774b88f7a466b9dbc20a7b8b8b11bb	wrong population		Round I
Kou, T. and Kanai, M. and Yamamoto, Y. and Kamada, M. and Nakatsui, M. and Sakuma, T. and Mochizuki, H. and Hiroshima, A. and Sugiyama, A. and Nakamura, E. and Miyake, H. and Minamiguchi, S. and Takaori, K. and Matsumoto, S. and Haga, H. and Seno, H. and Kosugi, S. and Okuno, Y. and Muto, M.	Clinical sequencing using a next-generation sequencing-based multiplex gene assay in patients with advanced solid tumors	2017	Cancer Science	https://www.scopus.com/inward/record.uri?eid=2-s2.0-85019868410&doi=10.1111%2fcas.13265&partnerID=40&md5=4556b9bcc29a9538f840251b22b2ffa0	wrong population		Round I
Wang, F. and Li, X. and Wang, C.	Editorial: Resident and Ectopic FGF Signaling in Development and Disease	2020	Frontiers in Cell and Developmental Biology	https://www.scopus.com/inward/record.uri?eid=2-s2.0-85090788046&doi=10.3389%2ffcell.2020.00720&partnerID=40&md5=c96af56d96114ef38d3aa55d0df3b23b	wrong population		Round I
Wang, B.-C. and Wang, L.-J. and Jiang, B. and Wang, S.-Y. and Wu, N. and Li, X.-Q. and Shi, D.-Y.	Application of fluorine in drug design during 2010-2015 years: A mini-review	2017	Mini-Reviews in Medicinal Chemistry	https://www.scopus.com/inward/record.uri?eid=2-s2.0-85020524325&doi=10.2174%2f1389557515666151016124957&partnerID=40&md5=ca0b35b39e1dd30bf0e290b6b7c3b1eb	wrong population		Round I

McNamara, M.G.	Emerging facets in the treatment of patients with hepatopancreaticobiliary malignancies	2018	Current Problems in Cancer	https://www.scopus.com/inward/record.uri?eid=2-s2.0-85041621613&doi=10.1016%2fj.currproblcancer.2017.12.003&partnerID=40&md5=de98fd589650069a5244e2f2b52c34eb	wrong population		Round I
Venkatraman, S. and Meller, J. and Hongeng, S. and Tohtong, R. and Chutipongtanate, S.	Transcriptional regulation of cancer immune checkpoints: Emerging strategies for immunotherapy	2020	Vaccines	https://www.scopus.com/inward/record.uri?eid=2-s2.0-85097237822&doi=10.3390%2fvaccines8040735&partnerID=40&md5=6959df5adebd59b28bcc3a09a6c06359	wrong population		Round I
Shukuya, T. and Patel, S. and Shane-Carson, K. and He, K. and Bertino, E.M. and Shilo, K. and Otterson, G.A. and Carbone, D.P.	Lung Cancer Patients with Germline Mutations Detected by Next-Generation Sequencing and/or Liquid Biopsy	2018	Journal of Thoracic Oncology	https://www.scopus.com/inward/record.uri?eid=2-s2.0-85032946146&doi=10.1016%2fj.jtho.2017.09.1962&partnerID=40&md5=854ff69838b98432aea2944947683bb7	wrong population		Round I
Collisson, E.A.	Bringing pancreas cancer into the lab	2018	Cancer Discovery	https://www.scopus.com/inward/record.uri?eid=2-s2.0-85053216316&doi=10.1158%2f2159-8290.CD-18-0811&partnerID=40&md5=e0bfb1b3b3c2a1e4d29422f1660d74f8	wrong population		Round I
Zeng, X. and Cao, Z. and Luo, W. and Zheng, L. and Zhang, T.	MicroRNA-381â€"A Key Transcriptional Regulator: Its Biological Function and Clinical Application Prospects in Cancer	2020	Frontiers in Oncology	https://www.scopus.com/inward/record.uri?eid=2-s2.0-85097655581&doi=10.3389%2ffonc.2020.535665&partnerID=40&md5=2293a9df174b7a86db9c1d9c2db08a5f	wrong population		Round I
Fujita, Y. and Matsuda, S. and Sasaki, Y. and Masugi, Y. and Kitago, M. and Yagi, H. and Abe, Y. and Shinoda, M. and Tokino, T. and Sakamoto, M. and Kitagawa, Y.	Pathogenesis of multiple pancreatic cancers involves multicentric carcinogenesis and intrapancreatic metastasis	2020	Cancer Science	https://www.scopus.com/inward/record.uri?eid=2-s2.0-85078041760&doi=10.1111%2fcas.14268&partnerID=40&md5=a66ee23347efcb0e8762611ecd65cb1d	wrong population		Round I
Urbanski, L.M. and Leclair, N. and AnczukÃ³w, O.	Alternative-splicing defects in cancer: Splicing regulators and their downstream targets, guiding the way to novel cancer therapeutics	2018	Wiley Interdisciplinary Reviews: RNA	https://www.scopus.com/inward/record.uri?eid=2-s2.0-85045907872&doi=10.1002%2fwrna.1476&partnerID=40&md5=cff806990e1677b2935878f1aeed5bb5	wrong population		Round I
Kim, S. and Kim, K.-M. and Kim, N.K.D. and Park, J. and Ahn, S. and Yun, J.-W. and Kim, K.-T. and Park, S. and Park, P.J. and Kim, H. and Sohn, T. and Cho, D.I. and Cho, J. and Heo, J. and Kwon, W. and Lee, H. and Min, B.-H. and Hong, S.N. and Park, Y. and Lim, H. and Kang, W. and Park, W.-Y. and Lee, J.	Clinical application of targeted deep sequencing in solid-cancer patients and utility for biomarker-selected clinical trials	2017	Oncologist	https://www.scopus.com/inward/record.uri?eid=2-s2.0-85034042262&doi=10.1634%2ftheoncologist.2017-0020&partnerID=40&md5=9891f66732e6b674c63a21a28aa32dc2	wrong population		Round I
Lankadasari, M.B. and Mukhopadhyay, P. and Mohammed, S. and Harikumar, K.B.	TAMing pancreatic cancer: Combat with a double edged sword	2019	Molecular Cancer	https://www.scopus.com/inward/record.uri?eid=2-s2.0-85063730896&doi=10.1186%2fs12943-019-0966-6&partnerID=40&md5=87e51559801cb7a9e5cc4ae7f0a34dde	wrong population		Round I
Said, R. and Guibert, N. and Oxnard, G.R. and Tsimberidou, A.M.	Circulating tumor DNA analysis in the era of precision oncology	2020	Oncotarget	https://www.scopus.com/inward/record.uri?eid=2-s2.0-85078887243&doi=10.18632%2foncotarget.27418&partnerID=40&md5=143f8f7240507b5e66f92a65c2c4840f	wrong population		Round I

Yao, F. and Wang, Q. and Wu, Q.	The prognostic value and mechanisms of LNCRNA UCA1 in human cancer	2019	Cancer Management and Research	https://www.scopus.com/inward/record.uri?eid=2-s2.0-85073290625&doi=10.2147%2fCMAR.S200436&partnerID=40&md5=ee38d2d90dff0023feca61693f657fc7	wrong population			Round I
Zhou, Y. and Wu, C. and Lu, G. and Hu, Z. and Chen, Q. and Du, X.	FGF/FGFR signaling pathway involved resistance in various cancer types	2020	Journal of Cancer	https://www.scopus.com/inward/record.uri?eid=2-s2.0-85080933274&doi=10.7150%2fjca.40531&partnerID=40&md5=548962a94c97cc90486ed69832ed74f5	wrong population			Round I
Gnatenko, D.A. and Kopantsev, E.P. and Sverdlov, E.D.	Fibroblast growth factors and pancreas organogenesis	2017	Biochemistry (Moscow) Supplement Series B: Biomedical Chemistry	https://www.scopus.com/inward/record.uri?eid=2-s2.0-85037028181&doi=10.1134%2fS1990750817040023&partnerID=40&md5=fe79741411c25e51d20c8b7b742a8c20	wrong population			Round I
Biel, N.M. and Siemann, D.W.	Targeting the Angiopoietin-2/Tie-2 axis in conjunction with VEGF signal interference	2016	Cancer Letters	https://www.scopus.com/inward/record.uri?eid=2-s2.0-84923003376&doi=10.1016%2fj.canlet.2014.09.035&partnerID=40&md5=3aea925fdd5c3c85073b31a4aba0b5fb	wrong population			Round I
Sakao, S. and Tatsumi, K.	Molecular mechanisms of lung-specific toxicity induced by epidermal growth factor receptor tyrosine kinase inhibitors (Review)	2012	Oncology Letters	https://www.scopus.com/inward/record.uri?eid=2-s2.0-84866059397&doi=10.3892%2fol.2012.872&partnerID=40&md5=6ad1ed3adc4e8a091f1016ac42f6d477	wrong population			Round I
Dittmer, J. and Leyh, B.	Paracrine effects of stem cells in wound healing and cancer progression (review)	2014	International Journal of Oncology	https://www.scopus.com/inward/record.uri?eid=2-s2.0-84899508957&doi=10.3892%2fijo.2014.2385&partnerID=40&md5=974ba92d635f330f699a5daba7a160d1	wrong population			Round I
Garralda, E. and Paz, K. and LÃ³pez-Casas, P.P. and Jones, S. and Katz, A. and Kann, L.M. and LÃ³pez-Rios, F. and Sarno, F. and Al-Shahrour, F. and Vasquez, D. and Bruckheimer, E. and Angiuoli, S.V. and Calles, A. and Diaz, L.A. and Velculescu, V.E. and Valencia, A. and Sidransky, D. and Hidalgo, M.	Integrated next-generation sequencing and avatar mouse models for personalized cancer treatment	2014	Clinical Cancer Research	https://www.scopus.com/inward/record.uri?eid=2-s2.0-84899707234&doi=10.1158%2f1078-0432.CCR-13-3047&partnerID=40&md5=3c1c63681c2a437956446473739447ac	wrong population			Round I
Campbell, J. and Ryan, C.J. and Brough, R. and Bajrami, I. and Pemberton, H.N. and Chong, I.Y. and Costa-Cabral, S. and Frankum, J. and Gulati, A. and Holme, H. and Miller, R. and Postel-Vinay, S. and Rafiq, R. and Wei, W. and Williamson, C.T. and Quigley, D.A. and Tym, J. and Al-Lazikani, B. and Fenton, T. and Natrajan, R. and Strauss, S.J. and Ashworth, A. and Lord, C.J.	Large-Scale Profiling of Kinase Dependencies in Cancer Cell Lines	2016	Cell Reports	https://www.scopus.com/inward/record.uri?eid=2-s2.0-84960407495&doi=10.1016%2fj.celrep.2016.02.023&partnerID=40&md5=a511191a1fa47181d416d05d890791b9	wrong population			Round I
Radhakrishnan, P. and Grandgenett, P.M. and Mohr, A.M. and Bunt, S.K. and Yu, F. and Chowdhury, S. and Hollingsworth, M.A.	Expression of core 3 synthase in human pancreatic cancer cells suppresses tumor growth and metastasis	2013	International Journal of Cancer	https://www.scopus.com/inward/record.uri?eid=2-s2.0-84885586596&doi=10.1002%2fijc.28322&partnerID=40&md5=095fefaa7eae8c7f82247d37682c1e27	wrong population			Round I

Ronca, R. and Giacomini, A. and Di Salle, E. and Coltrini, D. and Pagano, K. and Ragona, L. and Matarazzo, S. and Rezzola, S. and Maiolo, D. and Torrella, R. and Moroni, E. and Mazzieri, R. and Escobar, G. and Mor, M. and Colombo, G. and Presta, M.	Long-Pentraxin 3 Derivative as a Small-Molecule FGF Trap for Cancer Therapy	2015	Cancer Cell	https://www.scopus.com/inward/record.uri?eid=2-s2.0-84939444543&doi=10.1016%2fj.ccell.2015.07.002&partnerID=40&md5=8af0d66ae8f4bf3880daae76cd68bf5e	wrong population		Round I
MedovÃ¡, M. and Aebersold, D.M. and Zimmer, Y.	The molecular crosstalk between the MET receptor tyrosine kinase and the DNA damage response-biological and clinical aspects	2014	Cancers	https://www.scopus.com/inward/record.uri?eid=2-s2.0-84891319812&doi=10.3390%2fcancers6010001&partnerID=40&md5=230ac9ba9ddad253a56a68b874c8bdbe	wrong population		Round I
Azizidoost, S. and Ahmadzadeh, A. and Rahim, F. and Shahjahani, M. and Seghatoleslami, M. and Saki, N.	Hepatic metastatic niche: from normal to pre-metastatic and metastatic niche	2016	Tumor Biology	https://www.scopus.com/inward/record.uri?eid=2-s2.0-84949667798&doi=10.1007%2fs13277-015-4557-x&partnerID=40&md5=bc2320dd7cc23a13404769fcb417bbc7	wrong population		Round I
Liu, Y. and Sun, J. and Zhao, M.	Literature-based knowledgebase of pancreatic cancer gene to prioritize the key genes and pathways	2016	Journal of Genetics and Genomics	https://www.scopus.com/inward/record.uri?eid=2-s2.0-84990029642&doi=10.1016%2fj.jgg.2016.04.006&partnerID=40&md5=6c1e8034cb8c99a798297a579e8d85ef	wrong population		Round I
Dienstmann, R. and Dong, F. and Borger, D. and Dias-Santagata, D. and Ellisen, L.W. and Le, L.P. and Iafrate, A.J.	Standardized decision support in next generation sequencing reports of somatic cancer variants	2014	Molecular Oncology	https://www.scopus.com/inward/record.uri?eid=2-s2.0-84903553673&doi=10.1016%2fj.molonc.2014.03.021&partnerID=40&md5=36ce7356d3fbeaffd959c42f7427a0c3	wrong population		Round I
MaryÃ¡Å¡, J. and Faktor, J. and DvoÅ™Ã¡kovÃ¡, M. and StruhÃ¡rovÃ¡, I. and Grell, P. and Bouchal, P.	Proteomics in investigation of cancer metastasis: Functional and clinical consequences and methodological challenges	2014	Proteomics	https://www.scopus.com/inward/record.uri?eid=2-s2.0-84896778783&doi=10.1002%2fpmic.201300264&partnerID=40&md5=72bd48c615a9a3b94979e00a76e02b67	wrong population		Round I
Rizvi, S.M. and Wong, J. and Saif, M.W. and Jia, Y.	Pharmacogenetics in neuroendocrine tumors of the pancreas	2014	Journal of the Pancreas	https://www.scopus.com/inward/record.uri?eid=2-s2.0-84905190062&doi=10.6092%2f1590-8577%252F2659&partnerID=40&md5=ba0b6ff250564112628992b2b134b7a9	wrong population		Round I
Bottsford-Miller, J.N. and Coleman, R.L. and Sood, A.K.	Resistance and escape from antiangiogenesis therapy: Clinical implications and future strategies	2012	Journal of Clinical Oncology	https://www.scopus.com/inward/record.uri?eid=2-s2.0-84869407359&doi=10.1200%2fJCO.2012.41.9242&partnerID=40&md5=8e06aac9bf474582f5893b58079ff529	wrong population		Round I
Tysome, J.R. and Lemoine, N.R. and Wang, Y.	Update on oncolytic viral therapy - Targeting angiogenesis	2013	OncoTargets and Therapy	https://www.scopus.com/inward/record.uri?eid=2-s2.0-84881235162&doi=10.2147%2fOTT.S46974&partnerID=40&md5=5c3b4d13a49ba3c29c703f6b11f0d16d	wrong population		Round I
Druz, A. and Chen, Y.-C. and Guha, R. and Betenbaugh, M. and Martin, S.E. and Shiloach, J.	Large-scale screening identifies a novel microRNA, miR-15a-3p, which induces apoptosis in human cancer cell lines	2013	RNA Biology	https://www.scopus.com/inward/record.uri?eid=2-s2.0-84874616942&doi=10.4161%2frna.23339&partnerID=40&md5=564fde1f39dc4fe21cf29d2e95db760d	wrong population		Round I
Kreutzer, J.N. and Salvador, A. and Diana, P. and Cirrincione, G. and Vedaldi, D. and Litchfield, D.W. and Issinger, O.-G. and Guerra, B.	2-Triazenoazaindoles: A novel class of triazenes inducing transcriptional down-regulation of EGFR and HER-2 in human pancreatic cancer cells	2012	International Journal of Oncology	https://www.scopus.com/inward/record.uri?eid=2-s2.0-84858625349&doi=10.3892%2fijo.2011.1272&partnerID=40&md5=f74f615e0cbda7899bb25701ef47eaa5	wrong population		Round I

Wagner, S.C. and Riordan, N.H. and Ichim, T.E. and Szymanski, J. and Ma, H. and Perez, J.A. and Lopez, J. and Plata-Munoz, J.J. and Silva, F. and Patel, A.N. and Kesari, S.	Safety of targeting tumor endothelial cell antigens	2016	Journal of Translational Medicine	https://www.scopus.com/inward/record.uri?eid=2-s2.0-85007524675&doi=10.1186%2fs12967-016-0842-8&partnerID=40&md5=31052f086be77383f95f2b80995b21a7	wrong population		Round I
Hammond, E. and Khurana, A. and Shridhar, V. and Dredge, K.	The role of heparanase and sulfatases in the modification of heparan sulfate proteoglycans within the tumour microenvironment and opportunities for novel cancer therapeutics	2014	Frontiers in Oncology	https://www.scopus.com/inward/record.uri?eid=2-s2.0-84904684483&doi=10.3389%2ffonc.2014.00195&partnerID=40&md5=8dd4babddef8036a5ece7f02ff982a0b	wrong population		Round I
Awasthi, N. and Schwarz, R.E.	Profile of nintedanib in the treatment of solid tumors: The evidence to date	2015	OncoTargets and Therapy	https://www.scopus.com/inward/record.uri?eid=2-s2.0-84949658578&doi=10.2147%2fOTT.S78805&partnerID=40&md5=d9282562c899a2cb9c8f7e097e65c3ad	wrong population		Round I
Yegnasubramanian, S. and Maitra, A.	Aiming for the outliers: Cancer precision medicine through targeting kinases with extreme expression	2013	Cancer Discovery	https://www.scopus.com/inward/record.uri?eid=2-s2.0-84876064910&doi=10.1158%2f2159-8290.CD-13-0016&partnerID=40&md5=7ceb91fdc523e08e171139d91616d8d8	wrong population		Round I
Daniele, G. and Corral, J. and Molife, L.R. and De Bono, J.S.	FGF Receptor inhibitors: Role in cancer therapy	2012	Current Oncology Reports	https://www.scopus.com/inward/record.uri?eid=2-s2.0-84860878985&doi=10.1007%2fs11912-012-0225-0&partnerID=40&md5=e1702a06c65e16c53c94a28aa261544a	wrong population		Round I
Szasz, A.M. and Szirtes, I. and Tihanyi, B. and Barkaszi, B. and Baranyai, Z. and Tihanyi, T. and Harsanyi, L. and Timar, J. and Kulka, J.	Basaloid carcinoma of the pancreasâ€"clinicopathological presentation and oncogenetic snapshot of a rare entity	2015	Virchows Archiv	https://www.scopus.com/inward/record.uri?eid=2-s2.0-84925483209&doi=10.1007%2fs00428-014-1662-y&partnerID=40&md5=d6f467b6e6020b0622e60fc82b74ef16	wrong population		Round I
Wen, W. and Chen, W.S. and Xiao, N. and Bender, R. and Ghazalpour, A. and Tan, Z. and Swensen, J. and Millis, S.Z. and Basu, G. and Gatalica, Z. and Press, M.F.	Mutations in the kinase domain of the HER2/ERBB2 gene identified in a wide variety of human cancers	2015	Journal of Molecular Diagnostics	https://www.scopus.com/inward/record.uri?eid=2-s2.0-84952670695&doi=10.1016%2fj.jmoldx.2015.04.003&partnerID=40&md5=6fbf32c0697d5ef76b443b6c0c1c255e	wrong population		Round I
Corn, P.G. and Wang, F. and McKeehan, W.L. and Navone, N.	Targeting fibroblast growth factor pathways in prostate cancer	2013	Clinical Cancer Research	https://www.scopus.com/inward/record.uri?eid=2-s2.0-84887047233&doi=10.1158%2f1078-0432.CCR-13-1550&partnerID=40&md5=9f4f44baea29832964d893fd57b225a1	wrong population		Round I
Wilkerson, P.M. and Reis-Filho, J.S.	the 11q13-q14 amplicon: Clinicopathological correlations and potential drivers	2013	Genes Chromosomes and Cancer	https://www.scopus.com/inward/record.uri?eid=2-s2.0-84873717467&doi=10.1002%2fgcc.22037&partnerID=40&md5=14d5172e8ede8a0bc3c04a903b31a997	wrong population		Round I
Walters, D.M. and Lindberg, J.M. and Adair, S.J. and Newhook, T.E. and Cowan, C.R. and Stokes, J.B. and Borgman, C.A. and Stelow, E.B. and Lowrey, B.T. and Chopivsky, M.E. and Gilmer, T.M. and Parsons, J.T. and Bauer, T.W.	Inhibition of the growth of patient-derived pancreatic cancer xenografts with the MEK inhibitor trametinib is augmented by combined treatment with the epidermal growth factor receptor/HER2 inhibitor lapatinib	2013	Neoplasia (United States)	https://www.scopus.com/inward/record.uri?eid=2-s2.0-84873520791&doi=10.1593%2fneo.121712&partnerID=40&md5=842aadec51e4268d8f1fb396ab9b2115	wrong population		Round I

Castro, M.M. and Galdeano, N.L. and Zaera, M.G. and Bengoechea, C.V. and Rocabert, M.T.	American Association for Cancer Research (AACR) â€“ 108th annual meeting	2017	Drugs of the Future	https://www.scopus.com/inward/record.uri?eid=2-s2.0-85026315195&doi=10.1358%2fdof.2017.042.06.2634466&partnerID=40&md5=5d562e2b19612597ff97245b56db9cf8	wrong population		Round I
Weinberg, B.A. and Yabar, C.S. and Brody, J.R. and Pishvaian, M.J.	Current standards and novel treatment options for metastatic pancreatic adenocarcinoma	2015	ONCOLOGY (United States)	https://www.scopus.com/inward/record.uri?eid=2-s2.0-84947254692&partnerID=40&md5=970769e1e134d699bd92ed88d8bbaf86	wrong population		Round I
Utsugi, T.	New challenges and inspired answers for anticancer drug discovery and development	2013	Japanese Journal of Clinical Oncology	https://www.scopus.com/inward/record.uri?eid=2-s2.0-84885101279&doi=10.1093%2fjjco%2fhyt131&partnerID=40&md5=eefc1841adcea5174eff9e6290a4c900	wrong population		Round I
Koch, C. and Trojan, J.	Established and potential predictive biomarkers in gastrointestinal cancer - C-Kit, Her2, Ras and beyond	2015	Digestion	https://www.scopus.com/inward/record.uri?eid=2-s2.0-84928656113&doi=10.1159%2f000376573&partnerID=40&md5=100edb28bfb2b72f55bf13575b834d2e	wrong population		Round I
Afratis, N. and Gialeli, C. and Nikitovic, D. and Tsegenidis, T. and Karousou, E. and Theocharis, A.D. and PavÃ£o, M.S. and Tzanakakis, G.N. and Karamanos, N.K.	Glycosaminoglycans: Key players in cancer cell biology and treatment	2012	FEBS Journal	https://www.scopus.com/inward/record.uri?eid=2-s2.0-84859102182&doi=10.1111%2fj.1742-4658.2012.08529.x&partnerID=40&md5=67eee9b4653ff742a6b2ce226ef3b929	wrong population		Round I
Strumberg, D. and Schultheis, B.	Regorafenib for cancer	2012	Expert Opinion on Investigational Drugs	https://www.scopus.com/inward/record.uri?eid=2-s2.0-84861038995&doi=10.1517%2f13543784.2012.684752&partnerID=40&md5=1c456d26a1da5bc624f9d8f8ad72d71e	wrong population		Round I
GuÃ©guinou, M. and Gambade, A. and FÃ©lix, R. and ChantÃ´me, A. and Fourbon, Y. and Bougnoux, P. and Weber, G. and Potier-Cartereau, M. and Vandier, C.	Lipid rafts, KCa/ClCa/Ca2 + channel complexes and EGFR signaling: Novel targets to reduce tumor development by lipids?	2015	Biochimica et Biophysica Acta - Biomembranes	https://www.scopus.com/inward/record.uri?eid=2-s2.0-84941241604&doi=10.1016%2fj.bbamem.2014.10.036&partnerID=40&md5=ad19e58210a80a2925a36340218e8a0d	wrong population		Round I
Huang, Y.-J. and Frazier, M.L. and Zhang, N. and Liu, Q. and Wei, C.	Reverse-phase protein array analysis to identify biomarker proteins in human pancreatic cancer	2014	Digestive Diseases and Sciences	https://www.scopus.com/inward/record.uri?eid=2-s2.0-84899921080&doi=10.1007%2fs10620-013-2938-9&partnerID=40&md5=2dbab480d63ee126a435018676db5fb5	wrong population		Round I
Lehnen, N.C. and von MÃ¤ssenhausen, A. and Kalthoff, H. and Zhou, H. and Glowka, T. and SchÃ¼tte, U. and HÃ¶ller, T. and Riesner, K. and Boehm, D. and Merkelbach-Bruse, S. and Kirfel, J. and Perner, S. and GÃ¼tgemann, I.	Fibroblast growth factor receptor 1 gene amplification in pancreatic ductal adenocarcinoma	2013	Histopathology	https://www.scopus.com/inward/record.uri?eid=2-s2.0-84880750915&doi=10.1111%2fhis.12115&partnerID=40&md5=22a4a4d78ced0722d8c336954ae5dc87	wrong population		Round I
Tan, J.	Targeting Resistance	2016	Cell	https://www.scopus.com/inward/record.uri?eid=2-s2.0-84979497798&doi=10.1016%2fj.cell.2016.07.017&partnerID=40&md5=0f03be574a26eb37076f26af53e317be	wrong population		Round I

Bonne, N.J. and Wong, D.T.W.	Salivary biomarker development using genomic, proteomic and metabolomic approaches	2012	Genome Medicine	https://www.scopus.com/inward/record.uri?eid=2-s2.0-84867895007&doi=10.1186%2fgm383&partnerID=40&md5=57968ee4c155b793d8340f1db64bdebc	wrong population		Round I
Bonavita, E. and Galdiero, M.R. and Jaillon, S. and Mantovani, A.	Phagocytes as Corrupted Policemen in Cancer-Related Inflammation	2015	Advances in Cancer Research	https://www.scopus.com/inward/record.uri?eid=2-s2.0-84952629667&doi=10.1016%2fbs.acr.2015.04.013&partnerID=40&md5=468599b573e6c2867adae56c4aa9c33e	wrong population		Round I
Lazar, D.C. and Taban, S. and Cornianu, M. and Faur, A. and Goldis, A.	New advances in targeted gastric cancer treatment	2016	World Journal of Gastroenterology	https://www.scopus.com/inward/record.uri?eid=2-s2.0-84982162734&doi=10.3748%2fwjg.v22.i30.6776&partnerID=40&md5=3c6351e99b3eae0d462ace1129a930ed	wrong population		Round I
Waddell, N. and Pajic, M. and Patch, A.-M. and Chang, D.K. and Kassahn, K.S. and Bailey, P. and Johns, A.L. and Miller, D. and Nones, K. and Quek, K. and Quinn, M.C.J. and Robertson, A.J. and Fadlullah, M.Z.H. and Bruxner, T.J.C. and Christ, A.N. and Harliwong, I. and Idrisoglu, S. and Manning, S. and Nourse, C. and Nourbakhsh, E. and Wani, S. and Wilson, P.J. and Markham, E. and Cloonan, N. and Anderson, M.J. and Fink, J.L. and Holmes, O. and Kazakoff, S.H. and Leonard, C. and Newell, F. and Poudel, B. and Song, S. and Taylor, D. and Waddell, N. and Wood, S. and Xu, Q. and Wu, J. and Pinese, M. and Cowley, M.J. and Lee, H.C. and Jones, M.D. and Nagrial, A.M. and Humphris, J. and Chantrill, L.A. and Chin, V. and Steinmann, A.M. and Mawson, A. and Humphrey, E.S. and Colvin, E.K. and Chou, A. and Scarlett, C.J. and Pinho, A.V. and Giry-Laterriere, M. and Rooman, I. and Samra, J.S. and Kench, J.G. and Pettitt, J.A. and Merrett, N.D. and Toon, C. and Epari, K. and Nguyen, N.Q. and Barbour, A. and Zeps, N. and Jamieson, N.B. and Graham, J.S. and Niclou, S.P. and Bjerkvig, R. and GrÃ¼tzmann, R. and Aust, D. and Hruban, R.H. and Maitra, A. and Iacobuzio-Donahue, C.A. and Wolfgang, C.L. and Morgan, R.A. and Lawlor, R.T. and Corbo, V. and Bassi, C. and Falconi, M. and Zamboni, G. and Tortora, G. and Tempero, M.A. and Gill, A.J. and Eshleman, J.R. and Pilarsky, C. and Scarpa, A. and Musgrove, E.A. and Pearson, J.V. and Biankin, A.V. and Grimmond, S.M.	Whole genomes redefine the mutational landscape of pancreatic cancer	2015	Nature	https://www.scopus.com/inward/record.uri?eid=2-s2.0-84924056345&doi=10.1038%2fnature14169&partnerID=40&md5=1e7177cc73bee282c01cb97a3e2f0914	wrong population		Round I

Aprile, G. and Leone, F. and Giampieri, R. and Casagrande, M. and Marino, D. and Faloppi, L. and Cascinu, S. and Fasola, G. and Scartozzi, M.	Tracking the 2015 gastrointestinal cancers symposium: Bridging cancer biology to clinical gastrointestinal oncology	2015	OncoTargets and Therapy	https://www.scopus.com/inward/record.uri?eid=2-s2.0-84930651849&doi=10.2147%2fOTT.S82624&partnerID=40&md5=f627ed04d9c674e2461ca1800b88b6b2	wrong population		Round I
Wozniak, A.	Challenges in the current anti-angiogenic treatment paradigm for patients with non-small cell lung cancer	2012	Critical Reviews in Oncology/Hematology	https://www.scopus.com/inward/record.uri?eid=2-s2.0-84859426342&doi=10.1016%2fj.critrevonc.2011.05.003&partnerID=40&md5=8c6433022708d838252364ac6b9f3ef4	wrong population		Round I
He, H. and Zhang, H. and Li, Z. and Wang, R. and Li, N. and Zhu, L.	MiRNA-214: Expression, therapeutic and diagnostic potential in cancer	2015	Tumori	https://www.scopus.com/inward/record.uri?eid=2-s2.0-84942879254&doi=10.5301%2ftj.5000318&partnerID=40&md5=c49cdc461f076b84eeaffeb942ed2157	wrong population		Round I
Hojjat-Farsangi, M.	Small-molecule inhibitors of the receptor tyrosine kinases: Promising tools for targeted cancer therapies	2014	International Journal of Molecular Sciences	https://www.scopus.com/inward/record.uri?eid=2-s2.0-84907443858&doi=10.3390%2fijms150813768&partnerID=40&md5=f72d7058db43b287ef8b07b26ed7a178	wrong population		Round I
Lee, J. and Lee, J. and Kim, S.J. and Kim, J.H.	Quercetin-3-O-glucoside suppresses pancreatic cancer cell migration induced by tumor-deteriorated growth factors in vitro	2016	Oncology Reports	https://www.scopus.com/inward/record.uri?eid=2-s2.0-84959300981&doi=10.3892%2for.2016.4598&partnerID=40&md5=8473e1604ffe5eeddd0b641ad9931658	wrong population		Round I
Ceccarelli, S. and Romano, F. and Angeloni, A. and Marchese, C.	Potential dual role of KGF/KGFR as a target option in novel therapeutic strategies for the treatment of cancers and mucosal damages	2012	Expert Opinion on Therapeutic Targets	https://www.scopus.com/inward/record.uri?eid=2-s2.0-84859590200&doi=10.1517%2f14728222.2012.671813&partnerID=40&md5=2c2b8dc2c89b81ba84a8e0f4c5237b43	wrong population		Round I
Sereno, M. and Esteban, I.R. and Zambrana, F. and Merino, M. and GÃ³mez-Raposo, C. and LÃ³pez-GÃ³mez, M. and SÃ¡enz, E.C.	Squamous-cell carcinoma of the lungs: Is it really so different?	2012	Critical Reviews in Oncology/Hematology	https://www.scopus.com/inward/record.uri?eid=2-s2.0-84870323642&doi=10.1016%2fj.critrevonc.2012.06.009&partnerID=40&md5=031b9b0b6dc12709bdce57368afd6ab6	wrong population		Round I
Jiang, C. and Wu, Y. and Xu, X. and Wang, Z.	Novel molecular targets in malignant diseases of digestive system 2014	2015	Gastroenterology Research and Practice	https://www.scopus.com/inward/record.uri?eid=2-s2.0-84929379404&doi=10.1155%2f2015%2f634740&partnerID=40&md5=39cb99dee3fdac3a5e431bed6e6f0bd4	wrong population		Round I
Berge, E.M. and Bowles, D.W. and Flaig, T.W. and Lam, E.T. and Jimeno, A.	Tivozanib: Practical implications for renal cell carcinoma and other solid tumors	2013	Drugs of Today	https://www.scopus.com/inward/record.uri?eid=2-s2.0-84878710646&doi=10.1358%2fdot.2013.49.5.1960218&partnerID=40&md5=d1377ac4d6659080e8f0737b8cd5f2b6	wrong population		Round I
Tornesello, M.L. and Buonaguro, L. and Buonaguro, F.M.	An overview of new biomolecular pathways in pathogen-related cancers	2015	Future Oncology	https://www.scopus.com/inward/record.uri?eid=2-s2.0-84930606464&doi=10.2217%2ffon.15.87&partnerID=40&md5=e21909c089340e70b4fc6f18eeb5a081	wrong population		Round I
da Silva, V.C.H. and Ramos, C.H.I.	The network interaction of the human cytosolic 90kDa heat shock protein Hsp90: A target for cancer therapeutics	2012	Journal of Proteomics	https://www.scopus.com/inward/record.uri?eid=2-s2.0-84860339862&doi=10.1016%2fj.jprot.2011.12.028&partnerID=40&md5=442c24dc5e4006d3360d78af903d0c4d	wrong population		Round I

Wood, S.L. and Brown, J.E.	The Application of 'Omics' Techniques for Cancers That Metastasise to Bone: From Biological Mechanism to Biomarkers	2014	Cancer Metastasis - Biology and Treatment	https://www.scopus.com/inward/record.uri?eid=2-s2.0-84896867249&doi=10.1007%2f978-94-007-7569-5_7&partnerID=40&md5=d9629e757d97d3853e1bdbac40f420d3	wrong population		Round I
Li, W. and Zhao, K. and Kirberger, M. and Liao, W. and Yan, Y.	Next generation sequencing technologies in cancer diagnostics and therapeutics: A mini review	2015	Cellular and Molecular Biology	https://www.scopus.com/inward/record.uri?eid=2-s2.0-84963644393&doi=10.14715%2fcmb%2f2015.61.5.16&partnerID=40&md5=c841654e838603bede134675a0d072e4	wrong population		Round I
HÃ¸gdall, D. and O'Rourke, C.J. and Taranta, A. and Oliveira, D.V.N.P. and Andersen, J.B.	Molecular Pathogenesis and Current Therapy in Intrahepatic Cholangiocarcinoma	2016	Digestive Diseases	https://www.scopus.com/inward/record.uri?eid=2-s2.0-84973577932&doi=10.1159%2f000444562&partnerID=40&md5=cfae53a313ee6b836ffbee2388dc8519	wrong population		Round I
Wagner, S.C. and Ichim, T.E. and Ma, H. and Szymanski, J. and Perez, J.A. and Lopez, J. and Bogin, V. and Patel, A.N. and Marincola, F.M. and Kesari, S.	Cancer anti-angiogenesis vaccines: Is the tumor vasculature antigenically unique?	2015	Journal of Translational Medicine	https://www.scopus.com/inward/record.uri?eid=2-s2.0-84945931147&doi=10.1186%2fs12967-015-0688-5&partnerID=40&md5=10aff088a9a7b5e0b098de43619e7d91	wrong population		Round I
Abba, M. and Mudduluru, G. and Allgayer, H.	MicroRNAs in cancer: Small molecules, big chances	2012	Anti-Cancer Agents in Medicinal Chemistry	https://www.scopus.com/inward/record.uri?eid=2-s2.0-84866396585&doi=10.2174%2f187152012802650273&partnerID=40&md5=9bf037be8902948d34c0a9b646ab90d2	wrong population		Round I
Pierce, K.J. and de Abreu, F.B. and Peterson, J.D. and Suriawinata, A.A. and Tsongalis, G.J. and Liu, X.	The genomic profile of pancreatic adenocarcinoma and its relationship to metastatic disease	2016	Experimental and Molecular Pathology	https://www.scopus.com/inward/record.uri?eid=2-s2.0-84983792605&doi=10.1016%2fj.yexmp.2016.07.011&partnerID=40&md5=943ea73f006aa0f976613dff62043a46	wrong population		Round I
Guerra, B. and Hochscherf, J. and Jensen, N.B. and Issinger, O.-G.	Identification of a novel potent, selective and cell permeable inhibitor of protein kinase CK2 from the NIH/NCI Diversity Set Library	2015	Molecular and Cellular Biochemistry	https://www.scopus.com/inward/record.uri?eid=2-s2.0-84945903781&doi=10.1007%2fs11010-015-2433-z&partnerID=40&md5=b2c3251a4ece036fcb03f00782ba390a	wrong population		Round I
Li, Y. and Tang, K. and Zhang, L. and Li, C. and Niu, F. and Zhou, W. and Yang, H. and Feng, Z. and Chen, X.	The molecular mechanisms of a novel multi-kinase inhibitor ZLJ33 in suppressing pancreatic cancer growth	2015	Cancer Letters	https://www.scopus.com/inward/record.uri?eid=2-s2.0-84919432701&doi=10.1016%2fj.canlet.2014.09.040&partnerID=40&md5=6538acd9db95fe2ceac000a6197ec948	wrong population		Round I
Akl, M.R. and Nagpal, P. and Ayoub, N.M. and Tai, B. and Prabhu, S.A. and Capac, C.M. and Gliksman, M. and Goy, A. and Suh, K.S.	Molecular and clinical significance of fibroblast growth factor 2 (FGF2/bFGF) in malignancies of solid and hematological cancers for personalized therapies	2016	Oncotarget	https://www.scopus.com/inward/record.uri?eid=2-s2.0-84978712644&doi=10.18632%2foncotarget.8203&partnerID=40&md5=59388f93f062ae23608dbfe294dabab1	wrong population		Round I
Grando, S.A.	Connections of nicotine to cancer	2014	Nature Reviews Cancer	https://www.scopus.com/inward/record.uri?eid=2-s2.0-84901604202&doi=10.1038%2fnrc3725&partnerID=40&md5=4df986cb8769d2b35ef1ee5b0638b2b1	wrong population		Round I
Estival, A. and Louvel, D. and Vaysse, N. and Clemente, F. and Couderc, B. and Prats, H. and Hollande, E.	Morphological and Biological Modifications Induced in a Rat Pancreatic Acinar Cancer Cell Line (AR4-2J) by Unscheduled Expression of Basic Fibroblast Growth Factors	1993	Cancer Research	https://www.scopus.com/inward/record.uri?eid=2-s2.0-0027523148&partnerID=40&md5=5b8dece487c6bb899b4c1756421e50ec	wrong population		Round I

Mitry, E. and Hammel, P. and Deplanque, G. and Mornex, F. and Levy, P. and Seitz, J.-F. and Moussy, A. and Kinet, J.-P. and Hermine, O. and Rougier, P. and Raymond, E.	Safety and activity of masitinib in combination with gemcitabine in patients with advanced pancreatic cancer	2010	Cancer Chemotherapy and Pharmacology	https://www.scopus.com/inward/record.uri?eid=2-s2.0-77953048032&doi=10.1007%2fs00280-010-1299-8&partnerID=40&md5=34a1db83ec3c56402f5675bd02b077a5	wrong population		Round I
Friess, H. and Kleeff, J. and Klockow, N. and Ebert, M. and Malfertheiner, P. and BÃ¼chler, M.W.	Molecular alterations in pancreatic and periampullary cancers	1999	Chirurgische Gastroenterologie mit Interdisziplinaren Gesprachen	https://www.scopus.com/inward/record.uri?eid=2-s2.0-0032773044&doi=10.1159%2f000012547&partnerID=40&md5=94653b619d77958b641bd30094f3da8d	wrong population		Round I
Bramhall, S.R.	The use of molecular technology in the differentiation of pancreatic cancer and chronic pancreatitis	1998	International Journal of Pancreatology	https://www.scopus.com/inward/record.uri?eid=2-s2.0-0031842176&doi=10.1385%2fijgc%3a23%3a2%3a83&partnerID=40&md5=a224432a9fc79c2adc9422caa014d5f2	wrong population		Round I
Koliopanos, A. and Friess, H. and Ozawa, F. and Kleeff, J. and BÃ¼chler, M.	The molecular basis of pancreatic cancer	2000	Annals of Gastroenterology	https://www.scopus.com/inward/record.uri?eid=2-s2.0-34548329304&partnerID=40&md5=261203d71d6fc2c8317bd605af43fcee	wrong population		Round I
Pour, P.M.	Growth factors and their receptors in pancreatic cancer	2001	Teratogenesis Carcinogenesis and Mutagenesis	https://www.scopus.com/inward/record.uri?eid=2-s2.0-0035177036&doi=10.1002%2f1520-6866%282001%2921%3a1%3c27%3a%3aAID-TCM4%3e3.0.CO%3b2-9&partnerID=40&md5=cc56869ead44af6fe784d2bc2cb6f89c	wrong population		Round I
Garcea, G. and Lloyd, T.D. and Gescher, A. and Dennison, A.R. and Steward, W.P. and Berry, D.P.	Angiogenesis of gastrointestinal tumours and their metastases - A target for intervention?	2004	European Journal of Cancer	https://www.scopus.com/inward/record.uri?eid=2-s2.0-2942557161&doi=10.1016%2fj.ejca.2004.02.015&partnerID=40&md5=684b02495599d0b9322dca175cfb8a66	wrong population		Round I
Humbert, M. and CastÃ©ran, N. and Letard, S. and Hanssens, K. and Iovanna, J. and Finetti, P. and Bertucci, F. and Bader, T. and Mansfield, C.D. and Moussy, A. and Hermine, O. and Dubreuil, P.	Masitinib combined with standard gemcitabine chemotherapy: In vitro and in vivo studies in human pancreatic tumour cell lines and ectopic mouse model	2010	PLoS ONE	https://www.scopus.com/inward/record.uri?eid=2-s2.0-77950128955&doi=10.1371%2fjournal.pone.0009430&partnerID=40&md5=9a65958c470c91ee8bdad42080e66fbf	wrong population		Round I
Astsaturov, I.A. and Meropol, N.J. and Alpaugh, R.K. and Burtness, B.A. and Cheng, J.D. and McLaughlin, S. and Rogatko, A. and Xu, Z. and Watson, J.C. and Weiner, L.M. and Cohen, S.J.	Phase II and coagulation cascade biomarker study of bevacizumab with or without docetaxel in patients with previously treated metastatic pancreatic adenocarcinoma	2011	American Journal of Clinical Oncology: Cancer Clinical Trials	https://www.scopus.com/inward/record.uri?eid=2-s2.0-79951678333&doi=10.1097%2fCOC.0b013e3181d2734a&partnerID=40&md5=c95c413b630dea94ec7fe655e3d3a6be	wrong population		Round I
Qin, L. and Bromberg-White, J.L. and Qian, C.-N.	Opportunities and Challenges in Tumor Angiogenesis Research. Back and Forth Between Bench and Bed	2012	Advances in Cancer Research	https://www.scopus.com/inward/record.uri?eid=2-s2.0-84858376938&doi=10.1016%2fB978-0-12-394280-7.00006-3&partnerID=40&md5=412d27b3e4c050c6e39915ce37e985ab	wrong population		Round I
Lai, J.-P. and Sandhu, D.S. and Shire, A.M. and Roberts, L.R.	The tumor suppressor function of human sulfatase 1 (SULF1) in carcinogenesis	2008	Journal of Gastrointestinal Cancer	https://www.scopus.com/inward/record.uri?eid=2-s2.0-69849092570&doi=10.1007%2fs12029-009-9058-y&partnerID=40&md5=1cfedb3e61f769e360ef79d2665de42b	wrong population		Round I

Yeang, C.-H. and McCormick, F. and Levine, A.	Combinatorial patterns of somatic gene mutations in cancer	2008	FASEB Journal	https://www.scopus.com/inward/record.uri?eid=2-s2.0-48749118242&doi=10.1096%2ffj.08-108985&partnerID=40&md5=c91614e2af96875443c556b2b9cefbf6	wrong population		Round I
Hiscox, S. and Barrett-Lee, P. and Nicholson, R.I.	Therapeutic targeting of tumor-stroma interactions	2011	Expert Opinion on Therapeutic Targets	https://www.scopus.com/inward/record.uri?eid=2-s2.0-79954455022&doi=10.1517%2f14728222.2011.561201&partnerID=40&md5=bf8c735622b8ee3088b882b74379c7d7	wrong population		Round I
Owyang, C.	Neurohormonal control of the exocrine pancreas	1994	Current Opinion in Gastroenterology	https://www.scopus.com/inward/record.uri?eid=2-s2.0-0027932280&doi=10.1097%2f00001574-199409000-00003&partnerID=40&md5=5275d73a21cdd8f8d02c7f926ca6ede7	wrong population		Round I
Braguer, D. and Barret, J.-M. and McDaid, H. and Kruczynski, A.	Antitumor Activity of Vinflunine: Effector Pathways and Potential for Synergies	2008	Seminars in Oncology	https://www.scopus.com/inward/record.uri?eid=2-s2.0-44449132575&doi=10.1053%2fj.seminoncol.2008.01.011&partnerID=40&md5=d3fa82c9b9baff6382b74d711120acaa	wrong population		Round I
Tonon, G. and Wong, K.-K. and Maulik, G. and Brennan, C. and Feng, B. and Zhang, Y. and Khatry, D.B. and Protopopov, A. and You, M.J. and Aguirre, A.J. and Martin, E.S. and Yang, Z. and Ji, H. and Chin, L. and DePinho, R.A.	High-resolution genomic profiles of human lung cancer	2005	Proceedings of the National Academy of Sciences of the United States of America	https://www.scopus.com/inward/record.uri?eid=2-s2.0-22144433675&doi=10.1073%2fpnas.0504126102&partnerID=40&md5=e320dd60c8bd07860576f07ec11e13d1	wrong population		Round I
Couch, F.J. and Wang, X. and McWilliams, R.R. and Bamlet, W.R. and De Andrade, M. and Petersen, G.M.	Association of breast cancer susceptibility variants with risk of pancreatic cancer	2009	Cancer Epidemiology Biomarkers and Prevention	https://www.scopus.com/inward/record.uri?eid=2-s2.0-72749100690&doi=10.1158%2f1055-9965.EPI-09-0306&partnerID=40&md5=40cce223dfb4945824a6751ec661b76b	wrong population		Round I
Lieu, C. and Heymach, J. and Overman, M. and Tran, H. and Kopetz, S.	Beyond VEGF: Inhibition of the fibroblast growth factor pathway and antiangiogenesis	2011	Clinical Cancer Research	https://www.scopus.com/inward/record.uri?eid=2-s2.0-80053493427&doi=10.1158%2f1078-0432.CCR-11-0659&partnerID=40&md5=96e9b78e161f972613f3200fd620c540	wrong population		Round I
Ghayad, S.E. and Cohen, P.A.	Inhibitors of the PI3K/Akt/mTOR pathway: New hope for breast cancer patients	2010	Recent Patents on Anti-Cancer Drug Discovery	https://www.scopus.com/inward/record.uri?eid=2-s2.0-77649268890&doi=10.2174%2f157489210789702208&partnerID=40&md5=f0661fbb30a8db17e7734f86d6a1ab6f	wrong population		Round I
Peduto, L.	ADAM9 as a potential target molecule in cancer	2009	Current Pharmaceutical Design	https://www.scopus.com/inward/record.uri?eid=2-s2.0-69249117009&doi=10.2174%2f138161209788682415&partnerID=40&md5=6cd2d3606221b012875d06ca030d821e	wrong population		Round I

Thomas, R.K. and Baker, A.C. and DeBiasi, R.M. and Winckler, W. and LaFramboise, T. and Lin, W.M. and Wang, M. and Feng, W. and Zander, T. and MacConnaill, L.E. and Lee, J.C. and Nicoletti, R. and Hatton, C. and Goyette, M. and Girard, L. and Majmudar, K. and Ziaugra, L. and Wong, K.-K. and Gabriel, S. and Beroukhim, R. and Peyton, M. and Barretina, J. and Dutt, A. and Emery, C. and Greulich, H. and Shah, K. and Sasaki, H. and Gazdar, A. and Minna, J. and Armstrong, S.A. and Mellinghoff, I.K. and Hodi, F.S. and Dranoff, G. and Mischel, P.S. and Cloughesy, T.F. and Nelson, S.F. and Liau, L.M. and Mertz, K. and Rubin, M.A. and Moch, H. and Loda, M. and Catalona, W. and Fletcher, J. and Signoretti, S. and Kaye, F. and Anderson, K.C. and Demetri, G.D. and Dummer, R. and Wagner, S. and Herlyn, M. and Sellers, W.R. and Meyerson, M. and Garraway, L.A.	High-throughput oncogene mutation profiling in human cancer	2007	Nature Genetics	https://www.scopus.com/inward/record.uri?eid=2-s2.0-33847293670&doi=10.1038%2fng1975&partnerID=40&md5=ca085ba2ae04c047aa86eb241cfe9595	wrong population		Round I
Chen, T. and Georgea, J.A. and Taylor, C.C.	Src tyrosine kinase as a chemotherapeutic target: Is there a clinical case?	2006	Anti-Cancer Drugs	https://www.scopus.com/inward/record.uri?eid=2-s2.0-33646232229&doi=10.1097%2f00001813-200602000-00002&partnerID=40&md5=0b216dcf3d3cb6a217855fd19a15a57d	wrong population		Round I
Santarius, T. and Shipley, J. and Brewer, D. and Stratton, M.R. and Cooper, C.S.	A census of amplified and overexpressed human cancer genes	2010	Nature Reviews Cancer	https://www.scopus.com/inward/record.uri?eid=2-s2.0-72949103929&doi=10.1038%2fnrc2771&partnerID=40&md5=82b48c5b30dfcac6662393c4d303d06c	wrong population		Round I
Pollard, C. and Smith, S.C. and Theodorescu, D.	Molecular genesis of non-muscle-invasive urothelial carcinoma (NMIUC)	2010	Expert Reviews in Molecular Medicine	https://www.scopus.com/inward/record.uri?eid=2-s2.0-77953169090&doi=10.1017%2fS1462399410001407&partnerID=40&md5=ac7073657faf6df201c2f7e8e658188c	wrong population		Round I
Liao, D. and Johnson, R.S.	Hypoxia: A key regulator of angiogenesis in cancer	2007	Cancer and Metastasis Reviews	https://www.scopus.com/inward/record.uri?eid=2-s2.0-34547124062&doi=10.1007%2fs10555-007-9066-y&partnerID=40&md5=12e79845671b71966f9af4715202365c	wrong population		Round I
NOT REPORTED	Molecular biology of pancreatic cancer: overexpression of fibroblast growth factors	1994	Der Chirurg		wrong population		Round I
Parker, R.B. and Kohler, J.J.	Regulation of intracellular signaling by extracellular glycan remodeling	2010	ACS Chemical Biology	https://www.scopus.com/inward/record.uri?eid=2-s2.0-75749084864&doi=10.1021%2fcb9002514&partnerID=40&md5=d6393033e13a51a9cb7052c2e574387c	wrong population		Round I

Marrari, A. and George, S.	Broad-spectrum kinase inhibitor oncolytic	2011	Drugs of the Future	https://www.scopus.com/inward/record.uri?eid=2-s2.0-84952979890&doi=10.1358%2fdof.2011.036.01.1568343&partnerID=40&md5=e7f5b1c7b835a0b6718d553a3c8a846f	wrong population		Round I
Hasskarl, J.	Sorafenib	2010	Recent Results in Cancer Research	https://www.scopus.com/inward/record.uri?eid=2-s2.0-77953188742&doi=10.1007%2f978-3-642-01222-8_5&partnerID=40&md5=6ed9acb47d3e146b95e6fd8cfd362014	wrong population		Round I
JÃ¤nne, P.A. and Gray, N. and Settleman, J.	Factors underlying sensitivity of cancers to small-molecule kinase inhibitors	2009	Nature Reviews Drug Discovery	https://www.scopus.com/inward/record.uri?eid=2-s2.0-69949151386&doi=10.1038%2fnrd2871&partnerID=40&md5=ac66f3c809e2017df7ff4ff392e518d5	wrong population		Round I
Teillet, F. and Boumendjel, A. and Boutonnat, J. and Ronot, X.	Flavonoids as RTK inhibitors and potential anticancer agents	2008	Medicinal Research Reviews	https://www.scopus.com/inward/record.uri?eid=2-s2.0-50249085093&doi=10.1002%2fmed.20122&partnerID=40&md5=cfd7059565c110d08db9ad369614ce2f	wrong population		Round I
You, W.-K. and Sennino, B. and Williamson, C.W. and FalcÃ³n, B. and Hashizume, H. and Yao, L.-C. and Aftab, D.T. and McDonald, D.M.	VEGF and c-Met blockade amplify angiogenesis inhibition in Pancreatic Islet Cancer	2011	Cancer Research	https://www.scopus.com/inward/record.uri?eid=2-s2.0-79960408937&doi=10.1158%2f0008-5472.CAN-10-2527&partnerID=40&md5=0a1a1faed8ee2dbd33a34950fe616e85	wrong population		Round I
Jiang, H. and Conrad, C. and Fueyo, J. and Gomez-Manzano, C. and Liu, T.-J.	Oncolytic adenoviruses for malignant glioma therapy	2003	Frontiers in Bioscience	https://www.scopus.com/inward/record.uri?eid=2-s2.0-3242686373&partnerID=40&md5=40f17791eef16da7f8cc9b1d47f2af44	wrong population		Round I
Okunieff, P.	Mrna expression of fibroblast growth factors and receptors in human colon adenocarcinomas	1996	Journal of Investigative Medicine	https://www.scopus.com/inward/record.uri?eid=2-s2.0-33749442349&partnerID=40&md5=2dfd1bffd41ce8020149e71b0f574ca2	wrong population		Round I
Kornmann, M. and Beger, H.G. and Korc, M.	Role of fibroblast growth factors and their receptors in pancreatic cancer and chronic pancreatitis	1998	Pancreas	https://www.scopus.com/inward/record.uri?eid=2-s2.0-0031844173&doi=10.1097%2f00006676-199808000-00010&partnerID=40&md5=6364a05f8173d8c0e2f8d4274b47e9c0	wrong population		Round I
Dangle, P.P. and Zaharieva, B. and Jia, H. and Pohar, K.S.	Ras-MAPK pathway as a therapeutic target in cancer - Emphasis on bladder cancer	2009	Recent Patents on Anti-Cancer Drug Discovery	https://www.scopus.com/inward/record.uri?eid=2-s2.0-68949103943&doi=10.2174%2f157489209788452812&partnerID=40&md5=40942ff8b2a862db423e84f743eaaaa2	wrong population		Round I
BolÃ³s, V. and Gasent, J.M. and LÃ³pez-Tarruella, S. and Grande, E.	The dual kinase complex FAK-src as a promising therapeutic target in cancer	2010	OncoTargets and Therapy	https://www.scopus.com/inward/record.uri?eid=2-s2.0-79955727135&doi=10.2147%2fott.s6909&partnerID=40&md5=95fdd0f68796422802833745f1a8ecea	wrong population		Round I
McNeil, B.K. and Getzenberg, R.H.	Urinary protein biomarkers of cancer	2009	Expert Opinion on Medical Diagnostics	https://www.scopus.com/inward/record.uri?eid=2-s2.0-77953452982&doi=10.1517%2f17530050902824811&partnerID=40&md5=2eb071415a2763aa002de38e3471d459	wrong population		Round I

Hosking, F.J. and Dobbins, S.E. and Houlston, R.S.	Genome-wide association studies for detecting cancer susceptibility	2011	British Medical Bulletin	https://www.scopus.com/inward/record.uri?eid=2-s2.0-79952540140&doi=10.1093%2fbmb%2fldq038&partnerID=40&md5=2ea24b5fcdd1af1957dba9aac3f96fad	wrong population		Round I
Friess, H. and Kleeff, J. and Berberat, P. and BÃ¼chler, M.W.	Molecular biology and oncogenesis of pancreatic cancer	1997	Chirurgische Gastroenterologie mit Interdisziplinaren Gesprachen	https://www.scopus.com/inward/record.uri?eid=2-s2.0-0031045172&doi=10.1159%2f000190115&partnerID=40&md5=f17e5a43594f1bfe494d2acf31b27039	wrong population		Round I
Matejuk, A. and Leng, Q. and Chou, S.-T. and Mixson, A.J.	Vaccines targeting the neovasculature of tumors	2011	Vascular Cell	https://www.scopus.com/inward/record.uri?eid=2-s2.0-79955641941&doi=10.1186%2f2045-824X-3-7&partnerID=40&md5=cb004c372adad9ed6f06e89e34da3ba9	wrong population		Round I
Williams, J.A.	Receptor-mediated signal transduction pathways and the regulation of pancreatic acinar cell function	2008	Current Opinion in Gastroenterology	https://www.scopus.com/inward/record.uri?eid=2-s2.0-55549114708&doi=10.1097%2fMOG.0b013e32830b110c&partnerID=40&md5=42bf53307b57c030cca76add2e84d19b	wrong population		Round I
Katoh, Y. and Katoh, M.	FGF signaling inhibitor, SPRY4, is evolutionarily conserved target of WNT signaling pathway in progenitor cells	2006	International Journal of Molecular Medicine	https://www.scopus.com/inward/record.uri?eid=2-s2.0-33644936022&doi=10.3892%2fijmm.17.3.529&partnerID=40&md5=2f5d01ae24be10563c66c0921f117486	wrong population		Round I
LaConti, J.J. and Shivapurkar, N. and Preet, A. and Deslattes Mays, A. and Peran, I. and Kim, S.E. and Marshall, J.L. and Riegel, A.T. and Wellstein, A.	Tissue and serum microRNAs in the KrasG12D transgenic animal model and in patients with pancreatic cancer	2011	PLoS ONE	https://www.scopus.com/inward/record.uri?eid=2-s2.0-79959588396&doi=10.1371%2fjournal.pone.0020687&partnerID=40&md5=f17f1da3aaf16384c9d413ab762e388f	wrong population		Round I
Banti, I. and Nencetti, S. and Orlandini, E. and Lapucci, A. and Breschi, M.C. and Fogli, S.	Synthesis and in-vitro antitumour activity of new naphthyridine derivatives on human pancreatic cancer cells	2009	Journal of Pharmacy and Pharmacology	https://www.scopus.com/inward/record.uri?eid=2-s2.0-85047684386&doi=10.1211%2fjpp.61.08.0010&partnerID=40&md5=92800e7fb4c87b62bc7ad973a840d263	wrong population		Round I
Higashiyama, S. and Iwabuki, H. and Morimoto, C. and Hieda, M. and Inoue, H. and Matsushita, N.	Membrane-anchored growth factors, the epidermal growth factor family: Beyond receptor ligands	2008	Cancer Science	https://www.scopus.com/inward/record.uri?eid=2-s2.0-38949204418&doi=10.1111%2fj.1349-7006.2007.00676.x&partnerID=40&md5=4bf1372ef6e70f76a774ab7203bcdd6f	wrong population		Round I
Cavallaro, U. and Niedermeyer, J. and Fuxa, M. and Christofori, G.	N-CAM modulates tumour-cell adhesion to matrix by inducing FGF-receptor signalling	2001	Nature Cell Biology	https://www.scopus.com/inward/record.uri?eid=2-s2.0-0034945537&doi=10.1038%2f35083041&partnerID=40&md5=9a93a49cb8f3a20c68b397330603eed2	wrong population		Round I
Corbo, V. and Ritelli, R. and Barbi, S. and Funel, N. and Campani, D. and Bardelli, A. and Scarpal, A.	Mutational profiling of kinases in human tumours of pancreatic origin identifies candidate cancer genes in ductal and ampulla of vater carcinomas	2010	PLoS ONE	https://www.scopus.com/inward/record.uri?eid=2-s2.0-77958603990&doi=10.1371%2fjournal.pone.0012653&partnerID=40&md5=ae52853eeae192ed7ee49ee4cde87f3f	wrong population		Round I
Kneller, S.	American association for cancer research 98th annual meeting. Anticancer agents - Part I	2007	IDrugs	https://www.scopus.com/inward/record.uri?eid=2-s2.0-34250614003&partnerID=40&md5=4d805c2af5319ce783c61f4509c3178a	wrong population		Round I

Stokes, J.B. and Adair, S.J. and Slack-Davis, J.K. and Walters, D.M. and Tilghman, R.W. and Hershey, E.D. and Lowrey, B. and Thomas, K.S. and Bouton, A.H. and Hwang, R.F. and Stelow, E.B. and Parsons, J.T. and Bauer, T.W.	Inhibition of focal adhesion kinase by PF-562,271 inhibits the growth and metastasis of pancreatic cancer concomitant with altering the tumor microenvironment	2011	Molecular Cancer Therapeutics	https://www.scopus.com/inward/record.uri?eid=2-s2.0-81055127013&doi=10.1158%2f1535-7163.MCT-11-0261&partnerID=40&md5=13ce385200d34d6b60ca1195ada462b7	wrong population		Round I
Torrisani, J. and Buscail, L.	Molecular pathways of pancreatic carcinogenesis	2002	Annales de Pathologie	https://www.scopus.com/inward/record.uri?eid=2-s2.0-0036812597&partnerID=40&md5=898d63432da96ac08a365579c126810f	wrong population		Round I
Zachos, I. and Tzortzis, V. and Konstantinopoulos, P.A. and Karatzas, A. and Gravas, S. and Melekos, M. and Papavassiliou, A.G.	Molecular pathogenesis of non muscle-invasive bladder cancer: Implications for novel targeted therapies	2011	Current Molecular Medicine	https://www.scopus.com/inward/record.uri?eid=2-s2.0-80053937661&doi=10.2174%2f156652411797536697&partnerID=40&md5=22f0595359ebc7c4d2e21c50a9281833	wrong population		Round I
Ammerpohl, O. and Kalthoff, H.	The role of protein kinases in pancreatic carcinogenesis	2007	Clinica Chimica Acta	https://www.scopus.com/inward/record.uri?eid=2-s2.0-34248201080&doi=10.1016%2fj.cca.2007.02.024&partnerID=40&md5=a895f731173fdf28680ea6d0dfc5af28	wrong population		Round I
Joshi, S. and Tiwari, A.K. and Mondal, B. and Sharma, A.	Oncoproteomics	2011	Clinica Chimica Acta	https://www.scopus.com/inward/record.uri?eid=2-s2.0-78650234795&doi=10.1016%2fj.cca.2010.10.002&partnerID=40&md5=46ccd16907039d993f2c0f1f1d647e92	wrong population		Round I
Morandi, A. and Plaza-Menacho, I. and Isacke, C.M.	RET in breast cancer: Functional and therapeutic implications	2011	Trends in Molecular Medicine	https://www.scopus.com/inward/record.uri?eid=2-s2.0-79952444251&doi=10.1016%2fj.molmed.2010.12.007&partnerID=40&md5=7455b51df5463141692296ba7775195f	wrong population		Round I
Mie, T. and Sasaki, T. and Okamoto, T. and Furukawa, T. and Takeda, T. and Kasuga, A. and Ozaka, M. and Sasahira, N.	Current Status of Targeted Therapy for Biliary Tract Cancer in the Era of Precision Medicine	2024	Cancers	https://www.embase.com/search/results?subaction=viewrecord&id=L2028955632&from=export U2 - L2028955632	wrong population	10.3390/cancers16050879	Round I
Letissier, O. and Smolenschi, C. and Hollebecque, A. and Vasseur, D. and Champiat, S. and Bahleda, R. and Gazzah, A. and Michot, J.-M. and Danlos, F.-X. and Ouali, K. and Henon, C. and Parisi, C. and Mahjoubi, L. and Goldschmidt, V. and Loriot, Y. and Massard, C. and Bayle, A. and Italiano, A. and Baldini, C. and Ponce, S.A.	The use of liquid biopsy in patients with advanced pancreatic cancer (PDAC) to guide enrollment in phase I clinical trials	2024	Cancer Res.	https://www.embase.com/search/results?subaction=viewrecord&id=L643357920&from=export U2 - L643357920	wrong population		Round I
Eslinger, C. and Yee, C. and Seddighzadeh, B. and Elsabbagh, Z. and Pai, R. and Hartley, C.P. and Bekaii-Saab, T.S. and Starr, J.S. and Halfdanarson, T.R. and Sonbol, B.B.	Clinical outcomes and molecular characteristics of patients with pancreatic acinar cell carcinoma	2024	J. Clin. Oncol.	https://www.embase.com/search/results?subaction=viewrecord&id=L643533883&from=export U2 - L643533883	wrong population		Round I

Furuse, J. and Jiang, B. and Kuwahara, T. and Satoh, T. and Ma, X. and Yan, S. and Zhao, H.-T. and Ikeda, M. and Cui, T. and Sasaki, T. and Meng, Z. and Nakai, Y. and Ueno, M. and Komatsu, Y. and Nagano, H. and Morizane, C. and Funasaka, S. and Ikezawa, H. and Nakada, T. and Shen, L.	Pivotal single-arm, phase 2 trial of tasurgratinib for patients with fibroblast growth factor receptor (FGFR)-2 gene fusion-positive cholangiocarcinoma (CCA)	2024	J. Clin. Oncol.	https://www.embase.com/search/results?subaction=viewrecord&id=L643534075&from=export U2 - L643534075	wrong population		Round I
Gao, Z. and Houthuijzen, J.M. and ten Dijke, P. and Brazil, D.P.	GREM1 signaling in cancer: tumor promotor and suppressor?	2023	J. Cell Commun. Signal.	https://www.embase.com/search/results?subaction=viewrecord&id=L2025059516&from=export U2 - L2025059516	wrong outcome	10.1007/s12079-023-00777-4	Round I
Metekohy, C. and Pisini, M. and Janssen, E. and Fernandez, G. and Michaels-Igbokwe, C.	PCR182 Patient Preferences for Tumor-Agnostic Therapies: Qualitative Insights and Challenges	2023	Value Health	https://www.embase.com/search/results?subaction=viewrecord&id=L2029285516&from=export U2 - L2029285516	wrong population		Round I
Aoki, Y. and Inoue, Y. and Sasahira, N. and Ono, M. and Inamura, K. and Kataoka, A. and Takano, T. and Kanao, H. and Watanabe, M.	Primary ovarian insufficiency associated with lenvatinib therapy in a patient with hepatocellular carcinoma: A case report	2023	Oncol. Lett.	https://www.embase.com/search/results?subaction=viewrecord&id=L2027064524&from=export U2 - L2027064524	wrong population	10.3892/ol.2023.14037	Round I
Javle, M. and Kankeu Fonkoua, L.A. and Mahipal, A. and Liao, C.-Y. and Fountzilas, C. and Li, D. and Pelster, M.S. and Goel, S. and Peng, P. and Sun, C. and Wang, H. and Hennessy, K. and Fu, X. and Neo, L. and Shan, Y. and Huang, P. and Wu, D. and Wu, F. and Fan, J. and Piha-Paul, S.A.	95MO Tinengotinib in patients with advanced, fibroblast growth factor receptor (FGFR) inhibitor refractory/relapsed cholangiocarcinoma	2023	Ann. Oncol.	https://www.embase.com/search/results?subaction=viewrecord&id=L2027892084&from=export U2 - L2027892084	wrong population		Round I
Andersen, K. and Brusgaard, K. and Detlefsen, S. and Christesen, H.	Two new candidate genes, OGDH and FGFR1 discovered in an insulinoma from a fifteen-year-old male	2023	Horm. Res. Paediatr.	https://www.embase.com/search/results?subaction=viewrecord&id=L643129674&from=export U2 - L643129674	wrong population	10.1159/000533803	Round I
Carotenuto, P. and Gradilone, S.A. and Franco, B.	Cilia and Cancer: From Molecular Genetics to Therapeutic Strategies	2023	Genes	https://www.embase.com/search/results?subaction=viewrecord&id=L2024745420&from=export U2 - L2024745420	wrong publication type	10.3390/genes14071428	Round I
Li, Y. and Shi, Y. and Zhang, X. and Li, P. and Ma, L. and Hu, P. and Xu, L. and Dai, Y. and Xia, S. and Qiu, H.	FGFR2 upregulates PAI-1 via JAK2/STAT3 signaling to induce M2 polarization of macrophages in colorectal cancer	2023	Biochim. Biophys. Acta Mol. Basis Dis.	https://www.embase.com/search/results?subaction=viewrecord&id=L2022792526&from=export U2 - L2022792526	wrong population	10.1016/j.bbadis.2023.166665	Round I
Bigot, L. and Nobre, C. and Facchinetti, F. and Poiraudeau, L. and Braye, F. and Sabio, J. and Mensourri, N. and Deas, O. and Nicotra, C. and Ngo-Camus, M. and Tselikas, L. and Scoazec, J.Y. and Fizazi, K. and Ponce, S. and Besse, B. and Friboulet, L. and Loriot, Y.	MatchR a preclinical platform of models resistant to innovative therapies	2023	Cancer Res.	https://www.embase.com/search/results?subaction=viewrecord&id=L641473276&from=export U2 - L641473276	wrong population		Round I

Shi, G.-M. and Huang, X.-Y. and Wen, T.-F. and Song, T.-Q. and Kuang, M. and Mou, H.-B. and Bao, L.-Q. and Zhao, H.-T. and Zhao, H. and Feng, X.-L. and Zhang, B.-X. and Peng, T. and Zhang, Y.-B. and Li, X.-C. and Yu, H.-S. and Cao, Y. and Liu, L.-X. and Zhang, T. and Wang, W.-L. and Ran, J.-H. and Liu, Y.-B. and Gong, W. and Chen, M.-X. and Cao, L. and Luo, Y. and Wang, Y. and Zhou, H. and Yang, G.-H. and Fan, J. and Zhou, J.	Pemigatinib in previously treated Chinese patients with locally advanced or metastatic cholangiocarcinoma carrying FGFR2 fusions or rearrangements: A phase II study	2023	Cancer Med.	https://www.embase.com/search/results?subaction=viewrecord&id=L2019160267&from=export U2 - L2019160267	wrong population		Round I
Mughal, M.J. and Bhadresha, K. and Kwok, H.F.	CDK inhibitors from past to present: A new wave of cancer therapy	2023	Semin. Cancer Biol.	https://www.embase.com/search/results?subaction=viewrecord&id=L2022016350&from=export U2 - L2022016350	wrong drug	10.1016/j.semcancer.2022.12.006	Round I
da Silva, F.J. and Carvalho de Azevedo, J. and Ralph, A.C.L. and Pinheiro, J.D.J.V. and Freitas, V.M. and Calcagno, D.Q.	Salivary glands adenoid cystic carcinoma: a molecular profile update and potential implications	2023	Front. Oncol.	https://www.embase.com/search/results?subaction=viewrecord&id=L2024515441&from=export U2 - L2024515441	wrong population	10.3389/fonc.2023.1191218	Round I
Hou, N. and Cui, Y.	Novel tyrosine kinase inhibitor sulfatinib for the treatment of neuroendocrine neoplasms	2023	Pharm. Educ.	https://www.embase.com/search/results?subaction=viewrecord&id=L642266137&from=export U2 - L642266137	wrong population	10.46542/pe.2023.233.151155	Round I
Yang, J. and Zhu, Q. and Wu, Y. and Qu, X. and Liu, H. and Jiang, B. and Ge, D. and Song, X.	Utilization of macrocyclic peptides to target protein-protein interactions in cancer	2022	Front. Oncol.	https://www.embase.com/search/results?subaction=viewrecord&id=L2020390216&from=export U2 - L2020390216	wrong drug	10.3389/fonc.2022.992171	Round I
Tanaka, S. and Umemoto, K. and Kubo, S. and Sato, Y. and Mimaki, S. and Tsuchihara, K. and Takemura, S. and Shinkawa, H. and Mori, A. and Ikeda, M.	Nivolumab for treating patients with occupational cholangiocarcinoma	2022	J. Hepato-Biliary-Pancreatic Sci.	https://www.embase.com/search/results?subaction=viewrecord&id=L2018456857&from=export U2 - L2018456857	wrong drug	10.1002/jhbp.1215	Round I
Narayan, V. and Jonasch, E.	Systemic Therapy Development in Von Hippelâ€"Lindau Disease: An Outsized Contribution from an Orphan Disease	2022	Cancers	https://www.embase.com/search/results?subaction=viewrecord&id=L2020041027&from=export U2 - L2020041027	wrong drug	10.3390/cancers14215313	Round I
Heinrich, K. and Miller-Phillips, L. and Ziemann, F. and Hasselmann, K. and RÃ¼hlmann, K. and Von Bergwelt-Baildon, M. and Holch, J. and Herold, T. and Von Baumgarten, L. and Greif, P. and Jeremias, I. and Trillsch, F. and WÃ¼rstlein, R. and Jung, A. and Kirchner, T. and Klauschen, F. and Metzeler, K. and Heinemann, V. and Westphalen, B.	The CCCMLMU Molecular Tumorboard: clinical and molecular characteristics of the first 1000 patients	2022	Oncol. Res. Treat.	https://www.embase.com/search/results?subaction=viewrecord&id=L640065244&from=export U2 - L640065244	wrong population		Round I
Angre, T. and Kumar, A. and Singh, A.K. and Thareja, S. and Kumar, P.	Role of Collagen Regulators in Cancer Treatment: A Comprehensive Review	2022	Anti-Cancer Agents Med. Chem.	https://www.embase.com/search/results?subaction=viewrecord&id=L2017768751&from=export U2 - L2017768751	wrong drug	10.2174/187152062666220501162351	Round I

Knepper, T.C. and Kim, D.W. and Mauer, E. and Ronski, K. and Gulhati, P.	Comparative analysis of the targetable landscape in KRAS-mutant and wild-type pancreatic adenocarcinoma	2022	J. Clin. Oncol.	https://www.embase.com/search/results?subaction=viewrecord&id=L638836205&from=export U2 - L638836205	wrong population		Round I
Gosse, M. and Sompallae, R. and Guseva, N. and Snow, A. and Bossler, A. and Ma, D.	Genomic Profiling of Pancreatic Ductal Adenocarcinoma, Intrahepatic and Extrahepatic Cholangiocarcinoma	2022	Mod. Pathol.	https://www.embase.com/search/results?subaction=viewrecord&id=L638006474&from=export U2 - L638006474	wrong population		Round I
Javle, M.M. and Abou-Alfa, G.K. and Macarulla, T. and Personeni, N. and Adeva, J. and Bergamo, F. and Malka, D. and Vogel, A. and Knox, J.J. and Evans, T.R.J. and Dimova-Dobreva, M. and Harris, W.P. and Saulay, M. and Engelhardt, M. and Braun, S. and Dit Busset, M.D. and Borad, M.J.	Efficacy of derazantinib in intrahepatic cholangiocarcinoma patients with FGFR2 mutations or amplifications: Interim results from the phase 2 study FIDES-01	2022	J. Clin. Oncol.	https://www.embase.com/search/results?subaction=viewrecord&id=L637296716&from=export U2 - L637296716	wrong population		Round I
Wu, H. and Huang, N. and Zhao, C.	Outcomes of anlotinib plus nabpaclitaxel/gemcitabine as first-line treatment for patients with advanced pancreatic adenocarcinoma: A retrospective analysis in China	2022	J. Clin. Oncol.	https://www.embase.com/search/results?subaction=viewrecord&id=L637297129&from=export U2 - L637297129	wrong outcome	10.1200/JCO.2022.40.4_suppl.556	Round I
Lu W and Li X and Luo Y	FGF21 in obesity and cancer: New insights.	2021	Cancer letters	https://pubmed.ncbi.nlm.nih.gov/33264641/	wrong publication type	10.1016/j.canlet.2020.11.026	Round I
Katoh M and Katoh M	Precision medicine for human cancers with Notch signaling dysregulation (Review).	2020	International journal of molecular medicine	https://pubmed.ncbi.nlm.nih.gov/31894255/	wrong publication type	10.3892/ijmm.2019.4418	Round I
Syed YY	Surufatinib: First Approval.	2021	Drugs	https://pubmed.ncbi.nlm.nih.gov/33788183/	wrong population		Round I
Ndlovu R and Deng LC and Wu J and Li XK and Zhang JS	Fibroblast Growth Factor 10 in Pancreas Development and Pancreatic Cancer.	2018	Frontiers in genetics	https://pubmed.ncbi.nlm.nih.gov/30425728/	wrong outcome	10.3389/fgene.2018.00482	Round I
Ishiwata T	Role of fibroblast growth factor receptor-2 splicing in normal and cancer cells.	2018	Frontiers in bioscience (Landmark edition)	https://pubmed.ncbi.nlm.nih.gov/28930565/	wrong outcome	10.2741/4609	Round I
Carter EP and Coetzee AS and Tomas Bort E and Wang Q and Kocher HM and Grose RP	Dissecting FGF Signalling to Target Cellular Crosstalk in Pancreatic Cancer.	2021	Cells	https://pubmed.ncbi.nlm.nih.gov/33918004/	wrong publication type	10.3390/cells10040847	Round I
Tsumura T and Doi K and Marusawa H	Precision Medicine of Hepatobiliary and Pancreatic Cancers: Focusing on Clinical Trial Outcomes.	2022	Cancers	https://pubmed.ncbi.nlm.nih.gov/35954337/	wrong population	10.3390/cancers14153674	Round I
Coleman SJ and Bruce C and Chioni AM and Kocher HM and Grose RP	The ins and outs of fibroblast growth factor receptor signalling.	2014	Clinical science (London, England : 1979)	https://pubmed.ncbi.nlm.nih.gov/24780002/	wrong publication type	10.1042/CS20140100	Round I
Ratti M and Orlandi E and Hahne JC and Vecchia S and Citterio C and Anselmi E and Toscani I and Ghidini M	Targeting FGFR Pathways in Gastrointestinal Cancers: New Frontiers of Treatment.	2023	Biomedicines	https://pubmed.ncbi.nlm.nih.gov/37893023/	wrong population	10.3390/biomedicines11102650	Round I
Ishiwata T	Cancer stem cells and epithelial-mesenchymal transition: Novel therapeutic targets for cancer.	2016	Pathology international	https://pubmed.ncbi.nlm.nih.gov/27510923/	wrong publication type	10.1111/pin.12447	Round I
Gnatenko DA and Kopantzev EP and Sverdlov ED	[Fibroblast growth factors and their effects in pancreas organogenesis].	2017	Biomeditsinskaia khimiia	https://pubmed.ncbi.nlm.nih.gov/28781254/	wrong publication type	10.18097/PBMC20176303211	Round I

Huynh JC and Schwab E and Ji J and Kim E and Joseph A and Hendifar A and Cho M and Gong J	Recent Advances in Targeted Therapies for Advanced Gastrointestinal Malignancies.	2020	Cancers	https://pubmed.ncbi.nlm.nih.gov/32384640/	wrong population	10.3390/cancers12051168	Round I
Zhou X and Wang X	Klotho: a novel biomarker for cancer.	2015	Journal of cancer research and clinical oncology	https://pubmed.ncbi.nlm.nih.gov/25086986/	wrong publication type	10.1007/s00432-014-1788-y	Round I
Gnatenko DA and Kopantsev EP and Sverdlov ED	[Role of fibroblast growth factors in pancreatic cancer].	2016	Biomeditsinskaia khimiia	https://pubmed.ncbi.nlm.nih.gov/28026804/	wrong publication type	10.18097/PBMC20166206622	Round I
Wang KQ and Ye ML and Qiao X and Yu ZW and Wu CX and Zheng JF	Circular RNA Fibroblast Growth Factor Receptor 1 Promotes Pancreatic Cancer Progression by Targeting MicroRNA-532-3p/PIK3CB Axis.	2022	Pancreas	https://pubmed.ncbi.nlm.nih.gov/36607937/	wrong outcome	10.1097/MPA.0000000000002119	Round I
Itoh N	FGF10: A multifunctional mesenchymal-epithelial signaling growth factor in development, health, and disease.	2016	Cytokine & growth factor reviews	https://pubmed.ncbi.nlm.nih.gov/26559461/	wrong publication type	10.1016/j.cytogfr.2015.10.001	Round I
Unnisa A and Chettupalli AK and Hussain T and Kamal MA	Recent Advances in Epidermal Growth Factor Receptor Inhibitors (EGFRIs) and their Role in the Treatment of Cancer: A Review.	2022	Anti-cancer agents in medicinal chemistry	https://pubmed.ncbi.nlm.nih.gov/35400324/	wrong drug	10.2174/1871520622666220408090541	Round I
Liu G and Xiong D and Xiao R and Huang Z	Prognostic role of fibroblast growth factor receptor 2 in human solid tumors: A systematic review and meta-analysis.	2017	Tumour biology : the journal of the International Society for Oncodevelopmental Biology and Medicine	https://pubmed.ncbi.nlm.nih.gov/28618942/	wrong publication type	10.1177/1010428317707424	Round I
Waheed A and Purvey S and Saif MW	Masitinib in treatment of pancreatic cancer.	2018	Expert opinion on pharmacotherapy	https://pubmed.ncbi.nlm.nih.gov/29638149/	wrong publication type	10.1080/14656566.2018.1459566	Round I
Varna D and Geromichalou E and Karlioti G and Papi R and Dalezis P and Hatzidimitriou AG and Psomas G and Choli-Papadopoulou T and Trafalis DT and Angaridis PA	Inhibition of Cancer Cell Proliferation and Bacterial Growth by Silver(I) Complexes Bearing a CH(3)-Substituted Thiadiazole-Based Thioamide.	2023	Molecules (Basel, Switzerland)	https://pubmed.ncbi.nlm.nih.gov/36615533/	wrong publication type	10.3390/molecules28010336	Round I
Dasari A and Hamilton EP and Falchook GS and Wang JS and Li D and Sung MW and Chien C and Nanda S and Tucci C and Hahka-Kemppinen M and Paulson AS	A dose escalation/expansion study evaluating dose, safety, and efficacy of the novel tyrosine kinase inhibitor surufatinib, which inhibits VEGFR 1, 2, & 3, FGFR 1, and CSF1R, in US patients with neuroendocrine tumors.	2023	Investigational new drugs	https://pubmed.ncbi.nlm.nih.gov/37074571/	wrong population		Round I
Zhang G and Zhai N and Zhang X	Alkannin represses growth of pancreatic cancer cells based on the down regulation of miR-199a.	2020	BioFactors (Oxford, England)	https://pubmed.ncbi.nlm.nih.gov/31967380/	wrong drug	10.1002/biof.1613	Round I
Tong Z and Wang L and Shi W and Zeng Y and Zhang H and Liu L and Zheng Y and Chen C and Xia W and Fang W and Zhao P	Clonal Evolution Dynamics in Primary and Metastatic Lesions of Pancreatic Neuroendocrine Neoplasms.	2021	Frontiers in medicine	https://pubmed.ncbi.nlm.nih.gov/34026777/	wrong population	10.3389/fmed.2021.620988	Round I
Guo Y and Cui J and Liang X and Chen T and Lu C and Peng T	Pancreatic cancer stem cell-derived exosomal miR-210 mediates macrophage M2 polarization and promotes gemcitabine resistance by targeting FGFRL1.	2024	International immunopharmacology	https://pubmed.ncbi.nlm.nih.gov/38134594/	wrong population	10.1016/j.intimp.2023.111407	Round I

Preis M and Korc M	Signaling pathways in pancreatic cancer.	2011	Critical reviews in eukaryotic gene expression	https://pubmed.ncbi.nlm.nih.gov/22077151/	wrong publication type	10.1615/critreveukargeneexpr.v21.i2.20	Round I
Day HR Jr and Finn AP	Serous Retinopathy Associated With Combination MEK and Fibroblast Growth Factor Receptor Inhibitor.	2023	Journal of vitreoretinal diseases	https://pubmed.ncbi.nlm.nih.gov/37927314/	wrong outcome	10.1177/24741264231163393	Round I
Saloman DS and Bianco C and Ebert AD and Khan NI and De Santis M and Normanno N and Wechselberger C and Seno M and Williams K and Sanicola M and Foley S and Gullick WJ and Persico G	The EGF-CFC family: novel epidermal growth factor-related proteins in development and cancer.	2000	Endocrine-related cancer	https://pubmed.ncbi.nlm.nih.gov/11174844/	wrong outcome	10.1677/erc.0.0070199	Round I
Tian X and Chen G and Zhou S and Henne-Bruns D and Bachem M and Kornmann M	Interactions of pancreatic cancer and stellate cells are mediated by FGFR1-III isoform expression.	2012	Hepato-gastroenterology	https://pubmed.ncbi.nlm.nih.gov/22683979/	wrong outcome	10.5754/hge10366	Round I
Domenichini A and Casari I and Simpson PV and Desai NM and Chen L and Dustin C and Edmands JS and van der Vliet A and Mohammadi M and Massi M and Falasca M	Rhenium N-heterocyclic carbene complexes block growth of aggressive cancers by inhibiting FGFR- and SRC-mediated signalling.	2020	Journal of experimental & clinical cancer research : CR	https://pubmed.ncbi.nlm.nih.gov/33287862/	wrong population	10.1186/s13046-020-01777-7	Round I
Inaguma S and Ito H and Riku M and Ikeda H and Kasai K	Addiction of pancreatic cancer cells to zinc-finger transcription factor ZIC2.	2015	Oncotarget	https://pubmed.ncbi.nlm.nih.gov/26318045/	wrong population		Round I
Li Y and Wu L and Tao W and Wu D and Ma F and Li N	Expression Atlas of FGF and FGFR Genes in Pancancer Uncovered Predictive Biomarkers for Clinical Trials of Selective FGFR Inhibitors.	2020	BioMed research international	https://pubmed.ncbi.nlm.nih.gov/32596330/	wrong outcomes	10.1155/2020/5658904	Round I
Zhou L and Yao LT and Liang ZY and Zhou WX and You L and Shao QQ and Huang S and Guo JC and Zhao YP	Nuclear translocation of fibroblast growth factor receptor 3 and its significance in pancreatic cancer.	2015	International journal of clinical and experimental pathology	https://pubmed.ncbi.nlm.nih.gov/26823787/	wrong outcome		Round I
Tsujie, M. and Iwai, T. and Kubo, S. and Ura, T. and Hatano, E. and Sakai, D. and Takeda, Y. and Kaibori, M. and Kobayashi, T. and Katanuma, A. and Katayose, Y. and Fukase, K. and Sakurai, N. and Ito, Y. and Sato, F. and Maeda, A. and Asada, M. and Morizane, C.	Erratum: Fibroblast growth factor receptor 2 (FGFR2) fusions in Japanese patients with intrahepatic cholangiocarcinoma (Japanese Journal of Clinical Oncology DOI: 10.1093/jjco/hyab029)	2021	Jpn. J. Clin. Oncol.	https://www.embase.com/search/results?subaction=viewrecord&id=L2021197347&from=export U2 - L2021197347	wrong publication type	10.1093/jjco/hyab156	Round I
Filipiak, W. and Jaroch, K. and Szeliska, P. and Å»uchowska, K. and Bojko, B.	Application of thin-film microextraction to analyze volatile metabolites in a549 cancer cells	2021	Metabolites	https://www.embase.com/search/results?subaction=viewrecord&id=L2014331431&from=export U2 - L2014331431	wrong drug	10.3390/metabo11100704	Round I
Caccese, M. and Salani, F. and Catanese, S. and Massa, V. and Bernardini, L. and Cesario, S. and Genovesi, V. and Berra, D. and Graziani, J. and Vivaldi, C. and Lencioni, M. and Fornaro, L. and Masi, G. and Falcone, A. and Vasile, E.	Whole-genome next generation sequencing (WG-NGS) in advanced pancreatic cancer (PC)	2021	Tumori	https://www.embase.com/search/results?subaction=viewrecord&id=L636558980&from=export U2 - L636558980	wrong population		Round I
Devi, N. and Kaur, K. and Biharee, A. and Jaitak, V.	Recent development in indole derivatives as anticancer agent: A mechanistic approach	2021	Anti-Cancer Agents Med. Chem.	https://www.embase.com/search/results?subaction=viewrecord&id=L2013008020&from=export U2 - L2013008020	wrong drug	10.2174/1871520621999210104192644	Round I

Wiedlocha, A. and Haugsten, E.M. and Zakrzewska, M.	Roles of the fgf-fgfr signaling system in cancer development and inflammation	2021	Cells	https://www.embase.com/search/results?subaction=viewrecord&id=L2013565004&from=export U2 - L2013565004	wrong outcome	10.3390/cells10092231	Round I
Shi, G.-M. and Huang, X.-Y. and Wen, T.-F. and Song, T.-Q. and Kuang, M. and Mou, H.-B. and Bao, L.-Q. and Zhao, H.-T. and Zhao, H. and Feng, X.-L. and Zhang, B. and Peng, T. and Zhang, Y. and Li, X. and Yu, H. and Cao, Y. and Luo, Y. and Wang, Y. and Fan, J. and Zhou, J.	50P Efficacy and safety of pemigatinib in Chinese patients with unresectable, advanced/ recurrent or metastatic intrahepatic cholangiocarcinoma with FGFR2 fusion or rearrangement that failed to prior systemic therapy	2021	Ann. Oncol.	https://www.embase.com/search/results?subaction=viewrecord&id=L2014621326&from=export U2 - L2014621326	wrong population	10.1016/j.annonc.2021.08.329	Round I
Kuboki, Y. and Shitara, K. and Morizane, C. and Kojima, T. and Yoh, K. and Sakai, D. and Tahara, M. and Hirai, H. and Kurokawa, Y. and Kato, T. and Doi, T.	1383P Phase I study of the irreversible FGFR inhibitor futibatinib in Japanese patients with advanced solid tumors: Updated dose expansion results and activity in gastric cancer	2021	Ann. Oncol.	https://www.embase.com/search/results?subaction=viewrecord&id=L2014622580&from=export U2 - L2014622580	wrong population		Round I
Garcia-Corbacho, J. and Victoria Ruiz, I. and Angelats, L. and Indacochea, A. and MartÃ-nez-Vidal, J. and Sole Bentz, P. and Moreno, D. and Faull, I. and Campillo, M.L. and MuÃ±oz, S. and SaurÃ-, T. and Reguart Aransay, N. and ViÃ±olas, N. and Esposito, F.M. and Mezquita, L. and BastÃ©, N. and Oliveres, H. and Castillo, S. and NoguÃ©, M. and Prat, A.	92P First-results of the CLIMB360 study, a prospective molecular screening program across multiple cancer types based on circulating tumor DNA (ctDNA)	2021	Ann. Oncol.	https://www.embase.com/search/results?subaction=viewrecord&id=L2014623066&from=export U2 - L2014623066	wrong population		Round I
Hibino, Y. and Ito, M. and Satake, T. and Kondo, S.	Clinical benefits of precision medicine in treating solid cancers: European Society of Medical Oncology-Magnitude of Clinical Benefit Scale score-based analysis	2021	ESMO Open	https://www.embase.com/search/results?subaction=viewrecord&id=L2012927198&from=export U2 - L2012927198	wrong population		Round I
Sasaki, T. and Takeda, T. and Okamoto, T. and Ozaka, M. and Sasahira, N.	Chemotherapy for biliary tract cancer in 2021	2021	J. Clin. Med.	https://www.embase.com/search/results?subaction=viewrecord&id=L2007805735&from=export U2 - L2007805735	wrong population	10.3390/jcm10143108	Round I
Derby, S. and Wallace, J. and Venugopal, B. and Cascales Garcia, M.A.	Advanced penile cancer presenting with renal failure	2021	ONCOLOGY	https://www.embase.com/search/results?subaction=viewrecord&id=L2013847842&from=export U2 - L2013847842	wrong population		Round I
Dexheimer, T.S. and Silvers, T. and Delosh, R. and Laudeman, J. and Reinhart, R. and Ogle, C. and Coussens, N.P. and Wright, J. and Teicher, B.A. and Doroshow, J.H.	Combination screening of KRASG12C specific inhibitors with other targeted therapies in patient-derived multicellular tumor spheroids	2021	Cancer Res.	https://www.embase.com/search/results?subaction=viewrecord&id=L635903586&from=export U2 - L635903586	wrong drug	10.1158/1538-7445.AM2021-1028	Round I
Wang, Z. and Yang, X. and Li, W. and Wu, Z. and Ma, Q. and Qi, X. and Zhao, J.	Genomic profiling of Chinese adenosquamous carcinoma of the pancreas	2021	Cancer Res.	https://www.embase.com/search/results?subaction=viewrecord&id=L635903879&from=export U2 - L635903879	wrong outcomes	10.1158/1538-7445.AM2021-2659	Round I
Noguchi, R. and Yoshimatsu, Y. and Sei, A. and Hirabayashi, K. and Ozawa, I. and Kikuta, K. and Kondo, T.	Establishment and characterization of NCC-MLPS1-C1: a novel patient-derived cell line of myxoid liposarcoma	2021	Hum. Cell	https://www.embase.com/search/results?subaction=viewrecord&id=L2007157461&from=export U2 - L2007157461	wrong population		Round I

Shetty, S.R. and Yeeravalli, R. and Bera, T. and Das, A.	Recent advances on epidermal growth factor receptor as a molecular target for breast cancer therapeutics	2021	Anti-Cancer Agents Med. Chem.	https://www.embase.com/search/results?subaction=viewrecord&id=L2013008018&from=export U2 - L2013008018	wrong population	10.2174/187152062166620122143213	Round I
Xu, J.	Current treatments and future potential of surufatinib in neuroendocrine tumors (NETs)	2021	Ther. Adv. Med. Oncol.	https://www.embase.com/search/results?subaction=viewrecord&id=L2013581779&from=export U2 - L2013581779	wrong population	10.1177/175883592111042689	Round I
Gu, W. and Yang, J. and Wang, Y. and Xu, J. and Wang, X. and Du, F. and Hu, X. and Guo, H. and Song, C. and Tao, R. and Zhang, X.	Comprehensive identification of FGFR1-4 alterations in 5 557 Chinese patients with solid tumors by next-generation sequencing	2021	Am. J. Cancer Res.	https://www.embase.com/search/results?subaction=viewrecord&id=L2014397625&from=export U2 - L2014397625	wrong outcome		Round I
Ueno, M. and Ikeda, M. and Sasaki, T. and Nagashima, F. and Mizuno, N. and Shimizu, S. and Ikezawa, H. and Hayata, N. and Nakajima, R. and Morizane, C.	Phase 2 study of lenvatinib monotherapy as second-line treatment in unresectable biliary tract cancer: primary analysis results	2020	BMC Cancer	https://www.embase.com/search/results?subaction=viewrecord&id=L2007309543&from=export U2 - L2007309543	wrong population		Round I
Bono F and De Smet F and Herbert C and De Bock K and Georgiadou M and Fons P and Tjwa M and Alcouffe C and Ny A and Bianciotto M and Jonckx B and Murakami M and Lanahan AA and Michielsen C and Sibrac D and Dol-Gleizes F and Mazzone M and Zacchigna S and Herault JP and Fischer C and Rigon P and Ruiz de Almodovar C and Claes F and Blanc I and Poesen K and Zhang J and Segura I and Gueguen G and Bordes MF and Lambrechts D and Broussy R and van de Wouwer M and Michaux C and Shimada T and Jean I and Blacher S and Noel A and Motte P and Rom E and Rakic JM and Katsuma S and Schaeffer P and Yayon A and Van Schepdael A and Schwalbe H and Gervasio FL and Carmeliet G and Rozensky J and Dewerchin M and Simons M and Christopoulos A and Herbert JM and Carmeliet P	Inhibition of tumor angiogenesis and growth by a small-molecule multi-FGF receptor blocker with allosteric properties.	2013	Cancer cell	https://pubmed.ncbi.nlm.nih.gov/23597562/	wrong population	10.1016/j.ccr.2013.02.019	Round I
Rasam S and Lin Q and Shen S and Straubinger RM and Qu J	Highly Reproducible Quantitative Proteomics Analysis of Pancreatic Cancer Cells Reveals Proteome-Level Effects of a Novel Combination Drug Therapy That Induces Cancer Cell Death via Metabolic Remodeling and Activation of the Extrinsic Apoptosis Pathway.	2023	Journal of proteome research	https://pubmed.ncbi.nlm.nih.gov/37906173/	wrong outcome	10.1021/acs.jproteome.3c00463	Round I
El-Hariry I and Pignatelli M and Lemoine NR	FGF-1 and FGF-2 modulate the E-cadherin/catenin system in pancreatic adenocarcinoma cell lines.	2001	British journal of cancer	https://pubmed.ncbi.nlm.nih.gov/11401320/	wrong publication type	10.1054/bjoc.2001.1813	Round I
Ishiwata T and Matsuda Y and Yamamoto T and Uchida E and Korc M and Naito Z	Enhanced expression of fibroblast growth factor receptor 2 IIIc promotes human pancreatic cancer cell proliferation.	2012	The American journal of pathology	https://pubmed.ncbi.nlm.nih.gov/22440254/	wrong outcome	10.1016/j.ajpath.2012.01.020	Round I

Coetzee AS and Carter EP and RodrÃ-guez-FernÃ¡ndez L and Heward J and Wang Q and Karim SA and Boughetane L and Milton C and Uyulur F and Morton JP and Kocher HM and Grose RP	Nuclear FGFR1 promotes pancreatic stellate cell-driven invasion through up-regulation of Neuregulin 1.	2023	Oncogene	https://pubmed.ncbi.nlm.nih.gov/36357571/	wrong outcome	10.1038/s41388-022-02513-5	Round I
NOT REPORTED	Association between klotho expression and malignancies risk and progression: A meta-analysis.	2018	Clinica chimica acta		wrong population		Round I
D'Agosto S and Pezzini F and Veghini L and Delfino P and Fiorini C and Temgue Tane GD and Del Curatolo A and Vicentini C and Ferrari G and Pasini D and Andreani S and Lupo F and Fiorini E and Lorenzon G and Lawlor RT and Rusev B and Malinova A and Luchini C and Milella M and Sereni E and Pea A and Bassi C and Bailey P and Scarpa A and Bria E and Corbo V	Loss of FGFR4 promotes the malignant phenotype of PDAC.	2022	Oncogene	https://pubmed.ncbi.nlm.nih.gov/35963908/	wrong outcome	10.1038/s41388-022-02432-5	Round I
Luo Y and Yang Y and Liu M and Wang D and Wang F and Bi Y and Ji J and Li S and Liu Y and Chen R and Huang H and Wang X and Swidnicka-Siergiejko AK and Janowitz T and Beyaz S and Wang G and Xu S and Bialkowska AB and Luo CK and Pin CL and Liang G and Lu X and Wu M and Shroyer KR and Wolff RA and Plunkett W and Ji B and Li Z and Li E and Li X and Yang VW and Logsdon CD and Abbruzzese JL and Lu W	Oncogenic KRAS Reduces Expression of FGF21 in Acinar Cells to Promote Pancreatic Tumorigenesis in Mice on a High-Fat Diet.	2019	Gastroenterology	https://pubmed.ncbi.nlm.nih.gov/31352001/	wrong population		Round I
Haq F and Sung YN and Park I and Kayani MA and Yousuf F and Hong SM and Ahn SM	FGFR1 expression defines clinically distinct subtypes in pancreatic cancer.	2018	Journal of translational medicine	https://pubmed.ncbi.nlm.nih.gov/30593273/	wrong outcome	10.1186/s12967-018-1743-9	Round I
Zhang Z and Qin Y and Ji S and Xu W and Liu M and Hu Q and Ye Z and Fan G and Yu X and Liu W and Xu X	FGFBP1-mediated crosstalk between fibroblasts and pancreatic cancer cells via FGF22/FGFR2 promotes invasion and metastasis of pancreatic cancer.	2021	Acta biochimica et biophysica Sinica	https://pubmed.ncbi.nlm.nih.gov/34117747/	wrong outcome	10.1093/abbs/gmab074	Round I
Zhou L and Lu J and Liang ZY and Zhou WX and Jiang BL and Wang YZ and Liu CX and Huang BW and You L and Guo JC	FGFR3 Nuclear Translocation Contributes to Proliferative Potential and Poor Prognosis in Pancreatic Ductal Adenocarcinoma.	2022	Pancreas	https://pubmed.ncbi.nlm.nih.gov/35858182/	wrong outcome	10.1097/MPA.00000000000002056	Round I
El-Hariry I and Pignatelli M and Lemoine NR	FGF-1 and FGF-2 regulate the expression of E-cadherin and catenins in pancreatic adenocarcinoma.	2001	International journal of cancer	https://pubmed.ncbi.nlm.nih.gov/11745459/	wrong outcome	10.1002/ijc.1515	Round I
Korc M	Role of growth factors in pancreatic cancer.	1998	Surgical oncology clinics of North America	https://pubmed.ncbi.nlm.nih.gov/9443985/	wrong publication type		Round I
Marech I and Patruno R and Zizzo N and Gadaleta C and Introna M and Zito AF and Gadaleta CD and Ranieri G	Masitinib (AB1010), from canine tumor model to human clinical development: where we are?	2014	Critical reviews in oncology/hematology	https://pubmed.ncbi.nlm.nih.gov/24405856/	wrong population	10.1016/j.critrevonc.2013.12.011	Round I

Crnogorac-Jurcevic T and Efthimiou E and Capelli P and Blaveri E and Baron A and Terris B and Jones M and Tyson K and Bassi C and Scarpa A and Lemoine NR	Gene expression profiles of pancreatic cancer and stromal desmoplasia.	2001	Oncogene	https://pubmed.ncbi.nlm.nih.gov/11704875/	wrong publication type	10.1038/sj.onc.1204935	Round I
Ezzat S and Zheng L and Florez JC and Stefan N and Mayr T and Hliang MM and Jablonski K and Harden M and StanÃÂ¡kovÃ¡ A and Laakso M and Haring HU and Ullrich A and Asa SL	The cancer-associated FGFR4-G388R polymorphism enhances pancreatic insulin secretion and modifies the risk of diabetes.	2013	Cell metabolism	https://pubmed.ncbi.nlm.nih.gov/23747250/	wrong outcomes	10.1016/j.cmet.2013.05.002	Round I
Watase C and Fuse M and Ino Y and Naito C and Hiraoka N	Novel insights into immunohistochemical analysis for diagnosing serous neoplasm of the pancreas: aquaporin 1, stereocilin, and transmembrane protein 255B.	2021	Histopathology	https://pubmed.ncbi.nlm.nih.gov/34288030/	wrong publication type	10.1111/his.14456	Round I
Chen G and Tian X and Liu Z and Zhou S and Schmidt B and Henne-Bruns D and Bachem M and Kornmann M	Inhibition of endogenous SPARC enhances pancreatic cancer cell growth: modulation by FGFR1-III isoform expression.	2010	British journal of cancer	https://pubmed.ncbi.nlm.nih.gov/19920824/	wrong publication type	10.1038/sj.bjc.6605440	Round I
Oberg K	Neuroendocrine tumours in 2012: Insights into signalling pathways could individualize therapy.	2013	Nature reviews. Endocrinology	https://pubmed.ncbi.nlm.nih.gov/23296177/	wrong population	10.1038/nrendo.2012.250	Round I
Ohta T and Yamamoto M and Numata M and Iseki S and Tsukioka Y and Miyashita T and Kayahara M and Nagakawa T and Miyazaki I and Nishikawa K and Yoshitake Y	Expression of basic fibroblast growth factor and its receptor in human pancreatic carcinomas.	1995	British journal of cancer	https://pubmed.ncbi.nlm.nih.gov/7547227/	wrong outcome	10.1038/bjc.1995.420	Round I
Wulbrand U and Wied M and ZÃ¶fel P and GÃ¶ke B and Arnold R and Fehmann H	Growth factor receptor expression in human gastroenteropancreatic neuroendocrine tumours.	1998	European journal of clinical investigation	https://pubmed.ncbi.nlm.nih.gov/9893017/	wrong population	10.1046/j.1365-2362.1998.00397.x	Round I
Motoda N and Matsuda Y and Onda M and Ishiwata T and Uchida E and Naito Z	Overexpression of fibroblast growth factor receptor 4 in high-grade pancreatic intraepithelial neoplasia and pancreatic ductal adenocarcinoma.	2011	International journal of oncology	https://pubmed.ncbi.nlm.nih.gov/21109934/	wrong outcome		Round I
Leung H and Hughes C and Kloppel G and Williamson R and Lemoine N	Localization of expression of fibroblast growth-factors and their receptors in pancreatic adenocarcinoma by in-situ hybridization.	1994	International journal of oncology	https://pubmed.ncbi.nlm.nih.gov/21567040/	wrong population	10.3892/ijo.4.6.1219	Round I
Lafitte M and Moranvillier I and Garcia S and Peuchant E and Iovanna J and Rousseau B and Dubus P and Guyonnet-DupÃ©rat V and BelleannÃ©e G and Ramos J and Bedel A and de Verneuil H and Moreau-Gaudry F and Dabernat S	FGFR3 has tumor suppressor properties in cells with epithelial phenotype.	2013	Molecular cancer	https://pubmed.ncbi.nlm.nih.gov/23902722/	wrong outcome	10.1186/1476-4598-12-83	Round I
Xu J and Wang T and Cao Z and Huang H and Li J and Liu W and Liu S and You L and Zhou L and Zhang T and Zhao Y	MiR-497 downregulation contributes to the malignancy of pancreatic cancer and associates with a poor prognosis.	2014	Oncotarget	https://pubmed.ncbi.nlm.nih.gov/25149530/	wrong outcomes	10.18632/oncotarget.2184	Round I
Li N and Zhao X and You S	Identification of key regulators of pancreatic ductal adenocarcinoma using bioinformatics analysis of microarray data.	2019	Medicine	https://pubmed.ncbi.nlm.nih.gov/30633213/	wrong outcomes	10.1097/MD.0000000000014074	Round I
Itoh N and Ohta H	Fgf10: a paracrine-signaling molecule in development, disease, and regenerative medicine.	2014	Current molecular medicine	https://pubmed.ncbi.nlm.nih.gov/24730525/	wrong outcomes	10.2174/1566524014666140414204829	Round I

Chen S and Guo W and Meng M and Wu D and Zhou T and Wang L and Xu J	LncRNA SNHG1 Promotes the Progression of Pancreatic Cancer by Regulating FGFR1 Expression via Competitively Binding to miR-497.	2022	Frontiers in oncology	https://pubmed.ncbi.nlm.nih.gov/35141164/	wrong publication type	10.3389/fonc.2022.813850	Round I
Huang CC and Liu CY and Huang CJ and Hsu YC and Lien HH and Wong JU and Tai FC and Ku WH and Hung CF and Lin JT and Huang CS and Chiang HS	Deciphering Genetic Alterations of Taiwanese Patients with Pancreatic Adenocarcinoma through Targeted Sequencing.	2022	International journal of molecular sciences	https://pubmed.ncbi.nlm.nih.gov/35163506/	wrong outcome	10.3390/ijms23031579	Round I
Manchado E and Weissmueller S and Morris JP 4th and Chen CC and Wullenkord R and Lujambio A and de Stanchina E and Poirier JT and Gainor JF and Corcoran RB and Engelman JA and Rudin CM and Rosen N and Lowe SW	A combinatorial strategy for treating KRAS-mutant lung cancer.	2016	Nature	https://pubmed.ncbi.nlm.nih.gov/27338794/	wrong population		Round I
Ceccarelli S and Bei R and Vescarelli E and D'Amici S and di Gioia C and Modesti A and Romano F and Redler A and Marchese C and Angeloni A	Potential prognostic and diagnostic application of a novel monoclonal antibody against keratinocyte growth factor receptor.	2014	Molecular biotechnology	https://pubmed.ncbi.nlm.nih.gov/24899248/	wrong drug	10.1007/s12033-014-9773-x	Round I
Laitman Y and Kuchenbaecker KB and Rantala J and Hogervorst F and Peock S and Godwin AK and Arason A and Kirchhoff T and Offit K and Isaacs C and Schmutzler RK and Wappenschmidt B and Nevanlinna H and Chen X and Chenevix-Trench G and Healey S and Couch F and Peterlongo P and Radice P and Nathanson KL and Caligo MA and Neuhausen SL and Ganz P and Sinilnikova OM and McGuffog L and Easton DF and Antoniou AC and Wolf I and Friedman E	The KL-VS sequence variant of Klotho and cancer risk in BRCA1 and BRCA2 mutation carriers.	2012	Breast cancer research and treatment	https://pubmed.ncbi.nlm.nih.gov/22212556/	wrong drug	10.1007/s10549-011-1938-8	Round I
Chen G and Wang J and Liu Z and Kornmann M	Exon III splicing of fibroblast growth factor receptor 1 is modulated by growth factors and cyclin D1.	2008	Pancreas	https://pubmed.ncbi.nlm.nih.gov/18665077/	wrong publication type	10.1097/MPA.0b013e31816618a4	Round I
Serra S and Zheng L and Hassan M and Phan AT and Woodhouse LJ and Yao JC and Ezzat S and Asa SL	The FGFR4-G388R single-nucleotide polymorphism alters pancreatic neuroendocrine tumor progression and response to mTOR inhibition therapy.	2012	Cancer research	https://pubmed.ncbi.nlm.nih.gov/22986737/	wrong population	10.1158/0008-5472.CAN-12-2102	Round I
Kothari V and Wei I and Shankar S and Kalyana-Sundaram S and Wang L and Ma LW and Vats P and Grasso CS and Robinson DR and Wu YM and Cao X and Simeone DM and Chinnaiyan AM and Kumar-Sinha C	Outlier kinase expression by RNA sequencing as targets for precision therapy.	2013	Cancer discovery	https://pubmed.ncbi.nlm.nih.gov/23384775/	wrong population		Round I
Vickers SM and Huang ZQ and MacMillan-Crow L and Greendorfer JS and Thompson JA	Ligand activation of alternatively spliced fibroblast growth factor receptor-1 modulates pancreatic adenocarcinoma cell malignancy.	2002	Journal of gastrointestinal surgery : official journal of the Society for Surgery o f the Alimentary Tract	https://pubmed.ncbi.nlm.nih.gov/12127120/	wrong population		Round I

Sahin, I.H.	Immune checkpoint inhibitor response in mismatch repair-deficient colorectal cancer and other solid tumors: Is it truly disease-agnostic?	2020	Colorectal Cancer	https://www.embase.com/search/results?subaction=viewrecord&id=L633716303&from=export U2 - L633716303	wrong population	10.2217/crc-2020-0020	Round I
Morizane, C. and Ueno, M. and Ioka, T. and Tajika, M. and Ikeda, M. and Yamaguchi, K. and Hara, H. and Yabusaki, H. and Miyamoto, A. and Iwasa, S. and Muto, M. and Takashima, T. and Minashi, K. and Komatsu, Y. and Nishina, T. and Nakajima, T. and Sahara, T. and Funasaka, S. and Yashiro, M. and Furuse, J.	Clinical update with plasma and tumour-based genomic analyses in expansion part of phase I study of selective FGFR inhibitor E7090	2020	Ann. Oncol.	https://www.embase.com/search/results?subaction=viewrecord&id=L2008604068&from=export U2 - L2008604068	wrong population		Round I
Furuse, J. and Goyal, L. and Meric-Bernstam, F. and Hollebecque, A. and Valle, J.W. and Morizane, C. and Karasic, T.B. and Abrams, T.A. and Kelley, R.K. and Cassier, P.A. and Klumpen, H.-J. and Uboha, N. and Mahipal, A. and Mitchell, E. and Ahn, E. and Chang, H.-M. and Masuda, K. and He, Y. and Benhadji, K.A. and Bridgewater, J.A.	116MO Efficacy, safety, and quality of life (QoL) with futibatinib in patients (pts) with intrahepatic cholangiocarcinoma (iCCA) harboring FGFR2 fusions/rearrangements: FOENIX-CCA2	2020	Ann. Oncol.	https://www.embase.com/search/results?subaction=viewrecord&id=L2008604221&from=export U2 - L2008604221	wrong population		Round I
Lenkiewicz, E. and Malasi, S. and Hogenson, T.L. and Flores, L.F. and Barham, W. and Phillips, W.J. and Roesler, A.S. and Chambers, K.R. and Rajbhandari, N. and Hayashi, A. and Antal, C.E. and Downes, M. and Grandgenett, P.M. and Hollingsworth, M.A. and Cridebring, D. and Xiong, Y. and Lee, J.-H. and Ye, Z. and Yan, H. and Hernandez, M.C. and Leiting, J.L. and Evans, R.M. and Ordog, T. and Truty, M.J. and Borad, M.J. and Reya, T. and von Hoff, D.D. and Fernandez-Zapico, M.E. and Barrett, M.T.	Genomic and epigenomic landscaping defines new therapeutic targets for adenosquamous carcinoma of the pancreas	2020	Cancer Res.	https://www.embase.com/search/results?subaction=viewrecord&id=L2010904187&from=export U2 - L2010904187	wrong publication type	10.1158/0008-5472.CAN-20-0078	Round I
Sun, Y. and Li, G. and Zhu, W. and He, Q. and Liu, Y. and Chen, X. and Liu, J. and Lin, J. and Han-Zhang, H. and Yang, Z. and Lizaso, A. and Xiang, J. and Mao, X. and Liu, H. and Gao, Y.	A comprehensive pan-cancer study of fibroblast growth factor receptor aberrations in Chinese cancer patients	2020	Ann. Transl. Med.	https://www.embase.com/search/results?subaction=viewrecord&id=L2010026206&from=export U2 - L2010026206	wrong population		Round I
Rohr, M.W. and Lessans, S. and Aljabban, J. and Hadley, D. and Altomare, D.	Fibroblast Growth Factor Receptor 4 is a Putative Suppressor of Pancreatic Cancer Cell Migration and Gemcitabine Resistance	2020	Am. J. Gastroenterol.	https://www.embase.com/search/results?subaction=viewrecord&id=L633656341&from=export U2 - L633656341	wrong population		Round I
Wang, Y. and Zhong, X. and Zhou, L. and Lu, J. and Jiang, B. and Liu, C. and Guo, J.	Prognostic Biomarkers for Pancreatic Ductal Adenocarcinoma: An Umbrella Review	2020	Front. Oncol.	https://www.embase.com/search/results?subaction=viewrecord&id=L632997736&from=export U2 - L632997736	wrong publication type	10.3389/fonc.2020.01466	Round I

Bridgewater, J. and Meric-Bernstam, F. and Hollebecque, A. and Valle, J.W. and Morizane, C. and Karasic, T. and Abrams, T. and Furuse, J. and Kelley, R.K. and Cassier, P. and KlÃ¼mpen, H.-J. and Uboha, N. and Mahipal, A. and Mitchell, E. and Ahn, E.R. and Chang, H.-M. and Masuda, K. and He, Y. and Benhadji, K. and Goyal, L.	Efficacy and safety of futibatinib in intrahepatic cholangiocarcinoma (iCCA) harboring FGFR2 fusions/other rearrangements: Subgroup analyses of a phase II study (FOENIX-CCA2)	2020	Ann. Oncol.	https://www.embase.com/search/results?subaction=viewrecord&id=L2007889938&from=export U2 - L2007889938	wrong population		Round I
NOT REPORTED	Phase I study of the irreversible FGFR inhibitor (i) futibatinib (FBN				wrong population		Round I
Iyer, R.V. and Konda, B. and Fountzilas, C. and Mukherjee, S. and Owen, D. and Attwood, K. and Wang, C. and Suffren, S.-A. and Hicks, K. and Wilton, J. and Bies, R. and Casucci, D. and Reidy-Lagunes, D. and Shah, M.	Multicenter phase 2 trial of nintedanib in advanced nonpancreatic neuroendocrine tumors	2020	Cancer	https://www.embase.com/search/results?subaction=viewrecord&id=L2005195549&from=export U2 - L2005195549	wrong population	10.1002/cncr.32994	Round I
Nawalaniec, K. and Wang, Y. and Moench, R. and Cao, M. and Luo, Y. and Hsiao, L.-L. and Waaga-Gasser, A.M.	IL-6/STAT3-mediated inflammatory signaling results in dysregulated fibroblast growth factor receptor activation and tumor cell signaling in human pancreatic adenocarcinoma	2020	Cancer Res.	https://www.embase.com/search/results?subaction=viewrecord&id=L633627930&from=export U2 - L633627930	wrong publication type	10.1158/1538-7445.AM2020-992	Round I
NOT REPORTED	Molecularly matched therapy in the context of sensitivity, resistance, and safety				wrong population		Round I
Chae, Y.K. and Hong, F. and Vaklavas, C. and Cheng, H.H. and Hammerman, P. and Mitchell, E.P. and Zwiebel, J.A. and Ivy, S.P. and Gray, R.J. and Li, S. and McShane, L.M. and Rubinstein, L.V. and Patton, D. and Williams, P.M. and Hamilton, S.R. and Mansfield, A. and Conley, B.A. and Arteaga, C.L. and Harris, L.N. and O'Dwyer, P.J. and Chen, A.P. and Flaherty, K.T.	Phase II study of AZD4547 in patients with tumors harboring aberrations in the FGFR pathway: Results from the NCI-MATCH Trial (EAY131) subprotocol W	2020	J. Clin. Oncol.	https://www.embase.com/search/results?subaction=viewrecord&id=L2007511588&from=export U2 - L2007511588	wrong population	10.1200/JCO.19.02630	Round I
Allen, R. and Halpern, N. and Algaze, S. and Golan, T. and El-Khoueiry, A.B. and Shroff, R.T.	Moving beyond chemotherapy for pancreaticobiliary tumors: Targeted and immunotherapy strategies	2020	Am Soc Clin Oncol Educ Book	https://www.embase.com/search/results?subaction=viewrecord&id=L2007022537&from=export U2 - L2007022537	wrong drug	10.1200/EDBK_280901	Round I
Quan, M.-Y. and Guo, Q. and Liu, J. and Yang, R. and Bai, J. and Wang, W. and Cai, Y. and Han, R. and Lv, Y.-Q. and Ding, L. and Billadeau, D.D. and Lou, Z. and Bellusci, S. and Li, X. and Zhang, J.-S.	An FGFR/AKT/SOX2 Signaling Axis Controls Pancreatic Cancer Stemness	2020	Front. Cell Dev. Biol.	https://www.embase.com/search/results?subaction=viewrecord&id=L631801153&from=export U2 - L631801153	wrong publication type	10.3389/fcell.2020.00287	Round I

Maruki, Y. and Morizane, C. and Arai, Y. and Ikeda, M. and Ueno, M. and Ioka, T. and Naganuma, A. and Furukawa, M. and Mizuno, N. and Uwagawa, T. and Takahara, N. and Kanai, M. and Asagi, A. and Shimizu, S. and Miyamoto, A. and Yukisawa, S. and Kadokura, M. and Kojima, Y. and Furuse, J. and Nakajima, T.E. and Sudo, K. and Kobayashi, N. and Hama, N. and Yamanaka, T. and Shibata, T. and Okusaka, T.	Molecular detection and clinicopathological characteristics of advanced/recurrent biliary tract carcinomas harboring the FGFR2 rearrangements: a prospective observational study (PRELUDE Study)	2020	J. Gastroenterol.	https://www.embase.com/search/results?subaction=viewrecord&id=L2007075017&from=export U2 - L2007075017	wrong population		Round I
Kudo, M. and Ikeda, M. and Motomura, K. and Okusaka, T. and Kato, N. and Dutcus, C.E. and Hisai, T. and Suzuki, M. and Ikezawa, H. and Iwata, T. and Kumada, H. and Kobayashi, M.	A phase Ib study of lenvatinib (LEN) plus nivolumab (NIV) in patients (pts) with unresectable hepatocellular carcinoma (uHCC): Study 117	2020	J. Clin. Oncol.	https://www.embase.com/search/results?subaction=viewrecord&id=L630960691&from=export U2 - L630960691	wrong population		Round I
Philip, P.A. and Xiu, J. and Hall, M.J. and Hendifar, A.E. and Lou, E. and Hwang, J.J. and Gong, J. and Khushman, M.M. and Sohal, D. and Lockhart, A.C. and Weinberg, B.A. and Marshall, J. and Grothey, A. and Shields, A.F. and Korn, W.M.	Enrichment of alterations in targetable molecular pathways in KRAS wild-type (WT) pancreatic cancer (PC)	2020	J. Clin. Oncol.	https://www.embase.com/search/results?subaction=viewrecord&id=L632814225&from=export U2 - L632814225	wrong population		Round I
Hogenson, T. and Phillips, W. and Lenkiewicz, E. and Almada, L. and Koenig, A. and Xie, H. and Horn, I. and Levy, M. and Truty, M. and Petersen, G.M. and Barrett, M. and Ma, W.W. and Fernandez-Zapico, M.E.	Patient-Derived Organoids as a Model for Predicting Drug Response in Pancreas Malignancies	2020	Pancreas	https://www.embase.com/search/results?subaction=viewrecord&id=L633717933&from=export U2 - L633717933	wrong publication type		Round I
Llovet, J. and Shepard, K.V. and Finn, R.S. and Ikeda, M. and Sung, M. and Baron, A.D. and Kudo, M. and Okusaka, T. and Kobayashi, M. and Kumada, H. and Kaneko, S. and Pracht, M. and Mamontov, K. and Meyer, T. and Mody, K. and Kubota, T. and Saito, K. and Siegel, A.B. and Dubrovsky, L. and Zhu, A.X.	A phase Ib trial of lenvatinib (LEN) plus pembrolizumab (PEMBRO) in unresectable hepatocellular carcinoma (uHCC): Updated results	2019	Ann. Oncol.	https://www.embase.com/search/results?subaction=viewrecord&id=L2004531581&from=export U2 - L2004531581	wrong population		Round I
Kuznietsova, H. and Dziubenko, N. and Herheliuk, T. and Hurmach, V. and Lynchak, O. and Prylutskyy, Y. and Ritter, U.	C60fullerene: Anti fibrotic, anti tumor and antimetastatic nanotherapeutic for hepatocellular carcinoma treatment	2019	United Eur. Gastroenterol. J.	https://www.embase.com/search/results?subaction=viewrecord&id=L632137452&from=export U2 - L632137452	wrong population		Round I
Yao, M. and Wang, M. and Chen, M. and Shou, T. and Cao, J. and Chen, H. and Wang, A. and Chen, L. and Hu, J. and Zhang, S. and Wang, K.	Landscape of genomic alterations across solid tumors based on a comprehensive clinical sequencing analysis of 5355 Chinese cancer patients	2019	Cancer Res.	https://www.embase.com/search/results?subaction=viewrecord&id=L628695243&from=export U2 - L628695243	wrong publication type	10.1158/1538-7445.SABCS18-5134	Round I
Bu, K. and Lian, Z. and Wang, X. and Lv, G. and Ma, R. and Cui, Q. and Yao, M. and Wang, A. and Chen, H. and Lei, Y.	The landscape of FGFR alteration in Chinese solid tumor patients	2019	Cancer Res.	https://www.embase.com/search/results?subaction=viewrecord&id=L628695361&from=export U2 - L628695361	wrong population		Round I

Wang, W. and Xu, J. and Li, J. and Bai, C. and Xu, N. and Zhou, Z. and Li, Z. and Zhou, C. and Jia, R. and Lu, M. and Cheng, Y. and Mao, C. and Cheng, K. and Su, C. and Hua, Y. and Qi, C. and Li, J. and Li, K. and Sun, Q. and Ren, Y. and Su, W.	Surufatinib in advanced well-differentiated neuroendocrine tumors: A multicenter, single-arm, open-label, phase Ib/II trial	2019	Clin. Cancer Res.	https://www.em-base.com/search/re-sults?subaction=viewre-cord&id=L2002118135&from=export U2 - L2002118135	wrong popu-lation			Round I
Frullanti, E. and Palmieri, M. and Baldassarri, M. and Fava, F. and Fabbiani, A. and Gelli, E. and Tita, R. and Torre, P. and Petrioli, R. and Hadijstilianou, T. and Galimberti, D. and Cinotti, E. and Mencarelli, M.A. and Pinto, A.M. and Francini, E. and Marsili, S. and Francini, G. and Renieri, A.	Specific clonal expansion at disease progression (PD) in solid cancers pinpointed by cell free DNA analysis	2019	J. Clin. On-col.	https://www.em-base.com/search/re-sults?subaction=viewre-cord&id=L629438513&from=export U2 - L629438513	wrong publi-cation type	10.1200/jco.2019.37.15_suppl.e13144		Round I
Loukas, Y.L. and Thodi, G. and Triantafilli, O. and Chatzidaki, M.	Tumor DX: The most detailed molecular oncology profile-application to solid tumors (FFPE & liquid biopsy)	2019	Forum Clin. Oncol.	https://www.em-base.com/search/re-sults?subaction=viewre-cord&id=L631037487&from=export U2 - L631037487	wrong popu-lation			Round I
Senoo, S. and Ninomiya, T. and Makimoto, G. and Nishii, K. and Kano, H. and Watanabe, H. and Hata, Y. and Kubo, T. and Tanaka, T. and Hotta, K. and Maeda, Y. and Kiura, K.	Rapid and long-term response of pulmonary pleomorphic carcinoma to nivolumab	2019	Intern. Med.	https://www.em-base.com/search/re-sults?subaction=viewre-cord&id=L2001890854&from=export U2 - L2001890854	wrong publi-cation type	10.2169/inter-nalmedi-cine.0890-18		Round I
Finn, R.S. and Kudo, M. and Cheng, A.-L. and Wyr-wicz, L. and Ngan, R. and Blanc, J.F. and Baron, A.D. and Vogel, A. and Ikeda, M. and Piscaglia, F. and Han, K.-H. and Qin, S. and Minoshima, Y. and Kanekiyo, M. and Ren, M. and Dairiki, R. and Tamai, T. and Dutcus, C.E. and Fu-nahashi, Y. and Evans, T.R.J.	Final analysis of serum bi-omarkers in patients (pts) from the phase III study of len-vatinib (LEN) vs sorafenib (SOR) in unresectable hepato-cellular carcinoma (uHCC) [REFLECT]	2018	Ann. Oncol.	https://www.em-base.com/search/re-sults?subaction=viewre-cord&id=L628562644&from=export U2 - L628562644	wrong popu-lation			Round I
Ikeda, M. and Sung, M.W. and Kudo, M. and Koba-yashi, M. and Baron, A.D. and Finn, R.S. and Kaneko, S. and Zhu, A.X. and Ku-bota, T. and Kraljevic, S. and Ishikawa, K. and Siegel, A.B. and Kumada, H. and Okusaka, T.	A Phase 1b trial of lenvatinib plus pembrolizumab in pa-tients with unresectable hepa-tocellular carcinoma	2018	J. Gastroen-terol. Hepa-tol.	https://www.em-base.com/search/re-sults?subaction=viewre-cord&id=L624430867&from=export U2 - L624430867	wrong popu-lation			Round I
Kumar, S. and Purohit, P. and Dagar, S.	A review: Status of genetic modulated nonsmall cell lung cancer targets and treatment (current updates in drugs for non-small cell lung cancer treatment)	2018	Asian J. Pharm. Clin. Res.	https://www.em-base.com/search/re-sults?subaction=viewre-cord&id=L2002541185&from=export U2 - L2002541185	wrong popu-lation			Round I
Shi, M. and Shen, S. and Nie, S. and Yu, Y. and Qian, X. and Kong, B. and Zou, X.	FGFR Signaling Pathway Plays an Important Role in Regulating Pancreatic Acinar-to-Ductal Metaplasia	2018	Gastroenter-ology	https://www.em-base.com/search/re-sults?subaction=viewre-cord&id=L2002432358&from=export U2 - L2002432358	wrong popu-lation			Round I

Ibrahim Mitri, Z. and Parmar, S. and Johnson, B. and Kolodzie, A.K. and Keck, J. and Morris, M. and Guimaraes, A. and Beckett, B. and Borate, U. and Lopez, C.D. and Kemmer, K.A. and Alumkal, J.J. and Beer, T.M. and Corless, C.L. and Mills, G.B. and Bergan, R.C.	Implementing a comprehensive translational oncology platform: From molecular testing to actionability	2018	J. Clin. Oncol.	https://www.embase.com/search/results?subaction=viewrecord&id=L625972498&from=export U2 - L625972498	wrong publication type	10.1200/JCO.2018.36.15-suppl.e14521	Round I
Ikeda, M. and Sung, M.W. and Kudo, M. and Kobayashi, M. and Baron, A.D. and Finn, R.S. and Kaneko, S. and Zhu, A.X. and Kubota, T. and Kraljevic, S. and Ishikawa, K. and Siegel, A.B. and Kumada, H. and Okusaka, T.	A phase 1b trial of lenvatinib (LEN) plus pembrolizumab (PEM) in patients (pts) with unresectable hepatocellular carcinoma (uHCC)	2018	J. Clin. Oncol.	https://www.embase.com/search/results?subaction=viewrecord&id=L625973357&from=export U2 - L625973357	wrong population		Round I
Eberst, L. and Basse, C. and Morelle, M. and Simmet, V. and Terret, C. and Pissaloux, D. and Attignon, V. and Wang, Q. and Tredan, O. and Blay, J.-Y. and Cassier, P.A.	Biomarker selection for patients participating in phase i clinical trials	2018	J. Clin. Oncol.	https://www.embase.com/search/results?subaction=viewrecord&id=L625976564&from=export U2 - L625976564	wrong population		Round I
Goel, G.	Evolution of regorafenib from bench to bedside in colorectal cancer: Is it an attractive option or merely a â€œme tooâ€ drug?	2018	Cancer Manage. Res.	https://www.embase.com/search/results?subaction=viewrecord&id=L621319810&from=export U2 - L621319810	wrong population		Round I
Yuan, T.L. and Amzallag, A. and Bagni, R. and Yi, M. and Afghani, S. and Burgan, W. and Fer, N. and Strathern, L.A. and Powell, K. and Smith, B. and Waters, A.M. and Drubin, D. and Thomson, T. and Liao, R. and Greninger, P. and Stein, G.T. and Murchie, E. and Cortez, E. and Egan, R.K. and Procter, L. and Bess, M. and Cheng, K.T. and Lee, C.-S. and Lee, L.C. and Fellmann, C. and Stephens, R. and Luo, J. and Lowe, S.W. and Benes, C.H. and McCormick, F.	Differential Effector Engagement by Oncogenic KRAS	2018	Cell Rep.	https://www.embase.com/search/results?subaction=viewrecord&id=L620703724&from=export U2 - L620703724	wrong drug	10.1016/j.celrep.2018.01.051	Round I
Schuler, M. and Chan, S.L. and Yen, C. and Lin, C. and Choo, S.P. and Weiss, K. and Geier, A. and Okusaka, T. and Lim, H.Y. and Macarulla, T. and Zhu, A.X. and Kakizume, T. and Gu, Y. and Porta, D.G. and Myers, A.P. and Delord, J.	Phase I/II study of FGF401 in adult patients (pts) with hepatocellular carcinoma (HCC) or solid tumors and positive FGFR4/KLB expression	2017	Oncol. Res. Treat.	https://www.embase.com/search/results?subaction=viewrecord&id=L618607698&from=export U2 - L618607698	wrong population		Round I
Finn, R.S. and Kudo, M. and Cheng, A.-L. and Wyrwicz, L. and Ngan, R. and Blanc, J.-F. and Baron, A.D. and Vogel, A. and Ikeda, M. and Piscaglia, F. and Han, K.-H. and Qin, S. and Minoshima, Y. and Funahashi, Y. and Ren, M. and Dairiki, R. and Sachdev, P. and Tamai, T. and Dutcus, C. and Evans, T.R.J.	Analysis of serum biomarkers (BM) in patients (pts) from a phase 3 study of lenvatinib (LEN) vs sorafenib (SOR) as first-line treatment for unresectable hepatocellular carcinoma (uHCC)	2017	Ann. Oncol.	https://www.embase.com/search/results?subaction=viewrecord&id=L619624061&from=export U2 - L619624061	wrong population		Round I

Ikeda, M. and Sasaki, T. and Morizane, C. and Mizuno, N. and Nagashima, F. and Shimizu, S. and Hayata, N. and Ikezawa, H. and Suzuki, T. and Nakajima, R. and Dutcus, C. and Ueno, M.	A phase 2 study of lenvatinib monotherapy as second-line treatment in unresectable biliary tract cancer: Primary analysis results	2017	Ann. Oncol.	https://www.embase.com/search/results?subaction=viewrecord&id=L619624317&from=export U2 - L619624317	wrong drug	10.1093/annonc/mdx369.106	Round I
Kornmann M and Ishiwata T and Beger HG and Korc M	Fibroblast growth factor-5 stimulates mitogenic signaling and is overexpressed in human pancreatic cancer: evidence for autocrine and paracrine actions.	1997	Oncogene	https://pubmed.ncbi.nlm.nih.gov/9333017/	wrong publication type	10.1038/sj.onc.1201307	Round I
La Rosa S and Uccella S and Erba S and Capella C and Sessa F	Immunohistochemical detection of fibroblast growth factor receptors in normal endocrine cells and related tumors of the digestive system.	2001	Applied immunohistochemistry & molecular morphology : AIMM	https://pubmed.ncbi.nlm.nih.gov/11759058/	wrong population		Round I
Kobrin MS and Yamanaka Y and Friess H and Lopez ME and Korc M	Aberrant expression of type I fibroblast growth factor receptor in human pancreatic adenocarcinomas.	1993	Cancer research	https://pubmed.ncbi.nlm.nih.gov/8402651/	wrong publication type		Round I
Yamazaki K and Nagao T and Yamaguchi T and Saisho H and Kondo Y	Expression of basic fibroblast growth factor (FGF-2)-associated with tumour proliferation in human pancreatic carcinoma.	1997	Virchows Archiv : an international journal of pathology	https://pubmed.ncbi.nlm.nih.gov/9293890/	wrong publication type	10.1007/s004280050074	Round I
Rachagani S and Macha MA and Ponnusamy MP and Haridas D and Kaur S and Jain M and Batra SK	MUC4 potentiates invasion and metastasis of pancreatic cancer cells through stabilization of fibroblast growth factor receptor 1.	2012	Carcinogenesis	https://pubmed.ncbi.nlm.nih.gov/22791819/	wrong publication type	10.1093/carcin/bgs225	Round I
Liu Z and Neiss N and Zhou S and Henne-Bruns D and Korc M and Bachem M and Kornmann M	Identification of a fibroblast growth factor receptor 1 splice variant that inhibits pancreatic cancer cell growth.	2007	Cancer research	https://pubmed.ncbi.nlm.nih.gov/17363592/	wrong publication type	10.1158/0008-5472.CAN-06-3843	Round I
Leung HY and Gullick WJ and Lemoine NR	Expression and functional activity of fibroblast growth factors and their receptors in human pancreatic cancer.	1994	International journal of cancer	https://pubmed.ncbi.nlm.nih.gov/7960240/	wrong population		Round I
Yun Z and Meng F and Li S and Zhang P	Long non-coding RNA CERS6-AS1 facilitates the oncogenicity of pancreatic ductal adenocarcinoma by regulating the microRNA-15a-5p/FGFR1 axis.	2021	Aging	https://pubmed.ncbi.nlm.nih.gov/33581689/	wrong outcomes	10.18632/aging.202540	Round I
Akl L and Abd El-Hafeez AA and Ibrahim TM and Salem R and Marzouk HMM and El-Domany RA and Ghosh P and Eldehna WM and Abou-Seri SM	Identification of novel piperazine-tethered phthalazines as selective CDK1 inhibitors endowed with in vitro anticancer activity toward the pancreatic cancer.	2022	European journal of medicinal chemistry	https://pubmed.ncbi.nlm.nih.gov/36095992/	wrong population		Round I
Otte JM and Schwenger M and Brunke G and Sparmann G and Emmrich J and Schmitz F and FÃ¶lsch UR and Herzig KH	Expression of hepatocyte growth factor, keratinocyte growth factor and their receptors in experimental chronic pancreatitis.	2001	European journal of clinical investigation	https://pubmed.ncbi.nlm.nih.gov/11737224/	wrong outcomes	10.1046/j.1365-2362.2001.00894.x	Round I
Abramovitz L and Rubinek T and Ligumsky H and Bose S and Barshack I and Avivi C and Kaufman B and Wolf I	KL1 internal repeat mediates klotho tumor suppressor activities and inhibits bFGF and IGF-I signaling in pancreatic cancer.	2011	Clinical cancer research : an official journal of the American Association for Cancer Research	https://pubmed.ncbi.nlm.nih.gov/21571866/	wrong drug	10.1158/1078-0432.CCR-10-2749	Round I

Lu H and Liu C and Velazquez R and Wang H and Dunkl LM and Kazic-Legueux M and Haberkorn A and Billy E and Manchado E and Brachmann SM and Moody SE and Engelman JA and Hammerman PS and Caponigro G and Mohseni M and Hao HX	SHP2 Inhibition Overcomes RTK-Mediated Pathway Reactivation in KRAS-Mutant Tumors Treated with MEK Inhibitors.	2019	Molecular cancer therapeutics	https://pubmed.ncbi.nlm.nih.gov/31068384/	wrong drug	10.1158/1535-7163.MCT-18-0852	Round I
BÃ¼chler P and Reber HA and Roth MM and Shiroishi M and Friess H and Hines OJ	Target therapy using a small molecule inhibitor against angiogenic receptors in pancreatic cancer.	2007	Neoplasia (New York, N.Y.)	https://pubmed.ncbi.nlm.nih.gov/17356708/	wrong drug	10.1593/neo.06616	Round I
Ueda J and Matsuda Y and Yamahatsu K and Uchida E and Naito Z and Korc M and Ishiwata T	Epithelial splicing regulatory protein 1 is a favorable prognostic factor in pancreatic cancer that attenuates pancreatic metastases.	2014	Oncogene	https://pubmed.ncbi.nlm.nih.gov/24077287/	wrong publication type	10.1038/onc.2013.392	Round I
Awasthi N and Hinz S and Brekken RA and Schwarz MA and Schwarz RE	Nintedanib, a triple angiokinase inhibitor, enhances cytotoxic therapy response in pancreatic cancer.	2015	Cancer letters	https://pubmed.ncbi.nlm.nih.gov/25527450/	wrong drug	10.1016/j.canlet.2014.12.027	Round I
Zhang H and Lv L and Liu H and Cui L and Chen G and Bi P and Li Z	Profiling the potential biomarkers for cell differentiation of pancreatic cancer using iTRAQ and 2-D LC-MS/MS.	2009	Proteomics. Clinical applications	https://pubmed.ncbi.nlm.nih.gov/21136992/	wrong publication type	10.1002/prca.200800029	Round I
Zuiverloon TC and Boormans JL and Trapman J and van Leenders GJ and Zwarthoff EC	No evidence of FGFR3 mutations in prostate cancer.	2011	The Prostate	https://pubmed.ncbi.nlm.nih.gov/20957671/	wrong population		Round I
Hasegawa Y and Takada M and Yamamoto M and Saitoh Y	The gradient of basic fibroblast growth factor concentration in human pancreatic cancer cell invasion.	1994	Biochemical and biophysical research communications	https://pubmed.ncbi.nlm.nih.gov/8185597/	wrong publication type	10.1006/bbrc.1994.1611	Round I
Fakhar M and Najumuddin and Gul M and Rashid S	Antagonistic role of Klotho-derived peptides dynamics in the pancreatic cancer treatment through obstructing WNT-1 and Frizzled binding.	2018	Biophysical chemistry	https://pubmed.ncbi.nlm.nih.gov/30014891/	wrong drug	10.1016/j.bpc.2018.07.002	Round I
Jonasch E and McCutcheon IE and Waguespack SG and Wen S and Davis DW and Smith LA and Tannir NM and Gombos DS and Fuller GN and Matin SF	Pilot trial of sunitinib therapy in patients with von Hippel-Lindau disease.	2011	Annals of oncology : official journal of the European Society for Medical Oncology	https://pubmed.ncbi.nlm.nih.gov/22105611/	wrong population	10.1093/annonc/mdr011	Round I
Olson DC and Deng C and Hanahan D	Fibroblast growth factor receptor 4, implicated in progression of islet cell carcinogenesis by its expression profile, does not contribute functionally.	1998	Cell growth & differentiation : the molecular biology journal of the American Association for Cancer Research	https://pubmed.ncbi.nlm.nih.gov/9690623/	wrong publication type		Round I
Allen E and Walters IB and Hanahan D	Brivanib, a dual FGF/VEGF inhibitor, is active both first and second line against mouse pancreatic neuroendocrine tumors developing adaptive/evasive resistance to VEGF inhibition.	2011	Clinical cancer research : an official journal of the American Association for Cancer Research	https://pubmed.ncbi.nlm.nih.gov/21622725/	wrong population		Round I

Kutluk Cenik B and Ostapoff KT and Gerber DE and Brekken RA	BIBF 1120 (nintedanib), a triple angiokinase inhibitor, induces hypoxia but not EMT and blocks progression of preclinical models of lung and pancreatic cancer.	2013	Molecular cancer therapeutics	https://pubmed.ncbi.nlm.nih.gov/23729403/	wrong drug	10.1158/1535-7163.MCT-12-0995	Round I
Kornmann M and Ishiwata T and Matsuda K and Lopez ME and Fukahi K and Asano G and Beger HG and Korc M	IIIc isoform of fibroblast growth factor receptor 1 is overexpressed in human pancreatic cancer and enhances tumorigenicity of hamster ductal cells.	2002	Gastroenterology	https://pubmed.ncbi.nlm.nih.gov/12105858/	wrong population		Round I
Cho K and Ishiwata T and Uchida E and Nakazawa N and Korc M and Naito Z and Tajiri T	Enhanced expression of keratinocyte growth factor and its receptor correlates with venous invasion in pancreatic cancer.	2007	The American journal of pathology	https://pubmed.ncbi.nlm.nih.gov/17525264/	wrong publication type	10.2353/ajpath.2007.060935	Round I
Lee SJ and Hong JY and Kim K and Kim KM and Kang SY and Lee T and Kim ST and Park SH and Park YS and Lim HY and Kang WK and Lee J and Park JO	Detection of Fusion Genes Using a Targeted RNA Sequencing Panel in Gastrointestinal and Rare Cancers.	2020	Journal of oncology	https://pubmed.ncbi.nlm.nih.gov/32411236/	wrong publication type	10.1155/2020/4659062	Round I
Deramaudt TB and Takaoka M and Upadhyay R and Bowser MJ and Porter J and Lee A and Rhoades B and Johnstone CN and Weissleder R and Hingorani SR and Mahmood U and Rustgi AK	N-cadherin and keratinocyte growth factor receptor mediate the functional interplay between Ki-RASG12V and p53V143A in promoting pancreatic cell migration, invasion, and tissue architecture disruption.	2006	Molecular and cellular biology	https://pubmed.ncbi.nlm.nih.gov/16705170/	wrong drug	10.1128/MCB.01055-05	Round I
Liu Z and Ishiwata T and Zhou S and Maier S and Henne-Bruns D and Korc M and Bachem M and Kornmann M	Human fibroblast growth factor receptor 1-IIIb is a functional fibroblast growth factor receptor expressed in the pancreas and involved in proliferation and movement of pancreatic ductal cells.	2007	Pancreas	https://pubmed.ncbi.nlm.nih.gov/17632321/	wrong publication type	10.1097/mpa.0b013e318053e7e3	Round I
Yamamoto Y and Matsui J and Matsushima T and Obaishi H and Miyazaki K and Nakamura K and Tohyama O and Semba T and Yamaguchi A and Hoshi SS and Mimura F and Haneda T and Fukuda Y and Kamata JI and Takahashi K and Matsukura M and Wakabayashi T and Asada M and Nomoto KI and Watanabe T and Dezso Z and Yoshimatsu K and Funahashi Y and Tsuruoka A	Lenvatinib, an angiogenesis inhibitor targeting VEGFR/FGFR, shows broad antitumor activity in human tumor xenograft models associated with microvessel density and pericyte coverage.	2014	Vascular cell	https://pubmed.ncbi.nlm.nih.gov/25197551/	wrong publication type	10.1186/2045-824X-6-18	Round I
Chong Y and Thakur N and Paik KY and Lee EJ and Kang CS	Prognostic significance of stem cell/ epithelial-mesenchymal transition markers in periampullary/pancreatic cancers: FGFR1 is a promising prognostic marker.	2020	BMC cancer	https://pubmed.ncbi.nlm.nih.gov/32171280/	wrong publication type	10.1186/s12885-020-6673-2	Round I
Vickers SM and MacMillan-Crow LA and Green M and Ellis C and Thompson JA	Association of increased immunostaining for inducible nitric oxide synthase and nitrotyrosine with fibroblast growth factor transformation in pancreatic cancer.	1999	Archives of surgery (Chicago, Ill. : 1960)	https://pubmed.ncbi.nlm.nih.gov/10088562/	wrong publication type	10.1001/archsurg.134.3.245	Round I
Ogawa T and Takayama K and Takakura N and Kitano S and Ueno H	Anti-tumor angiogenesis therapy using soluble receptors: enhanced inhibition of tumor growth when soluble fibroblast growth factor receptor-1 is used with soluble vascular endothelial growth factor receptor.	2002	Cancer gene therapy	https://pubmed.ncbi.nlm.nih.gov/12136423/	wrong population		Round I

Su Y and Li J and Witkiewicz AK and Brennan D and Neill T and Talarico J and Radice GL	N-cadherin haploinsufficiency increases survival in a mouse model of pancreatic cancer.	2012	Oncogene	https://pubmed.ncbi.nlm.nih.gov/22158044/	wrong drug	10.1038/onc.2011.574	Round I
Zang XP and Lerner M and Brackett D and Pento JT	Influence of KGF on the progression of pancreatic cancer.	2009	Anticancer research	https://pubmed.ncbi.nlm.nih.gov/19661366/	wrong publication type		Round I
Wang L and Lu J and Wu H and Wang L and Liang X and Liang Z and Liu T	Expression of signaling adaptor proteins predicts poor prognosis in pancreatic ductal adenocarcinoma.	2017	Diagnostic pathology	https://pubmed.ncbi.nlm.nih.gov/28558797/	wrong population		Round I
Chen CH and Hsieh TH and Lin YC and Liu YR and Liou JP and Yen Y	Targeting Autophagy by MPT0L145, a Highly Potent PIK3C3 Inhibitor, Provides Synergistic Interaction to Targeted or Chemotherapeutic Agents in Cancer Cells.	2019	Cancers	https://pubmed.ncbi.nlm.nih.gov/31514441/	wrong publication type	10.3390/cancers11091345	Round I
Morikawa Y and Ishihara Y and Tohya K and Kakudo K and Seo MK and Matsuura N	Expression of the fibroblast growth factor receptor-1 in human normal tissues and tumors determined by a new monoclonal antibody.	1996	Archives of pathology & laboratory medicine	https://pubmed.ncbi.nlm.nih.gov/8639054/	wrong publication type		Round I
Huang X and Ding L and Bennewith KL and Tong RT and Welford SM and Ang KK and Story M and Le QT and Giaccia AJ	Hypoxia-inducible mir-210 regulates normoxic gene expression involved in tumor initiation.	2009	Molecular cell	https://pubmed.ncbi.nlm.nih.gov/19782034/	wrong drug	10.1016/j.molcel.2009.09.006	Round I
Griffin RJ and Williams BW and Wild R and Cherrington JM and Park H and Song CW	Simultaneous inhibition of the receptor kinase activity of vascular endothelial, fibroblast, and platelet-derived growth factors suppresses tumor growth and enhances tumor radiation response.	2002	Cancer research	https://pubmed.ncbi.nlm.nih.gov/11912143/	wrong publication type		Round I
Schaap FG and van der Gaag NA and Gouma DJ and Jansen PL	High expression of the bile salt-homeostatic hormone fibroblast growth factor 19 in the liver of patients with extrahepatic cholestasis.	2009	Hepatology (Baltimore, Md.)	https://pubmed.ncbi.nlm.nih.gov/19185005/	wrong outcomes	10.1002/hep.22771	Round I
Petersen LG and StÃ¸rling J and Heding P and Li S and Berezin V and Saldeen J and Billestrup N and Bock E and Mandrup-Poulsen T	IL-1beta-induced pro-apoptotic signalling is facilitated by NCAM/FGF receptor signalling and inhibited by the C3d ligand in the INS-1E rat beta cell line.	2006	Diabetologia	https://pubmed.ncbi.nlm.nih.gov/16718462/	wrong drug	10.1007/s00125-006-0296-2	Round I
Escaffit F and Estival A and Bertrand C and Vaysse N and Hollande E and Clemente F	FGF-2 isoforms of 18 and 22.5 kDa differentially modulate t-PA and PAI-1 expressions on the pancreatic carcinoma cells AR4-2J: consequences on cell spreading and invasion.	2000	International journal of cancer	https://pubmed.ncbi.nlm.nih.gov/10699930/	wrong publication type	10.1002/(sici)1097-0215(20000215)85:4<555::aid-ijc18>3.0.co	Round I
Magdeldin S and Yoshida Y and Li H and Maeda Y and Yokoyama M and Enany S and Zhang Y and Xu B and Fujinaka H and Yaoita E and Sasaki S and Yamamoto T	Murine colon proteome and characterization of the protein pathways.	2012	BioData mining	https://pubmed.ncbi.nlm.nih.gov/22929016/	wrong outcomes	10.1186/1756-0381-5-11	Round I
Marr A and Nissen F and Maisch D and Altmann A and Rana S and Debus J and Huber PE and Haberkorn U and Askoxylakis V	Peptide arrays for development of PDGFRÎ² Affine molecules.	2013	Molecular imaging and biology	https://pubmed.ncbi.nlm.nih.gov/23381728/	wrong drug	10.1007/s11307-013-0616-0	Round I
Martin Huertas R and Fuentes-Mateos R and Serrano Domingo JJ and Corral de la Fuente E and RodrÃguez-Garrote M	Albumin-bound paclitaxel as new treatment for metastatic cholangiocarcinoma: A case report.	2020	World journal of clinical oncology	https://pubmed.ncbi.nlm.nih.gov/33200077/	wrong population		Round I
Shah RN and Ibbitt JC and Alitalo K and Hurst HC	FGFR4 overexpression in pancreatic cancer is mediated by an intronic enhancer activated by HNF1alpha.	2002	Oncogene	https://pubmed.ncbi.nlm.nih.gov/12447688/	wrong publication type	10.1038/sj.onc.1206020	Round I

Mohite P and Lokwani DK and Sakle NS	Exploring the therapeutic potential of SGLT2 inhibitors in cancer treatment: integrating in silico and in vitro investigations.	2024	Naunyn-Schmiedeberg's archives of pharmacology	https://pubmed.ncbi.nlm.nih.gov/38416196/	wrong drug	10.1007/s00210-024-03021-x	Round I
Berger AK and Mughal SS and AllgÃ¤uer M and Springfeld C and Hackert T and Weber TF and Naumann P and Hutter B and Horak P and Jahn A and SchrÃ¶ck E and Haag GM and Apostolidis L and JÃ¤ger D and Stenzinger A and FrÃ¶hling S and Glimm H and Heining C	Metastatic adult pancreatoblastoma: Multimodal treatment and molecular characterization of a very rare disease.	2020	Pancreatology : official journal of the International Association of Pancreatology (IAP) ... [et al.]	https://pubmed.ncbi.nlm.nih.gov/32156527/	wrong population		Round I
Albrecht T and Mehrabi A and Strobel O and Vogel MN and Stenzinger A and Bergmann F and Schirmacher P and Goeppert B	[Spindle and giant cell type undifferentiated carcinoma of the distal bile duct: a case report].	2019	Zeitschrift fur Gastroenterologie	https://pubmed.ncbi.nlm.nih.gov/30641603/	wrong publication type	10.1055/a-0784-8763	Round I
Jiang B and Gu Y and Chen Y	Identification of novel predictive markers for the prognosis of pancreatic ductal adenocarcinoma.	2014	Cancer investigation	https://pubmed.ncbi.nlm.nih.gov/24745611/	wrong publication type	10.3109/07357907.2014.905586	Round I
Takaishi S and Sawada M and Morita Y and Seno H and Fukuzawa H and Chiba T	Identification of a novel alternative splicing of human FGF receptor 4: soluble-form splice variant expressed in human gastrointestinal epithelial cells.	2000	Biochemical and biophysical research communications	https://pubmed.ncbi.nlm.nih.gov/10631118/	wrong population		Round I
Pattison S and Zalcberg JR	Bespoke treatment: drivers beware!	2014	Asia-Pacific journal of clinical oncology	https://pubmed.ncbi.nlm.nih.gov/25155963/	wrong publication type	10.1111/ajco.12265	Round I
ZsÃ¡kai L and NÃ©meth G and SzÃ¡ntai-Kis C and Greff Z and HorvÃ¡th Z and Szokol B and Baska F and Boon TC and Orfi L and KÃ©ri G	[Developing FGFR inhibitors as potential anti-cancer agents].	2013	Acta pharmaceutica Hungarica	https://pubmed.ncbi.nlm.nih.gov/23926649/	wrong publication type		Round I
Ikeda, K. and Kudo, M. and Kawazoe, S. and Osaki, Y. and Ikeda, M. and Okusaka, T. and Tamai, T. and Suzuki, T. and Hisai, T. and Hayato, S. and Okita, K. and Kumada, H.	Phase 2 study of lenvatinib in patients with advanced hepatocellular carcinoma	2017	J. Gastroenterol.	https://www.embase.com/search/results?subaction=viewrecord&id=L612645093&from=export U2 - L612645093	wrong population		Round I
Morizane, C. and Ueno, M. and Sasaki, T. and Nagashima, F. and Mizuno, N. and Shimizu, S. and Hayata, N. and Ikezawa, H. and Suzuki, T. and Nakajima, R. and Dutcus, C.E. and Ikeda, M.	Interim analysis of a phase 2 study of lenvatinib (LEN) monotherapy as second-line treatment in unresectable biliary tract cancer (BTC)	2017	J. Clin. Oncol.	https://www.embase.com/search/results?subaction=viewrecord&id=L618087165&from=export U2 - L618087165	wrong population		Round I
Morizane, C.	Fibroblast growth factor receptors as a potential therapeutic target in patients with intrahepatic cholangiocarcinoma	2016	Ann. Oncol.	https://www.embase.com/search/results?subaction=viewrecord&id=L615048844&from=export U2 - L615048844	wrong population	10.1093/annonc/mdw486	Round I
Meng, X. and Vander Ark, A. and Lee, P. and Hostetter, G. and Bhowmick, N.A. and Matrisian, L.M. and Williams, B.O. and Miranti, C.K. and Li, X.	Myeloid-specific TGF-Î² signaling in bone promotes basic-FGF and breast cancer bone metastasis	2016	Oncogene	https://www.embase.com/search/results?subaction=viewrecord&id=L605654879&from=export U2 - L605654879	wrong publication type	10.1038/onc.2015.297	Round I
Tuxen, I.E.V. and Mau-Soerensen, M. and Yde, C.W. and Joenson, L. and Oestrup, O. and Hasselby, J.P. and Santoni-Rugiu, E. and Lassen, U.N. and Nielsen, F.C.	Actionable targets in recurrent bile duct and pancreatic cancer in a prospective cohort of patients evaluated by whole exome sequencing and SNP array analysis	2016	J. Clin. Oncol.	https://www.embase.com/search/results?subaction=viewrecord&id=L611754873&from=export U2 - L611754873	wrong publication type		Round I

Varchetta, V. and Montagnese, F. and Sibio, S. and Sica, G. and Muscaritoli, M. and Soda, G. and Giordano, G. and Brandi, R. and Tarsi, S. and Mancuso, G. and Mancuso, A.P.	PD-1/PD-L1 expression and regorafenib clinical efficacy on refractory pancreatic cancer patient	2016	J. Clin. Oncol.	https://www.embase.com/search/results?subaction=viewrecord&id=L611756305&from=export U2 - L611756305	wrong drug		Round I
Cidon, E.U. and Alonso, P. and Masters, B.	Markers of response to antiangiogenic therapies in colorectal cancer: Where are we now and what should be next?	2016	Clin. Med. Insights: Oncol.	https://www.embase.com/search/results?subaction=viewrecord&id=L612220816&from=export U2 - L612220816	wrong population	10.4137/CMO.S34542	Round I
Sharif, G.M. and Schmidt, M.O. and Shuptrine, C. and Louis, W.M. and Riegel, A.T. and Wellstein, A.	SMC2 role in regulating tumor angiogenesis via FGF signaling	2016	Cancer Res.	https://www.embase.com/search/results?subaction=viewrecord&id=L613568634&from=export U2 - L613568634	wrong publication type	10.1158/15387445.TUMMET15A15	Round I
Vijayvergia, N. and Cohen, S.J. and Boland, P.M. and Gustafson, K.S. and Sherrif, F. and Cooper, H. and Astaturov, I. and Engstrom, P.F.	Molecular profiling defines subsets of neuroendocrine tumors (NETs) with aggressive disease: A fox chase cancer center (FCCC) study	2016	Pancreas	https://www.embase.com/search/results?subaction=viewrecord&id=L617770764&from=export U2 - L617770764	wrong population	10.1097/MPA.00000000000000615	Round I
Jordan, E. and Basturk, O. and Leach, S.D. and Klimstra, D.S. and Allen, P.J. and Berger, M.F. and Askan, G. and Yu, K.H. and O'Reilly, E.M. and Lowery, M.A.	Assessment of genomic alterations in adenosquamous carcinoma of the pancreas (ASCOP)	2016	J. Clin. Oncol.	https://www.embase.com/search/results?subaction=viewrecord&id=L72224832&from=export U2 - L72224832	wrong population		Round I
Engebretson, A. and Brody, J.R. and Rahib, L. and Matrlslan, L. and Hendifar, A.E. and Hoos, W.A. and Mikhail, S. and Chung, V.M. and Picozzi, V.J. and Heartwell, C. and Mason, K. and Varieur, K. and Madhavan, S. and Petricoin, E. and Pishvaian, M.J.	The Know Your Tumor (KYT) initiative: A national program of multi-omic molecular profiling (MoP) for patients (Pts) with pancreatic cancer (PDA)	2016	J. Clin. Oncol.	https://www.embase.com/search/results?subaction=viewrecord&id=L72224850&from=export U2 - L72224850	wrong population		Round I
Nunes, Q.M. and Li, Y. and Sun, C. and Kinnunen, T.K. and Fernig, D.G.	Fibroblast growth factors as tissue repair and regeneration therapeutics	2016	PeerJ	https://www.embase.com/search/results?subaction=viewrecord&id=L607824000&from=export U2 - L607824000	wrong publication type	10.7717/peerj.1535	Round I
David, W.-C.L.	Editorial look for changes in 2016	2016	Curr. Mol. Med.	https://www.embase.com/search/results?subaction=viewrecord&id=L608745769&from=export U2 - L608745769	wrong publication type		Round I
Iriana, S. and Ahmed, S. and Gong, J. and Annamalai, A.A. and Tuli, R. and Hendifar, A.E.	Targeting mTOR in pancreatic ductal adenocarcinoma	2016	Front. Oncol.	https://www.embase.com/search/results?subaction=viewrecord&id=L610667337&from=export U2 - L610667337	wrong population		Round I
Bekaii-Saab, T. and El-Rayes, B.	Taking aim at the genomic diversity of gastrointestinal cancers: A changing landscape	2016	J. Gastrointest. Oncol.	https://www.embase.com/search/results?subaction=viewrecord&id=L613007652&from=export U2 - L613007652	wrong publication type	10.21037/jgo.2016.09.09	Round I
Qi, L. and Song, W. and Li, L. and Cao, L. and Yu, Y. and Song, C. and Wang, Y. and Zhang, F. and Li, Y. and Zhang, B. and Cao, W.	FGF4 induces epithelial-mesenchymal transition by inducing store-operated calcium entry in lung adenocarcinoma	2016	Oncotarget	https://www.embase.com/search/results?subaction=viewrecord&id=L613339186&from=export U2 - L613339186	wrong population	10.18632/oncotarget.12187	Round I

Rahib, L. and Engebretson, A. and Pishvaian, M.J. and Brody, J.R. and Hoos, W.A. and Lyons, E.E. and Bender, J. and Petricoin, E.F. and Madhavan, S. and Heartwell, C. and Matrisian, L.M.	Molecular profiling of pancreatic cancer patients from a wide geographical distribution across the US	2016	Cancer Res.	https://www.embase.com/search/results?subaction=viewrecord&id=L613611215&from=export U2 - L613611215	wrong publication type	10.1158/1538-7445.AM2016-93	Round I
Forest, A. and Nakasone, S. and Wang, Y. and Guo, X. and Mack, T.R. and Zhao, G. and Yue, Y.G. and Gong, X. and Stewart, T. and Buchanan, S. and Reinhard, C. and Novosiadly, R.	Beta-klotho expression is associated with the antitumor activity of pan-FGFR inhibitor in human malignancies with FGF19 amplification	2015	Mol. Cancer Ther.	https://www.embase.com/search/results?subaction=viewrecord&id=L72186138&from=export U2 - L72186138	wrong outcome		Round I
Davies, B.R. and Guan, N. and Logie, A. and Crafter, C. and Hanson, L. and Jacobs, V. and James, N. and Dudley, P. and Jacques, K. and Ladd, B. and D'Cruz, C.M. and Zinda, M. and Lindemann, J. and Kodaira, M. and Tamura, K. and Jenkins, E.L.	Tumors with AKT1E17K mutations are rational targets for single agent or combination therapy with AKT inhibitors	2015	Mol. Cancer Ther.	https://www.embase.com/search/results?subaction=viewrecord&id=L608241320&from=export U2 - L608241320	wrong drug	10.1158/1535-7163.MCT-15-0230	Round I
Ichikawa, K. and Miyano, S.W. and Adachi, Y. and Yamamoto, Y. and Ozawa, Y. and Funahashi, Y. and Okamoto, K. and Nomoto, K. and Matsui, J.	Lenvatinib, Tri-specific targeted therapy to VEGFR/FGFR/RET, suppresses angiogenesis through the inhibition of both VEGFR and FGFR signaling pathways	2015	Cancer Res.	https://www.embase.com/search/results?subaction=viewrecord&id=L72191950&from=export U2 - L72191950	wrong publication type	10.1158/1538-7445.AM2015-1374	Round I
Mondejar, T. and Hernadez-Agudo, E. and Soto-Montenegro, M. and Megias, D. and Cebrian, D. and Sanchez, J. and Mulero, F. and Colomer, R. and Hidalgo, M. and Desco, M. and Quintela-Fandino, M.	18F-misonidazole PET (FMISO-PET) monitors vascular normalization (VN) and predicts benefit from antiangiogenic treatment plus chemotherapy in pancreas cancer	2015	Cancer Res.	https://www.embase.com/search/results?subaction=viewrecord&id=L72192067&from=export U2 - L72192067	wrong drug	10.1158/1538-7445.AM2015-1493	Round I
Itamochi, H. and Oumi, N. and Oishi, T. and Taniguchi, F. and Shoji, T. and Fujiwara, H. and Sugiyama, T. and Suzuki, M. and Kigawa, J. and Harada, T.	Fibroblast growth factor receptor 2 is associated with poor overall survival in clear cell carcinoma of the ovary and may be a novel therapeutic approach	2015	Cancer Res.	https://www.embase.com/search/results?subaction=viewrecord&id=L72194050&from=export U2 - L72194050	wrong population	10.1158/1538-7445.AM2015-3556	Round I
Arnett, A.L.H. and Chang, K. and Sio, T.T. and Miller, R.J.	Mutational analysis by next generation sequencing in patients with biliary and pancreatic adenocarcinoma	2015	Radiother. Oncol.	https://www.embase.com/search/results?subaction=viewrecord&id=L71961236&from=export U2 - L71961236	wrong outcomes		Round I
Van Der Ark, A. and Lee, P. and Hostetter, G. and Bhowmick, N.A. and Matrisian, L.M. and Williams, B.O. and Miranti, C.K. and Li, X.	Osteoclast TGF-Î² signaling-mediated basic-FGF promotes breast cancer bone metastasis	2015	J. Bone Miner. Res.	https://www.embase.com/search/results?subaction=viewrecord&id=L620770127&from=export U2 - L620770127	wrong population	10.1002/jbmr.2763	Round I
Alzahrani, A.S. and Almohanna, M. and Al-Hindi, H. and Qasem, E. and Almehthel, M.	Low frequency of BRAFV600E and TERT promotor mutations in adrenocortical tumors and lack of association of FGFR4 G388R polymorphism with adrenocortical cancer occurrence and histopathological score	2015	Endocr. Rev.	https://www.embase.com/search/results?subaction=viewrecord&id=L613816259&from=export U2 - L613816259	wrong population		Round I
Tian, F.	Cell line genomic DNAs for molecular diagnosis of cancer	2014	J. Mol. Diagn.	https://www.embase.com/search/results?subaction=viewrecord&id=L71660075&from=export U2 - L71660075	wrong publication type		Round I

Giacomini, A. and Di Salle, E. and Coltrini, D. and Rezzola, S. and Belleri, M. and Presta, M. and Ronca, R.	Stromal delivery of long Pentraxin-3 impairs FGF/FGFR-dependent tumor growth and metastasis	2014	Eur. J. Cancer	https://www.embase.com/search/results?subaction=viewrecord&id=L71734383&from=export U2 - L71734383	wrong publication type		Round I
Ishiwata, T. and Yoshimura, H. and Matsuda, Y. and Murase, M. and Suzuki, T. and Kawamoto, Y. and Kawahara, K. and Ishiwata, S. and Naito, Z.	Decrease in fibroblast growth factor receptor-4 levels inhibited the proliferation, migration, and invasion of pancreatic cancer	2014	Pancreas	https://www.embase.com/search/results?subaction=viewrecord&id=L71837580&from=export U2 - L71837580	wrong drug	10.1097/MPA.00000000000231	Round I
Schatz, C.A. and Kopitz, C. and Wittemer-Rump, S. and Sommer, A. and Lindbom, L. and Osada, M. and Yamanouchi, H. and Huynh, H. and Krahn, T. and Asadullah, K.	Pharmacodynamic and stratification biomarker for the anti-FGFR2 antibody (BAY1179470) and the FGFR2-ADC	2014	Cancer Res.	https://www.embase.com/search/results?subaction=viewrecord&id=L71719744&from=export U2 - L71719744	wrong population	10.1158/1538-7445.AM2014-4766	Round I
Volpe, L.A. and Bal, A. and Foulke, J. and Jackson, M. and Chen, L. and Tian, F.	Understanding the molecular nature of cancer cell lines	2014	Cancer Res.	https://www.embase.com/search/results?subaction=viewrecord&id=L71722802&from=export U2 - L71722802	wrong publication type	10.1158/1538-7445.AM2014-3920	Round I
Baschnagel, A.M. and Stone, B.M. and Thibodeau, B.J. and Fortier, L.E. and Ahmed, S. and Geddes, T.J. and Ketelsen, B.E. and Pruetz, B.L. and Jury, R.P. and Wilson, G.D.	The effects of neoadjuvant chemoradiation on gene expression patterns of pancreatic ductal adenocarcinoma	2014	Int. J. Radiat. Oncol. Biol. Phys.	https://www.embase.com/search/results?subaction=viewrecord&id=L71624087&from=export U2 - L71624087	wrong drug		Round I
Rolfo, C. and Raez, L.E. and Bronte, G. and Santos, E.S. and Papadimitriou, K. and Buffoni, L. and Van Meerbeeck, J.P. and Russo, A.	BIBF 1120/nintedanib: A new triple angiokinase inhibitor-directed therapy in patients with non-small cell lung cancer	2013	Expert Opin. Invest. Drugs	https://www.embase.com/search/results?subaction=viewrecord&id=L369334267&from=export U2 - L369334267	wrong population	10.1517/13543784.2013.812630	Round I
Rusman, Y. and Kawakami, Y. and Spike, A. and Bagchi, A. and Salomon, C.E.	Limb regeneration in zebrafish as a discovery tool for modulators of receptor tyrosine kinase signaling in cancer	2013	Planta Med.	https://www.embase.com/search/results?subaction=viewrecord&id=L71354059&from=export U2 - L71354059	wrong drug	10.1055/s-0033-1348515	Round I
Cunningham, I. and Shiomi, T. and D'Armiento, J.M. and Rui, H. and Cordon-Cardo, C. and Hamele-Bena, D.	Hiding from chemo: Leukemic cells forming tumors to survive	2013	Cancer Res.	https://www.embase.com/search/results?subaction=viewrecord&id=L71348385&from=export U2 - L71348385	wrong publication type	10.1158/1538-7445.AM2013-1096	Round I
Pavel, M.	Translation of molecular pathways into clinical trials of neuroendocrine tumors	2013	Neuroendocrinology	https://www.embase.com/search/results?subaction=viewrecord&id=L51962340&from=export U2 - L51962340	wrong publication type	10.1159/000336089	Round I
Richard, S. and Gardie, B. and CouvÃ©, S. and Gad, S.	Von Hippel-Lindau: How a rare disease illuminates cancer biology	2013	Semin. Cancer Biol.	https://www.embase.com/search/results?subaction=viewrecord&id=L52073716&from=export U2 - L52073716	wrong outcomes	10.1016/j.semcancer.2012.05.005	Round I
Kallakury, B.V.S. and Al-Rohil, R.N. and Presta, M.J. and Sheehan, G.M. and Sheehan, C.E. and Boguniewicz, A.B. and Ross, J.S.	Prognostic significance of fibroblast growth factor receptor 2 (FGFR-2) overexpression in invasive mammary carcinoma (BRCA)	2013	Lab. Invest.	https://www.embase.com/search/results?subaction=viewrecord&id=L70994871&from=export U2 - L70994871	wrong outcome		Round I
Zhou, Y. and Chen, Y. and Tong, L. and Xie, H. and Wen, W. and Zhang, J. and Xi, Y. and Shen, Y. and Geng, M. and Wang, Y. and Jiang, H. and Luo, C. and Lin, L. and Ding, J.	AL3810, a multi-tyrosine kinase inhibitor, exhibits potent anti-angiogenic and anti-tumour activity via targeting VEGFR, FGFR and PDGFR	2012	J. Cell. Mol. Med.	https://www.embase.com/search/results?subaction=viewrecord&id=L365729031&from=export U2 - L365729031	wrong population	10.1111/j.1582-4934.2012.01541.x	Round I

Takahashi, I. and Sato, T. and Sato, F. and Ohashi, K. and Nata, K.	Involvement of heparan sulfate 6-O-sulfotransferase isoform-1 in the mouse beta cell proliferation during pregnancy	2012	Diabetologia	https://www.embase.com/search/results?subaction=viewrecord&id=L70888447&from=export U2 - L70888447	wrong publication type	10.1007/s00125-012-2688-9	Round I
Ishiwata, T. and Yoshimura, H. and Korc, M. and Matsuda, Y.	Inhibitory effects of fibroblast growth factor receptor 2 in pancreatic cancer	2012	Pancreas	https://www.embase.com/search/results?subaction=viewrecord&id=L71125991&from=export U2 - L71125991	wrong publication type	10.1097/MPA.0b013e31826a15f2	Round I
Ueda, J. and Matsuda, Y. and Yamahatsu, K. and Uchida, E. and Naito, Z. and Ishiwata, T.	Epithelial splicing regulatory protein 1 modulates cell growth, migration, and invasion of human pancreatic ductal adenocarcinoma	2012	Cancer Res.	https://www.embase.com/search/results?subaction=viewrecord&id=L71089797&from=export U2 - L71089797	wrong publication type	10.1158/1538-7445.AM2012-2412	Round I
Askoxylakis, V. and Marr, A. and Markert, A. and Altmann, A. and Rana, S. and Debus, J. and Haberkorn, U.	Peptide arrays for optimization of the properties of a new PDGFRÎ² binding peptide	2012	Mol. Imaging Biol.	https://www.embase.com/search/results?subaction=viewrecord&id=L70788778&from=export U2 - L70788778	wrong publication type	10.1007/s11307-012-0543-5	Round I
Rachagani, S. and MacHa, M.A. and Ponnusamy, M.P. and Chakraborty, S. and Batra, S.K.	MUC4 induces epithelial mesenchymal transition through N-cadherin upregulation in pancreatic cancer	2011	Pancreas	https://www.embase.com/search/results?subaction=viewrecord&id=L70567683&from=export U2 - L70567683	wrong publication type	10.1097/MPA.0b013e318232ea83	Round I
Sharp, M. and Sadis, S. and Rhodes, D. and Thompson, N. and Otte, M. and Alvarez, J. and King, P. and Winkler, H. and Platero, S.	Identification of predictive and pharmacodynamic biomarkers for an FGFR inhibitor	2011	Mol. Cancer Ther.	https://www.embase.com/search/results?subaction=viewrecord&id=L70609291&from=export U2 - L70609291	wrong publication type	10.1158/1535-7163.TARG-11-C12	Round I
Furuse, J. and Sasaki, Y. and Okusaka, T. and Ikeda, M. and Nagashima, F. and Sunakawa, Y. and Ueno, H. and Nakachi, K. and Hashizume, K. and Ito, Y.	Phase I study to assess the safety, tolerability and pharmacokinetics of the multikinase inhibitor regorafenib (bay 73-4506) in Japanese patients with advanced solid tumours	2011	Eur. J. Cancer	https://www.embase.com/search/results?subaction=viewrecord&id=L70548447&from=export U2 - L70548447	wrong outcome		Round I
Lehnen, N.C. and Perner, S. and SchÃ¼tte, U. and Feldmann, G. and GÃ¼tgemann, I.	FGFR1 amplifications in pancreatic cancer	2011	Onkologie	https://www.embase.com/search/results?subaction=viewrecord&id=L70569210&from=export U2 - L70569210	wrong publication type	10.1159/000333303	Round I
Coleman, S.J. and Myrto-Chioni, A. and Grose, R.P. and Kocher, H.	Investigating the role of FGF signaling in pancreatic cancer	2011	Gastroenterology	https://www.embase.com/search/results?subaction=viewrecord&id=L70412268&from=export U2 - L70412268	wrong publication type	10.1016/S0016-5085(11)62965-X	Round I
Nassiri, M. and Ramos, S. and Jorda, M.	FGFR3 transcript and protein down-regulation is a negative prognostic factor and correlates with promoter methylation in pancreatic islet cell tumors	2011	Lab. Invest.	https://www.embase.com/search/results?subaction=viewrecord&id=L70424520&from=export U2 - L70424520	wrong outcome		Round I
Shimizu, T. and Tolcher, A.W. and Patnaik, A. and Papadopoulos, K. and Christensen, O. and Lin, T. and Blumenschein, G.R.	Phase I dose-escalation study of continuously administered regorafenib (BAY 73-4506), an inhibitor of oncogenic and angiogenic kinases, in patients with advanced solid tumors	2010	J. Clin. Oncol.	https://www.embase.com/search/results?subaction=viewrecord&id=L70258775&from=export U2 - L70258775	wrong outcome		Round I
Ishiwata, T. and Matsuda, Y. and Kawahara, K. and Yamamoto, T. and Teduka, K. and Uchida, E. and Korc, M. and Naito, Z.	Fibroblast growth factor receptor (FGFR) 2 IIIc, is a novel molecular target for suppressing pancreatic cancer cell growth and migration	2010	Cancer Res.	https://www.embase.com/search/results?subaction=viewrecord&id=L71452323&from=export U2 - L71452323	wrong publication type	10.1158/1538-7445.AM10-4101	Round I

Allen, E. and Walters, I.B. and Rivera, I.C. and Hanahan, D.	Anti-angiogenic therapy using brivanib, a combined VEGF and FGF pathway inhibitor, in a mouse model of pancreatic neuroendocrine cancer (PNET), results in sustained vascular blockade, without evidence for evasive/acquired resistance in the form of VEGF-independent revascularization, in contrast to other VEGF inhibitors	2009	Mol. Cancer Ther.	https://www.embase.com/search/results?subaction=viewrecord&id=L70266021&from=export U2 - L70266021	wrong outcome		Round I
Ramsay, A.K. and Leung, H.Y.	Signalling pathways in prostate carcinogenesis: Potentials for molecular-targeted therapy	2009	Clin. Sci.	https://www.embase.com/search/results?subaction=viewrecord&id=L355321276&from=export U2 - L355321276	wrong publication type	10.1042/CS20080391	Round I
Serra, S. and Ezzat, S. and Chetty, R. and Asa, S.L.	The role FGFR4 in pancreatic endocrine tumors	2009	Lab. Invest.	https://www.embase.com/search/results?subaction=viewrecord&id=L70116739&from=export U2 - L70116739	wrong outcome		Round I
Ishiwata, T. and Kawamoto, M. and Kawamoto, Y. and Teduka, K. and Matsuda, Y. and Onda, M. and Uchida, E. and Tajiri, T. and Korc, M. and Naito, Z.	Over-expression of FGFR2 IIIC isoform enhances the growth of pancreatic cancer	2008	Pancreas	https://www.embase.com/search/results?subaction=viewrecord&id=L70033705&from=export U2 - L70033705	wrong publication type	10.1097/01.MPA.0000335480.46603.d9	Round I
Nomura, S. and Yoshitomi, H. and Takano, S. and Shida, T. and Kobayashi, S. and Ohtsuka, M. and Kimura, F. and Shimizu, H. and Yoshidome, H. and Kato, A. and Miyazaki, M.	FGF10/FGFR2 signal induces cell migration and invasion in pancreatic cancer	2008	Br. J. Cancer	https://www.embase.com/search/results?subaction=viewrecord&id=L50194639&from=export U2 - L50194639	wrong publication type	10.1038/sj.bjc.6604473	Round I
Li, H.-J. and Pang, Z.-L. and Mai-Mai-Ti, M.-L.-A.-S.	Effects of vascular endothelial growth factor antisense oligodeoxynucleotide on the mRNA expression of vascular endothelial growth factor, fms-like tyrosine kinase-1 and kinase insert domain-containing receptor and vascular endothelial growth factor protein excretion of gallbladder carcinoma GBC-SD cells in vitro	2007	World Chin. J. Dig.	https://www.embase.com/search/results?subaction=viewrecord&id=L47104483&from=export U2 - L47104483	wrong outcome		Round I
Wang, H. and Iezzi, M. and Theander, S. and Antinozzi, P.A. and Gauthier, B.R. and Halban, P.A. and Wollheim, C.B.	Suppression of Pdx-1 perturbs proinsulin processing, insulin secretion and GLP-1 signalling in INS-1 cells	2005	Diabetologia	https://www.embase.com/search/results?subaction=viewrecord&id=L40591228&from=export U2 - L40591228	wrong drug	10.1007/s00125-005-1692-8	Round I
Cohen Jr., M.M.	Neoplasms associated with alterations in fibroblast growth factor receptors	2003	Am. J. Med. Genet.	https://www.embase.com/search/results?subaction=viewrecord&id=L37063877&from=export U2 - L37063877	wrong publication type	10.1002/ajmg.a.10058	Round I
Alitalo, K.	The fatal detachment	2001	Nature Cell Biol.	https://www.embase.com/search/results?subaction=viewrecord&id=L32624409&from=export U2 - L32624409	wrong publication type	10.1038/35083104	Round I
Compagni, A. and Wilgenbus, P. and Impagnatiello, M.-A. and Cotten, M. and Christofori, G.	Fibroblast growth factors are required for efficient tumor angiogenesis	2000	Cancer Res.	https://www.embase.com/search/results?subaction=viewrecord&id=L32059199&from=export U2 - L32059199	wrong publication type		Round I

Ishiwata, T. and Friess, H. and BÃ¼chler, M.W. and Lopez, M.E. and Korc, M.	Characterization of keratinocyte growth factor and receptor expression in human pancreatic cancer	1998	Am. J. Pathol.	https://www.embase.com/search/results?subaction=viewrecord&id=L28317524&from=export U2 - L28317524	wrong publication type	10.1016/S0002-9440(10)65562-9	Round I
Alsharif, Shaker	Development of novel implantable hydrogels and aerogels for localised cancer treatment	2023		https://search.ebscohost.com/login.aspx?direct=true&db=ddu&AN=ddu.oai.ethos.bl.uk.888562&site=ehost-live	wrong outcome		Round I
Render, Jasmine	Investigating the effects of varying levels of EB2 expression on migration in cancer cells	2022		https://search.ebscohost.com/login.aspx?direct=true&db=ddu&AN=ddu.oai.ethos.bl.uk.884046&site=ehost-live	wrong outcome		Round I
Shah, Riyaz Nazeerul Haq	The transcriptional mechanisms of fibroblast growth factor receptor type 4 overexpression in pancreatic carcinoma	2002		https://search.ebscohost.com/login.aspx?direct=true&db=ddu&AN=ddu.oai.ethos.bl.uk.271806&site=ehost-live	wrong outcome		Round I
Coleman, Stacey J.	The role of nuclear FGFR1 and FGF2 in pancreatic cancer	2013		https://search.ebscohost.com/login.aspx?direct=true&db=ddu&AN=ddu.oai.ethos.bl.uk.664959&site=ehost-live	wrong outcome		Round I
Ã˜ien, Karin A.	Profiling, comparison and validation of gene expression in gastric carcinoma and normal stomach	2002		https://search.ebscohost.com/login.aspx?direct=true&db=ddu&AN=ddu.oai.ethos.bl.uk.272870&site=ehost-live	wrong outcome		Round I
NCT03827850	FGFR Inhibitor in FGFR Dysregulated Cancer		clinical-trial.gov registry				Round II
NCT04972253	Phase I BLASST-3 Trial		clinical-trial.gov registry				Round II
NCT05522309	FGFR Inhibitor ET0111 Monotherapy in Patients With Advanced Solid Tumors		clinical-trial.gov registry				Round II
NCT01976741	Dose Escalation Pan-FGFR (Fibroblast Growth Factor Receptor) Inhibitor (Rogaratinib)		clinical-trial.gov registry				Round II
NCT02052778	A Study of TAS-120 in Patients With Advanced Solid Tumors		clinical-trial.gov registry				Round II
NCT04258527	Phase 1 Study of Pemigatinib in Patients With Advanced Malignancies With FGF/FGFR Alterations		clinical-trial.gov registry				Round II
NCT05173142	HMPL-453 (FGFR Inhibitor) in Combination With Chemotherapy or Anti-PD-1 Antibody in Advanced Solid Tumors		clinical-trial.gov registry				Round II
NCT04439240	Testing AZD4547 as a Potential Targeted Treatment in Cancers With FGFR Genetic Changes (MATCH-Subprotocol W)		clinical-trial.gov registry				Round II
NCT02575508	Pan FGFR Kinase Inhibitor BGJ398 and Combination Chemotherapy in Treating Patients With Untreated Metastatic Pancreatic Cancer		clinical-trial.gov registry				Round II
NCT01283945	Study of Oral Lucitanib (E-3810), a Dual VEGFR-FGFR Tyrosine Kinase Inhibitor, in Patients With Solid Tumors		clinical-trial.gov registry				Round II
NCT01752920	Phase 1/2 Study of Derazantinib (ARQ 087) in Adult Subjects With Advanced Solid Tumors With FGFR Genetic Alterations		clinical-trial.gov registry				Round II

NCT02529553	A Study of LY3076226 in Participants With Advanced or Metastatic Cancer		clinical-trial.gov registry				Round II
NCT03822117	Efficacy and Safety of Pemigatinib in Previously Treated Locally Advanced/Metastatic or Surgically Unresectable Solid Tumor Malignancies Harboring Activating FGFR Mutations or Translocations (FIGHT-207)		clinical-trial.gov registry				Round II
NCT03517956	Phase 1 Study of the Combination of Rogaratinib With Copanlisib in Patients With Fibroblast Growth Factor Receptor (FGFR)-Positive, Locally Advanced or Metastatic Solid Tumors		clinical-trial.gov registry				Round II
NCT02038673	An Open-label Phase I Study of Orally Available Novel Small-molecule Fibroblast Growth Factor Receptors (FGFR) 1,2,3 and 4 Inhibitor, ASP5878 at Single and Multiple Doses in Patients With Solid Tumors		clinical-trial.gov registry				Round II
NCT03235570	A Safety and Tolerability Study of Pemigatinib in Japanese Subjects With Advanced Malignancies - (FIGHT-102)		clinical-trial.gov registry				Round II
NCT02393248	Open-Label, Dose-Escalation Study of Pemigatinib in Subjects With Advanced Malignancies - (FIGHT-101)		clinical-trial.gov registry				Round II
NCT00789633,	Masitinib in Combination With Gemcitabine for Treatment of Patients With Advanced/Metastatic Pancreatic Cancer	2008	clinical-trial.gov (protocol cochrane)	https://www.cochranelibrary.com/central/doi/10.1002/central/CN-02043017/full		wrong drug	Round II
Xiao, WJ and Chen, XA and Yang, L and Mao, YQ and Wei, YQ and Chen, LJ	Co-delivery of doxorubicin and plasmid by a novel FGFR-mediated cationic liposome	2010	INTERNATIONAL JOURNAL OF PHARMACEUTICS		10.1016/j.ijpharm.2010.04.018	wrong drug	Round II
Burman, DR and Das, S and Das, C and Bhattacharya, R	Alternative splicing modulates cancer aggressiveness: role in EMT/metastasis and chemoresistance	2021	MOLECULAR BIOLOGY REPORTS		10.1007/s11033-020-06094-y	wrong population	Round II
Lang, S and Taeger, J and Moser, C and Hack, C and Schlitt, H and Geissler, E and Stoeltzing, O	Inhibition of FGFR/PDGFR /VEGFR signaling reduces pancreatic cancer growth by direct effects on tumor cells, endothelial cells and pericytes	2009	CANCER RESEARCH			wrong population	Round II
Fu, X.-F. and Zhao, H.-C. and Yang, C.-L. and Chen, C.-Z. and Wang, K. and Gao, F. and Tian, Y.-Z. and Zhao, H.-L.	MicroRNA-203-3p inhibits the proliferation, invasion and migration of pancreatic cancer cells by downregulating fibroblast growth factor 2	2021	Oncology Letters	https://www.scopus.com/inward/record.uri?eid=2-s2.0-85109105323&doi=10.3892%2fol.2021.12887&partnerID=40&md5=6f1e1be6254264962e8f5a45dda08e3a		wrong drug	Round II
Fang, D. and Zhang, C. and Xu, P. and Liu, Y. and Mo, X. and Sun, Q. and Abdelatty, A. and Hu, C. and Xu, H. and Zhou, G. and Xia, H. and Lan, L.	S100A16 promotes metastasis and progression of pancreatic cancer through FGF19-mediated AKT and ERK1/2 pathways	2021	Cell Biology and Toxicology	https://www.scopus.com/inward/record.uri?eid=2-s2.0-85098740448&doi=10.1007%2fs10565-020-09574-w&partnerID=40&md5=cb85ce06213dd5c923becb89f0f85b45		wrong outcome	Round II
Kato, S.	Tumour-agnostic therapy for pancreatic cancer and biliary tract cancer	2021	Diagnostics	https://www.scopus.com/inward/record.uri?eid=2-s2.0-85108782535&doi=10.3390%2fdiagnostics11020252&partnerID=40&md5=2434d70331d0ba84f4b236fa925f9eea		wrong population	Round II

Singhi, A.D. and George, B. and Greenbowe, J.R. and Chung, J. and Suh, J. and Maitra, A. and Klempner, S.J. and Hendifar, A. and Milind, J.M. and Golan, T. and Brand, R.E. and Zureikat, A.H. and Roy, S. and Schrock, A.B. and Miller, V.A. and Ross, J.S. and Ali, S.M. and Bahary, N.	Real-Time Targeted Genome Profile Analysis of Pancreatic Ductal Adenocarcinomas Identifies Genetic Alterations That Might Be Targeted With Existing Drugs or Used as Biomarkers	2019	Gastroenterology	https://www.scopus.com/inward/record.uri?eid=2-s2.0-85064216307&doi=10.1053%2fj.gastro.2019.02.037&partnerID=40&md5=57b31cff4eb79b8a634510d16583d0e3		wrong outcome	Round II
Lai, E. and Puzzoni, M. and Ziranu, P. and Pretta, A. and Impera, V. and Mariani, S. and Liscia, N. and Soro, P. and Musio, F. and Persano, M. and Donisi, C. and Tolu, S. and Balconi, F. and Pireddu, A. and Demurtas, L. and Pusceddu, V. and Camera, S. and Sclafani, F. and Scartozzi, M.	New therapeutic targets in pancreatic cancer	2019	Cancer Treatment Reviews	https://www.scopus.com/inward/record.uri?eid=2-s2.0-85074978410&doi=10.1016%2fj.ctrv.2019.101926&partnerID=40&md5=7560d1412bd6cb0a88b3dac679fc73e7		wrong outcome	Round II
Ma, W.W. and Xie, H. and Fetterly, G. and Pitzonka, L. and Whitworth, A. and Levea, C. and Wilton, J. and Mantione, K. and Schihl, S. and Dy, G.K. and Boland, P. and Iyer, R. and Tan, W. and Brady, W. and Straubinger, R.M. and Adjei, A.A.	A Phase Ib Study of the FGFR/VEGFR Inhibitor Dovitinib with Gemcitabine and Capecitabine in Advanced Solid Tumor and Pancreatic Cancer Patients	2019	American Journal of Clinical Oncology: Cancer Clinical Trials	https://www.scopus.com/inward/record.uri?eid=2-s2.0-85056549699&doi=10.1097%2fCOC.0000000000000492&partnerID=40&md5=7cc060c11a33fa400423bf38949d9aaa		wrong drug	Round II
Mardhian, D.F. and Vrynas, A. and Storm, G. and Bansal, R. and Prakash, J.	FGF2 engineered SPIONs attenuate tumor stroma and potentiate the effect of chemotherapy in 3D heterospheroidal model of pancreatic tumor	2020	Nanotheranostics	https://www.scopus.com/inward/record.uri?eid=2-s2.0-85077564535&doi=10.7150%2fntno.38092&partnerID=40&md5=77bfbeccb67ffb72e02c29b519cb68a8		wrong drug	Round II
Costa, R. and Carneiro, B.A. and Taxter, T. and Tavora, F.A. and Kalyan, A. and Pai, S.A. and Chae, Y.K. and Giles, F.J.	FGFR3-TACC3 fusion in solid tumors: Mini review	2016	Oncotarget	https://www.scopus.com/inward/record.uri?eid=2-s2.0-84983487456&doi=10.18632%2foncotarget.10482&partnerID=40&md5=b08c82bae4aed20ee1c7007b681b9fc6		wrong population	Round II
Egeli, U. and Tezcan, G. and Cecener, G. and Tunca, B. and Sevinc, E.D. and Kaya, E. and Ak, S. and Dundar, H.Z. and Sarkut, P. and Ugras, N. and Yerci, O. and Ozen, Y. and Evrensel, T.	MiR-216b targets FGFR1 and confers sensitivity to radiotherapy in pancreatic ductal adenocarcinoma patients without EGFR or KRAS mutation	2016	Pancreas	https://www.scopus.com/inward/record.uri?eid=2-s2.0-84964319199&doi=10.1097%2fMPA.0000000000000640&partnerID=40&md5=da056821c1c90c60a34ac40226020d83		Wrong study type	Round II
Liu, S.-X. and Xia, Z.-S. and Zhong, Y.-Q.	Gene therapy in pancreatic cancer	2014	World Journal of Gastroenterology	https://www.scopus.com/inward/record.uri?eid=2-s2.0-84910014382&doi=10.3748%2fwjg.v20.i37.13343&partnerID=40&md5=bf76be4b79dca4aa6dfa6eb8e5b51226		wrong population	Round II
Zhu, J. and Song, Z. and Zhao, Y. and Zhang, J. and Li, Y. and Li, D. and Schneider, R. and Ding, Y. and Sun, Z. and Qin, Y. and Chang, Z.-W. and Wang, Z. and Zhang, Z. and Zhang, P. and Wang, Y.	45O First-in-human study of ABSK061: A selective fibroblast growth factor receptor (FGFR) 2/3 inhibitor for treating patients with advanced solid tumors	2024	ESMO Open	https://www.embase.com/search/results?subaction=viewrecord&id=L2030487337&from=export U2 - L2030487337	10.1016/j.esmoop.2024.102274	wrong population	Round II
Zhang, D.-S. and Liu, F.-R. and Lu, Y.-X. and Bai, B and Zhang, Y. and Wang, Z.-Q. and Liu, Y. and Chen, J.-W. and Wang, F. and Xu, R.-H.	Preliminary results of a phase II study of surufatinib plus sintilimab, nab-paclitaxel and gemcitabine (AG) as first-line therapy in patients (pts) with locally advanced or metastatic pancreatic adenocarcinoma (mPDAC)	2024	J. Clin. Oncol.	https://www.embase.com/search/results?subaction=viewrecord&id=L643534528&from=export U2 - L643534528		Updates of previous reported study or abstract	Round II

Pant, S. and Arnold, D. and Tabernero, J. and Loriot, Y. and Folprecht, G. and Haag, G.M. and Palmer, D. and Prenen, H. and Coward, J. and Lugowska, I. and Goeminne, J.-C. and Cervantes, A. and Gutierrez, M. and Sweiti, H. and Hammond, C. and Najmi, S. and Thomas, S. and Triantos, S. and Crow, L. and Schuler, M.H.H.	1621P Efficacy and safety of erdafitinib in adults with pancreatic cancer and prespecified fibroblast growth factor receptor alterations (FGFRalt) in the phase II open-label: Single-arm RAGNAR trial	2023	Ann. Oncol.	https://www.embase.com/search/results?subaction=viewrecord&id=L2027894684&from=export U2 - L2027894684		Updates of previous reported study or abstract	Round II
Marshall, J. and Grose, R.	Targeting pancreatic cancer: preclinical work	2023	Pancreatology	https://www.embase.com/search/results?subaction=viewrecord&id=L2024799668&from=export U2 - L2024799668	10.1016/j.pan.2023.04.021	wrong drug	Round II
Gao, S. and Zhu, W. and Guo, X. and Liang, Y. and Wang, H. and Xu, P. and Song, Y. and Hao, J. and Wen, S. and Lv, X. and Zhou, J.	Mechanism study of surufatinib on the optimized strategy of AG plus PD-1 antibody treatment in pancreatic cancer	2023	Cancer Res.	https://www.embase.com/search/results?subaction=viewrecord&id=L641472348&from=export U2 - L641472348	10.1158/1538-7445.AM2023-2781	wrong drug	Round II
Rodon, J. and Damian, S. and Furqan, M. and Garcia-Donas, J. and Imai, H. and Italiano, A. and Spanggaard, I. and Ueno, M. and Yokota, T. and Veronese, L. and Oliveira, N. and Li, X. and Gilmartin, A. and Goyal, L.	Clinical and translational findings of pemigatinib in previously treated solid tumors with activating FGFR1-3 alterations in the FIGHT-207 study	2023	Cancer Res.	https://www.embase.com/search/results?subaction=viewrecord&id=L641474423&from=export U2 - L641474423	10.1158/1538-7445.AM2023-CT016	Updates of previous reported study or abstract	Round II
Shubham Pant, Martin H. Schuler, Gopa Iyer, Olaf Witt, Toshihiko Doi, Shukui Qin, Josep Tabernero, David A. Reardon, Christophe Massard, Liam Welsh, Iwona A. Lugowska, Omar Carranza, Kim Stuyckens, Huimin Liao, Saltanat Najmi, Constance Hammond, Shibu Thomas, Spyros Triantos, Hussein Sweiti, Yohann Loriot;	Tumor agnostic efficacy and safety of erdafitinib in patients (pts) with advanced solid tumors with prespecified fibroblast growth factor receptor alterations (FGFRalt) in RAGNAR: Interim analysis (IA) results	2022	J. Clin. Oncol.	https://www.embase.com/search/results?subaction=viewrecord&id=L638843565&from=export U2 - L638843565		Updates of previous reported study or abstract	Round II
Meric-Bernstam, F. and Sharma, M. and Sommerhalder, D. and Skeel, R.T. and El-Khoueiry, A.B. and Caswell-Jin, J.L. and Patel, P.H. and Rosen, E.	First-in-human phase 1/2 dose escalation and expansion study evaluating first-in-class eIF4A inhibitor zotatifin in patients with solid tumors	2022	J. Clin. Oncol.	https://www.embase.com/search/results?subaction=viewrecord&id=L638844006&from=export U2 - L638844006		wrong population	Round II
Tarabay, A. and Hollebecque, A. and Smolenschi, C. and Antoun, L. and Klotz, C. and Fuerea, A. and Perret, A. and Gouriou, C. and Burtin, P. and Belkhodja, H. and Boige, V. and Malka, D. and Ducreux, M.	Molecular precision medicine in pancreatic cancer: A single-center experience	2022	J. Clin. Oncol.	https://www.embase.com/search/results?subaction=viewrecord&id=L637297090&from=export U2 - L637297090		wrong population	Round II
Li, J. and Hu, K. and Huang, J. and Zhou, L. and Yan, Y. and Xu, Z.	A Pancancer Analysis of the Expression Landscape and Clinical Relevance of Fibroblast Growth Factor Receptor 2 in Human Cancers	2021	Front. Oncol.	https://www.embase.com/search/results?subaction=viewrecord&id=L634920044&from=export U2 - L634920044	10.3389/fonc.2021.644854	wrong population	Round II
Khan T and Seddon AM and Dalgleish AG and Khelwatty S and Ioannou N and Mudan S and Modjtahedi H	Synergistic activity of agents targeting growth factor receptors, CDKs and downstream signaling molecules in a panel of pancreatic cancer cell lines and the identification of antagonistic combinations: Implications for future clinical trials in pancreatic cancer.	2020	Oncology reports	https://pubmed.ncbi.nlm.nih.gov/33125153/	10.3892/or.2020.7822	wrong population	Round II

BensaÃ¯d M and Tahiri-Jouti N and Cambillau C and Viguerie N and Colas B and Vidal C and Tauber JP and EstÃ¨ve JP and Susini C and Vaysse N	Basic fibroblast growth factor induces proliferation of a rat pancreatic cancer cell line. Inhibition by somatostatin.	1992	International journal of cancer	https://pubmed.ncbi.nlm.nih.gov/1347515/	10.1002/ijc.2910500522	wrong population	Round II
OÊ¼Sullivan H and Kelleher FC and Lavelle M and McGovern B and Murphy J and Swan N and McDermott R	Therapeutic Potential for FGFR Inhibitors in SOX9-FGFR2 Coexpressing Pancreatic Cancer.	2017	Pancreas	https://pubmed.ncbi.nlm.nih.gov/28796141/	10.1097/MPA.0000000000000870	wrong outcome	Round II
Kornmann M and Lopez M and Beger H and Korc M	Expression of the IIIc Variant of FGF Receptor-1 Confers Mitogenic Responsiveness to Heparin and FGF-5 in TAKA-1 Pancreatic Ductal Cells.	2001	International journal of gastrointestinal cancer	https://pubmed.ncbi.nlm.nih.gov/12754391/		wrong outcome	Round II
Cho K and Ishiwata T and Naito Z and Uchida E	New therapeutic strategy for intractable pancreatic cancer and its fundamental research.	2010	Journal of Nippon Medical School = Nippon Ika Daigaku zasshi	https://pubmed.ncbi.nlm.nih.gov/20154464/	10.1272/jnms.77.62	wrong drug	Round II
Gnatenko, D.A. and Kopantsev, E.P. and Sverdlov, E.D.	The role of the signaling pathway FGF/FGFR in pancreatic cancer	2017	Biochem. (Moscow) Suppl. Ser. B Biomed. Chem.	https://www.embase.com/search/results?subaction=viewrecord&id=L616410528&from=export U2 - L616410528	10.1134/S1990750817020032	wrong outcome	Round II
Ciprotti, M. and Abraham, R. and Jansen, M. and Tokuhiro, S. and Hanai, M. and Oitate, M. and Shuster, D.E. and Martinez, A.	A phase I, open label, two part , safety and tolerability study of U3-1784 in patients with advanced solid tumours	2016	J. Clin. Oncol.	https://www.embase.com/search/results?subaction=viewrecord&id=L611756041&from=export U2 - L611756041		wrong population	Round II
Romeo, C. and Cassier, P. and De La FouchardiÃ¨re, A. and Pissaloux, D. and Attignon, V. and Wang, Q. and Sarabi, M. and Desseigne, F. and Guibert, P. and Perol, D. and Tredan, O. and Blay, J.-Y. and De La Fouchardiere, C.	Personalized medicine for advanced pancreas cancer: Access to treatment according to molecular profile	2016	Ann. Oncol.	https://www.embase.com/search/results?subaction=viewrecord&id=L613912152&from=export U2 - L613912152	10.1093/annonc/mdw371.57	wrong outcome	Round II
Ma, W.W. and Fetterly, G. and LeVea, C. and Zhao, Y. and Dy, G.K. and Iyer, R. and Tan, W. and Brady, W. and Whitworth, A. and Adjei, A.A.	A phase Ib study of the FGFR/VEGFR inhibitor dovitinib (D) combined with gemcitabine (G) and Capecitabine (C) in advanced pancreatic cancer patients	2015	Eur. J. Cancer	https://www.embase.com/search/results?subaction=viewrecord&id=L72067897&from=export U2 - L72067897		Updates of previous reported study or abstract	Round II
Fetterly, G.J. and Pitzonka, L. and Zhao, Y. and Dy, G.K. and Iyer, R.V. and Taubes, S. and Perez, J.R. and Culver, K.W. and Wilton, J. and Mongiardo, K. and Tan, W. and Brady, W.E. and Whitworth, A. and Adjei, A.A. and Ma, W.W.	A phase I study to assess the safety, tolerability, and PK of dovitinib (D) in combination with gemcitabine (G) and capecitabine (C) in patients with advanced solid tumors	2014	J. Clin. Oncol.	https://www.embase.com/search/results?subaction=viewrecord&id=L71524410&from=export U2 - L71524410		wrong population	Round II
Xu, J. and Wang, Y. and Ye, C. and Shen, L. and Huang, C. and Yang, L. and Sai, Y. and Mu, H.	Phase I study on safety and pharmacokinetics of sulfatinib, a selective VEGFR/FGFR dual inhibitor, in Chinese patients with advanced solid tumors	2011	Mol. Cancer Ther.	https://www.embase.com/search/results?subaction=viewrecord&id=L70609905&from=export U2 - L70609905	10.1158/1535-7163.TARG-11-C198	wrong population	Round II
Tardy, C. and Gueguen, G. and Mazoyer, S. and Morin, G. and Planel, S. and Herbert, C. and Toulas, C. and Moyal, E.C.-J. and Schaeffer, P. and Bono, F.	FGFR1-induced radioresistance of Panc02 pancreatic tumor cells is decreased by SSR128129 treatment in vitro and in vivo	2010	Cancer Res.	https://www.embase.com/search/results?subaction=viewrecord&id=L71450309&from=export U2 - L71450309	10.1158/1538-7445.AM10-1396	wrong outcome	Round II

Table S3. Characteristics of primary research.

AUTHOR	YEAR	COUNTRY	STUDY DESING	REFERENCE
Pant	2023	USA, GERMANY, SPAIN, ARGENTINA, FRANCE, ENGLAND, BELGIUM, POLAND, CHINA, JAPAN	RCT PHASE II	Pant S, Schuler M, Lyer G et al. Erdafitinib in patients with advanced solid tumours with FGFR alterations (RAGNAR): an international, single-arm, phase 2 study. Lancet Oncol. 2023; 24:925-935.
Lu	2022	USA	PRECLINICAL	Lu S, Hua J, Xu J et al. Turning towards nonimmunoreactive tumors: Evaluation of cancer-associated fibroblasts enables prediction of the immune microenvironment and treatment sensitivity in pancreatic cancer. Computational and Structural Biotechnology Journal. 2022; 20:3911-3923.
Peran	2022	SPAIN, USA	PRECLINICAL	Peran I, Vietsch EE, Yan G et al. Distinct Response of Circulating microRNAs to the Treatment of Pancreatic Cancer Xenografts with FGFR and ALK Kinase Inhibitors. Cancers. 2022; 14:1517
Saka	2017	GERMANY	RCT PHASE I	Saka H, Kitagawa C, Kogure Y et al. Safety, tolerability and pharmacokinetics of the fibroblast growth factor receptor inhibitor AZD4547 in Japanese patients with advanced solid tumours: a Phase I study. Invest New Drugs. 2017; 30:451-462.
Quintela-Fandino	2014	SPAIN, USA	RCT PHASE I	Quintela-Fandino M, Bueno MJ, Lombardia L et al. Elective activity over a constitutively active RET-variant of the oral multikinase inhibitor dovitinib: Results of the CNIO-BR002 phase I-trial. Mol Oncol. 2014; 8:1719-1728.
Taeger	2011	ITALY	PRECLINICAL	Taeger J, Moser C, Hellerbrand C et al. Targeting FGFR/PDGFR/VEGFR impairs tumor growth, angiogenesis, and metastasis by effects on tumor cells, endothelial cells, and pericytes in pancreatic cancer. Mol Cancer Ther. 2011; 10:2157-2167
Huch	2006	USA	PRECLINICAL	Huch M, Abate-Daga D, Roig JP et al. Targeting the CYP2B1/cyclophosphamide suicide system to fibroblast growth factor receptors results in a potent antitumoral response in pancreatic cancer models. Hum Gene Ther. 2006; 17:1187-200.
Mancini	2024	Italy	PRECLINICAL	Mancini V, Raffa S, Pla AF. TRPA1 Contributes to FGFR2c Signaling and to Its Oncogenic Outcomes in Pancreatic Ductal Adenocarcinoma-Derived Cell Lines. Cancers. 2024; 16:609.
Schram	2023	USA, EUROPE, KOREA, AUSTRALIA	RCT PHASE I/II	Schram AM, Subbiah V, Chih-Yi L et al. Clinical activity of lirafugratinib (RLY-4008), a highly selective FGFR2 inhibitor, in patients with advanced FGFR2-altered solid tumors: The ReFocus study. Mol Cancer Ther. 2023; https://doi.org/10.1158/1535-7163. TARG-23-IA006.

Wang	2023	UNITED KINGDOM	PRECLINI-CAL	Wang Q, Grose R. Targeting FGFR signalling as a therapeutic approach in pancreatic ductal adenocarcinoma. Abstract/Pancreatology. 2023; 23 e1-e15.
Stein	2023	USA	CASE RE-PORT	Stein L, Reeser JW, Wing MR et al. Clinical impact of FGFR inhibitors on FGFR2 positive pancreatic cancer. Cancer Res. 2023; https://doi.org/10.1158/1538-7445.AM2023-3395.
Doi	2023	JAPAN	RCT PHASE I	Doi T, Shitara K, Kojima T et al. Phase I study of the irreversible fibroblast growth factor receptor 1–4 inhibitor futibatinib in Japanese patients with advanced solid tumors. Cancer Science. 2023; 114:574-585.
Subbiah	2022	USA, KENYA	RCT PHASE II – BASKET TRIAL	Subbiah V, Iannotti NO, Gutierrez M et al. FIGHT-101, a first-in-human study of potent and selective FGFR 1-3 inhibitor pemigatinib in pan-cancer patients with FGF/FGFR alterations and advanced malignancies. Ann Oncol. 2022; 33:522-533.
Resovi	2023	ITALY	PRECLINI-CAL	Resovi A, Persichetti P, Brunelli L et al. Fibronectin fragments generated by pancreatic trypsin act as endogenous inhibitors of pancreatic tumor growth. J Exp Clin Cancer Res. 2023; 42:201
Lin	2024	USA	PRECLINI-CAL	Lin Q, Serratore A, Nu J et al. Fibroblast growth factor receptor 1 inhibition suppresses pancreatic cancer chemoresistance and chemotherapy-driven aggressiveness. Drug Resist Updat. 2024; 73:101064.
Li	2016	CHINA, USA	PRECLINI-CAL	Li J, Luo M, Wang Y et al. Celecoxib suppresses fibroblast growth factor-2 expression in pancreatic ductal adenocarcinoma PANC-1 cells. Oncol Rep. 2016; 36:1345-1352.
Lin	2024	USA	PRECLINI-CAL	Lin Q, Serratore A, Perri J et al. Expression of fibroblast growth factor receptor 1 correlatesinversely with the efficacy of single-agent fibroblast growth factor receptor-specific inhibitors in pancreatic cancer. Br J Pharmacol. 2024; 181:1383-1403.
Li	2020	CHINA	PRECLINI-CAL	Li XY, Tao H, Can J et al. Cordycepin inhibits pancreatic cancer cell growth in vitro and in vivo via targeting FGFR2 and blocking ERK. Chin J Nat Med. 2020; 18:345-355.
Li	2014	CHINA	PRECLINI-CAL	LI Z, Li X, Wang M et al. MicroRNA-100 regulates pancreatic cancer cells growth and sensitivity to chemotherapy through targeting FGFR3. Tumor Biol. 2014; 35:11751-11759.
Jiang	2018	CHINA	PRECLINI-CAL	Jiang H, Wang S, Zhou X et al. New path to treating pancreatic cancer: TRAIL gene delivery targeting the fibroblast-enriched tumor microenvironment. J Control Release. 2018;286: 254-263.
Matsuda	2014	JAPAN	PRECLINI-CAL	Matsuda Y, Yoshimura H, Suzuki T et al. Inhibition of fibroblast growth factor receptor 2 attenuates proliferation and invasion of pancreatic cancer. Cancer Sci. 2014; 105:1212-1219.
Chaudhuri	2024	USA, IRE-LAND	PRECLINI-CAL	Chaudhuri TR, Lin Q, Stachowiak EK et al. Dual-Hit Strategy for Therapeutic Targeting of Pancreatic Cancer in Patient-Derived Xenograft Tumors. Clin Cancer Res. 2024; 30:1367-1381.
Lin	2021	USA	PRECLINI-CAL	Li Q. Qian Z, Jusko WJ et al. Synergistic Pharmacodynamic Effects of Gemcitabine and Fibroblast Growth Factor Receptor Inhibitors on Pancreatic Cancer Cell Cycle

				Kinetics and Proliferation. J Pharmacol Exp Ther. 2021; 377:370-384.
Zhang	2014	USA	PRECLINI-CAL	Zhang H, Hylander BL, LeVea C et al. Enhanced FGFR signalling predisposes pancreatic cancer to the effect of a potent FGFR inhibitor in preclinical models. Br J Cancer. 2014; 110:320-329.
Sasaki	2020	JAPAN, USA	PRECLINI-CAL	Sasaki N, Gomi F, Yoshimura H et al. FGFR4 Inhibitor BLU9931 Attenuates Pancreatic Cancer Cell Proliferation and Invasion While Inducing Senescence: Evidence for Senolytic Therapy Potential in Pancreatic Cancer. Cancers. 2020; 12:2976.
Wagner	2020	USA	PRECLINI-CAL	Wagner M. Lopez ME, Cahn M et al. Suppression of fibro-blast growth factor receptor signaling inhibits pancreatic cancer growth in vitro and in vivo. Gastroenterology. 1998; 114: 798-807.
Kleef	1998	USA, GER-MANY	PRECLINI-CAL	Kleef J, Fukahi K, Lopez Me et al. Targeting of suicide gene delivery in pancreatic cancer cells via FGF receptors. Cancer Gen Ther. 2002; 9: 522-532.
Guan	2019	CHINA	PRECLINI-CAL	Guan Z, Lan H, Sun D et al. A potential novel therapy for FGFR1-amplified pancreatic cancer with bone metastasis, screened by next-generation sequencing and a patient-de-rived xenograft model. 2019; 17: 2303 -2307.
Lin	2020	USA	PRECLINI-CAL	Lin Q, Serratore A, Kandel E et al. FGFR1 expression cor-relates inversely with the in vitro and in vivo efficacy of FGFR inhibitors in pancreatic cancer. Cancer Res. 2020; https://doi.org/10.1158/1538-7445.PANCA20-PO-042
Kim	2019	KOREA, GER-MANY, USA, SWITZER-LAND	RCT PHASE I	Kim SB, Meric-Bernstam, Kalyan A et al. First-in-Human Phase I Study of Aprutumab Ixadotin, a Fibroblast Growth Factor Receptor 2 Antibodyâ€“Drug Conjugate (BAY 1187982) in Patients with Advanced Cancer. Target On-col.2019; 14: 591-601.
Coetzee	2019	UNITED KINGDOM	PRECLINI-CAL	Coetzee A, Carter E, Heward J et al. Targeting FGFR sig-naling to disrupt cellular crosstalk in pancreatic cancer. Cancer Res. 2019; https:/doi.org10.1158/15387445.sabcs18-5170.
Kuboki	2017	JAPAN	RCT PHASE II – BASKET TRIAL	Kuboki Y, Matsubara N, Bando H et al. First-in-human (FIH) study of TAS-120, a highly selective covalent oral fi-broblast growth factor receptor (FGFR) inhibitor, in pa-tients (pts) with advanced solid tumors. https://doi.org/10.1093/annonc/mdx367.006.
Chaudur i	2017	USA	PRECLINI-CAL	Chauduri TR, Lin Q, Straubinger NL et al. FGFR1 inhibi-tor reverses Smoothened inhibition-induced epithelial-tomesenchymal transition in pancreatic ductal adenocarci-noma. Cancer Res. 2017; https://doi.org/10.1158/1538-7445.AM2017-4071.
Lin	2017	USA	PRECLINI-CAL	Lin Q; Chaudhuri TR; Straubinger NL et al. FGFR inhibi-tors enhance gemcitabine sensitivity in pancreatic ductal adenocarcinoma. Cancer Res. 2017; https://doi.org/10.1158/1538-7445.AM2017-4072.
Lai	2018	TAIWAN	PRECLINI-CAL	Lai SW, Bamodu OA, Tsai WC et al. The therapeutic tar-geting of the FGFR1/Src/NF-Î°B signaling axis inhibits pancreatic ductal adenocarcinoma stemness and oncogenic-ity. Clin Exp Metastasis. 2018; 35:663-677.

Kleef	2004	USA, GERMANY	PRECLINICAL	Kleeff J, Kothari NH, Friess H et al. Adenovirus-mediated transfer of a truncated fibroblast growth factor (FGF) type I receptor blocks FGF-2 signaling in multiple pancreatic cancer cell lines. Pancreas. 2004; 28:25-30.
Lai	2018	TAIWAN	PRECLINICAL	Lai SW, Bamodu OA, Chen JH et al. Targeted PARP Inhibition Combined with FGFR1 Blockade is Synthetically Lethal to Malignant Cells in Patients with Pancreatic Cancer. Cells. 2020; 9:911.
NG	2022	USA	CASE REPORT	Ng CF, Glaspy J, Placencio-Hickok VR et al. Exceptional Response to Erdafitinib in FGFR2-Mutated Metastatic Pancreatic Ductal Adenocarcinoma. J Natl Compr Canc Netw. 2022; 20:1076-1079.
Poon	2021	SINGAPORE	CASE REPORT	Poon D and Tan MH and Khor D. Stage 4 pancreatic adenocarcinoma harbouring an FGFR2-TACC2 fusion mutation with complete response to erdafitinib a pan-fibroblastic growth factor receptor inhibitor. BMJ Case Rep. 2021; 14:e244271.
Helal	2022	FRANCE	CASE REPORT	Helal C, Valéry M, Ducreux M et al. FGFR2 fusion in metastatic pancreatic ductal adenocarcinoma: Is there hope? Eur J Cancer. 2022; 176:168-170.
Tolcher	2016	USA	RCT PHASE I	Tolcher AW, Papadopoulos KP, Patnaik A. A phase I, first in human study of FP-1039 (GSK3052230), a novel FGF ligand trap, in patients with advanced solid tumors. Ann Oncol. 2016; 27:526-32.
Ishiwata	2016	JAPAN	PRECLINICAL	IshiwataT, Yoshimura H; Matsuda Y et al. Fibroblast growth factor receptor-4 (FGFR-4) as a novel therapeutic target for pancreatic cancer. Cancer Res. 2016; https://doi.org/10.1158/1538-7445.AM2016-4577.
Zhang	2012	USA	PRECLINICAL	Zhang, H. and Hylander, B. and LeVea et al. Effects of dovitinib on fibroblast growth factor receptor-2 (FGFR2) expressing pancreatic cancer. Br J Cancer. 2014; 110:320-9.
Ishiwata	2011	JAPAN	PRECLINICAL	Ishiwata T, Matsuda, Y, Kawahara K et al. Fibroblast growth factor receptor 2 as a novel therapeutic target for cancer cell growth and angiogenesis in human pancreatic cancer. Cancer Res. 2011; https://doi.org/10.1158/1538-7445.AM2011-1204
Rodon	2024	USA	RCT PHASE II - BASKET TRIAL	Rodon J, Damian S, Furqan M et al.Pemigatinib in previously treated solid tumors with activating FGFR1-FGFR3 alterations: phase 2 FIGHT-207 basket trial. Nat Med. 2024;doi: 10.1038/s41591-024-02934-7.
Chae	2020	USA	RCT PHASE II - BASKET TRIAL	Chae KC, Hong F, et al. Vaklavas C. Phase II Study of AZD4547 in Patients With Tumors Harboring Aberrations in the FGFR Pathway: Results From the NCI-MATCH Trial (EAY131) Subprotocol W. J Clin Oncol. 2020; 38:2407-2417.

Table S4. Primary researches*: population characteristics.

First Author	Year	Type of studies	FGFR alteration type	Reporting only pancreatic cancer patients (Y/N)	Drug	FGFR exclusion	Comutations (Y/N)	Pt n (%)	Age	ECOG-PS	Stage	Primary Outcomes
Pant S	2023	RCT phase II	FGFR1–4 alterations (mutations or fusions)	N	Erdafitinib	FGFR gatekeeper and resistance alterations	N	18 (8%)	≥12 years	0-1	IV	ORR
Rodon J	2024	RCT phase II	FGFR1–FGFR3 fusions/rearrangements or mutations	N	Pemigatinib	FGFR kinase domain SNVs	N	8 (7.5%)	≥18 years	0-2	IV	ORR
Chae Y K	2020	RCT phase II	FGFR 1-3 aberrations	N	Fexagratinib	N	Y	1 (2.1%)	≥25 years	NA	IV	ORR
Schram	2023	RCT phase I/II	FGFR2 alterations (fusions/rearrangements, amplifications, mutations)	N	Lirafugratinib	N	N	5 (5.1%)	NA	NA	IV	ORR, DOR, safety
Kuboki	2017	RCT phase I/II	FGFR abnormalities	N	Futibatinib	N	N	3 (8.3%)	NA	NA	IV	safety, tolerability, efficacy
Subbiah V	2022	RCT phase I/II	amplifications, mutations, or translocations of FGFR1, FGFR2, or FGFR3, or alteration of FGF1 through FGF23.	N	Pemigatinib	N	N	4 (3.1%)	≥18 years	0-2	IV	safety, tolerability
Saka H	2017	RCT phase I	FGFR-amplified	N	Fexagratinib	N	N	1 (3.3%)	≥25 years	0-1	IV	safety, tolerability
Quintela-Fandino M	2014	RCT phase I	FGFR1-2-3 mutations/amplifications	N	dovitinib	N	Y (RET)	1 (9%)	NA	0-2	IV	safety
Doi T	2023	RCT phase I	FGFR abnormality	N	futibatinib	N	N	3 (3.6%)	≥20 years	0-1	IV	safety
Kim SB	2019	RCT phase I	FGFR2-positive	N	aprutumab ixadotin	N	N	1 (5%)	≥18 years	0-1	IV	safety, tolerability
Tolcher AW	2016	RCT phase I	FGFR1 expression, amplification	N	FP-1039	N	N	1 (2.6%)	≥18 years	0-2	IV	safety, tolerability
Ng CF	2022	Case reports	FGFR2 rearrangement intron 17	Y	Erdafitinib	NA	Y	1	28	NA	IV	NA
Poon D	2021	Case reports	FGFR2-TACC2 fusion	Y	Erdafitinib	NA	Y	1	68	2	IV	NA
Helal C	2022	Case reports	FGFR2 fusions	Y	FGFR2 specific inhibitor	NA	Y	4	adult	NA	IV	NA
Stein L	2023	Case reports	FGFR2 fusion	Y	pemigatinib(2), pazopanib, ponatinib	NA	Y (KRAS)	4	NA	NA	IV	clinical outcome

*excluded preclinical publications; ORR: Overall Response Rate; DOR: Duration Of Response; PT: Patient; Y/N: YES/NO; NA: Not Applicable

Table S5. FGRF receptor alteration characteristics in preclinical studies.

Authors	Year	Receptor
Wagner M	1998	FGFR1
Kleeff J	2004	FGFR1
Huch M	2006	FGFR1
Chaudhuri RT (ABS)	2017	FGFR1
Lin Q (ABS)	2017	FGFR1
Lai S-W	2018	FGFR1
Guan Z	2019	FGFR1
Coetzee A	2019	FGFR1
Lin Q (ABS)	2020	FGFR1
Lai S-W	2020	FGFR1
Peran I	2022	FGFR1
Kleef J	2022	FGFR1
Wang Q (ABS)	2023	FGFR1
Resovi A	2023	FGFR1
Lin Q	2024	FGFR1
Chaudhuri RT	2024	FGFR1
Taeger J	2011	FGFR1-2-3
Lin Q	2024	FGFR1-2-3
Jiang H	2018	FGFR1-2-3-4
Lin Q	2021	FGFR1-2-3-4
Ishiwata T (ABS)	2011	FGFR2

Zhang H (ABS)	2012	FGFR2
Matsuda Y	2014	FGFR2
Zhang H	2014	FGFR2
Li J	2016	FGFR2
Kim SB	2019	FGFR2
Li XY	2020	FGFR2
Mancini V	2024	FGFR2
Li Z	2014	FGFR3
Ishiwata T (ABS)	2016	FGFR4
Sasaki N	2020	FGFR4
Lu S	2022	FGFR4

Table S6. Included grey literature characteristic from clinicaltrial.gov.

NCT Number	Study Status	Conditions	Interventions	Primary Outcome Measures	Age	Phase
NCT04003623	CLOSED	Advanced or Metastatic Solid Tumors	Pemigatinib	ORR	ADULT, OLDER_ADULT	PHASE2
NCT06022289	RECRUITING	Solid Tumor	Pemigatinib	PFS2/PFS1	ADULT, OLDER_ADULT	PHASE2
NCT03825484	APPROVED_FOR_MARKETING	Advanced Cancers	Erdafitinib	NA	CHILD, ADULT, OLDER_ADULT	POST MARKET
NCT04962867	ACTIVE_NOT_RECRUITING	Advanced or Recurrent Solid Tumors	E7090	ORR	ADULT, OLDER_ADULT	PHASE2
NCT04189445	ACTIVE_NOT_RECRUITING	Advanced or Metastatic Solid Tumor|Advanced or Metastatic Gastric or Gastroesophageal Cancer|Myeloid or Lymphoid Neoplasms (MLN)	Futibatinib	ORR	ADULT, OLDER_ADULT	PHASE2
NCT03929965	UNKNOWN	Advanced Solid Tumor	Anlotinib	ORR	ADULT, OLDER_ADULT	NA
NCT02846766	WITHDRAWN	Cancer	Lenvatinib	ORR	ADULT, OLDER_ADULT	PHASE2
NCT02160041	TERMINATED	Solid Tumor|Hematologic Malignancies	BGJ398 (Infingratinib)	ORR and clinical benefit rate	ADULT, OLDER_ADULT	PHASE2
NCT05651672	RECRUITING	Gastrointestinal Cancer	Pemigatinib	ORR	ADULT, OLDER_ADULT	PHASE2
NCT06308822	ACTIVE_NOT_RECRUITING	Advanced Lymphoma|Advanced Malignant Solid Neoplasm|Refractory Lymphoma|Refractory Malignant Solid Neoplasm|Refractory Multiple Myeloma	Erdafinitinb	ORR	ADULT, OLDER_ADULT	PHASE2
NCT02747797	WITHDRAWN	Advanced Cancer	Lucitanib	ORR	ADULT, OLDER_ADULT	PHASE2
NCT06351371	ACTIVE_NOT_RECRUITING	Malignant Solid Neoplasm	Erdafitinib	ORR	ADULT, OLDER_ADULT	PHASE3
NCT04083976	ACTIVE_NOT_RECRUITING	Advanced Solid Tumor	Erdafitinib	ORR	CHILD, ADULT, OLDER_ADULT	PHASE2
NCT05325866	RECRUITING	Solid Tumors	Bemarituzumab	Part 1: (DLT), Part 2: ORR	ADULT, OLDER_ADULT	PHASE1|PHASE2
NCT04233567	ACTIVE_NOT_RECRUITING	Advanced Malignant Solid Neoplasm|Cholangiocarcinoma|Metastatic Malignant Solid	Infigratinib	ORR	ADULT, OLDER_ADULT	PHASE2

		Neoplasm|Refractory Malignant Solid Neoplasm				
NCT01948297	TERMINATED	Solid Tumours	Debio1347 (CH5183284)	Part A: PDLTs, Part B: SAEs	ADULT, OLDER_ADULT	PHASE1
NCT05372120	RECRUITING	Advanced Solid Tumor	ICP-192	ORR	ADULT, OLDER_ADULT	PHASE2
NCT05019794	UNKNOWN	Gastric Cancer|Gastroesophageal Junction Adenocarcinoma|Solid Tumor	Infigratinib	ORR	ADULT, OLDER_ADULT	PHASE2
NCT06302621	RECRUITING	Advanced Solid Tumor|Unresectable Solid Tumor|Metastatic Solid Tumor|Cholangiocarcinoma	Afatinib|DRUG: Pemigatinib	MTD, ORR	ADULT, OLDER_ADULT	PHASE1
NCT02272998	ACTIVE_NOT_RECRUITING	Malignant Neoplasm	ponatinib hydrochloride	ORR	ADULT, OLDER_ADULT	PHASE2
NCT02368951	TERMINATED	Medical Oncology	BAY1187982	MTD	ADULT, OLDER_ADULT	PHASE1
NCT05559775	RECRUITING	Gastrointestinal Cancer	Pemigatinib Pill	ORR	ADULT, OLDER_ADULT	PHASE2
NCT03834220	TERMINATED	Solid Tumor	Debio 1347	ORR	ADULT, OLDER_ADULT	PHASE2
NCT03210714	ACTIVE_NOT_RECRUITING	Advanced Malignant Solid Neoplasm|Recurrent Childhood Ependymoma|Recurrent Childhood Malignant Germ Cell Tumor|Recurrent Childhood Medulloblastoma|Recurrent Childhood Non-Hodgkin Lymphoma|Recurrent Childhood Osteosarcoma|Recurrent Childhood Rhabdomyosarcoma|Recurrent Childhood Soft Tissue Sarcoma|Recurrent Ewing Sarcoma/Peripheral Primitive Neuroectodermal Tumor|Recurrent Hepatoblastoma|Recurrent Langerhans Cell Histiocytosis|Recurrent Malignant Glioma|Recurrent Malignant Solid Neoplasm|Recurrent	Erdafitinib	ORR	CHILD, ADULT	PHASE2

		Neuroblastoma|Recurrent Primary Central Nervous System Neoplasm|Recurrent Rhabdoid Tumor|Refractory Childhood Malignant Germ Cell Tumor|Refractory Childhood Osteosarcoma|Refractory Childhood Rhabdomyosarcoma|Refractory Childhood Soft Tissue Sarcoma|Refractory Ependymoma|Refractory Ewing Sarcoma/Peripheral Primitive Neuroectodermal Tumor|Refractory Hepatoblastoma|Refractory Langerhans Cell Histiocytosis|Refractory Malignant Glioma|Refractory Malignant Solid Neoplasm|Refractory Medulloblastoma|Refractory Neuroblastoma|Refractory Non-Hodgkin Lymphoma|Refractory Primary Central Nervous System Neoplasm|Refractory Rhabdoid Tumor|Wilms Tumor				
NCT05242822	ACTIVE_NOT_RECRUITING	Solid Tumor, Adult|Intrahepatic Cholangiocarcinoma|Urothelial Carcinoma	KIN-3248	Part A: DLTs, Part B: ORR, DOR, PFS	ADULT, OLDER_ADULT	PHASE1

ORR: OVERALL RESPONCE RATE

DOR: DURATION OF RESPONCE

DLT: DOSE LIMITING TOXICITIES

PFS: PROGRESSION FREE SURVIVAL

MTD: MAXIMUN TOLLERATED DOSE

SAE: SERIOUS ADVERSE EVENT

NA: NOT AVAILABLE
